# Gene expression profiles during postnatal development of the liver and pancreas in giant pandas

**DOI:** 10.18632/aging.103783

**Published:** 2020-08-15

**Authors:** Jinnan Ma, Fujun Shen, Lei Chen, Honglin Wu, Yan Huang, Zhenxin Fan, Rong Hou, Bisong Yue, Xiuyue Zhang

**Affiliations:** 1 Key Laboratory of Bio-resources and Eco-environment, Ministry of Education, College of Life Sciences, Sichuan University, Chengdu 610065, China; 2 The Sichuan Key Laboratory for Conservation Biology of Endangered Wildlife, Chengdu Research Base of Giant Panda Breeding, Chengdu 610081, China; 3 Sichuan Key Laboratory of Conservation Biology on Endangered Wildlife, College of Life Sciences, Sichuan University, Chengdu 610065, China; 4 China Conservation and Research Center for the Giant Panda, Wolong, Sichuan 623006, China

**Keywords:** giant panda, gene expression pattern, liver, pancreas, development

## Abstract

Giant pandas are unique Carnivora with a strict bamboo diet. To investigate the molecular mechanism of giant panda nutrient metabolism from newborn to adult, the gene expression profiles of giant panda liver and pancreas tissues collected from three important feeding stages were investigated using RNA-seq. We found a total of 3,211 hepatic and 3,343 pancreatic differentially expressed genes (DEGs) from three comparisons between suckling and no feeding, adult and no feeding, and adult and suckling groups. Few differences in gene-expression profiles were exhibited between no feeding and suckling groups in both tissues. GO and KEGG analyses were performed to further understand the biological functions of the DEGs. In both the liver and pancreas, genes related mainly to cell cycle processes were highly up-regulated in newborn samples whereas genes related to metabolism and immunity were up-regulated in adult giant pandas. The high expression of metabolism-related genes in adult samples probably helps to fulfill the metabolic function requirements of the liver and pancreas. In contrast, several vital genes involved in cholesterol metabolism and protein digestion and absorption were over-expressed in newborn samples. This may indicate the importance of cholesterol metabolism and protein digestion and absorption processes in giant panda infancy.

## INTRODUCTION

Postnatal development is usually accompanied by changes in several physiological functions and nutritional environment. In utero, the fetus receives nutrients transported by the placenta for growth and oxidative metabolism [1]. Meanwhile, fetal waste products are dissipated into the mother organism through the placenta for disposal [2]. The substrates supplemented by the mother ceases immediately on birth and the newborn requires nutrition from its mother’s milk. Milk is replaced by solid food towards the end of the suckling period, which usually has lower fat and protein and higher carbohydrates than milk [3]. The successful adaptation to the changes in the nutritional environment requires crucial adjustments of metabolic function in metabolic organs [3–6]. Furthermore, the dietary change appears to be very important for the postnatal development of metabolic organs [6].

Giant pandas are unique within Carnivora because they are specialized herbivores with a strict bamboo diet. However, their digestive tracts have not evolved the long tortuous twists and turns that facilitate the digestion of cellulose [7]. Understanding how giant pandas absorb nutrients from a low-nutrition bamboo diet has long interested evolutionary biologists. Previous studies have highlighted some interesting findings of the nutrient utilization of giant pandas [8, 9], but research on the molecular mechanisms of nutrient metabolism are rare [10]. In addition to genome research, studies employing transcriptome-wide mining of the functional genes in nutrient metabolism are needed. Exploring the expression changes of metabolism-related genes as neonatal giant pandas transition from a high protein and lipid milk to low protein and lipid bamboo will help us to comprehensively understand the characteristics of nutrition utilization in giant pandas.

In this study, we sequenced the RNA-seq of giant panda liver and pancreas tissue at three different stages using Next-Generation sequencing. The changes of differentially expressed genes (DEGs) between three-life stages in liver and pancreas were investigated and the dynamic changes of the DEGs involved in nutrient digestion and metabolism pathways were analyzed in detail.

## RESULTS

### Overview of the landscape of the giant panda liver and pancreas transcriptome

**Figure 1:**
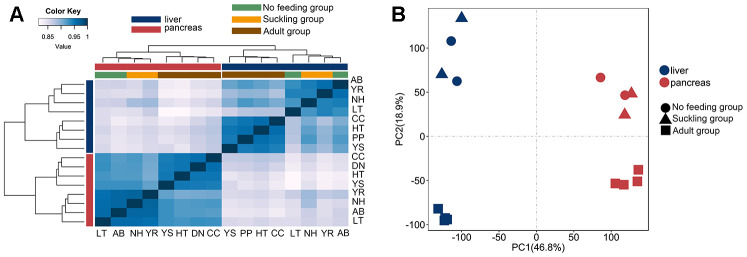
**Clustering and PCA analyses of the expression for liver and pancreas samples.** (**A**) Clustering of liver and pancreas samples based on log-transformed normalized CPM expression values. Distance between samples was measured by Spearman's rank correlation coefficient. (**B**) PCA of the log-transformed normalized CPM expression levels of all liver and pancreas samples. Species are represented by point shape. Tissues are represented by different colors.

To investigate the gene expression changes in the postnatal giant panda liver and pancreas, 8 liver and 8 pancreas RNA-seq libraries were constructed. These RNA-seq libraries generated 2.0 to 3.5 million clean paired-end reads (Table 1). We aligned each of the 16 cDNA libraries onto the giant panda reference genome separately and found that the final efficiency of RNA-seq read alignments ranged from 69.20 to 96.72%. Hierarchical clustering analysis based on Spearman’s correlation coefficients was performed to investigate gene expression patterns across all tissues. Liver and pancreas samples were separated into different groups, indicating a universal tissue-specific expression pattern (Figure 1A). The mRNA expression profiles of both tissues showed a similar clustering pattern. Two major branches were defined in both the liver and pancreas: one representing no feeding and suckling, and the other representing the adult group. This clustering pattern may indicate relatively large differences in gene expression profiles between adult groups and no feeding and suckling groups, since postnatal developmental stages would share a more consistent expression profile. Principal components analysis also clearly verified these findings (Figure 1B).

**Table 1:** Summary of sequencing of transcriptome.

**Sample ID**	**Organ**	**Developmental groups**	**Sex**	**Read length (bp)**	**Layout**	**Total clean reads**	**% aligned reads**	**SRA accessions**
NH	Liver	No feeding	Female	100	Paired	24136987	94.96	SRR11445254
YR	Liver	No feeding	Male	150	Paired	27075636	95.26	SRR11445253
AB	Liver	Suckling	Male	150	Paired	24005186	93.53	SRR11445252
LT	Liver	Suckling	Female	150	Paired	27213030	95.72	SRR11445251
PP	Liver	Adult	Male	150	Paired	21302638	95.83	SRR11301096
YS	Liver	Adult	Male	150	Paired	22807879	92.82	SRR11301095
CC	Liver	Adult	Male	150	Paired	20345289	94.81	SRR11301092
HT	Liver	Adult	Female	150	Paired	32302526	93.44	SRR11301091
NH	Pancreas	No feeding	Female	100	Paired	21440505	94.69	SRR11445250
YR	Pancreas	No feeding	Male	150	Paired	35010545	96.72	SRR11445249
AB	Pancreas	Suckling	Male	150	Paired	25356005	92.73	SRR11445248
LT	Pancreas	Suckling	Female	150	Paired	28997077	95.98	SRR11445247
DN	Pancreas	Adult	Male	150	Paired	25856130	72.22	SRR11301090
YS	Pancreas	Adult	Male	150	Paired	26842343	71.05	SRR11301089
CC	Pancreas	Adult	Male	150	Paired	20885045	70.31	SRR11301088
HT	Pancreas	Adult	Female	150	Paired	21181582	69.20	SRR11301087

### Transcriptomic profiles of the liver

We collected 8 liver samples from three postnatal developmental stages, and there were 15,251 qualified coding genes with average normalized CPM greater than 0 in a given stage. The top 10 of the most highly expressed genes in different stages were analyzed (Supplementary Table 1). Specifically, four genes (*ALB*: albumin, *ENSAMEG00000006318*: novel gene, *ATP6*: ATP synthase F0 subunit 6 and *ND4*: NADH dehydrogenase subunit 4) were found highly co-expressed in the three developmental stages, which suggested important roles for these genes in the growth and development of giant panda liver tissue.

**Figure 2:**
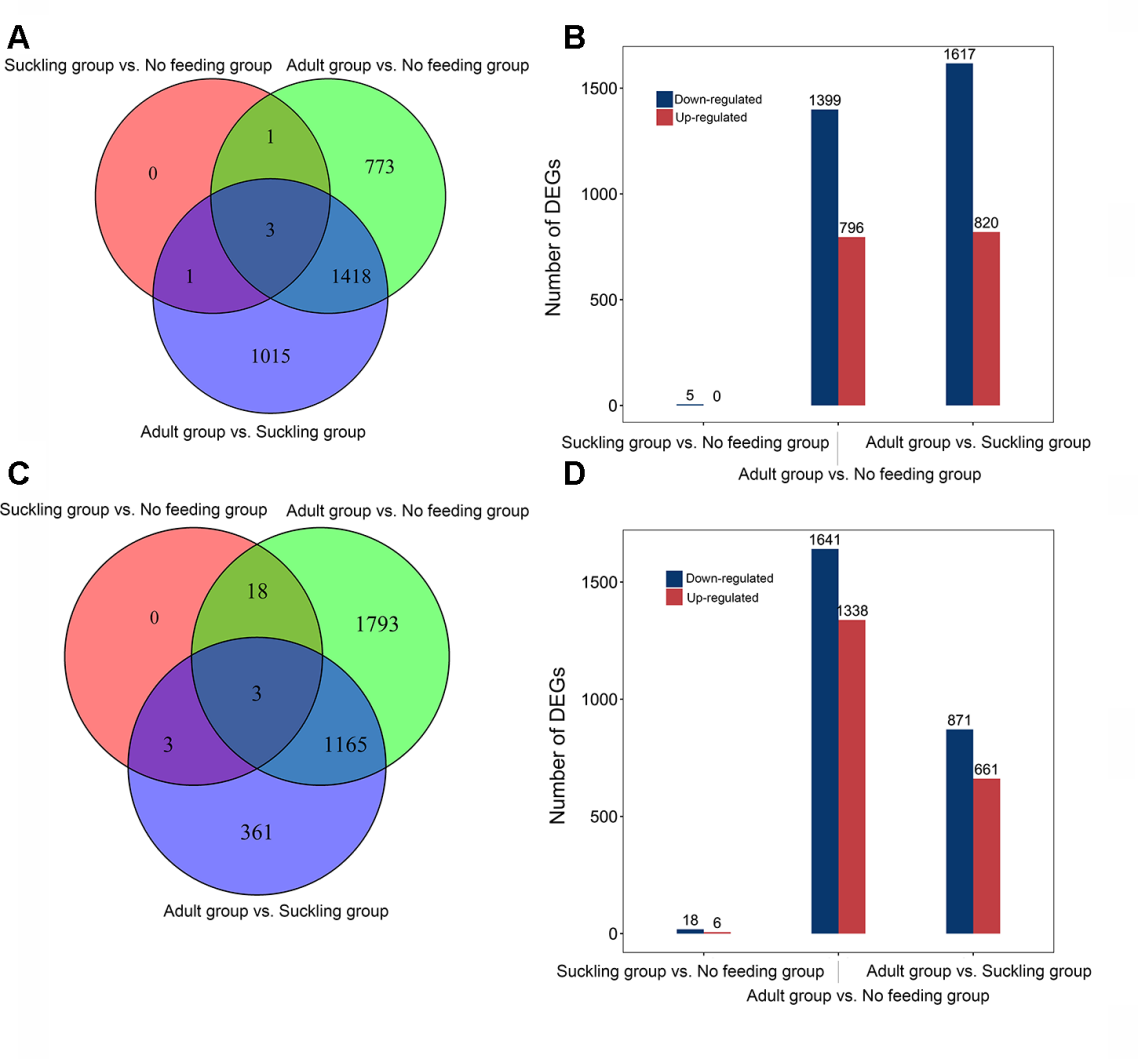
**Distribution of differentially expressed genes of giant panda liver and pancreas at different postnatal stages.** (**A**) Venn diagram indicating DEGs that are shared between different comparisons in the liver. (**B**) The number of up-and down-regulated DEGs in different comparisons in the liver. (**C**) Venn diagram indicating DEGs that are shared between different comparisons in the pancreas. (**D**) The number of up-and down-regulated DEGs in different comparisons in the pancreas.

We conducted a pairwise comparison to identify DEGs between the three life stages. A total of 3,211 DEGs were identified (Figure 2A). We found five DEGs (*ENSAMEG00000013554*, *ENSAMEG00000013541*, *HAMP*, *SEMA5A*, *SLC47A2*) between the no feeding and suckling groups, and all DEGs were down-regulated in the suckling group compared to the no feeding group. We identified 2,437 DEGs in the adult group when compared to the suckling group, including 820 up-regulated and 1617 down-regulated DEGs. There were 796 up-regulated genes and 1,399 down-regulated genes in the adult group when compared to the no feeding group (Figure 2B). The distribution of DEGs showed that the proportion of down-regulated genes was larger than up-regulated genes in these three pair-wise comparisons.

### Enrichment analysis of DEGs in the liver

GO and KEGG analyses were performed to further understand the biological functions of the genes within the significant gene expression profiles between three life stages sequentially. The terms (ordered by the corrected P-value) of the enrichment results are shown in Supplementary Tables 2–9. DEGs between the suckling and no feeding groups were not significantly enriched to any terms. Compared to the suckling group, genes down-regulated in the adult group were mainly involved in cell cycle progression, such as DNA replication (GO:0006260), chromosome segregation (GO:0007059), mitotic cell cycle (GO:0000278), and Cell cycle (PATH: aml04110), and no metabolism-related terms were enriched. The GO and KEGG terms related to the immune system were significantly enriched for up-regulated genes in the adult group. This may indicate that the immunity of a newborn giant panda is weak and gradually improves with the development of the immune system. We also found that up-regulated genes were enriched in terms involved in nutrient metabolism, including long-chain fatty acid-CoA ligase activity (GO:0004467), Glycine, serine and threonine metabolism (PATH:aml00260), Starch and sucrose metabolism (PATH:aml00500), Arachidonic acid metabolism (PATH:aml00590), Linoleic acid metabolism (PATH:aml00591), Tryptophan metabolism (PATH:aml00380), and Protein digestion and absorption (PATH:aml04974). The DEGs involved in cell cycle progression identified between the no feeding and adult groups, were also highly expressed in the no feeding group samples, and Cell cycle (PATH:aml04110) and DNA replication (GO:0006260) were the most significant KEGG pathway and GO category, respectively. Thirty-five GO terms and 42 KEGG pathways were found in up-regulated DEGs in the adult group compared to liver samples from the no feeding group. Similar to the adult and suckling groups’ comparison, many nutrient utilization-related terms were obtained from the adult-no feeding enriched terms (Table 2).

**Table 2:** Metabolism-related terms enriched by DEGs in giant panda liver at different developmental stages.

**Functional classes**	**Comparison**	**Terms**	**Adjust P**	**Gene number**
carbohydrate metabolism and energy production	Up-regulated in adult group when compared to suckling group	Starch and sucrose metabolism [PATH:aml00500]	0.00308	8
carbohydrate metabolism and energy production	Up-regulated in adult group when compared to no feeding group	electron transport chain [GO:0022900]	0.0312	9
carbohydrate metabolism and energy production	Up-regulated in adult group when compared to no feeding group	Oxidative phosphorylation [PATH:aml00190]	4.23E-06	21
amino acid and protein metabolism	Up-regulated in adult group when compared to suckling group	negative regulation of endopeptidase activity [GO:0010951]	9.39E-05	16
amino acid and protein metabolism	Up-regulated in adult group when compared to suckling group	endopeptidase inhibitor activity [GO:0004866]	0.00238	6
amino acid and protein metabolism	Up-regulated in adult group when compared to suckling group	pyridoxal phosphate binding [GO:0030170]	0.0132	9
amino acid and protein metabolism	Up-regulated in adult group when compared to suckling group	proteolysis [GO:0006508]	0.0160	38
amino acid and protein metabolism	Up-regulated in adult group when compared to suckling group	Glycine, serine and threonine metabolism [PATH:aml00260]	0.000277	11
amino acid and protein metabolism	Up-regulated in adult group when compared to suckling group	Tryptophan metabolism [PATH:aml00380]	0.0403	7
amino acid and protein metabolism	Up-regulated in adult group when compared to suckling group	Protein digestion and absorption [PATH:aml04974]	0.0378	11
lipid metabolism	Up-regulated in adult group when compared to no feeding group	lipid biosynthetic process [GO:0008610]	0.0169	5
lipid metabolism	Up-regulated in adult group when compared to no feeding group	Steroid hormone biosynthesis [PATH:aml00140]	4.57E-05	11
lipid metabolism	Up-regulated in adult group when compared to no feeding group	Linoleic acid metabolism [PATH:aml00591]	0.000201	8
lipid metabolism	Up-regulated in adult group when compared to no feeding group	Arachidonic acid metabolism [PATH:aml00590]	0.00311	11
lipid metabolism	Up-regulated in adult group when compared to suckling group	steroid metabolic process [GO:0008202]	0.0368	5
lipid metabolism	Up-regulated in adult group when compared to suckling group	long-chain fatty acid-CoA ligase activity [GO:0004467]	0.0442	4
lipid metabolism	Up-regulated in adult group when compared to suckling group	Steroid hormone biosynthesis [PATH:aml00140]	0.000277	11
lipid metabolism	Up-regulated in adult group when compared to suckling group	Arachidonic acid metabolism [PATH:aml00590]	0.00411	10
lipid metabolism	Up-regulated in adult group when compared to suckling group	Linoleic acid metabolism [PATH:aml00591]	0.0146	6

### Identification of metabolism-related DEGs in the liver

**Figure 3:**
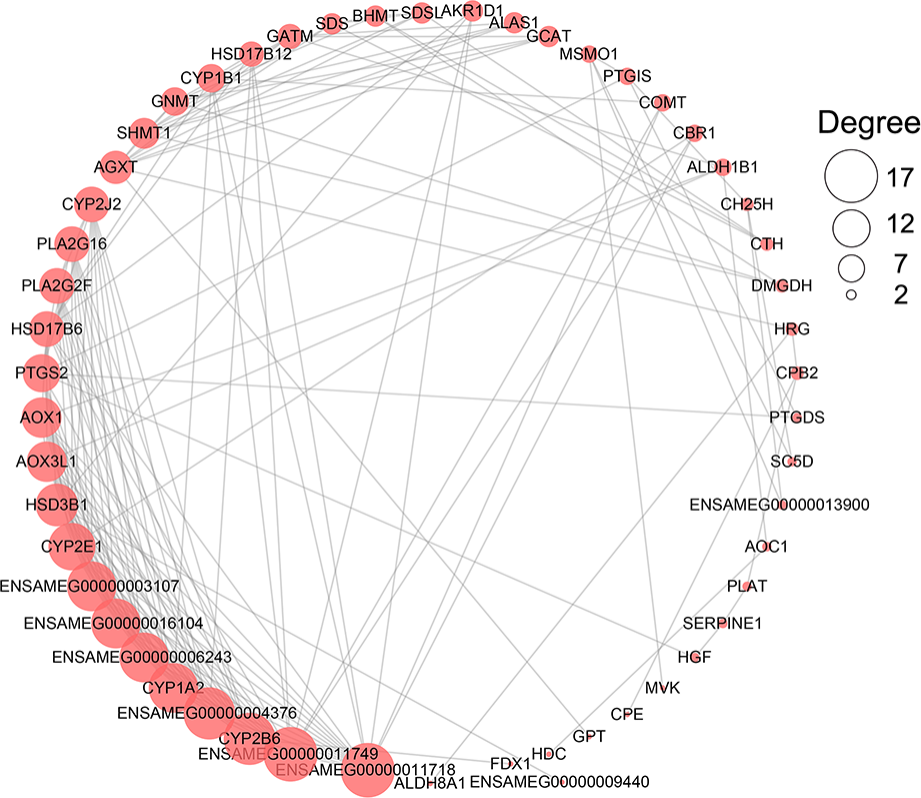
**The network of PPI analysis of metabolism-related DEGs in the liver.** The sub-network contained hub genes was extracted. The size of the circle represents the degree level of node gene. The bigger the circle, the more degree of node gene.

According to the enrichment result, the metabolism-related terms identified from three development stages were grouped into three functional classes, being carbohydrate metabolism and energy production, amino acid and protein metabolism, and lipid metabolism (Table 2). We found 146 metabolism-related DEGs involved in these categories (Supplementary Table 10 and Supplementary Figure 1). A protein-protein interaction analysis showed *ENSAMEG00000011749* (UDP-glucuronosyltransferase 2C1) and *ENSAMEG00000011718* (UDP-glucuronosyltransferase 2B31) were at the key position of the interaction network with the highest degree (Figure 3).

More metabolism-related DEGs were analyzed. Eight genes (*ENSAMEG00000001684*, *GBA3*, *SI*, *ENPP3*, *GBE1*, *MGAM*, *GCK*, *PYGM*) were involved in carbohydrate metabolism, and they participated in fundamental biochemical processes that ensured the supply of energy to living cells. Furthermore, a series of genes encoded the subunit of the ubiquinol-cytochrome c oxidoreductase complex (*UQCRB*, *UQCRQ*), the cytochrome c oxidase (*COX6B2*, *COX17*, *COX7B*, *COX7C*, *COX11*), and the ubiquinone oxidoreductase (e.g., *NDUFA1*, *NDUFS4*, *NDUFA12*, *NDUFB6*) and were also over-expressed in the liver at the adult stage (Supplementary Table 10). Fat biosynthesis occurs mainly in the liver and plays a key role in providing fat that organisms can use to obtain energy. Our results showed that the DEGs enriched in the liver lipid biosynthetic process were all up-regulated in the adult group when compared to no feeding and suckling groups, such as *CYP1A2*, *CYP2B6*, *SC5D*, and *ACSL1*. Also, fatty acid metabolism genes (*SLC27A2*, *PLA2G2F*, *ACSBG1*) showed the same gene expression pattern as lipid biosynthesis genes that are involved in energy generation and storage in animals [16]. Similarly, amino acid and protein metabolism-related genes that included many peptidases and the major proteases (e.g., *CPE*, *CPB2*, *ENPEP*, *PHEX*, *HTRA1*, *PCSK6*, *PCSK5*, *CTSV*, *CTSB*) were all highly expressed at the adult stages in liver (Supplementary Table 10).

### Transcriptomic profiles of the pancreas

With the same experimental design, we tested 8 pancreas samples and identified 15,851 coding genes with average normalized CPM greater than 0 in each stage. The shared top 10 highly expressed genes across all stages were *ENSAMEG00000014546*, *PLA2G1B,* and *CPA1* (Supplementary Table 11). These three genes encoded proteins that have protease activity and play an essential function in the pancreas. Comparisons of gene expression were performed in the pancreas to evaluate the expression of development-dependent DEGs after birth. We obtained a total of 3,343 DEGs from three comparisons, equating to 6, 1,338, and 661 up-regulated DEGs, and 18, 1,641, and 871 down-regulated DEGs in the suckling and no feeding comparison, the adult and no feeding comparison, and the adult and suckling comparison, respectively (Figure 2D). There were 1,168 DEGs shared between the adult and no feeding groups and the adult and suckling groups. We identified 1,793 and 361 unique DEGs in the adult and no feeding comparison and the adult and suckling comparison, respectively (Figure 2C). *AZGP1*, *FN1*, and *FBN2* were shared by all the groups. *FN1* and *FBN2* were consistently down-regulated during development but up-regulated for *AZGP1*. *AZGP1* plays important functions in promoting glucose utilization and regulating insulin sensitivity [11]. The continuous increase in expression of *AZGP1* may reflect the gradual improvement of pancreas metabolic function after birth. *FN1* and *FBN2* are ECM proteins associated with tissue morphogenesis in the early stages [12, 13]. High expression of those two genes in the early postnatal days may be important for tissue formation and maturation during pancreas development.

### Enrichment analysis of DEGs in the pancreas

The enrichment of GO and KEGG pathways for the significantly up- and down-regulated gene lists from each comparison in the pancreas was also examined (Supplementary Tables 12–22). When comparing the suckling and no feeding groups only four functional terms related to protein digestion were enriched in the up-regulated genes in the suckling group samples, being serine-type peptidase activity (GO:0008236), serine-type endopeptidase activity (GO:0004252), peptidase activity (GO:0008233), and proteolysis (GO:0006508) (Table 3). No KEGG pathway was significantly enriched. Down-regulated genes in the suckling group were enriched in seven GO categories and 2 KEGG pathways. A majority of these genes play an important role in negative regulation of endopeptidase activity. The GO and KEGG enrichment results of both the adult and no feeding comparison and the adult and suckling comparison showed that there were several items enriched in the up-regulated genes related to immunity in adult group samples. For example, immune system process (GO:0002376), MHC class II protein complex (GO:0042613) and Antigen processing and presentation (PATH:aml04612). Pancreatic immune cells are essential for pancreatic cell proliferation and development [14, 15]. High expression of immune-related genes in the adult pancreas is likely an important contributor to the function of the pancreas. Furthermore, terms associated with amino acid and protein metabolism displayed signatures of up-regulation in the adult group when compared to the suckling group (negative regulation of endopeptidase activity (GO:0010951), Alanine, aspartate and glutamate metabolism (PATH:aml00250)) (Table 3). Conversely, protein digestion and absorption (PATH:aml04974) and Cholesterol metabolism (PATH:aml04979) pathways were enriched in down-regulated genes in the adult group when compared to the no feeding or suckling groups. Additionally, up-regulated DEGs in the no feeding and suckling groups were enriched for both KEGG and GO terms related to cell cycle, cell proliferation, and nucleotide biosynthesis, in accordance with a neonatal proliferative state.

**Table 3:** Metabolism-related terms enriched by DEGs in giant panda pancreas at different developmental stages.

**Functional classes**	**Comparison**	**Terms**	**Adjust P**	**Gene number**
carbohydrate metabolism and energy production	Up-regulated in adult group when compared to no feeding group	electron transport chain [GO:0022900]	0.00256	15
carbohydrate metabolism and energy production	Up-regulated in adult group when compared to no feeding group	Oxidative phosphorylation [PATH:aml00190]	6.65E-08	30
amino acid and protein metabolism	Down-regulated in suckling group when compared to no feeding group	negative regulation of endopeptidase activity [GO:0010951]	2.16E-06	5
amino acid and protein metabolism	Down-regulated in suckling group when compared to no feeding group	cysteine-type endopeptidase inhibitor activity [GO:0004869]	0.00805	2
amino acid and protein metabolism	Down-regulated in suckling group when compared to no feeding group	serine-type endopeptidase inhibitor activity [GO:0004867]	0.0213	2
amino acid and protein metabolism	Up-regulated in suckling group when compared to no feeding group	serine-type peptidase activity [GO:0008236]	0.0137	2
amino acid and protein metabolism	Up-regulated in suckling group when compared to no feeding group	serine-type endopeptidase activity [GO:0004252]	0.0137	2
amino acid and protein metabolism	Up-regulated in suckling group when compared to no feeding group	peptidase activity [GO:0008233]	0.0201	2
amino acid and protein metabolism	Up-regulated in suckling group when compared to no feeding group	proteolysis [GO:0006508]	0.0295	2
amino acid and protein metabolism	Up-regulated in adult group when compared to no feeding group	Protein digestion and absorption [PATH:aml04974]	0.0165	16
amino acid and protein metabolism	Up-regulated in adult group when compared to suckling group	negative regulation of endopeptidase activity [GO:0010951]	0.0433	10
amino acid and protein metabolism	Up-regulated in adult group when compared to suckling group	Protein digestion and absorption [PATH:aml04974]	0.00633	12
amino acid and protein metabolism	Up-regulated in adult group when compared to suckling group	Alanine, aspartate and glutamate metabolism [PATH:aml00250]	0.0271	6
amino acid and protein metabolism	Down-regulated in adult group when compared to suckling group	Protein digestion and absorption [PATH:aml04974]	0.0240	12
amino acid and protein metabolism	Down-regulated in adult group when compared to no feeding group	Protein digestion and absorption [PATH:aml04974]	7.72E-09	29
lipid metabolism	Down-regulated in adult group when compared to no feeding group	Cholesterol metabolism [PATH:aml04979]	0.0301	11

### Identification of metabolism-related DEGs in the pancreas

**Figure 4:**
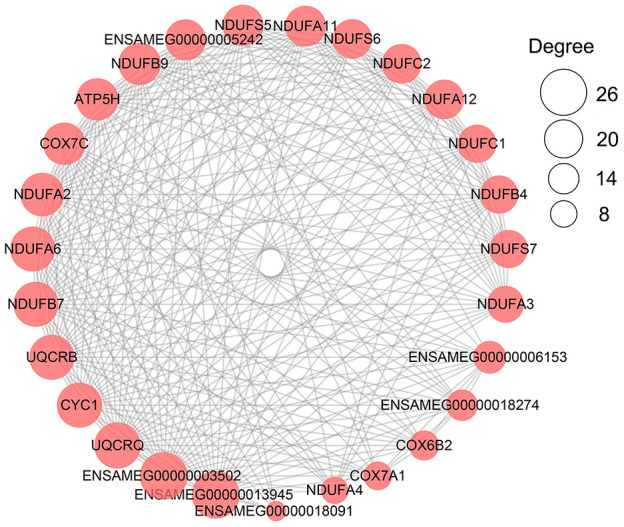
**The network of PPI analysis of metabolism-related DEGs in the pancreas.** The sub-network contained hub genes was extracted. The size of the circle represents the degree level of node gene. The bigger the circle, the more degree of node gene.

Similar to liver samples, the metabolism-related terms identified from the three development stages were grouped into carbohydrate metabolism and energy production, lipid metabolism, and amino acid and protein metabolism (Table 3). All genes involved in these metabolism-related terms were extracted (Supplementary Table 23 and Supplementary Figure 2). In protein-protein interaction analysis of these metabolism-related DEGs, *ENSAMEG00000013945* (cytochrome b-c1 complex subunit 6) and *ENSAMEG00000003502* (cytochrome b-c1 complex subunit 10) were hub genes. *UQCRQ*, *UQCRB*, *CYC1*, *NDUFA6*, and *NDUFB7* that are related to energy production also had high degrees next to *ENSAMEG00000013945* (cytochrome b-c1 complex subunit 6) and *ENSAMEG00000003502* (cytochrome b-c1 complex subunit 10) (Figure 4).

**Figure 5:**
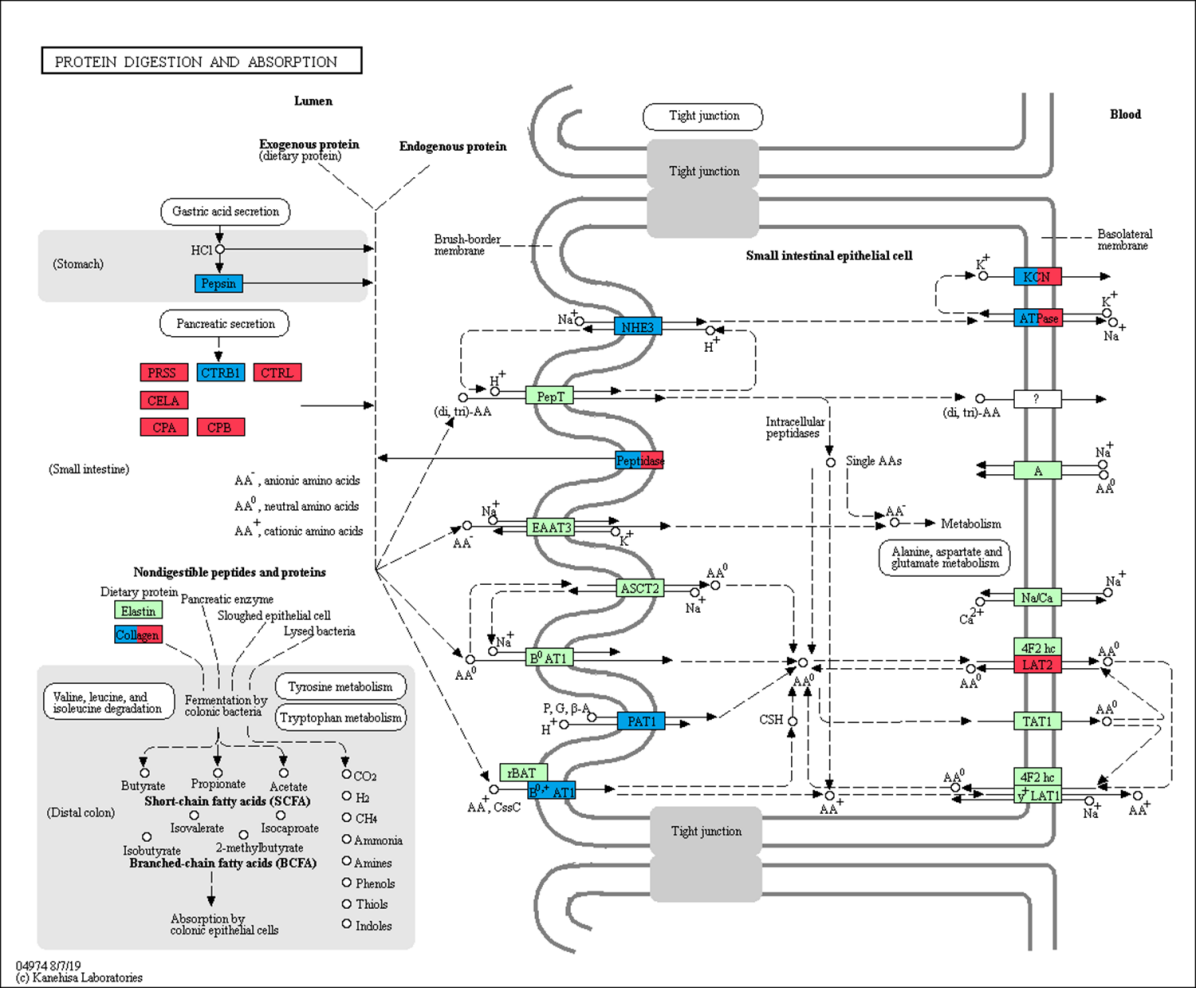
**Expression trend of DEGs involved in protein digestion and absorption pathway in the pancreas.** Red: genes that were continuously increased and had the highest expression in adult group during development; Blue: genes that were continuously decreased and had the lowest expression in adult group during development; Green: genes were not differentially expressed during development.

**Figure 6:**
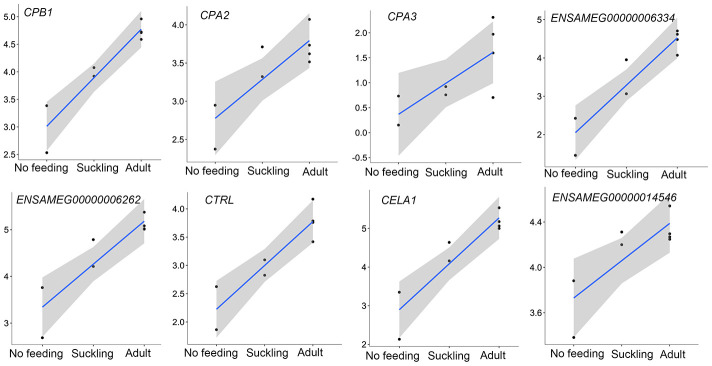
**The expression of pancreatic proteases-related genes that were continuously increased during postnatal development in the pancreas.** The vertical axis indicates the log10-transformed normalized CPM expression values. The gray shadow represents the 95% confidence field of the fitted curve.

**Figure 7:**
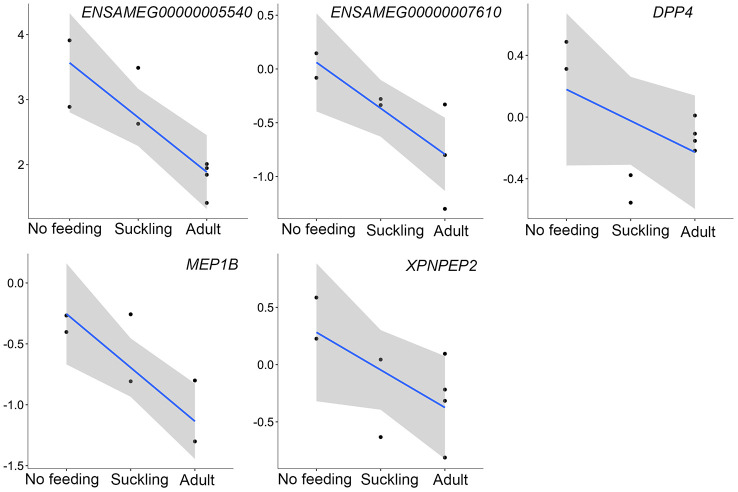
**The expression of proteases-related genes that were continuously decreased during postnatal development in the pancreas.** The vertical axis indicates the log10-transformed normalized CPM expression values. The gray shadow represents the 95% confidence field of the fitted curve.

**Figure 8:**
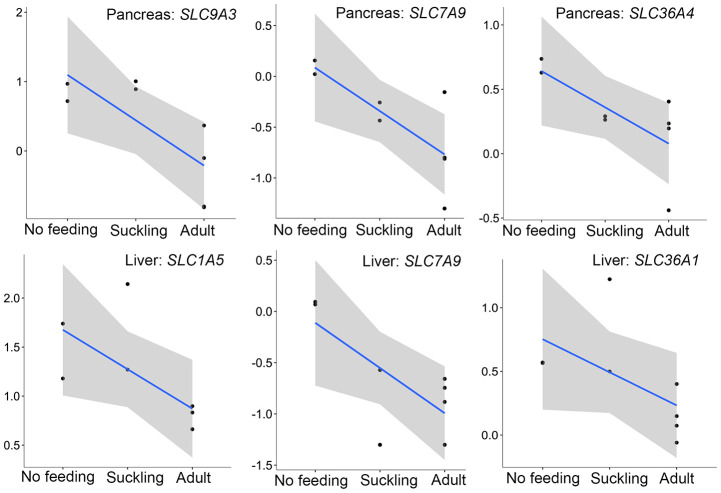
**The expression patterns of SLC family genes involved in amino acid transport in the pancreas and liver.** The vertical axis indicates the log10-transformed normalized CPM expression values. The gray shadow represents the 95% confidence field of the fitted curve.

In addition to the above, we identified genes that encoded major proteins in the electron transport chain (*PTGES2*, *NQO2*, *GLRX5*, *TXNRD2*, *FDX1*) and oxidative phosphorylation (e.g., *COX7A1*, *COX7C*, *NDUFA12*, *NDUFS7*, *ATP6V1F*, *ATP5PD*) (Supplementary Table 23). These were found up-regulated, and exhibiting the highest gene expression values, in the adult group when compared to the no feeding group. A series of genes associated with cholesterol metabolism represented the lowest expression values in the adult group (*ENSAMEG00000001014*, *LIPC*, *TSPO2*, *ANGPTL4*, *ABCA1*, *APOA1*, *ABCB11*, *SOAT2*, *APOC1*, *ENSAMEG00000013086*, *APOA2*). We also found 65 DEGs involved in amino acid and protein metabolism. Of the 65, 46 were related to protein digestion and absorption, and expression patterns of these genes are shown in Figure 5. Eight of 13 digesting enzyme coding genes increased in expression with developmental stage, and all eight had the highest expression in the adult group (*CPB1*, *CPA2*, *CPA3*, *ENSAMEG00000006334* anionic trypsin), *ENSAMEG00000006262* ((cationic trypsin), *CTRL*, *CELA1*, *ENSAMEG00000014546* (proproteinase E)) (Figure 6). The other five proteases coding genes (*ENSAMEG00000005540* (chymotrypsinogen B-like), *ENSAMEG00000007610* (pepsin A), *DPP4*, *MEP1B,* and *XPNPEP2*) displayed signatures of down-regulation in the adult group during development (Figure 7). The products from protein digestion are transported to small intestinal epithelial cells by different transport proteins located in the brush-border membrane. Three members of a solute carrier (SLC) family genes (*SLC9A3*, *SLC7A9*, *SLC36A4*) located in the brush-border membrane exhibited the lowest expression in the adult group (Figure 8).

## DISCUSSION

The liver and the pancreas are digestive organs that perform crucial roles in digestion and metabolism. Analyzing the gene expression in different postnatal hepatic and pancreatic developmental stages using RNA-seq could help us to compare expression levels of thousands of genes at one time [4, 16, 17]. Our study is the first to describe the expression profiles and dynamic changes in genes associated with nutrition metabolism in the giant panda liver and pancreas.

### Carbohydrate metabolism and energy production

The liver is the main site for carbohydrate metabolism. We found that genes related to carbohydrate metabolism were highly expressed in adult liver compared to samples from the suckling group (Table 2). After birth, animals need to adjust to a new situation and emerging new metabolic pathways [18]. For example, the suckling period corresponds to a lower glucose availability compared to adulthood, because less than 10% of the calories are available as glucose in the milk [19]. In this study, eight genes involved in starch and sucrose metabolism were all highly expressed in adult liver samples. Of these eight, *alpha-amylase 2B*, *MGAM,* and *SI* have key roles in digestion of dietary carbohydrates [20]. While, *GBA3*, *ENPP3*, *PYGM*, *GCK*, and *GBE1* encode enzymes that are involved in glycosides hydrolysis [21], extracellular nucleotides hydrolysis [22], glycogenolysis [23], glucose metabolism [24], and synthesis of glycogen [25], respectively. After weaning, the giant panda diet changes from low-carbohydrate and high-fat to a high-fiber diet [3]. Previous research reported that giant pandas can digest structural carbohydrates from bamboo without relying on microbial degradation [26]. However, the genetic mechanism of giant panda’s carbohydrate utilization from bamboo still remains unknown. High expression of carbohydrate metabolism-related genes in adult liver samples would help efficient sugar components digestion to fulfill their nutritional requirements from bamboo.

Oxidative phosphorylation, which is also known as electron transport-linked phosphorylation, is an effective way for cells to use enzymes to oxidize glucose and release energy [27]. In particular, the same two pathways, oxidative phosphorylation (aml00190) and electron transport chain (GO:0022900), which were enriched in up-regulated genes in adult liver samples were also found enriched in up-regulated genes in adult pancreas samples (Tables 2 and 3). Post-birth, the “incomplete” fetal liver organelles are transformed into mature and adult-type mitochondria by controlling the regulation of the stability and translational efficiency of the mRNAs encoding mitochondrial proteins [28]. It has been shown that mitochondrial mass increases during postnatal development, which is largely controlled at both transcriptional and posttranscriptional levels [29]. In this study, a series of mitochondrial genes were also found over-expression at the adult stage, such as *NDUFA12*, *COX6B2,* and *UQCRB*. Increased expression of these genes may be associated with an increase in the function of the adult liver and pancreas mitochondria. Similarly, down-regulation of genes related to oxidative phosphorylation during the early development stages suggested newborn giant pandas had a lower capacity of energy production [30].

### Lipid metabolism

The liver has a central role in the control of various aspects of lipid metabolism. Genes associated with the synthesis of lipids, long-chain fatty acid utilization, steroid hormone biosynthesis in the liver were all up-regulated in the adult group when compared to no feeding and suckling groups. A similar expression profile was also found during mouse liver development. Genes in pathways such as bile acid biosynthesis and fatty acid metabolism were found expressed mainly in the later stage of the mouse liver development [31, 32]. After birth, hepatoblasts gradually become mature hepatocytes with the liver function switching from hematopoiesis to metabolism [33, 34]. The high expression of lipid metabolism-related genes in adult giant panda samples may reflect the gradual improvement of liver metabolic function after birth. Interestingly, among lipid metabolism-related genes, particularly in members of cytochrome P-450 and UDP-glucuronosyltransferase were all up-regulated in the adult liver. Six of the ten CYP450 family genes belong to Cytochrome P450 family 2. *CYP2J2*, *ENSAMEG00000003107* (cytochrome P450 2C23-like), *CYP2E1*, *ENSAMEG00000006243* (cytochrome P450 2C41), *CYP2B6*, *ENSAMEG00000016104* (cytochrome P450 2C21) specifically involved in detoxification of xenobiotic compounds by mono-oxygenation or hydroxylation [35]. Likewise, *ENSAMEG00000005730* (UDP-glucuronosyltransferase 2B31), *ENSAMEG00000011718* (UDP-glucuronosyltransferase 2B31), *ENSAMEG00000011749* (UDP-glucuronosyltransferase 2C1) that belong to UDP-glucuronosyltransferase 2 family play an important role in hepatic detoxification [36]. Herbivores have acquired detoxification pathways to protect themselves against plant-derived toxicants [37]. As strict bamboo-diet species, high expression of cytochrome P-450 and UDP-glucuronosyltransferase families can help giant pandas to detoxify phytoalexins from bamboos.

The pancreas is an important exocrine organ and regulates lipid metabolism mainly by enzymes. In the pancreas, four lipase enzymes (*PNLIP*, *CEL*, *PNLIPRP2*, *PLA2G1B*) exhibited the highest expression in the adult group, although they were not enriched in any GO or KEGG terms. This is in accordance with previous research that found dietary triacylglycerol hydrolysis during adulthood was mainly accomplished by lipase secreted from the pancreas [38]. Neonatal cubs may not be able to digest TAGs efficiently because the function of the pancreas was not yet fully developed. Instead, lipase from milk was thought to help digestion of triglyceride during the neonatal period [39]. Conversely, genes in cholesterol metabolism-related terms had the lowest expression values in the adult group. *APOA1*, *APOA2*, *APOB*, and *APOC1* are the main components of CM particles [40], which are involved in the transport of dietary cholesterol and triglycerides to peripheral tissues [41]. Like bovine and human milk, giant panda milk is a primary source of cholesterol during the suckling period [42]. In humans, feeding neonates milk with high cholesterol has been found necessary for preventing the occurrence of hypercholesterolemia in later life [43]. Over-expression of cholesterol metabolism-related genes in the early stages of giant panda development would also be of special biological significance and needs further research.

### Amino acid and protein metabolism

In the liver, amino acid and protein metabolism related terms were only found in enrichment analysis of up-regulated genes in the adult group. For example, *AGXT* is the gene encoding the AGT protein, which can catalyze the conversion of glyoxylate to glycine [44]. *SHMT* encodes serine hydroxymethyltransferase, which correlates with the interconversion of serine and glycine [45]. This result was similar to genes involved in lipid metabolism, carbohydrate metabolism, and energy production, suggesting adult giant pandas have established and enhanced their nutrient metabolic ability compared to newborn pandas.

In the pancreas, except for the genes involved in the protein digestion and absorption pathway, genes associated with the metabolism of amino acids and protein were enriched in up-regulated genes between the adult and suckling comparison or the adult and no feeding comparison. Both up-regulated and down-regulated genes in the adult pancreas were observed in the protein digestion and absorption pathway (Table 3). The pancreas plays a significant role in digestion. The transcriptions of eight pancreatic proteases encoding genes, which had obvious roles in protein digestion in the intestine, increased with age and had the highest expression in the adult group (Figure 6). *CPB1*, *CPA2,* and *CPA3* produce three different carboxypeptidases that can hydrolyze the carboxy-terminal peptide bond of a protein or peptide [46, 47]. *ENSAMEG00000006334* and *ENSAMEG00000006262* are trypsin encoding genes, and tryptic digestion is an essential step in protein digestion [48]. *CTRL* encodes a serine-type endopeptidase with chymotrypsin- and elastase-2-like activities [49]. Chymotrypsin like elastase 1, also known as elastase-1 (ELA1), is encoded by *CELA1*. Chymotrypsin like elastase 1 only selects Ala-Gly and Ala-Ala bonds to be hydrolyzed and is complementary to chymotrypsin and trypsin [50]. After birth, the increase in the expression of those pancreatic proteases suggested the gradual improvement of pancreatic function in protein digestion. This was also supported by research of Efird who observed that weaning pigs had greater proteolytic activity and an apparent increase in pancreatic secretion of trypsin and chymotrypsin than suckling pigs [51]. Similarly, enzyme activity and gene expression level of chymotrypsin, trypsin, and amylase were increased in weaning calves [5].

However, the mRNA level of five additional proteases coding genes peaked at birth before decreasing during the postnatal development phase (Figure 7). *ENSAMEG00000005540* encodes chymotrypsinogen B-like, a preproprotein that is synthesized in the pancreas and secretes into the small intestine to generate a functional enzyme. Pepsin A (*ENSAMEG00000007610)* is expressed through the digestive tract, especially in the stomach. It is most efficient in hydrolyzing peptide bonds involving phenylalanine, tryptophan, and tyrosine [52]. DPP4, MEP1B, and XPNPEP2 are mainly produced by enterocytes of the small intestine. These enzymes digest small peptides produced by hydrolysis of proteins by gastric and pancreatic enzymes [49].

Interestingly, we found three pancreatic genes (*SLC9A3*, *SLC7A9*, *SLC36A4*) that had the lowest expression in the adult group were directly related to protein absorption (Figure 8). *SLC9A3*, *SLC36A4,* and *SLC7A9* are members of a SLC family and play important roles in intestinal absorption of amino acids [53]. SLC9A3 (NHE3) Na+/H+ exchanger converts the Na+ gradient to an inwardly directed H+ gradient, enabling the uptake of H+-coupled amino acid. SLC7A9 functions as a high-affinity and sodium-independent transporter for cystine and neutral and dibasic amino acids. SLC36A4 plays a role in the sodium-independent electroneutral transport of tryptophan, proline, and alanine. We also analyzed the expression profiles of SLC family genes involved in amino acid transport in the liver. Only four SLC family genes (*SLC1A5*, *SLC36A1*, *SLC7A9*, *SLC16A10*) were significantly expressed during liver development. Similar to the pancreas, *SLC1A5*, *SLC36A1*, *SLC7A9* that play important roles in the transportation of AA^0^ and AA^+^ into small intestinal epithelial cells were expressed least in adult liver samples (Figure 8).

Protein is an important component of infant nutrition, as it is required for infant growth and development [54]. In humans, the highest rate of protein deposition that occurs within a lifetime sustains the rapid growth during the neonatal period (0.27 g/kg/day for the first 2 months of life and 0.07 g/kg/day for 16- to 18-year-old individuals) [55]. While inefficient utilization of milk protein can cause a low rate of growth of the suckling piglet [56]. In this study, the expression of dietary protein digestion and absorption related genes peak after birth and decline during the postnatal period. The high expression of those genes during the neonatal period might be vital for adequate protein absorption from milk and energy utilization to guarantee the fast growth rate of giant pandas after birth [57].

In summary, our genomic expression profiling provided a global assessment of changes in gene expression patterns associated with nutrition metabolism. PCA and clustering analyses showed that the suckling group and newborn (no feeding) group were clustered together and separated from the adult group, which may indicate the similarity of metabolic function between milk feeding individuals and newborns in this study. Over-expression of cholesterol metabolism, protein digestion and absorption related genes in early stages of giant panda development may meet the nutritional requirement of highly altricial neonates. Up-regulation of other genes related to carbohydrate metabolism and energy production, amino acid and protein metabolism, and lipid metabolism in adult liver and pancreas groups suggested that the metabolism was higher in mature giant pandas. This observation indicated the molecular mechanism by which giant pandas gradually build and strengthen the digestive system at the RNA level to maintain a healthy state.

## MATERIALS AND METHODS

### Sample collection

Giant panda tissue samples were provided by Chengdu Research Base of Giant Panda Breeding in Chengdu and China Research and Conservation Center for the Giant Panda at Wolong, Sichuan Province, China (Table 1). Eight liver and eight pancreas tissue samples were obtained from nine giant pandas that had died at three life stages. Samples NH and YR had died shortly after birth and had not been fed (no feeding group). AB and LT had died during the suckling period and were suckled for four and six days, respectively (suckling group). NH, YR, AB, and LT had all died by asphyxiation in accidents. The remaining samples were sourced from five wild adults with a total bamboo diet (adult group). The wild adults were seriously injured when found during ecological investigations and died during the rescue attempt. None of liver and pancreas tissues sampled exhibited pathological changes. Fresh excised liver and pancreas tissue samples were immediately stored at -80°C. All the animal work was conducted according to the guidelines approved by the Ethics Committee, College of Life Sciences, Sichuan University (Grant No: 20190506001).

### Isolation of total RNA

Each organ was dissected and homogenized before RNA extraction. The giant panda liver and pancreas total RNA were extracted using TRIzol reagent (Invitrogen, Carlsbad, CA, USA) according to the manufacturer’s protocol, including treatment with DNase. RNA integrity numbers (RINs) of samples greater than 5.8 were available for RNA-seq libraries construction.

### Library preparation and sequencing

Sequencing libraries were prepared using NEBNext® UltraTM RNA Library Prep Kit for Illumina® (NEB, USA) following manufacturer’s instructions. Index codes were added to attribute sequences to each sample. After column purification, the quality of the resulting libraries was assessed on the Agilent Bioanalyzer 2100 system. The library preparations were sequenced on the Illumina HiSeq2000 platform. All reads have been submitted to NCBI Sequence Read Archive with SRA numbers SRR11301087- SRR11301092, SRR11301095- SRR11301096, SRR11445247- SRR11445254.

### RNA-seq read mapping

Giant panda reference genome and reference annotation were obtained from Ensembl, release 98. Low-quality reads and any adapter sequences were removed using NGS QC Toolkit [58] with a quality score of 20. We mapped the high-quality reads that passed filter thresholds to the giant panda genome using HISAT2 [59]. We then used SAMtools to convert the alignments in SAM format to BAM format [60]. After reading in the reference annotations to count fragments, a count of all of exons grouped by gene was calculated by featureCounts [61].

### Gene expression estimation and differentially expressed genes analysis

Raw gene counts were normalized using TMM algorithm as implemented in the R package edgeR [62]. Normalized gene expression matrix of all samples was log2 transformed. The PCA was performed on these transformed data using the ‘prcomp’ function in the R package ‘stats’. We calculated the Spearman correlation of each sample using the function “cor” in R, and the function ‘heatmap.2’ in package ‘gplots’ was used to plot the results.

Low expressed genes were filtered to include only genes expressed at least greater than 0 count-per-million (CPM) in the samples of a given stage. Then, the normalized CPM values were used to analyze the gene expression changes in the postnatal giant panda liver and pancreas samples, comparing no feeding with suckling, and suckling with adult. Significant differentially expressed genes were taken as those with an absolute value of log2 fold change > 1 and Benjamini-Hochberg false discovery rate < 0.05.

### Gene enrichment analysis

GO term and KEGG pathway enrichment analyses were performed by using ‘enricher’ function in the ‘clusterProfiler’ package in R [63]. GO term of each gene was downloaded via the highly customizable BioMart data mining tool. KEGG pathway of each gene was downloaded from KEGG PATHWAY database (https://www.genome.jp/kegg/pathway.html). The corrected P values of Benjamini-Hochberg multiple testing less than 0.05 were considered to be significantly enriched.

### Protein-protein interaction network analysis

Protein-protein interaction (PPI) network analysis of metabolism-related DEGs was performed by using STRING web server (https://string-db.org) version 11.0. The network plot was drawn by Cytoscape 3.7.1 [64] and the gene with the highest number of degrees was regarded as a hub gene.

## Supplementary Material

Supplementary Figures

Supplementary Table 1

Supplementary Table 2

Supplementary Table 3

Supplementary Table 4

Supplementary Table 5

Supplementary Table 6

Supplementary Table 7

Supplementary Table 8

Supplementary Table 9

Supplementary Table 10

Supplementary Tables 11, 12, 13 and 14

Supplementary Table 15

Supplementary Table 16

Supplementary Table 17

Supplementary Table 18

Supplementary Table 19

Supplementary Table 20

Supplementary Table 21

Supplementary Table 22

Supplementary Table 23
