# Supplementary material for: Gene expression profiles during postnatal development of the liver and pancreas in giant pandas
Source: Aging (Albany NY). 2020 Aug 15;12(15):15705–29. doi: 10.18632/aging.103783 (PMC7467380; doi:10.18632/aging.103783)
Supplement: Supplementary Figures [file aging-12-103783-s021..pdf]

SUPPLEMENTARY FIGURES

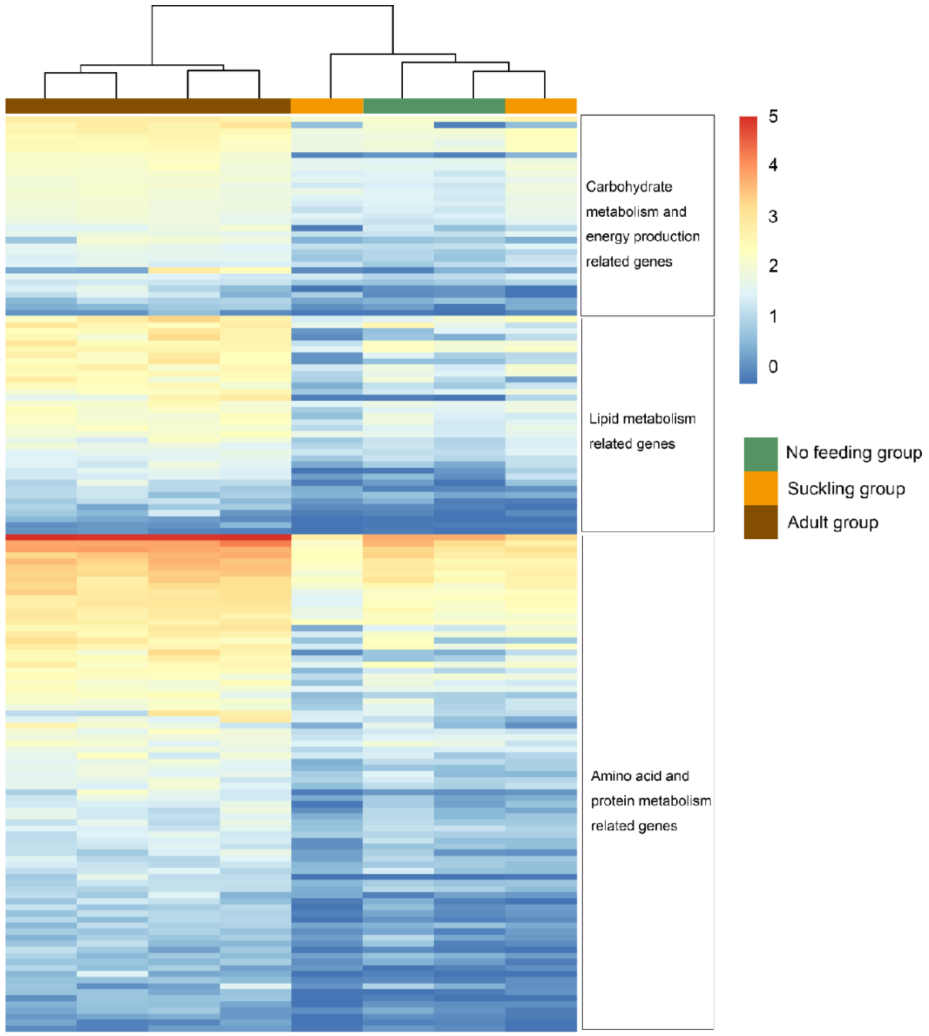

Supplementary Figure 1 Heat map plot of 146 metabolism-related DEGs in the liver. Each column represents a specimen and each row represents a gene. Clustering is performed in column. Color indicates the log10-transformed normalized CPM expression values.

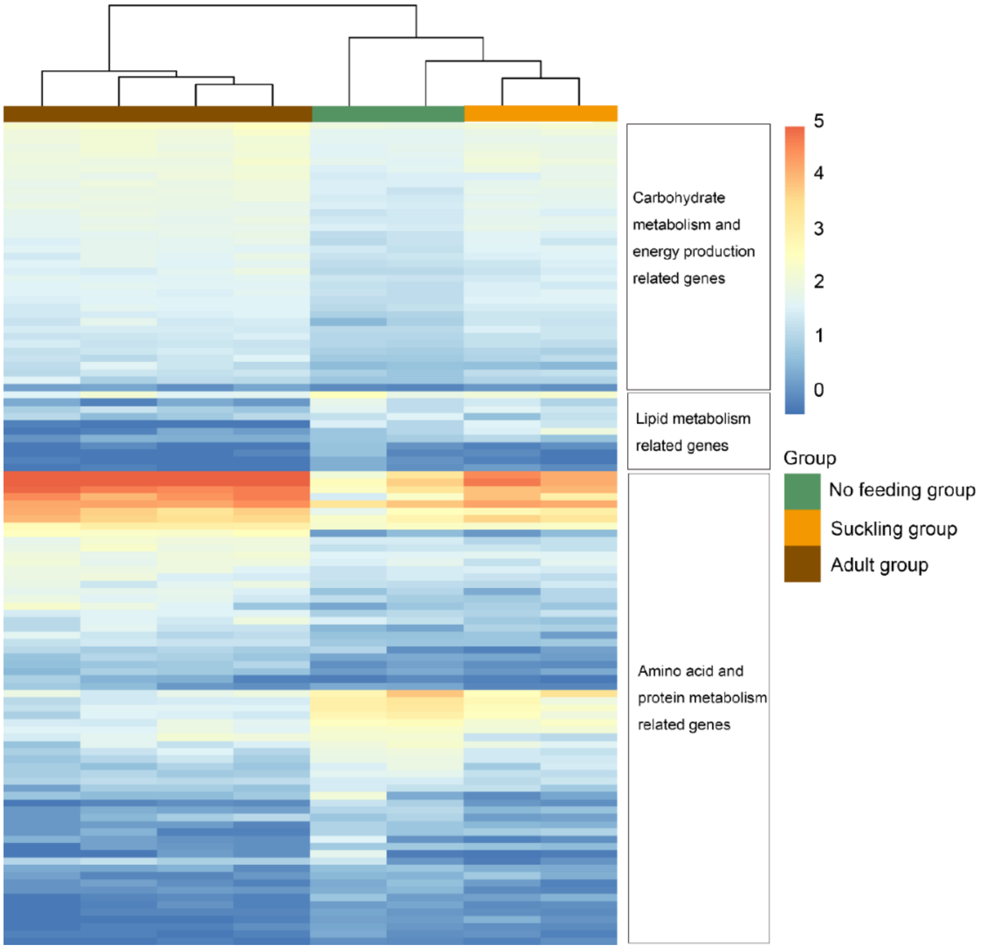

Supplementary Figure 2. Heat map plot of 113 metabolism-related DEGs in the pancreas. Each column represents a specimen and each row represents a gene. Clustering is performed in column. Color indicates the log10-transformed normalized CPM expression values.
