# Supplementary material for: Gene expression profiles during postnatal development of the liver and pancreas in giant pandas
Source: Aging (Albany NY). 2020 Aug 15;12(15):15705–29. doi: 10.18632/aging.103783 (PMC7467380; doi:10.18632/aging.103783)
Supplement: Supplementary Table 1 [file aging-12-103783-s020..pdf]

SUPPLEMENTARY TABLES

Supplementary Table 1. The top 10 of the most highly expressed genes in the three postnatal developmental stages of liver.

Group	Ensemble ID	Symbol	NH	YR		
No feeding group	ENSAMEG00000023436	COX1	9756.00	147337.42		
	ENSAMEG00000004932	ALB	61178.21	76816.95		
	ENSAMEG00000023448	ND4	3683.22	81150.51		
	ENSAMEG00000006318	None	28117.38	55300.09		
	ENSAMEG00000006871	LOC100472013	5084.93	67570.66		
	ENSAMEG00000023443	COX3	3508.02	64004.35		
	ENSAMEG00000023455	CYTB	4054.62	58126.088		
	ENSAMEG00000023442	ATP6	3188.95	58681.40		
	ENSAMEG00000023439	COX2	2988.19	55884.78		
	ENSAMEG00000023430	ND2	2216.17	42200.65		
**Group**	**Ensemble ID**	**Symbol**	**AB**	**LT**		
Suckling group	ENSAMEG00000004932	ALB	136531.04	19953.67		
	ENSAMEG00000023436	COX1	27751.54	111204.65		
	ENSAMEG00000006871	LOC100472013	23118.74	91085.25		
	ENSAMEG00000006318	None	56316.83	19755.60		
	ENSAMEG00000023448	ND4	13905.71	47491.15		
	ENSAMEG00000023442	ATP6	14956.36	41074.12		
	ENSAMEG00000023455	CYTB	11770.93	40879.74		
	ENSAMEG00000023443	COX3	12716.66	38222.35		
	ENSAMEG00000023439	COX2	13989.96	36363.50		
	ENSAMEG00000023430	ND2	10971.47	28860.23		
**Group**	**Ensemble ID**	**Symbol**	**YS**	**HT**	**CC**	**PP**
Adult group	ENSAMEG00000004932	ALB	167548.34	76797.42	109881.46	245062.94
	ENSAMEG00000002287	LOC100482523	88143.42	97980.01	86923.31	107729.87
	ENSAMEG00000006318	None	27056.67	20188.85	16740.06	38674.56
	ENSAMEG00000023442	ATP6	38376.79	21968.23	18248.36	12448.58
	ENSAMEG00000008662	APOA1	27843.45	14730.88	12507.82	34044.19
	ENSAMEG00000016713	None	21586.80	16322.76	20602.57	10035.15
	ENSAMEG00000018459	FGB	11069.02	9978.05	21084.40	21685.78
	ENSAMEG00000018463	FGG	9891.64	10553.21	22160.24	19838.73
	ENSAMEG00000023448	ND4	26147.82	11195.78	6908.49	13117.63
	ENSAMEG00000012556	LOC100474871	28393.64	1724.16	17656.30	8186.53
