# Supplementary material for: Gene expression profiles during postnatal development of the liver and pancreas in giant pandas
Source: Aging (Albany NY). 2020 Aug 15;12(15):15705–29. doi: 10.18632/aging.103783 (PMC7467380; doi:10.18632/aging.103783)
Supplement: Supplementary Table 2 [file aging-12-103783-s013..docx]

**Supplementary Table 2. Significantly enriched GO categories for up-regulated DEGs in liver adult group compared with liver suckling group.**

| **ID** | **Description** | **pvalue** | **p.adjust** | **qvalue** | **geneID** | **Count** |
| --- | --- | --- | --- | --- | --- | --- |
| GO:0009986 | cell surface | 8.17E-11 | 6.75E-08 | 6.02E-08 | ENSAMEG00000000141/ENSAMEG00000015921/ENSAMEG00000003004/ENSAMEG00000018351/ENSAMEG00000004654/ENSAMEG00000002390/ENSAMEG00000017869/ENSAMEG00000014110/ENSAMEG00000000862/ENSAMEG00000018451/ENSAMEG00000016910/ENSAMEG00000001013/ENSAMEG00000007760/ENSAMEG00000017910/ENSAMEG00000013098/ENSAMEG00000011169/ENSAMEG00000019083/ENSAMEG00000012707/ENSAMEG00000008895/ENSAMEG00000016574/ENSAMEG00000005307/ENSAMEG00000012078/ENSAMEG00000011357/ENSAMEG00000005401/ENSAMEG00000004883/ENSAMEG00000014874/ENSAMEG00000004244/ENSAMEG00000018463/ENSAMEG00000004240/ENSAMEG00000009345/ENSAMEG00000001184/ENSAMEG00000011737/ENSAMEG00000016144/ENSAMEG00000007834/ENSAMEG00000012157/ENSAMEG00000011706/ENSAMEG00000018459/ENSAMEG00000009619/ENSAMEG00000017756/ENSAMEG00000009222/ENSAMEG00000001811/ENSAMEG00000014069 | 42 |
| GO:0004497 | monooxygenase activity | 1.35E-10 | 6.75E-08 | 6.02E-08 | ENSAMEG00000011960/ENSAMEG00000005138/ENSAMEG00000001708/ENSAMEG00000004376/ENSAMEG00000006243/ENSAMEG00000008842/ENSAMEG00000003132/ENSAMEG00000002714/ENSAMEG00000013578/ENSAMEG00000016104/ENSAMEG00000016920/ENSAMEG00000011824/ENSAMEG00000009440/ENSAMEG00000006404/ENSAMEG00000002316/ENSAMEG00000002179/ENSAMEG00000017038/ENSAMEG00000017636 | 18 |
| GO:0005506 | iron ion binding | 1.40E-10 | 6.75E-08 | 6.02E-08 | ENSAMEG00000011960/ENSAMEG00000005138/ENSAMEG00000001708/ENSAMEG00000004376/ENSAMEG00000006243/ENSAMEG00000005288/ENSAMEG00000008842/ENSAMEG00000003132/ENSAMEG00000020100/ENSAMEG00000002714/ENSAMEG00000013578/ENSAMEG00000016104/ENSAMEG00000011824/ENSAMEG00000001527/ENSAMEG00000009440/ENSAMEG00000006404/ENSAMEG00000004309/ENSAMEG00000002316/ENSAMEG00000017058/ENSAMEG00000000726/ENSAMEG00000005445/ENSAMEG00000002179/ENSAMEG00000017636/ENSAMEG00000008222 | 24 |
| GO:0020037 | heme binding | 4.29E-10 | 1.56E-07 | 1.39E-07 | ENSAMEG00000011960/ENSAMEG00000005138/ENSAMEG00000011825/ENSAMEG00000001708/ENSAMEG00000004376/ENSAMEG00000006243/ENSAMEG00000015468/ENSAMEG00000005288/ENSAMEG00000008842/ENSAMEG00000003132/ENSAMEG00000001013/ENSAMEG00000015532/ENSAMEG00000002714/ENSAMEG00000013578/ENSAMEG00000016104/ENSAMEG00000011824/ENSAMEG00000009440/ENSAMEG00000002316/ENSAMEG00000015408/ENSAMEG00000017636/ENSAMEG00000005154/ENSAMEG00000006039 | 22 |
| GO:0062023 | collagen-containing extracellular matrix | 3.25E-09 | 9.42E-07 | 8.40E-07 | ENSAMEG00000000687/ENSAMEG00000009601/ENSAMEG00000014674/ENSAMEG00000003004/ENSAMEG00000003222/ENSAMEG00000003695/ENSAMEG00000017473/ENSAMEG00000011201/ENSAMEG00000001256/ENSAMEG00000013516/ENSAMEG00000009345/ENSAMEG00000002331/ENSAMEG00000012327/ENSAMEG00000000126/ENSAMEG00000001432/ENSAMEG00000002621/ENSAMEG00000004454 | 17 |
| GO:0016705 | oxidoreductase activity, acting on paired donors, with incorporation or reduction of molecular oxygen | 1.20E-08 | 2.90E-06 | 2.59E-06 | ENSAMEG00000011960/ENSAMEG00000005138/ENSAMEG00000001708/ENSAMEG00000004376/ENSAMEG00000006243/ENSAMEG00000005288/ENSAMEG00000008842/ENSAMEG00000003132/ENSAMEG00000002714/ENSAMEG00000013578/ENSAMEG00000016104/ENSAMEG00000011824/ENSAMEG00000009440/ENSAMEG00000002316/ENSAMEG00000000726/ENSAMEG00000017636 | 16 |
| GO:0016712 | oxidoreductase activity, acting on paired donors, with incorporation or reduction of molecular oxygen, reduced flavin or flavoprotein as one donor, and incorporation of one atom of oxygen | 3.26E-08 | 6.76E-06 | 6.03E-06 | ENSAMEG00000011960/ENSAMEG00000004376/ENSAMEG00000005288/ENSAMEG00000008842/ENSAMEG00000003132/ENSAMEG00000011824/ENSAMEG00000009440/ENSAMEG00000002316/ENSAMEG00000017636 | 9 |
| GO:0016491 | oxidoreductase activity | 2.18E-07 | 3.95E-05 | 3.52E-05 | ENSAMEG00000009705/ENSAMEG00000011960/ENSAMEG00000005138/ENSAMEG00000014033/ENSAMEG00000009706/ENSAMEG00000018686/ENSAMEG00000017684/ENSAMEG00000004376/ENSAMEG00000006243/ENSAMEG00000015468/ENSAMEG00000008842/ENSAMEG00000018748/ENSAMEG00000003132/ENSAMEG00000017691/ENSAMEG00000020100/ENSAMEG00000014144/ENSAMEG00000002714/ENSAMEG00000016104/ENSAMEG00000005535/ENSAMEG00000011824/ENSAMEG00000017964/ENSAMEG00000018008/ENSAMEG00000009440/ENSAMEG00000018713/ENSAMEG00000017058/ENSAMEG00000005739/ENSAMEG00000006562/ENSAMEG00000000726/ENSAMEG00000005445/ENSAMEG00000005398/ENSAMEG00000010966/ENSAMEG00000017297/ENSAMEG00000017038/ENSAMEG00000017636/ENSAMEG00000005154/ENSAMEG00000012577/ENSAMEG00000016662/ENSAMEG00000011835/ENSAMEG00000009730/ENSAMEG00000008222 | 40 |
| GO:0042613 | MHC class II protein complex | 3.86E-07 | 6.22E-05 | 5.54E-05 | ENSAMEG00000004654/ENSAMEG00000002390/ENSAMEG00000002352/ENSAMEG00000001952/ENSAMEG00000002099/ENSAMEG00000002361/ENSAMEG00000002342 | 7 |
| GO:0010951 | negative regulation of endopeptidase activity | 6.47E-07 | 9.39E-05 | 8.38E-05 | ENSAMEG00000008108/ENSAMEG00000017742/ENSAMEG00000016366/ENSAMEG00000016040/ENSAMEG00000014526/ENSAMEG00000004015/ENSAMEG00000001013/ENSAMEG00000017421/ENSAMEG00000012033/ENSAMEG00000013869/ENSAMEG00000004046/ENSAMEG00000011447/ENSAMEG00000001045/ENSAMEG00000014114/ENSAMEG00000002621/ENSAMEG00000010076 | 16 |
| GO:0009897 | external side of plasma membrane | 7.49E-07 | 9.88E-05 | 8.81E-05 | ENSAMEG00000010324/ENSAMEG00000018351/ENSAMEG00000004654/ENSAMEG00000012894/ENSAMEG00000016542/ENSAMEG00000013166/ENSAMEG00000007760/ENSAMEG00000007133/ENSAMEG00000012707/ENSAMEG00000014850/ENSAMEG00000014063/ENSAMEG00000018463/ENSAMEG00000017297/ENSAMEG00000007985/ENSAMEG00000012157/ENSAMEG00000018459/ENSAMEG00000001432/ENSAMEG00000003697/ENSAMEG00000014069 | 19 |
| GO:0042101 | T cell receptor complex | 1.19E-06 | 1.35E-04 | 1.20E-04 | ENSAMEG00000004919/ENSAMEG00000007493/ENSAMEG00000013166/ENSAMEG00000013155/ENSAMEG00000000022/ENSAMEG00000010191 | 6 |
| GO:0002250 | adaptive immune response | 1.21E-06 | 1.35E-04 | 1.20E-04 | ENSAMEG00000005863/ENSAMEG00000002390/ENSAMEG00000002352/ENSAMEG00000001952/ENSAMEG00000002099/ENSAMEG00000004883/ENSAMEG00000002361/ENSAMEG00000002342/ENSAMEG00000007109 | 9 |
| GO:0007155 | cell adhesion | 1.97E-06 | 2.05E-04 | 1.82E-04 | ENSAMEG00000011252/ENSAMEG00000009150/ENSAMEG00000003156/ENSAMEG00000015855/ENSAMEG00000000862/ENSAMEG00000017867/ENSAMEG00000013890/ENSAMEG00000014604/ENSAMEG00000010408/ENSAMEG00000007760/ENSAMEG00000016286/ENSAMEG00000017585/ENSAMEG00000008607/ENSAMEG00000002289/ENSAMEG00000016294/ENSAMEG00000010667/ENSAMEG00000010373/ENSAMEG00000014874/ENSAMEG00000016517/ENSAMEG00000010706/ENSAMEG00000010729/ENSAMEG00000011794/ENSAMEG00000017636/ENSAMEG00000007109/ENSAMEG00000000126/ENSAMEG00000012157/ENSAMEG00000000278/ENSAMEG00000009619 | 28 |
| GO:0010596 | negative regulation of endothelial cell migration | 2.29E-06 | 2.22E-04 | 1.98E-04 | ENSAMEG00000017643/ENSAMEG00000014110/ENSAMEG00000016821/ENSAMEG00000006196/ENSAMEG00000004377/ENSAMEG00000002851/ENSAMEG00000001811 | 7 |
| GO:0002504 | antigen processing and presentation of peptide or polysaccharide antigen via MHC class II | 4.84E-06 | 4.39E-04 | 3.92E-04 | ENSAMEG00000002390/ENSAMEG00000002352/ENSAMEG00000001952/ENSAMEG00000002099/ENSAMEG00000002361/ENSAMEG00000002342 | 6 |
| GO:0006955 | immune response | 8.86E-06 | 7.57E-04 | 6.75E-04 | ENSAMEG00000010324/ENSAMEG00000015230/ENSAMEG00000015352/ENSAMEG00000004654/ENSAMEG00000002390/ENSAMEG00000002352/ENSAMEG00000014902/ENSAMEG00000001952/ENSAMEG00000017754/ENSAMEG00000002099/ENSAMEG00000017094/ENSAMEG00000016736/ENSAMEG00000014444/ENSAMEG00000015402/ENSAMEG00000013850/ENSAMEG00000005780/ENSAMEG00000014335/ENSAMEG00000002361/ENSAMEG00000002342/ENSAMEG00000000999/ENSAMEG00000004830 | 21 |
| GO:0005887 | integral component of plasma membrane | 1.19E-05 | 9.56E-04 | 8.53E-04 | ENSAMEG00000003963/ENSAMEG00000008476/ENSAMEG00000018351/ENSAMEG00000011052/ENSAMEG00000014072/ENSAMEG00000007248/ENSAMEG00000016542/ENSAMEG00000014152/ENSAMEG00000009861/ENSAMEG00000000245/ENSAMEG00000009070/ENSAMEG00000018451/ENSAMEG00000015783/ENSAMEG00000000130/ENSAMEG00000019083/ENSAMEG00000000974/ENSAMEG00000010947/ENSAMEG00000006846/ENSAMEG00000016574/ENSAMEG00000001347/ENSAMEG00000002295/ENSAMEG00000001124/ENSAMEG00000004240/ENSAMEG00000001184/ENSAMEG00000014354/ENSAMEG00000016787/ENSAMEG00000011849/ENSAMEG00000000189/ENSAMEG00000001343/ENSAMEG00000007834/ENSAMEG00000006264/ENSAMEG00000007109/ENSAMEG00000004900/ENSAMEG00000016101/ENSAMEG00000012249/ENSAMEG00000010085/ENSAMEG00000017756/ENSAMEG00000018567/ENSAMEG00000001811 | 39 |
| GO:0005102 | signaling receptor binding | 1.86E-05 | 1.35E-03 | 1.20E-03 | ENSAMEG00000000141/ENSAMEG00000008621/ENSAMEG00000009150/ENSAMEG00000003490/ENSAMEG00000004441/ENSAMEG00000004961/ENSAMEG00000013890/ENSAMEG00000001013/ENSAMEG00000017263/ENSAMEG00000007102/ENSAMEG00000005307/ENSAMEG00000012078/ENSAMEG00000005401/ENSAMEG00000013869/ENSAMEG00000001256/ENSAMEG00000018463/ENSAMEG00000004240/ENSAMEG00000001343/ENSAMEG00000018459/ENSAMEG00000002621/ENSAMEG00000012348 | 21 |
| GO:0045766 | positive regulation of angiogenesis | 1.86E-05 | 1.35E-03 | 1.20E-03 | ENSAMEG00000017643/ENSAMEG00000010197/ENSAMEG00000006561/ENSAMEG00000014090/ENSAMEG00000013578/ENSAMEG00000013593/ENSAMEG00000009345/ENSAMEG00000000640/ENSAMEG00000017636/ENSAMEG00000019454/ENSAMEG00000013675/ENSAMEG00000004830/ENSAMEG00000002621/ENSAMEG00000001811 | 14 |
| GO:0004866 | endopeptidase inhibitor activity | 3.61E-05 | 2.38E-03 | 2.13E-03 | ENSAMEG00000004015/ENSAMEG00000012033/ENSAMEG00000013869/ENSAMEG00000004046/ENSAMEG00000011447/ENSAMEG00000001045 | 6 |
| GO:0044344 | cellular response to fibroblast growth factor stimulus | 3.61E-05 | 2.38E-03 | 2.13E-03 | ENSAMEG00000008499/ENSAMEG00000014902/ENSAMEG00000016821/ENSAMEG00000005780/ENSAMEG00000011706/ENSAMEG00000004830 | 6 |
| GO:0005178 | integrin binding | 4.98E-05 | 3.14E-03 | 2.80E-03 | ENSAMEG00000003817/ENSAMEG00000019897/ENSAMEG00000003156/ENSAMEG00000012894/ENSAMEG00000014604/ENSAMEG00000007760/ENSAMEG00000010197/ENSAMEG00000013850/ENSAMEG00000011357/ENSAMEG00000014874/ENSAMEG00000018076/ENSAMEG00000007109 | 12 |
| GO:0034116 | positive regulation of heterotypic cell-cell adhesion | 9.34E-05 | 5.65E-03 | 5.03E-03 | ENSAMEG00000004919/ENSAMEG00000007760/ENSAMEG00000007672/ENSAMEG00000018463/ENSAMEG00000018459 | 5 |
| GO:0007219 | Notch signaling pathway | 1.11E-04 | 6.44E-03 | 5.74E-03 | ENSAMEG00000019897/ENSAMEG00000015855/ENSAMEG00000009070/ENSAMEG00000016821/ENSAMEG00000002945/ENSAMEG00000014090/ENSAMEG00000016144/ENSAMEG00000016700/ENSAMEG00000005526/ENSAMEG00000010817 | 10 |
| GO:0001525 | angiogenesis | 1.15E-04 | 6.44E-03 | 5.74E-03 | ENSAMEG00000017643/ENSAMEG00000016821/ENSAMEG00000007760/ENSAMEG00000010197/ENSAMEG00000014090/ENSAMEG00000014850/ENSAMEG00000012768/ENSAMEG00000015317/ENSAMEG00000009219/ENSAMEG00000001343/ENSAMEG00000017636/ENSAMEG00000007109/ENSAMEG00000002621/ENSAMEG00000001811 | 14 |
| GO:0005604 | basement membrane | 1.61E-04 | 8.67E-03 | 7.73E-03 | ENSAMEG00000003817/ENSAMEG00000016366/ENSAMEG00000013890/ENSAMEG00000013964/ENSAMEG00000002289/ENSAMEG00000017272/ENSAMEG00000000726/ENSAMEG00000004084/ENSAMEG00000009997 | 9 |
| GO:0019882 | antigen processing and presentation | 1.91E-04 | 9.89E-03 | 8.82E-03 | ENSAMEG00000004654/ENSAMEG00000002390/ENSAMEG00000002352/ENSAMEG00000001952/ENSAMEG00000002099/ENSAMEG00000002361/ENSAMEG00000002342 | 7 |
| GO:0030247 | polysaccharide binding | 2.20E-04 | 1.09E-02 | 9.70E-03 | ENSAMEG00000010324/ENSAMEG00000008566/ENSAMEG00000002390/ENSAMEG00000017754/ENSAMEG00000015402 | 5 |
| GO:0043433 | negative regulation of DNA-binding transcription factor activity | 2.25E-04 | 1.09E-02 | 9.70E-03 | ENSAMEG00000010926/ENSAMEG00000004441/ENSAMEG00000015410/ENSAMEG00000018773/ENSAMEG00000000089/ENSAMEG00000009673/ENSAMEG00000008322/ENSAMEG00000016060/ENSAMEG00000010817 | 9 |
| GO:0008201 | heparin binding | 2.58E-04 | 1.21E-02 | 1.08E-02 | ENSAMEG00000014033/ENSAMEG00000003004/ENSAMEG00000012158/ENSAMEG00000001013/ENSAMEG00000010197/ENSAMEG00000006196/ENSAMEG00000018298/ENSAMEG00000017272/ENSAMEG00000015317/ENSAMEG00000002331/ENSAMEG00000018076 | 11 |
| GO:0031012 | extracellular matrix | 2.82E-04 | 1.27E-02 | 1.13E-02 | ENSAMEG00000011536/ENSAMEG00000009601/ENSAMEG00000010197/ENSAMEG00000006561/ENSAMEG00000008895/ENSAMEG00000018298/ENSAMEG00000017272/ENSAMEG00000013530/ENSAMEG00000013516/ENSAMEG00000002331/ENSAMEG00000012327/ENSAMEG00000016417/ENSAMEG00000005545 | 13 |
| GO:0003824 | catalytic activity | 2.89E-04 | 1.27E-02 | 1.13E-02 | ENSAMEG00000010324/ENSAMEG00000004732/ENSAMEG00000001684/ENSAMEG00000008843/ENSAMEG00000007629/ENSAMEG00000012518/ENSAMEG00000011122/ENSAMEG00000017691/ENSAMEG00000013098/ENSAMEG00000014144/ENSAMEG00000005491/ENSAMEG00000016920/ENSAMEG00000016574/ENSAMEG00000008883/ENSAMEG00000008790/ENSAMEG00000015963/ENSAMEG00000005193/ENSAMEG00000012056/ENSAMEG00000013351/ENSAMEG00000018730/ENSAMEG00000004467/ENSAMEG00000016662/ENSAMEG00000011706/ENSAMEG00000009102/ENSAMEG00000009787/ENSAMEG00000016700/ENSAMEG00000011973/ENSAMEG00000000028/ENSAMEG00000014965/ENSAMEG00000012013/ENSAMEG00000004846/ENSAMEG00000010195/ENSAMEG00000004675/ENSAMEG00000003009 | 34 |
| GO:0030170 | pyridoxal phosphate binding | 3.08E-04 | 1.32E-02 | 1.17E-02 | ENSAMEG00000008632/ENSAMEG00000005491/ENSAMEG00000008883/ENSAMEG00000017831/ENSAMEG00000009102/ENSAMEG00000016700/ENSAMEG00000000028/ENSAMEG00000010195/ENSAMEG00000004675 | 9 |
| GO:0005518 | collagen binding | 3.25E-04 | 1.35E-02 | 1.20E-02 | ENSAMEG00000014674/ENSAMEG00000014152/ENSAMEG00000006561/ENSAMEG00000004377/ENSAMEG00000001264/ENSAMEG00000002331/ENSAMEG00000006251/ENSAMEG00000014069 | 8 |
| GO:0002376 | immune system process | 3.67E-04 | 1.48E-02 | 1.32E-02 | ENSAMEG00000002390/ENSAMEG00000002352/ENSAMEG00000018451/ENSAMEG00000001952/ENSAMEG00000002099/ENSAMEG00000002361/ENSAMEG00000002342 | 7 |
| GO:0006508 | proteolysis | 4.08E-04 | 1.60E-02 | 1.43E-02 | ENSAMEG00000002287/ENSAMEG00000011536/ENSAMEG00000015921/ENSAMEG00000018429/ENSAMEG00000003004/ENSAMEG00000010813/ENSAMEG00000005123/ENSAMEG00000006287/ENSAMEG00000003447/ENSAMEG00000013878/ENSAMEG00000009070/ENSAMEG00000017754/ENSAMEG00000016581/ENSAMEG00000002945/ENSAMEG00000005307/ENSAMEG00000014850/ENSAMEG00000010855/ENSAMEG00000016398/ENSAMEG00000011357/ENSAMEG00000012868/ENSAMEG00000017272/ENSAMEG00000001264/ENSAMEG00000013566/ENSAMEG00000013559/ENSAMEG00000007801/ENSAMEG00000004751/ENSAMEG00000015492/ENSAMEG00000015089/ENSAMEG00000000467/ENSAMEG00000016417/ENSAMEG00000010760/ENSAMEG00000009461/ENSAMEG00000013144/ENSAMEG00000006251/ENSAMEG00000005545/ENSAMEG00000007262/ENSAMEG00000006033/ENSAMEG00000006522 | 38 |
| GO:0004364 | glutathione transferase activity | 4.46E-04 | 1.70E-02 | 1.52E-02 | ENSAMEG00000005027/ENSAMEG00000005016/ENSAMEG00000004970/ENSAMEG00000013796/ENSAMEG00000004985 | 5 |
| GO:0030336 | negative regulation of cell migration | 5.25E-04 | 1.95E-02 | 1.74E-02 | ENSAMEG00000003357/ENSAMEG00000004654/ENSAMEG00000014110/ENSAMEG00000006196/ENSAMEG00000002851/ENSAMEG00000011357/ENSAMEG00000017636/ENSAMEG00000011973/ENSAMEG00000002621/ENSAMEG00000000455/ENSAMEG00000001811 | 11 |
| GO:0046426 | negative regulation of receptor signaling pathway via JAK-STAT | 6.00E-04 | 2.15E-02 | 1.92E-02 | ENSAMEG00000019175/ENSAMEG00000012849/ENSAMEG00000001343/ENSAMEG00000009824 | 4 |
| GO:0050431 | transforming growth factor beta binding | 6.09E-04 | 2.15E-02 | 1.92E-02 | ENSAMEG00000016910/ENSAMEG00000017473/ENSAMEG00000012327/ENSAMEG00000011706/ENSAMEG00000001811 | 5 |
| GO:0030324 | lung development | 6.33E-04 | 2.19E-02 | 1.95E-02 | ENSAMEG00000018351/ENSAMEG00000015855/ENSAMEG00000008842/ENSAMEG00000010197/ENSAMEG00000006561/ENSAMEG00000018266/ENSAMEG00000015317/ENSAMEG00000001820/ENSAMEG00000012157 | 9 |
| GO:0005201 | extracellular matrix structural constituent | 8.88E-04 | 2.89E-02 | 2.57E-02 | ENSAMEG00000000687/ENSAMEG00000003817/ENSAMEG00000012158/ENSAMEG00000013964/ENSAMEG00000002289/ENSAMEG00000004084 | 6 |
| GO:0048146 | positive regulation of fibroblast proliferation | 8.88E-04 | 2.89E-02 | 2.57E-02 | ENSAMEG00000018351/ENSAMEG00000004654/ENSAMEG00000004441/ENSAMEG00000000266/ENSAMEG00000001256/ENSAMEG00000014348 | 6 |
| GO:0030308 | negative regulation of cell growth | 8.95E-04 | 2.89E-02 | 2.57E-02 | ENSAMEG00000017643/ENSAMEG00000014110/ENSAMEG00000009648/ENSAMEG00000001013/ENSAMEG00000006196/ENSAMEG00000011357/ENSAMEG00000000266/ENSAMEG00000020088/ENSAMEG00000011706/ENSAMEG00000003009/ENSAMEG00000001811 | 11 |
| GO:0070062 | extracellular exosome | 9.35E-04 | 2.90E-02 | 2.59E-02 | ENSAMEG00000014033/ENSAMEG00000016542/ENSAMEG00000014604/ENSAMEG00000012054/ENSAMEG00000011357/ENSAMEG00000005696/ENSAMEG00000015089/ENSAMEG00000002621/ENSAMEG00000017756 | 9 |
| GO:0009617 | response to bacterium | 9.40E-04 | 2.90E-02 | 2.59E-02 | ENSAMEG00000012726/ENSAMEG00000014563/ENSAMEG00000002945/ENSAMEG00000015385/ENSAMEG00000008236/ENSAMEG00000001343/ENSAMEG00000001820/ENSAMEG00000005154/ENSAMEG00000010658/ENSAMEG00000006033 | 10 |
| GO:0002020 | protease binding | 1.06E-03 | 3.15E-02 | 2.81E-02 | ENSAMEG00000006561/ENSAMEG00000013869/ENSAMEG00000009345/ENSAMEG00000015492/ENSAMEG00000011447/ENSAMEG00000012157/ENSAMEG00000014114/ENSAMEG00000006033/ENSAMEG00000002621 | 9 |
| GO:0050873 | brown fat cell differentiation | 1.06E-03 | 3.15E-02 | 2.81E-02 | ENSAMEG00000016542/ENSAMEG00000014563/ENSAMEG00000015532/ENSAMEG00000015933/ENSAMEG00000011054 | 5 |
| GO:0008305 | integrin complex | 1.09E-03 | 3.16E-02 | 2.82E-02 | ENSAMEG00000017867/ENSAMEG00000016286/ENSAMEG00000017585/ENSAMEG00000016294/ENSAMEG00000012157/ENSAMEG00000009619 | 6 |
| GO:0031668 | cellular response to extracellular stimulus | 1.32E-03 | 3.68E-02 | 3.28E-02 | ENSAMEG00000009809/ENSAMEG00000000266/ENSAMEG00000011737/ENSAMEG00000007834 | 4 |
| GO:0071347 | cellular response to interleukin-1 | 1.32E-03 | 3.68E-02 | 3.28E-02 | ENSAMEG00000014902/ENSAMEG00000013578/ENSAMEG00000005780/ENSAMEG00000000376/ENSAMEG00000011706/ENSAMEG00000004830 | 6 |
| GO:0005520 | insulin-like growth factor binding | 1.37E-03 | 3.68E-02 | 3.28E-02 | ENSAMEG00000003357/ENSAMEG00000012135/ENSAMEG00000017421/ENSAMEG00000008607/ENSAMEG00000014788 | 5 |
| GO:0008202 | steroid metabolic process | 1.37E-03 | 3.68E-02 | 3.28E-02 | ENSAMEG00000003132/ENSAMEG00000011824/ENSAMEG00000017636/ENSAMEG00000012592/ENSAMEG00000016630 | 5 |
| GO:0051289 | protein homotetramerization | 1.68E-03 | 4.38E-02 | 3.91E-02 | ENSAMEG00000015468/ENSAMEG00000012753/ENSAMEG00000013025/ENSAMEG00000010228/ENSAMEG00000000022/ENSAMEG00000000028/ENSAMEG00000004675/ENSAMEG00000003009 | 8 |
| GO:0007229 | integrin-mediated signaling pathway | 1.69E-03 | 4.38E-02 | 3.91E-02 | ENSAMEG00000017867/ENSAMEG00000007760/ENSAMEG00000016286/ENSAMEG00000017585/ENSAMEG00000016294/ENSAMEG00000011357/ENSAMEG00000018076/ENSAMEG00000012157/ENSAMEG00000009619 | 9 |
| GO:0001516 | prostaglandin biosynthetic process | 1.84E-03 | 4.42E-02 | 3.94E-02 | ENSAMEG00000004654/ENSAMEG00000015532/ENSAMEG00000013578/ENSAMEG00000013340 | 4 |
| GO:0004467 | long-chain fatty acid-CoA ligase activity | 1.84E-03 | 4.42E-02 | 3.94E-02 | ENSAMEG00000008843/ENSAMEG00000012518/ENSAMEG00000012056/ENSAMEG00000004846 | 4 |
| GO:0042310 | vasoconstriction | 1.84E-03 | 4.42E-02 | 3.94E-02 | ENSAMEG00000016787/ENSAMEG00000001343/ENSAMEG00000002011/ENSAMEG00000005640 | 4 |
| GO:0046697 | decidualization | 1.84E-03 | 4.42E-02 | 3.94E-02 | ENSAMEG00000015532/ENSAMEG00000013578/ENSAMEG00000018076/ENSAMEG00000006251 | 4 |
| GO:0001666 | response to hypoxia | 1.89E-03 | 4.42E-02 | 3.94E-02 | ENSAMEG00000017263/ENSAMEG00000007445/ENSAMEG00000005307/ENSAMEG00000002179/ENSAMEG00000001343/ENSAMEG00000019096/ENSAMEG00000005154/ENSAMEG00000016417/ENSAMEG00000010258 | 9 |
| GO:0006935 | chemotaxis | 1.89E-03 | 4.42E-02 | 3.94E-02 | ENSAMEG00000018351/ENSAMEG00000004961/ENSAMEG00000016126/ENSAMEG00000014902/ENSAMEG00000013850/ENSAMEG00000005780/ENSAMEG00000014335/ENSAMEG00000019454/ENSAMEG00000004830 | 9 |
| GO:0007623 | circadian rhythm | 2.02E-03 | 4.66E-02 | 4.15E-02 | ENSAMEG00000007114/ENSAMEG00000009673/ENSAMEG00000016787/ENSAMEG00000013351/ENSAMEG00000005154/ENSAMEG00000016060/ENSAMEG00000010817 | 7 |
| GO:0046718 | viral entry into host cell | 2.16E-03 | 4.90E-02 | 4.37E-02 | ENSAMEG00000015921/ENSAMEG00000004441/ENSAMEG00000011737/ENSAMEG00000011706/ENSAMEG00000006251 | 5 |
