# Supplementary material for: Gene expression profiles during postnatal development of the liver and pancreas in giant pandas
Source: Aging (Albany NY). 2020 Aug 15;12(15):15705–29. doi: 10.18632/aging.103783 (PMC7467380; doi:10.18632/aging.103783)
Supplement: Supplementary Table 3 [file aging-12-103783-s010..docx]

**Supplementary Table 3. Significantly enriched KEGG pathways for up-regulated DEGs in liver adult group compared with liver suckling group.**

| **ID** | **Description** | **pvalue** | **p.adjust** | **qvalue** | **geneID** | **Count** |
| --- | --- | --- | --- | --- | --- | --- |
| aml05204 | Chemical carcinogenesis [PATH:aml05204] | 2.37E-10 | 6.62E-08 | 5.57E-08 | ENSAMEG00000011960/ENSAMEG00000005027/ENSAMEG00000005016/ENSAMEG00000004376/ENSAMEG00000006243/ENSAMEG00000008842/ENSAMEG00000003132/ENSAMEG00000015532/ENSAMEG00000015358/ENSAMEG00000016104/ENSAMEG00000011824/ENSAMEG00000005398/ENSAMEG00000013784/ENSAMEG00000004970/ENSAMEG00000013796/ENSAMEG00000004985/ENSAMEG00000011749 | 17 |
| aml04610 | Complement and coagulation cascades [PATH:aml04610] | 1.38E-09 | 1.92E-07 | 1.61E-07 | ENSAMEG00000015230/ENSAMEG00000017524/ENSAMEG00000000141/ENSAMEG00000015352/ENSAMEG00000009692/ENSAMEG00000014526/ENSAMEG00000003480/ENSAMEG00000019083/ENSAMEG00000017094/ENSAMEG00000005307/ENSAMEG00000013869/ENSAMEG00000013566/ENSAMEG00000018463/ENSAMEG00000004240/ENSAMEG00000009345/ENSAMEG00000001045/ENSAMEG00000013144/ENSAMEG00000018459/ENSAMEG00000002621 | 19 |
| aml00260 | Glycine, serine and threonine metabolism [PATH:aml00260] | 2.99E-06 | 2.77E-04 | 2.33E-04 | ENSAMEG00000012753/ENSAMEG00000008632/ENSAMEG00000012647/ENSAMEG00000008612/ENSAMEG00000005739/ENSAMEG00000005696/ENSAMEG00000009102/ENSAMEG00000016700/ENSAMEG00000000028/ENSAMEG00000010195/ENSAMEG00000004675 | 11 |
| aml00140 | Steroid hormone biosynthesis [PATH:aml00140] | 3.97E-06 | 2.77E-04 | 2.33E-04 | ENSAMEG00000017684/ENSAMEG00000004376/ENSAMEG00000006243/ENSAMEG00000008842/ENSAMEG00000003132/ENSAMEG00000016104/ENSAMEG00000011824/ENSAMEG00000018008/ENSAMEG00000009440/ENSAMEG00000011617/ENSAMEG00000011749 | 11 |
| aml00982 | Drug metabolism - cytochrome P450 [PATH:aml00982] | 5.21E-06 | 2.91E-04 | 2.45E-04 | ENSAMEG00000005027/ENSAMEG00000005016/ENSAMEG00000008842/ENSAMEG00000003132/ENSAMEG00000005445/ENSAMEG00000013784/ENSAMEG00000017038/ENSAMEG00000004970/ENSAMEG00000013796/ENSAMEG00000004985/ENSAMEG00000011749 | 11 |
| aml04512 | ECM-receptor interaction [PATH:aml04512] | 1.11E-05 | 4.47E-04 | 3.76E-04 | ENSAMEG00000003817/ENSAMEG00000009150/ENSAMEG00000003156/ENSAMEG00000017867/ENSAMEG00000017585/ENSAMEG00000013964/ENSAMEG00000002327/ENSAMEG00000002289/ENSAMEG00000002331/ENSAMEG00000004084/ENSAMEG00000012157/ENSAMEG00000004524/ENSAMEG00000010085/ENSAMEG00000015074/ENSAMEG00000009619 | 15 |
| aml00980 | Metabolism of xenobiotics by cytochrome P450 [PATH:aml00980] | 1.12E-05 | 4.47E-04 | 3.76E-04 | ENSAMEG00000011960/ENSAMEG00000005027/ENSAMEG00000005016/ENSAMEG00000008842/ENSAMEG00000003132/ENSAMEG00000005398/ENSAMEG00000013784/ENSAMEG00000004970/ENSAMEG00000013796/ENSAMEG00000004985/ENSAMEG00000011749 | 11 |
| aml00983 | Drug metabolism - other enzymes [PATH:aml00983] | 1.73E-05 | 6.04E-04 | 5.09E-04 | ENSAMEG00000005027/ENSAMEG00000004732/ENSAMEG00000005016/ENSAMEG00000003132/ENSAMEG00000013784/ENSAMEG00000004970/ENSAMEG00000016662/ENSAMEG00000008351/ENSAMEG00000013796/ENSAMEG00000004985/ENSAMEG00000003009/ENSAMEG00000011749 | 12 |
| aml05418 | Fluid shear stress and atherosclerosis [PATH:aml05418] | 2.05E-05 | 6.37E-04 | 5.36E-04 | ENSAMEG00000005027/ENSAMEG00000005016/ENSAMEG00000019083/ENSAMEG00000005307/ENSAMEG00000011201/ENSAMEG00000001347/ENSAMEG00000005780/ENSAMEG00000008746/ENSAMEG00000013784/ENSAMEG00000013088/ENSAMEG00000007357/ENSAMEG00000015317/ENSAMEG00000001343/ENSAMEG00000000376/ENSAMEG00000016417/ENSAMEG00000004970/ENSAMEG00000013796/ENSAMEG00000004985/ENSAMEG00000006522 | 19 |
| aml05145 | Toxoplasmosis [PATH:aml05145] | 7.61E-05 | 2.12E-03 | 1.79E-03 | ENSAMEG00000009150/ENSAMEG00000002390/ENSAMEG00000002352/ENSAMEG00000001952/ENSAMEG00000002099/ENSAMEG00000013964/ENSAMEG00000002327/ENSAMEG00000002289/ENSAMEG00000002342/ENSAMEG00000015629/ENSAMEG00000007357/ENSAMEG00000000376/ENSAMEG00000005154/ENSAMEG00000006039/ENSAMEG00000018827 | 15 |
| aml05416 | Viral myocarditis [PATH:aml05416] | 8.49E-05 | 2.15E-03 | 1.81E-03 | ENSAMEG00000002302/ENSAMEG00000009150/ENSAMEG00000002390/ENSAMEG00000002352/ENSAMEG00000001952/ENSAMEG00000008182/ENSAMEG00000002099/ENSAMEG00000002342/ENSAMEG00000001343/ENSAMEG00000006039 | 10 |
| aml00500 | Starch and sucrose metabolism [PATH:aml00500] | 1.32E-04 | 3.08E-03 | 2.59E-03 | ENSAMEG00000010324/ENSAMEG00000006494/ENSAMEG00000001684/ENSAMEG00000007629/ENSAMEG00000011122/ENSAMEG00000017601/ENSAMEG00000017831/ENSAMEG00000014965 | 8 |
| aml04612 | Antigen processing and presentation [PATH:aml04612] | 1.53E-04 | 3.29E-03 | 2.77E-03 | ENSAMEG00000004654/ENSAMEG00000002302/ENSAMEG00000002390/ENSAMEG00000002352/ENSAMEG00000001952/ENSAMEG00000002099/ENSAMEG00000002342/ENSAMEG00000006251/ENSAMEG00000018827/ENSAMEG00000006522 | 10 |
| aml05150 | Staphylococcus aureus infection [PATH:aml05150] | 2.15E-04 | 4.11E-03 | 3.46E-03 | ENSAMEG00000012726/ENSAMEG00000002390/ENSAMEG00000001682/ENSAMEG00000002352/ENSAMEG00000001952/ENSAMEG00000002099/ENSAMEG00000002342/ENSAMEG00000013566/ENSAMEG00000018463/ENSAMEG00000001045/ENSAMEG00000005526 | 11 |
| aml00590 | Arachidonic acid metabolism [PATH:aml00590] | 2.21E-04 | 4.11E-03 | 3.46E-03 | ENSAMEG00000006243/ENSAMEG00000013878/ENSAMEG00000003132/ENSAMEG00000015532/ENSAMEG00000013578/ENSAMEG00000016104/ENSAMEG00000011824/ENSAMEG00000013340/ENSAMEG00000005398/ENSAMEG00000003219 | 10 |
| aml04658 | Th1 and Th2 cell differentiation [PATH:aml04658] | 3.47E-04 | 6.04E-03 | 5.08E-03 | ENSAMEG00000002390/ENSAMEG00000002352/ENSAMEG00000016821/ENSAMEG00000013155/ENSAMEG00000001952/ENSAMEG00000013141/ENSAMEG00000002099/ENSAMEG00000007672/ENSAMEG00000002342/ENSAMEG00000015629/ENSAMEG00000000376/ENSAMEG00000004069 | 12 |
| aml04510 | Focal adhesion [PATH:aml04510] | 3.83E-04 | 6.29E-03 | 5.29E-03 | ENSAMEG00000003817/ENSAMEG00000018351/ENSAMEG00000002628/ENSAMEG00000009150/ENSAMEG00000003156/ENSAMEG00000004961/ENSAMEG00000017867/ENSAMEG00000016581/ENSAMEG00000017585/ENSAMEG00000013964/ENSAMEG00000000974/ENSAMEG00000002327/ENSAMEG00000002289/ENSAMEG00000001347/ENSAMEG00000002331/ENSAMEG00000001343/ENSAMEG00000004084/ENSAMEG00000012157/ENSAMEG00000004524/ENSAMEG00000015074/ENSAMEG00000009619 | 21 |
| aml05222 | Small cell lung cancer [PATH:aml05222] | 4.84E-04 | 7.50E-03 | 6.31E-03 | ENSAMEG00000005608/ENSAMEG00000003817/ENSAMEG00000009150/ENSAMEG00000015532/ENSAMEG00000013964/ENSAMEG00000002327/ENSAMEG00000002289/ENSAMEG00000000266/ENSAMEG00000015629/ENSAMEG00000004084/ENSAMEG00000005154/ENSAMEG00000012157/ENSAMEG00000006039 | 13 |
| aml05323 | Rheumatoid arthritis [PATH:aml05323] | 5.14E-04 | 7.54E-03 | 6.35E-03 | ENSAMEG00000002390/ENSAMEG00000002352/ENSAMEG00000014902/ENSAMEG00000001952/ENSAMEG00000002099/ENSAMEG00000013850/ENSAMEG00000005780/ENSAMEG00000014335/ENSAMEG00000001264/ENSAMEG00000002342/ENSAMEG00000004830/ENSAMEG00000006522 | 12 |
| aml04514 | Cell adhesion molecules (CAMs) [PATH:aml04514] | 5.45E-04 | 7.60E-03 | 6.39E-03 | ENSAMEG00000008621/ENSAMEG00000002302/ENSAMEG00000002390/ENSAMEG00000002352/ENSAMEG00000000245/ENSAMEG00000017867/ENSAMEG00000007493/ENSAMEG00000001952/ENSAMEG00000002099/ENSAMEG00000012707/ENSAMEG00000002342/ENSAMEG00000019949/ENSAMEG00000019096/ENSAMEG00000007109/ENSAMEG00000018735 | 15 |
| aml04940 | Type I diabetes mellitus [PATH:aml04940] | 6.81E-04 | 9.04E-03 | 7.61E-03 | ENSAMEG00000002302/ENSAMEG00000005123/ENSAMEG00000002390/ENSAMEG00000002352/ENSAMEG00000001952/ENSAMEG00000002099/ENSAMEG00000002342 | 7 |
| aml05332 | Graft-versus-host disease [PATH:aml05332] | 8.26E-04 | 1.05E-02 | 8.82E-03 | ENSAMEG00000002302/ENSAMEG00000002390/ENSAMEG00000002352/ENSAMEG00000001952/ENSAMEG00000002099/ENSAMEG00000002342 | 6 |
| aml00830 | Retinol metabolism [PATH:aml00830] | 9.11E-04 | 1.10E-02 | 9.29E-03 | ENSAMEG00000004376/ENSAMEG00000006243/ENSAMEG00000008842/ENSAMEG00000016104/ENSAMEG00000011824/ENSAMEG00000005445/ENSAMEG00000013288/ENSAMEG00000011749 | 8 |
| aml05330 | Allograft rejection [PATH:aml05330] | 1.07E-03 | 1.24E-02 | 1.04E-02 | ENSAMEG00000002302/ENSAMEG00000002390/ENSAMEG00000002352/ENSAMEG00000001952/ENSAMEG00000002099/ENSAMEG00000002342 | 6 |
| aml04060 | Cytokine-cytokine receptor interaction [PATH:aml04060] | 1.15E-03 | 1.29E-02 | 1.08E-02 | ENSAMEG00000002226/ENSAMEG00000017643/ENSAMEG00000016126/ENSAMEG00000014902/ENSAMEG00000001739/ENSAMEG00000006523/ENSAMEG00000013850/ENSAMEG00000005780/ENSAMEG00000014335/ENSAMEG00000006426/ENSAMEG00000003826/ENSAMEG00000003557/ENSAMEG00000013088/ENSAMEG00000015317/ENSAMEG00000000999/ENSAMEG00000019454/ENSAMEG00000002611/ENSAMEG00000004069/ENSAMEG00000003697/ENSAMEG00000004830/ENSAMEG00000006524/ENSAMEG00000001811 | 22 |
| aml00591 | Linoleic acid metabolism [PATH:aml00591] | 1.36E-03 | 1.46E-02 | 1.23E-02 | ENSAMEG00000004376/ENSAMEG00000006243/ENSAMEG00000008842/ENSAMEG00000003132/ENSAMEG00000016104/ENSAMEG00000003219 | 6 |
| aml04640 | Hematopoietic cell lineage [PATH:aml04640] | 1.82E-03 | 1.88E-02 | 1.58E-02 | ENSAMEG00000002390/ENSAMEG00000002352/ENSAMEG00000014604/ENSAMEG00000013155/ENSAMEG00000001952/ENSAMEG00000013141/ENSAMEG00000002099/ENSAMEG00000002342/ENSAMEG00000013088/ENSAMEG00000012157/ENSAMEG00000004069 | 11 |
| aml04659 | Th17 cell differentiation [PATH:aml04659] | 2.21E-03 | 2.21E-02 | 1.86E-02 | ENSAMEG00000002390/ENSAMEG00000002352/ENSAMEG00000013155/ENSAMEG00000001952/ENSAMEG00000013141/ENSAMEG00000002099/ENSAMEG00000007672/ENSAMEG00000002342/ENSAMEG00000015629/ENSAMEG00000010559/ENSAMEG00000000376/ENSAMEG00000004069 | 12 |
| aml05146 | Amoebiasis [PATH:aml05146] | 2.44E-03 | 2.27E-02 | 1.91E-02 | ENSAMEG00000003817/ENSAMEG00000009150/ENSAMEG00000010227/ENSAMEG00000013964/ENSAMEG00000017094/ENSAMEG00000002327/ENSAMEG00000002289/ENSAMEG00000014335/ENSAMEG00000013088/ENSAMEG00000004084/ENSAMEG00000005154/ENSAMEG00000004830 | 12 |
| aml05322 | Systemic lupus erythematosus [PATH:aml05322] | 2.44E-03 | 2.27E-02 | 1.91E-02 | ENSAMEG00000015230/ENSAMEG00000015352/ENSAMEG00000002390/ENSAMEG00000002352/ENSAMEG00000001952/ENSAMEG00000002099/ENSAMEG00000017094/ENSAMEG00000002342/ENSAMEG00000013566/ENSAMEG00000019926/ENSAMEG00000001045/ENSAMEG00000019745 | 12 |
| aml04151 | PI3K-Akt signaling pathway [PATH:aml04151] | 3.13E-03 | 2.77E-02 | 2.33E-02 | ENSAMEG00000003817/ENSAMEG00000018351/ENSAMEG00000009150/ENSAMEG00000003156/ENSAMEG00000004961/ENSAMEG00000017867/ENSAMEG00000010197/ENSAMEG00000004527/ENSAMEG00000016581/ENSAMEG00000014090/ENSAMEG00000017585/ENSAMEG00000011340/ENSAMEG00000013964/ENSAMEG00000000974/ENSAMEG00000002327/ENSAMEG00000002289/ENSAMEG00000015983/ENSAMEG00000003826/ENSAMEG00000000266/ENSAMEG00000002331/ENSAMEG00000004084/ENSAMEG00000012157/ENSAMEG00000014348/ENSAMEG00000004069/ENSAMEG00000004524/ENSAMEG00000015074/ENSAMEG00000009619 | 27 |
| aml04672 | Intestinal immune network for IgA production [PATH:aml04672] | 3.17E-03 | 2.77E-02 | 2.33E-02 | ENSAMEG00000002390/ENSAMEG00000002352/ENSAMEG00000001952/ENSAMEG00000002099/ENSAMEG00000013850/ENSAMEG00000002342 | 6 |
| aml05140 | Leishmaniasis [PATH:aml05140] | 3.47E-03 | 2.93E-02 | 2.47E-02 | ENSAMEG00000002390/ENSAMEG00000002352/ENSAMEG00000015532/ENSAMEG00000001952/ENSAMEG00000002099/ENSAMEG00000002342/ENSAMEG00000015629/ENSAMEG00000000376/ENSAMEG00000005154 | 9 |
| aml05020 | Prion diseases [PATH:aml05020] | 3.82E-03 | 3.05E-02 | 2.57E-02 | ENSAMEG00000015230/ENSAMEG00000015352/ENSAMEG00000014902/ENSAMEG00000017094/ENSAMEG00000002289/ENSAMEG00000001045 | 6 |
| aml05320 | Autoimmune thyroid disease [PATH:aml05320] | 3.82E-03 | 3.05E-02 | 2.57E-02 | ENSAMEG00000002302/ENSAMEG00000002390/ENSAMEG00000002352/ENSAMEG00000001952/ENSAMEG00000002099/ENSAMEG00000002342 | 6 |
| aml05310 | Asthma [PATH:aml05310] | 4.34E-03 | 3.36E-02 | 2.83E-02 | ENSAMEG00000002390/ENSAMEG00000002352/ENSAMEG00000001952/ENSAMEG00000002099/ENSAMEG00000002342 | 5 |
| aml04974 | Protein digestion and absorption [PATH:aml04974] | 5.02E-03 | 3.78E-02 | 3.18E-02 | ENSAMEG00000003817/ENSAMEG00000015921/ENSAMEG00000002184/ENSAMEG00000016366/ENSAMEG00000012158/ENSAMEG00000015783/ENSAMEG00000015089/ENSAMEG00000004084/ENSAMEG00000013144/ENSAMEG00000016828/ENSAMEG00000004524 | 11 |
| aml05200 | Pathways in cancer [PATH:aml05200] | 5.33E-03 | 3.91E-02 | 3.29E-02 | ENSAMEG00000005027/ENSAMEG00000005608/ENSAMEG00000003817/ENSAMEG00000005016/ENSAMEG00000018351/ENSAMEG00000009150/ENSAMEG00000004961/ENSAMEG00000015532/ENSAMEG00000016821/ENSAMEG00000010197/ENSAMEG00000004527/ENSAMEG00000016581/ENSAMEG00000013964/ENSAMEG00000000974/ENSAMEG00000002327/ENSAMEG00000016574/ENSAMEG00000002289/ENSAMEG00000013850/ENSAMEG00000000266/ENSAMEG00000001256/ENSAMEG00000015629/ENSAMEG00000013784/ENSAMEG00000015317/ENSAMEG00000017625/ENSAMEG00000004084/ENSAMEG00000005154/ENSAMEG00000016417/ENSAMEG00000004970/ENSAMEG00000012157/ENSAMEG00000006039/ENSAMEG00000002011/ENSAMEG00000004069/ENSAMEG00000013796/ENSAMEG00000004830/ENSAMEG00000004985/ENSAMEG00000005640 | 36 |
| aml00380 | Tryptophan metabolism [PATH:aml00380] | 5.63E-03 | 4.03E-02 | 3.39E-02 | ENSAMEG00000014033/ENSAMEG00000018686/ENSAMEG00000016329/ENSAMEG00000015468/ENSAMEG00000008842/ENSAMEG00000005445/ENSAMEG00000009730 | 7 |
