# Supplementary material for: Gene expression profiles during postnatal development of the liver and pancreas in giant pandas
Source: Aging (Albany NY). 2020 Aug 15;12(15):15705–29. doi: 10.18632/aging.103783 (PMC7467380; doi:10.18632/aging.103783)
Supplement: Supplementary Table 4 [file aging-12-103783-s014..docx]

**Supplementary Table 4. Significantly enriched GO categories for down-regulated DEGs in liver adult group compared with liver suckling group.**

| **ID** | **Description** | **pvalue** | **p.adjust** | **qvalue** | **geneID** | **Count** |
| --- | --- | --- | --- | --- | --- | --- |
| GO:0006260 | DNA replication | 1.78E-21 | 3.28E-18 | 2.84E-18 | ENSAMEG00000014998/ENSAMEG00000001634/ENSAMEG00000005841/ENSAMEG00000018028/ENSAMEG00000009254/ENSAMEG00000014870/ENSAMEG00000018347/ENSAMEG00000013505/ENSAMEG00000008540/ENSAMEG00000015091/ENSAMEG00000018281/ENSAMEG00000013454/ENSAMEG00000005460/ENSAMEG00000014758/ENSAMEG00000013104/ENSAMEG00000009547/ENSAMEG00000000792/ENSAMEG00000011150/ENSAMEG00000013843/ENSAMEG00000001421/ENSAMEG00000005102/ENSAMEG00000017155/ENSAMEG00000013111/ENSAMEG00000009089/ENSAMEG00000011132/ENSAMEG00000017258/ENSAMEG00000000475/ENSAMEG00000002731/ENSAMEG00000017565/ENSAMEG00000000109/ENSAMEG00000008804/ENSAMEG00000004198/ENSAMEG00000008231/ENSAMEG00000007706/ENSAMEG00000009566/ENSAMEG00000012243/ENSAMEG00000011474/ENSAMEG00000014866 | 38 |
| GO:0000776 | kinetochore | 6.53E-20 | 6.02E-17 | 5.21E-17 | ENSAMEG00000015869/ENSAMEG00000014095/ENSAMEG00000009532/ENSAMEG00000017537/ENSAMEG00000006525/ENSAMEG00000009491/ENSAMEG00000010069/ENSAMEG00000001187/ENSAMEG00000005342/ENSAMEG00000012771/ENSAMEG00000017084/ENSAMEG00000002580/ENSAMEG00000000913/ENSAMEG00000014549/ENSAMEG00000014232/ENSAMEG00000016838/ENSAMEG00000006979/ENSAMEG00000014641/ENSAMEG00000017203/ENSAMEG00000009168/ENSAMEG00000000371/ENSAMEG00000012392/ENSAMEG00000003251/ENSAMEG00000014068/ENSAMEG00000005053/ENSAMEG00000001352/ENSAMEG00000002642/ENSAMEG00000003458/ENSAMEG00000005149/ENSAMEG00000003155/ENSAMEG00000005084/ENSAMEG00000002231/ENSAMEG00000008231/ENSAMEG00000009521/ENSAMEG00000009192/ENSAMEG00000000707 | 36 |
| GO:0005813 | centrosome | 6.86E-19 | 4.21E-16 | 3.65E-16 | ENSAMEG00000017945/ENSAMEG00000010767/ENSAMEG00000003520/ENSAMEG00000001259/ENSAMEG00000007168/ENSAMEG00000002675/ENSAMEG00000010721/ENSAMEG00000010069/ENSAMEG00000005225/ENSAMEG00000001187/ENSAMEG00000000013/ENSAMEG00000013455/ENSAMEG00000014201/ENSAMEG00000008931/ENSAMEG00000011443/ENSAMEG00000008991/ENSAMEG00000008540/ENSAMEG00000000953/ENSAMEG00000000913/ENSAMEG00000009390/ENSAMEG00000004603/ENSAMEG00000008648/ENSAMEG00000002666/ENSAMEG00000001549/ENSAMEG00000014232/ENSAMEG00000006979/ENSAMEG00000003270/ENSAMEG00000002590/ENSAMEG00000003215/ENSAMEG00000013840/ENSAMEG00000010293/ENSAMEG00000004713/ENSAMEG00000007412/ENSAMEG00000010512/ENSAMEG00000000503/ENSAMEG00000009369/ENSAMEG00000004449/ENSAMEG00000012848/ENSAMEG00000011827/ENSAMEG00000012346/ENSAMEG00000003208/ENSAMEG00000012392/ENSAMEG00000009089/ENSAMEG00000012375/ENSAMEG00000019816/ENSAMEG00000016790/ENSAMEG00000001233/ENSAMEG00000001855/ENSAMEG00000017216/ENSAMEG00000000309/ENSAMEG00000002721/ENSAMEG00000017740/ENSAMEG00000017370/ENSAMEG00000013304/ENSAMEG00000001250/ENSAMEG00000017365/ENSAMEG00000012237/ENSAMEG00000014294/ENSAMEG00000004426/ENSAMEG00000008333/ENSAMEG00000008731/ENSAMEG00000009136/ENSAMEG00000012644/ENSAMEG00000005084/ENSAMEG00000007023/ENSAMEG00000017110/ENSAMEG00000017811/ENSAMEG00000002382/ENSAMEG00000015487/ENSAMEG00000001092/ENSAMEG00000013211/ENSAMEG00000001007/ENSAMEG00000009852/ENSAMEG00000003166/ENSAMEG00000002777/ENSAMEG00000003586/ENSAMEG00000000983/ENSAMEG00000019673/ENSAMEG00000003168/ENSAMEG00000003720/ENSAMEG00000015890/ENSAMEG00000011164/ENSAMEG00000010861/ENSAMEG00000008828/ENSAMEG00000000800/ENSAMEG00000010566/ENSAMEG00000011958/ENSAMEG00000013710 | 88 |
| GO:0007059 | chromosome segregation | 3.60E-16 | 1.66E-13 | 1.44E-13 | ENSAMEG00000015869/ENSAMEG00000007989/ENSAMEG00000017537/ENSAMEG00000009491/ENSAMEG00000013933/ENSAMEG00000016113/ENSAMEG00000010069/ENSAMEG00000000137/ENSAMEG00000006653/ENSAMEG00000001187/ENSAMEG00000000171/ENSAMEG00000017084/ENSAMEG00000000913/ENSAMEG00000010875/ENSAMEG00000016838/ENSAMEG00000014641/ENSAMEG00000011337/ENSAMEG00000012346/ENSAMEG00000012524/ENSAMEG00000000371/ENSAMEG00000000280/ENSAMEG00000001233/ENSAMEG00000000501/ENSAMEG00000003458/ENSAMEG00000009521/ENSAMEG00000011643 | 26 |
| GO:0006281 | DNA repair | 2.01E-13 | 7.42E-11 | 6.43E-11 | ENSAMEG00000013934/ENSAMEG00000011867/ENSAMEG00000012174/ENSAMEG00000003715/ENSAMEG00000000171/ENSAMEG00000014825/ENSAMEG00000016965/ENSAMEG00000015091/ENSAMEG00000013454/ENSAMEG00000009390/ENSAMEG00000011892/ENSAMEG00000002666/ENSAMEG00000016051/ENSAMEG00000015678/ENSAMEG00000001064/ENSAMEG00000013843/ENSAMEG00000003608/ENSAMEG00000012957/ENSAMEG00000019971/ENSAMEG00000012375/ENSAMEG00000011132/ENSAMEG00000000475/ENSAMEG00000016796/ENSAMEG00000005053/ENSAMEG00000010406/ENSAMEG00000013304/ENSAMEG00000004324/ENSAMEG00000015826/ENSAMEG00000017712/ENSAMEG00000017063/ENSAMEG00000004483/ENSAMEG00000016518/ENSAMEG00000001905/ENSAMEG00000008438/ENSAMEG00000007706/ENSAMEG00000009566/ENSAMEG00000017905/ENSAMEG00000008960/ENSAMEG00000007626/ENSAMEG00000003657/ENSAMEG00000011474/ENSAMEG00000016419 | 42 |
| GO:0006974 | cellular response to DNA damage stimulus | 4.08E-13 | 1.25E-10 | 1.09E-10 | ENSAMEG00000007989/ENSAMEG00000002586/ENSAMEG00000001259/ENSAMEG00000005534/ENSAMEG00000000171/ENSAMEG00000014870/ENSAMEG00000016965/ENSAMEG00000013505/ENSAMEG00000015091/ENSAMEG00000009390/ENSAMEG00000011892/ENSAMEG00000016051/ENSAMEG00000013902/ENSAMEG00000001064/ENSAMEG00000011150/ENSAMEG00000003946/ENSAMEG00000004713/ENSAMEG00000013843/ENSAMEG00000003608/ENSAMEG00000012595/ENSAMEG00000015491/ENSAMEG00000012375/ENSAMEG00000000352/ENSAMEG00000010605/ENSAMEG00000001323/ENSAMEG00000008484/ENSAMEG00000010406/ENSAMEG00000000109/ENSAMEG00000013304/ENSAMEG00000012527/ENSAMEG00000016250/ENSAMEG00000008461/ENSAMEG00000001238/ENSAMEG00000003233/ENSAMEG00000004324/ENSAMEG00000015826/ENSAMEG00000011076/ENSAMEG00000014635/ENSAMEG00000017063/ENSAMEG00000004483/ENSAMEG00000007872/ENSAMEG00000016518/ENSAMEG00000007706/ENSAMEG00000002560/ENSAMEG00000009321/ENSAMEG00000017905/ENSAMEG00000002043/ENSAMEG00000011177/ENSAMEG00000000410/ENSAMEG00000007626/ENSAMEG00000016419/ENSAMEG00000000707/ENSAMEG00000005584 | 53 |
| GO:0000922 | spindle pole | 1.29E-12 | 3.39E-10 | 2.94E-10 | ENSAMEG00000015869/ENSAMEG00000014099/ENSAMEG00000013678/ENSAMEG00000017540/ENSAMEG00000014771/ENSAMEG00000010721/ENSAMEG00000001187/ENSAMEG00000000953/ENSAMEG00000000913/ENSAMEG00000014232/ENSAMEG00000006979/ENSAMEG00000013777/ENSAMEG00000010293/ENSAMEG00000004713/ENSAMEG00000007868/ENSAMEG00000004686/ENSAMEG00000010512/ENSAMEG00000012392/ENSAMEG00000019816/ENSAMEG00000001233/ENSAMEG00000001860/ENSAMEG00000009136/ENSAMEG00000017110/ENSAMEG00000002382/ENSAMEG00000001007/ENSAMEG00000000410/ENSAMEG00000010861 | 27 |
| GO:0032508 | DNA duplex unwinding | 1.06E-10 | 2.43E-08 | 2.11E-08 | ENSAMEG00000001634/ENSAMEG00000005841/ENSAMEG00000018028/ENSAMEG00000014870/ENSAMEG00000008540/ENSAMEG00000005460/ENSAMEG00000004713/ENSAMEG00000013843/ENSAMEG00000001421/ENSAMEG00000008785/ENSAMEG00000002148/ENSAMEG00000007469/ENSAMEG00000000475/ENSAMEG00000000109/ENSAMEG00000017063/ENSAMEG00000009566/ENSAMEG00000006307/ENSAMEG00000002043/ENSAMEG00000016233/ENSAMEG00000007626 | 20 |
| GO:0000278 | mitotic cell cycle | 1.71E-10 | 3.51E-08 | 3.04E-08 | ENSAMEG00000015869/ENSAMEG00000017537/ENSAMEG00000009491/ENSAMEG00000007168/ENSAMEG00000014358/ENSAMEG00000014232/ENSAMEG00000000135/ENSAMEG00000014641/ENSAMEG00000012466/ENSAMEG00000007868/ENSAMEG00000012346/ENSAMEG00000000371/ENSAMEG00000003841/ENSAMEG00000017275/ENSAMEG00000000280/ENSAMEG00000001855/ENSAMEG00000016483/ENSAMEG00000003458/ENSAMEG00000013304/ENSAMEG00000009566/ENSAMEG00000000983/ENSAMEG00000016594 | 22 |
| GO:0006270 | DNA replication initiation | 2.11E-10 | 3.89E-08 | 3.37E-08 | ENSAMEG00000014998/ENSAMEG00000001634/ENSAMEG00000005841/ENSAMEG00000011443/ENSAMEG00000013505/ENSAMEG00000008540/ENSAMEG00000009668/ENSAMEG00000011150/ENSAMEG00000007868/ENSAMEG00000001421/ENSAMEG00000005590/ENSAMEG00000010605 | 12 |
| GO:0003682 | chromatin binding | 3.95E-10 | 6.33E-08 | 5.48E-08 | ENSAMEG00000012588/ENSAMEG00000007989/ENSAMEG00000013934/ENSAMEG00000000014/ENSAMEG00000003520/ENSAMEG00000007193/ENSAMEG00000009254/ENSAMEG00000014870/ENSAMEG00000002791/ENSAMEG00000013454/ENSAMEG00000011892/ENSAMEG00000013104/ENSAMEG00000002114/ENSAMEG00000014221/ENSAMEG00000011823/ENSAMEG00000004713/ENSAMEG00000011337/ENSAMEG00000013940/ENSAMEG00000003344/ENSAMEG00000000371/ENSAMEG00000017674/ENSAMEG00000009089/ENSAMEG00000017275/ENSAMEG00000002954/ENSAMEG00000015260/ENSAMEG00000012914/ENSAMEG00000005053/ENSAMEG00000015524/ENSAMEG00000010057/ENSAMEG00000001685/ENSAMEG00000010406/ENSAMEG00000011651/ENSAMEG00000018450/ENSAMEG00000017438/ENSAMEG00000015779/ENSAMEG00000008496/ENSAMEG00000001319/ENSAMEG00000007608/ENSAMEG00000008231/ENSAMEG00000002912/ENSAMEG00000005029/ENSAMEG00000001160/ENSAMEG00000006307/ENSAMEG00000017905/ENSAMEG00000007181/ENSAMEG00000005490/ENSAMEG00000015675/ENSAMEG00000003974/ENSAMEG00000018342/ENSAMEG00000010211/ENSAMEG00000009674/ENSAMEG00000011474/ENSAMEG00000006185/ENSAMEG00000010799/ENSAMEG00000000707/ENSAMEG00000003040 | 56 |
| GO:0003678 | DNA helicase activity | 4.12E-10 | 6.33E-08 | 5.48E-08 | ENSAMEG00000014998/ENSAMEG00000001634/ENSAMEG00000005841/ENSAMEG00000018028/ENSAMEG00000008540/ENSAMEG00000011150/ENSAMEG00000004713/ENSAMEG00000013843/ENSAMEG00000001421/ENSAMEG00000008785/ENSAMEG00000000109/ENSAMEG00000017063/ENSAMEG00000009566/ENSAMEG00000006307/ENSAMEG00000002043/ENSAMEG00000016233/ENSAMEG00000007626 | 17 |
| GO:0017056 | structural constituent of nuclear pore | 6.62E-10 | 9.38E-08 | 8.12E-08 | ENSAMEG00000012008/ENSAMEG00000019816/ENSAMEG00000000721/ENSAMEG00000015857/ENSAMEG00000005149/ENSAMEG00000016303/ENSAMEG00000010461/ENSAMEG00000016715/ENSAMEG00000009783/ENSAMEG00000009151/ENSAMEG00000009192/ENSAMEG00000014220 | 12 |
| GO:0051726 | regulation of cell cycle | 1.81E-09 | 2.32E-07 | 2.01E-07 | ENSAMEG00000017945/ENSAMEG00000006109/ENSAMEG00000002586/ENSAMEG00000001259/ENSAMEG00000009662/ENSAMEG00000011622/ENSAMEG00000008991/ENSAMEG00000009668/ENSAMEG00000019297/ENSAMEG00000012415/ENSAMEG00000012466/ENSAMEG00000005436/ENSAMEG00000017610/ENSAMEG00000017392/ENSAMEG00000005590/ENSAMEG00000003640/ENSAMEG00000001855/ENSAMEG00000016250/ENSAMEG00000008731/ENSAMEG00000016303/ENSAMEG00000013018/ENSAMEG00000004645/ENSAMEG00000003944/ENSAMEG00000003168/ENSAMEG00000001223/ENSAMEG00000007321 | 26 |
| GO:0030496 | midbody | 1.89E-09 | 2.32E-07 | 2.01E-07 | ENSAMEG00000015869/ENSAMEG00000013667/ENSAMEG00000009205/ENSAMEG00000017540/ENSAMEG00000001093/ENSAMEG00000016438/ENSAMEG00000003520/ENSAMEG00000012611/ENSAMEG00000001035/ENSAMEG00000001187/ENSAMEG00000014201/ENSAMEG00000002580/ENSAMEG00000001082/ENSAMEG00000014232/ENSAMEG00000016838/ENSAMEG00000013840/ENSAMEG00000004713/ENSAMEG00000001746/ENSAMEG00000004686/ENSAMEG00000016390/ENSAMEG00000002139/ENSAMEG00000005226/ENSAMEG00000016790/ENSAMEG00000002058/ENSAMEG00000003458/ENSAMEG00000014294/ENSAMEG00000009852/ENSAMEG00000017479/ENSAMEG00000008124/ENSAMEG00000000769 | 30 |
| GO:0051301 | cell division | 7.27E-09 | 8.37E-07 | 7.25E-07 | ENSAMEG00000004841/ENSAMEG00000012611/ENSAMEG00000014358/ENSAMEG00000000346/ENSAMEG00000007193/ENSAMEG00000008950/ENSAMEG00000007868/ENSAMEG00000003344/ENSAMEG00000012524/ENSAMEG00000000371/ENSAMEG00000003640/ENSAMEG00000000280/ENSAMEG00000017365/ENSAMEG00000005084/ENSAMEG00000017110/ENSAMEG00000000983/ENSAMEG00000016594/ENSAMEG00000011643 | 18 |
| GO:0000775 | chromosome, centromeric region | 1.39E-08 | 1.42E-06 | 1.23E-06 | ENSAMEG00000015869/ENSAMEG00000014095/ENSAMEG00000002112/ENSAMEG00000017537/ENSAMEG00000009491/ENSAMEG00000016113/ENSAMEG00000000913/ENSAMEG00000001082/ENSAMEG00000016838/ENSAMEG00000005435/ENSAMEG00000013510/ENSAMEG00000002148/ENSAMEG00000017438 | 13 |
| GO:0007052 | mitotic spindle organization | 1.39E-08 | 1.42E-06 | 1.23E-06 | ENSAMEG00000016924/ENSAMEG00000007168/ENSAMEG00000010069/ENSAMEG00000000013/ENSAMEG00000000953/ENSAMEG00000010875/ENSAMEG00000011337/ENSAMEG00000004449/ENSAMEG00000010298/ENSAMEG00000015472/ENSAMEG00000001250/ENSAMEG00000009136/ENSAMEG00000005084 | 13 |
| GO:0000790 | nuclear chromatin | 2.47E-08 | 2.24E-06 | 1.94E-06 | ENSAMEG00000012407/ENSAMEG00000003466/ENSAMEG00000000014/ENSAMEG00000013206/ENSAMEG00000011867/ENSAMEG00000008991/ENSAMEG00000002791/ENSAMEG00000015678/ENSAMEG00000003946/ENSAMEG00000004713/ENSAMEG00000011337/ENSAMEG00000017674/ENSAMEG00000017275/ENSAMEG00000018491/ENSAMEG00000005560/ENSAMEG00000015152/ENSAMEG00000003503/ENSAMEG00000013676/ENSAMEG00000014262/ENSAMEG00000000622/ENSAMEG00000007608/ENSAMEG00000010868/ENSAMEG00000002002/ENSAMEG00000015417/ENSAMEG00000010750/ENSAMEG00000016108/ENSAMEG00000017926/ENSAMEG00000011366/ENSAMEG00000010799/ENSAMEG00000017055 | 30 |
| GO:0000724 | double-strand break repair via homologous recombination | 2.54E-08 | 2.24E-06 | 1.94E-06 | ENSAMEG00000000171/ENSAMEG00000016965/ENSAMEG00000005645/ENSAMEG00000009390/ENSAMEG00000002666/ENSAMEG00000002533/ENSAMEG00000015678/ENSAMEG00000013902/ENSAMEG00000010293/ENSAMEG00000011132/ENSAMEG00000016796/ENSAMEG00000010605/ENSAMEG00000001323/ENSAMEG00000013304/ENSAMEG00000014287/ENSAMEG00000014294/ENSAMEG00000004324/ENSAMEG00000007706/ENSAMEG00000007626 | 19 |
| GO:0005814 | centriole | 2.55E-08 | 2.24E-06 | 1.94E-06 | ENSAMEG00000017945/ENSAMEG00000007989/ENSAMEG00000002675/ENSAMEG00000000013/ENSAMEG00000008931/ENSAMEG00000008648/ENSAMEG00000001549/ENSAMEG00000014232/ENSAMEG00000012848/ENSAMEG00000003208/ENSAMEG00000000293/ENSAMEG00000017216/ENSAMEG00000001860/ENSAMEG00000001250/ENSAMEG00000017365/ENSAMEG00000009136/ENSAMEG00000010387/ENSAMEG00000015487/ENSAMEG00000001007/ENSAMEG00000017728/ENSAMEG00000015126/ENSAMEG00000015890/ENSAMEG00000010566/ENSAMEG00000011958 | 24 |
| GO:0016607 | nuclear speck | 3.28E-08 | 2.71E-06 | 2.35E-06 | ENSAMEG00000007247/ENSAMEG00000014825/ENSAMEG00000016520/ENSAMEG00000016777/ENSAMEG00000002220/ENSAMEG00000008321/ENSAMEG00000012055/ENSAMEG00000003215/ENSAMEG00000009372/ENSAMEG00000003804/ENSAMEG00000005341/ENSAMEG00000017518/ENSAMEG00000006089/ENSAMEG00000001807/ENSAMEG00000012914/ENSAMEG00000018404/ENSAMEG00000001352/ENSAMEG00000001685/ENSAMEG00000006373/ENSAMEG00000017587/ENSAMEG00000017412/ENSAMEG00000003503/ENSAMEG00000018018/ENSAMEG00000015502/ENSAMEG00000010877/ENSAMEG00000007576/ENSAMEG00000009101/ENSAMEG00000010075/ENSAMEG00000016270/ENSAMEG00000007789/ENSAMEG00000003944/ENSAMEG00000018390/ENSAMEG00000002912/ENSAMEG00000011540/ENSAMEG00000000379/ENSAMEG00000002161/ENSAMEG00000015431/ENSAMEG00000003904/ENSAMEG00000002777/ENSAMEG00000006883/ENSAMEG00000018179/ENSAMEG00000009201/ENSAMEG00000010542/ENSAMEG00000000259/ENSAMEG00000013379/ENSAMEG00000013117/ENSAMEG00000018326/ENSAMEG00000009522/ENSAMEG00000011668/ENSAMEG00000002802/ENSAMEG00000017689 | 51 |
| GO:0031297 | replication fork processing | 3.39E-08 | 2.71E-06 | 2.35E-06 | ENSAMEG00000005645/ENSAMEG00000002666/ENSAMEG00000002533/ENSAMEG00000002114/ENSAMEG00000004713/ENSAMEG00000013843/ENSAMEG00000009089/ENSAMEG00000001323/ENSAMEG00000009321/ENSAMEG00000002043/ENSAMEG00000016419 | 11 |
| GO:0000070 | mitotic sister chromatid segregation | 4.44E-08 | 3.35E-06 | 2.90E-06 | ENSAMEG00000001187/ENSAMEG00000005342/ENSAMEG00000017084/ENSAMEG00000014232/ENSAMEG00000016838/ENSAMEG00000012346/ENSAMEG00000016796/ENSAMEG00000001352/ENSAMEG00000009521 | 9 |
| GO:0000793 | condensed chromosome | 4.59E-08 | 3.35E-06 | 2.90E-06 | ENSAMEG00000007989/ENSAMEG00000005936/ENSAMEG00000014358/ENSAMEG00000000171/ENSAMEG00000016051/ENSAMEG00000008461/ENSAMEG00000017438/ENSAMEG00000014262/ENSAMEG00000002281/ENSAMEG00000017905/ENSAMEG00000003168/ENSAMEG00000011177 | 12 |
| GO:0000281 | mitotic cytokinesis | 4.76E-08 | 3.35E-06 | 2.90E-06 | ENSAMEG00000015972/ENSAMEG00000002112/ENSAMEG00000009205/ENSAMEG00000016762/ENSAMEG00000001093/ENSAMEG00000016438/ENSAMEG00000001035/ENSAMEG00000014201/ENSAMEG00000009390/ENSAMEG00000014232/ENSAMEG00000016838/ENSAMEG00000013840/ENSAMEG00000002139/ENSAMEG00000011827/ENSAMEG00000010298/ENSAMEG00000014294 | 16 |
| GO:0051276 | chromosome organization | 4.91E-08 | 3.35E-06 | 2.90E-06 | ENSAMEG00000017537/ENSAMEG00000009491/ENSAMEG00000005936/ENSAMEG00000006653/ENSAMEG00000009390/ENSAMEG00000002220/ENSAMEG00000001082/ENSAMEG00000013902/ENSAMEG00000005053/ENSAMEG00000010022/ENSAMEG00000002560/ENSAMEG00000015675/ENSAMEG00000007626 | 13 |
| GO:0090305 | nucleic acid phosphodiester bond hydrolysis | 7.73E-08 | 5.09E-06 | 4.41E-06 | ENSAMEG00000013934/ENSAMEG00000011867/ENSAMEG00000018028/ENSAMEG00000005534/ENSAMEG00000010455/ENSAMEG00000002666/ENSAMEG00000002114/ENSAMEG00000003270/ENSAMEG00000003608/ENSAMEG00000013111/ENSAMEG00000019971/ENSAMEG00000012538/ENSAMEG00000014205/ENSAMEG00000016002/ENSAMEG00000005278/ENSAMEG00000012436/ENSAMEG00000017693/ENSAMEG00000016594/ENSAMEG00000001645/ENSAMEG00000007626/ENSAMEG00000016419 | 21 |
| GO:0003697 | single-stranded DNA binding | 8.97E-08 | 5.70E-06 | 4.93E-06 | ENSAMEG00000003715/ENSAMEG00000016965/ENSAMEG00000013505/ENSAMEG00000009390/ENSAMEG00000015678/ENSAMEG00000011150/ENSAMEG00000004713/ENSAMEG00000013843/ENSAMEG00000012375/ENSAMEG00000011132/ENSAMEG00000014262/ENSAMEG00000004324/ENSAMEG00000009792/ENSAMEG00000001964/ENSAMEG00000001905/ENSAMEG00000007706/ENSAMEG00000002560/ENSAMEG00000017905/ENSAMEG00000002043/ENSAMEG00000009674 | 20 |
| GO:0051382 | kinetochore assembly | 1.15E-07 | 7.06E-06 | 6.11E-06 | ENSAMEG00000002112/ENSAMEG00000017537/ENSAMEG00000009491/ENSAMEG00000013933/ENSAMEG00000011135/ENSAMEG00000003458/ENSAMEG00000003155/ENSAMEG00000000622 | 8 |
| GO:0019901 | protein kinase binding | 2.62E-07 | 1.55E-05 | 1.35E-05 | ENSAMEG00000013667/ENSAMEG00000014099/ENSAMEG00000013678/ENSAMEG00000006109/ENSAMEG00000001093/ENSAMEG00000016438/ENSAMEG00000011957/ENSAMEG00000007168/ENSAMEG00000012611/ENSAMEG00000010721/ENSAMEG00000004869/ENSAMEG00000013455/ENSAMEG00000016520/ENSAMEG00000000646/ENSAMEG00000009668/ENSAMEG00000001398/ENSAMEG00000001549/ENSAMEG00000014232/ENSAMEG00000012055/ENSAMEG00000016390/ENSAMEG00000001260/ENSAMEG00000014202/ENSAMEG00000011370/ENSAMEG00000017478/ENSAMEG00000009661/ENSAMEG00000009575/ENSAMEG00000001233/ENSAMEG00000005246/ENSAMEG00000008484/ENSAMEG00000015932/ENSAMEG00000015158/ENSAMEG00000017365/ENSAMEG00000002927/ENSAMEG00000008832/ENSAMEG00000008731/ENSAMEG00000013018/ENSAMEG00000005155/ENSAMEG00000003944/ENSAMEG00000006798/ENSAMEG00000018388/ENSAMEG00000008158/ENSAMEG00000005096/ENSAMEG00000019817/ENSAMEG00000005077/ENSAMEG00000008223/ENSAMEG00000005378/ENSAMEG00000002515/ENSAMEG00000017753/ENSAMEG00000010769/ENSAMEG00000013710/ENSAMEG00000016139/ENSAMEG00000000769 | 52 |
| GO:0007099 | centriole replication | 3.44E-07 | 1.94E-05 | 1.68E-05 | ENSAMEG00000002675/ENSAMEG00000008931/ENSAMEG00000000953/ENSAMEG00000001549/ENSAMEG00000001233/ENSAMEG00000017365/ENSAMEG00000008333/ENSAMEG00000003168/ENSAMEG00000011958 | 9 |
| GO:0006302 | double-strand break repair | 3.47E-07 | 1.94E-05 | 1.68E-05 | ENSAMEG00000000137/ENSAMEG00000000171/ENSAMEG00000014870/ENSAMEG00000009390/ENSAMEG00000008267/ENSAMEG00000013902/ENSAMEG00000016796/ENSAMEG00000008484/ENSAMEG00000010022/ENSAMEG00000009586/ENSAMEG00000007365/ENSAMEG00000001905/ENSAMEG00000004887/ENSAMEG00000002562/ENSAMEG00000007626/ENSAMEG00000011164 | 16 |
| GO:0005876 | spindle microtubule | 4.94E-07 | 2.68E-05 | 2.32E-05 | ENSAMEG00000004841/ENSAMEG00000014099/ENSAMEG00000016762/ENSAMEG00000003520/ENSAMEG00000007168/ENSAMEG00000014232/ENSAMEG00000014641/ENSAMEG00000004686/ENSAMEG00000000280/ENSAMEG00000005084/ENSAMEG00000011643/ENSAMEG00000010861 | 12 |
| GO:0018024 | histone-lysine N-methyltransferase activity | 8.20E-07 | 4.27E-05 | 3.70E-05 | ENSAMEG00000002791/ENSAMEG00000014221/ENSAMEG00000013308/ENSAMEG00000005436/ENSAMEG00000012595/ENSAMEG00000007540/ENSAMEG00000000319/ENSAMEG00000001160/ENSAMEG00000012781/ENSAMEG00000011260/ENSAMEG00000006766/ENSAMEG00000006918 | 12 |
| GO:0008017 | microtubule binding | 8.50E-07 | 4.27E-05 | 3.70E-05 | ENSAMEG00000014095/ENSAMEG00000013667/ENSAMEG00000014099/ENSAMEG00000009205/ENSAMEG00000016762/ENSAMEG00000001458/ENSAMEG00000012038/ENSAMEG00000001093/ENSAMEG00000016438/ENSAMEG00000011957/ENSAMEG00000012611/ENSAMEG00000018442/ENSAMEG00000014201/ENSAMEG00000014825/ENSAMEG00000018397/ENSAMEG00000008648/ENSAMEG00000014549/ENSAMEG00000014232/ENSAMEG00000004686/ENSAMEG00000012848/ENSAMEG00000004598/ENSAMEG00000000293/ENSAMEG00000012351/ENSAMEG00000000280/ENSAMEG00000016790/ENSAMEG00000001233/ENSAMEG00000005084/ENSAMEG00000015215/ENSAMEG00000017110/ENSAMEG00000002382/ENSAMEG00000017728/ENSAMEG00000003586/ENSAMEG00000011643/ENSAMEG00000010861/ENSAMEG00000000769 | 35 |
| GO:0042393 | histone binding | 8.57E-07 | 4.27E-05 | 3.70E-05 | ENSAMEG00000014998/ENSAMEG00000013206/ENSAMEG00000016113/ENSAMEG00000014358/ENSAMEG00000005645/ENSAMEG00000011337/ENSAMEG00000009661/ENSAMEG00000002954/ENSAMEG00000001685/ENSAMEG00000004696/ENSAMEG00000013676/ENSAMEG00000001319/ENSAMEG00000007365/ENSAMEG00000010868/ENSAMEG00000013061/ENSAMEG00000002161/ENSAMEG00000006307/ENSAMEG00000017390/ENSAMEG00000011177/ENSAMEG00000002111/ENSAMEG00000004446 | 21 |
| GO:0005819 | spindle | 9.76E-07 | 4.73E-05 | 4.10E-05 | ENSAMEG00000015869/ENSAMEG00000013667/ENSAMEG00000014099/ENSAMEG00000013678/ENSAMEG00000016762/ENSAMEG00000016438/ENSAMEG00000014201/ENSAMEG00000014232/ENSAMEG00000016838/ENSAMEG00000001746/ENSAMEG00000004686/ENSAMEG00000002139/ENSAMEG00000001260/ENSAMEG00000009369/ENSAMEG00000003640/ENSAMEG00000017110 | 16 |
| GO:0031965 | nuclear membrane | 1.27E-06 | 6.02E-05 | 5.22E-05 | ENSAMEG00000015778/ENSAMEG00000012331/ENSAMEG00000010051/ENSAMEG00000013800/ENSAMEG00000004354/ENSAMEG00000007412/ENSAMEG00000011337/ENSAMEG00000008785/ENSAMEG00000012008/ENSAMEG00000015537/ENSAMEG00000019816/ENSAMEG00000014068/ENSAMEG00000012140/ENSAMEG00000000721/ENSAMEG00000005548/ENSAMEG00000013202/ENSAMEG00000005634/ENSAMEG00000008461/ENSAMEG00000001319/ENSAMEG00000013719/ENSAMEG00000002689/ENSAMEG00000013767/ENSAMEG00000016715/ENSAMEG00000014031/ENSAMEG00000009783/ENSAMEG00000013773/ENSAMEG00000009151/ENSAMEG00000009192/ENSAMEG00000006513/ENSAMEG00000016755/ENSAMEG00000006185 | 31 |
| GO:0072686 | mitotic spindle | 1.57E-06 | 7.24E-05 | 6.27E-05 | ENSAMEG00000014099/ENSAMEG00000013678/ENSAMEG00000001093/ENSAMEG00000010767/ENSAMEG00000016924/ENSAMEG00000003520/ENSAMEG00000007168/ENSAMEG00000001035/ENSAMEG00000014201/ENSAMEG00000014825/ENSAMEG00000017084/ENSAMEG00000000492/ENSAMEG00000019816/ENSAMEG00000017390/ENSAMEG00000000410/ENSAMEG00000010861/ENSAMEG00000017479 | 17 |
| GO:0016604 | nuclear body | 1.76E-06 | 7.91E-05 | 6.85E-05 | ENSAMEG00000017537/ENSAMEG00000013934/ENSAMEG00000016983/ENSAMEG00000005342/ENSAMEG00000010307/ENSAMEG00000005645/ENSAMEG00000016051/ENSAMEG00000016838/ENSAMEG00000009547/ENSAMEG00000005435/ENSAMEG00000003270/ENSAMEG00000009168/ENSAMEG00000001673/ENSAMEG00000012524/ENSAMEG00000000371/ENSAMEG00000009089/ENSAMEG00000012375/ENSAMEG00000011132/ENSAMEG00000000352/ENSAMEG00000016796/ENSAMEG00000013188/ENSAMEG00000017889/ENSAMEG00000011495/ENSAMEG00000011355/ENSAMEG00000003458/ENSAMEG00000016250/ENSAMEG00000008461/ENSAMEG00000001226/ENSAMEG00000015826/ENSAMEG00000014449/ENSAMEG00000011868/ENSAMEG00000007365/ENSAMEG00000015417/ENSAMEG00000004887/ENSAMEG00000005178/ENSAMEG00000005490/ENSAMEG00000011177/ENSAMEG00000012634/ENSAMEG00000007476/ENSAMEG00000003790/ENSAMEG00000008828/ENSAMEG00000010799/ENSAMEG00000017492 | 43 |
| GO:0000777 | condensed chromosome kinetochore | 2.59E-06 | 1.14E-04 | 9.85E-05 | ENSAMEG00000009532/ENSAMEG00000016983/ENSAMEG00000016113/ENSAMEG00000010069/ENSAMEG00000010875/ENSAMEG00000012524/ENSAMEG00000001352/ENSAMEG00000009151 | 8 |
| GO:0036297 | interstrand cross-link repair | 3.29E-06 | 1.30E-04 | 1.12E-04 | ENSAMEG00000015678/ENSAMEG00000000684/ENSAMEG00000003270/ENSAMEG00000006335/ENSAMEG00000015491/ENSAMEG00000001323/ENSAMEG00000015524/ENSAMEG00000015826/ENSAMEG00000011534 | 9 |
| GO:0000307 | cyclin-dependent protein kinase holoenzyme complex | 3.31E-06 | 1.30E-04 | 1.12E-04 | ENSAMEG00000006109/ENSAMEG00000003520/ENSAMEG00000009668/ENSAMEG00000009089/ENSAMEG00000013018/ENSAMEG00000017811/ENSAMEG00000003168 | 7 |
| GO:0006779 | porphyrin-containing compound biosynthetic process | 3.31E-06 | 1.30E-04 | 1.12E-04 | ENSAMEG00000003988/ENSAMEG00000015511/ENSAMEG00000014813/ENSAMEG00000002427/ENSAMEG00000015509/ENSAMEG00000013467/ENSAMEG00000002188 | 7 |
| GO:0007064 | mitotic sister chromatid cohesion | 3.31E-06 | 1.30E-04 | 1.12E-04 | ENSAMEG00000006653/ENSAMEG00000001637/ENSAMEG00000009369/ENSAMEG00000002148/ENSAMEG00000005053/ENSAMEG00000011868/ENSAMEG00000000622 | 7 |
| GO:0051298 | centrosome duplication | 3.31E-06 | 1.30E-04 | 1.12E-04 | ENSAMEG00000000013/ENSAMEG00000008931/ENSAMEG00000009390/ENSAMEG00000001549/ENSAMEG00000017740/ENSAMEG00000017365/ENSAMEG00000011958 | 7 |
| GO:0032991 | protein-containing complex | 3.63E-06 | 1.39E-04 | 1.21E-04 | ENSAMEG00000003546/ENSAMEG00000007989/ENSAMEG00000014099/ENSAMEG00000000014/ENSAMEG00000013206/ENSAMEG00000001187/ENSAMEG00000000171/ENSAMEG00000008991/ENSAMEG00000009390/ENSAMEG00000001082/ENSAMEG00000000646/ENSAMEG00000011446/ENSAMEG00000008899/ENSAMEG00000015678/ENSAMEG00000016838/ENSAMEG00000013902/ENSAMEG00000006979/ENSAMEG00000011337/ENSAMEG00000008979/ENSAMEG00000005436/ENSAMEG00000013940/ENSAMEG00000004449/ENSAMEG00000003208/ENSAMEG00000019971/ENSAMEG00000008051/ENSAMEG00000017674/ENSAMEG00000000293/ENSAMEG00000006467/ENSAMEG00000011015/ENSAMEG00000005246/ENSAMEG00000005560/ENSAMEG00000012527/ENSAMEG00000018450/ENSAMEG00000016250/ENSAMEG00000013062/ENSAMEG00000013676/ENSAMEG00000016129/ENSAMEG00000010726/ENSAMEG00000014262/ENSAMEG00000004324/ENSAMEG00000004214/ENSAMEG00000009792/ENSAMEG00000007608/ENSAMEG00000016518/ENSAMEG00000003364/ENSAMEG00000002002/ENSAMEG00000016411/ENSAMEG00000008158/ENSAMEG00000014506/ENSAMEG00000006307/ENSAMEG00000004665/ENSAMEG00000017693/ENSAMEG00000003720/ENSAMEG00000006521/ENSAMEG00000010861/ENSAMEG00000011366/ENSAMEG00000000707/ENSAMEG00000011958/ENSAMEG00000005584/ENSAMEG00000008705/ENSAMEG00000016649/ENSAMEG00000016139/ENSAMEG00000017055/ENSAMEG00000009724 | 64 |
| GO:0004386 | helicase activity | 3.74E-06 | 1.41E-04 | 1.22E-04 | ENSAMEG00000014998/ENSAMEG00000001634/ENSAMEG00000005841/ENSAMEG00000008540/ENSAMEG00000005460/ENSAMEG00000011150/ENSAMEG00000004713/ENSAMEG00000013843/ENSAMEG00000001421/ENSAMEG00000008785/ENSAMEG00000007401/ENSAMEG00000000475/ENSAMEG00000009566/ENSAMEG00000006307/ENSAMEG00000015543/ENSAMEG00000016233/ENSAMEG00000011474/ENSAMEG00000002227 | 18 |
| GO:0005643 | nuclear pore | 4.80E-06 | 1.74E-04 | 1.50E-04 | ENSAMEG00000003555/ENSAMEG00000012008/ENSAMEG00000015537/ENSAMEG00000019816/ENSAMEG00000000721/ENSAMEG00000005149/ENSAMEG00000010461/ENSAMEG00000016715/ENSAMEG00000009783/ENSAMEG00000009151/ENSAMEG00000009192/ENSAMEG00000014220 | 12 |
| GO:0006310 | DNA recombination | 4.80E-06 | 1.74E-04 | 1.50E-04 | ENSAMEG00000013934/ENSAMEG00000000171/ENSAMEG00000016965/ENSAMEG00000015091/ENSAMEG00000005460/ENSAMEG00000013843/ENSAMEG00000011132/ENSAMEG00000000475/ENSAMEG00000004324/ENSAMEG00000007706/ENSAMEG00000009566/ENSAMEG00000016233 | 12 |
| GO:0007051 | spindle organization | 5.59E-06 | 1.98E-04 | 1.72E-04 | ENSAMEG00000014099/ENSAMEG00000017540/ENSAMEG00000007168/ENSAMEG00000017084/ENSAMEG00000002580/ENSAMEG00000006979/ENSAMEG00000010293/ENSAMEG00000008333 | 8 |
| GO:0003684 | damaged DNA binding | 6.16E-06 | 2.14E-04 | 1.85E-04 | ENSAMEG00000012174/ENSAMEG00000003715/ENSAMEG00000000171/ENSAMEG00000016965/ENSAMEG00000010293/ENSAMEG00000013940/ENSAMEG00000009089/ENSAMEG00000011132/ENSAMEG00000010937/ENSAMEG00000012527/ENSAMEG00000014262/ENSAMEG00000016518/ENSAMEG00000007706/ENSAMEG00000010799 | 14 |
| GO:0000082 | G1/S transition of mitotic cell cycle | 6.82E-06 | 2.33E-04 | 2.02E-04 | ENSAMEG00000014672/ENSAMEG00000013454/ENSAMEG00000009668/ENSAMEG00000005514/ENSAMEG00000011337/ENSAMEG00000011370/ENSAMEG00000017478/ENSAMEG00000005590/ENSAMEG00000003640/ENSAMEG00000002927/ENSAMEG00000003168/ENSAMEG00000006185/ENSAMEG00000010769 | 13 |
| GO:0030218 | erythrocyte differentiation | 7.09E-06 | 2.37E-04 | 2.06E-04 | ENSAMEG00000016195/ENSAMEG00000012588/ENSAMEG00000012407/ENSAMEG00000000014/ENSAMEG00000009186/ENSAMEG00000000508/ENSAMEG00000008051/ENSAMEG00000017518/ENSAMEG00000014449/ENSAMEG00000007576/ENSAMEG00000011594/ENSAMEG00000001394 | 12 |
| GO:0000785 | chromatin | 7.36E-06 | 2.42E-04 | 2.10E-04 | ENSAMEG00000014998/ENSAMEG00000013206/ENSAMEG00000001061/ENSAMEG00000000137/ENSAMEG00000016777/ENSAMEG00000014232/ENSAMEG00000000792/ENSAMEG00000001064/ENSAMEG00000011337/ENSAMEG00000009089/ENSAMEG00000011132/ENSAMEG00000002148/ENSAMEG00000010691/ENSAMEG00000011868/ENSAMEG00000007608/ENSAMEG00000002231/ENSAMEG00000002043/ENSAMEG00000015675/ENSAMEG00000011474 | 19 |
| GO:0007076 | mitotic chromosome condensation | 8.43E-06 | 2.72E-04 | 2.36E-04 | ENSAMEG00000000194/ENSAMEG00000005936/ENSAMEG00000014358/ENSAMEG00000007193/ENSAMEG00000008950/ENSAMEG00000002220/ENSAMEG00000002281 | 7 |
| GO:0005874 | microtubule | 1.00E-05 | 3.12E-04 | 2.70E-04 | ENSAMEG00000014095/ENSAMEG00000014099/ENSAMEG00000013678/ENSAMEG00000016762/ENSAMEG00000001458/ENSAMEG00000017540/ENSAMEG00000016438/ENSAMEG00000012611/ENSAMEG00000012145/ENSAMEG00000018442/ENSAMEG00000014201/ENSAMEG00000014825/ENSAMEG00000018397/ENSAMEG00000014549/ENSAMEG00000012472/ENSAMEG00000013184/ENSAMEG00000006872/ENSAMEG00000004598/ENSAMEG00000016734/ENSAMEG00000001233/ENSAMEG00000016380/ENSAMEG00000017110/ENSAMEG00000001007/ENSAMEG00000011431/ENSAMEG00000016735/ENSAMEG00000017728/ENSAMEG00000007112/ENSAMEG00000011055 | 28 |
| GO:0007098 | centrosome cycle | 1.02E-05 | 3.12E-04 | 2.70E-04 | ENSAMEG00000007168/ENSAMEG00000000171/ENSAMEG00000014232/ENSAMEG00000006979/ENSAMEG00000000503/ENSAMEG00000019816/ENSAMEG00000001233/ENSAMEG00000006467/ENSAMEG00000013304/ENSAMEG00000012644/ENSAMEG00000002777 | 11 |
| GO:0010212 | response to ionizing radiation | 1.02E-05 | 3.12E-04 | 2.70E-04 | ENSAMEG00000000171/ENSAMEG00000013104/ENSAMEG00000013902/ENSAMEG00000000352/ENSAMEG00000001323/ENSAMEG00000001238/ENSAMEG00000003233/ENSAMEG00000007365/ENSAMEG00000004887/ENSAMEG00000002562/ENSAMEG00000011164 | 11 |
| GO:0004843 | thiol-dependent ubiquitin-specific protease activity | 1.21E-05 | 3.67E-04 | 3.18E-04 | ENSAMEG00000002210/ENSAMEG00000003616/ENSAMEG00000014832/ENSAMEG00000008169/ENSAMEG00000002927/ENSAMEG00000009899/ENSAMEG00000010726/ENSAMEG00000012591/ENSAMEG00000006687/ENSAMEG00000012420/ENSAMEG00000012970/ENSAMEG00000013061/ENSAMEG00000013920/ENSAMEG00000011298/ENSAMEG00000006853/ENSAMEG00000008960/ENSAMEG00000012785/ENSAMEG00000004155 | 18 |
| GO:0005815 | microtubule organizing center | 1.74E-05 | 5.17E-04 | 4.48E-04 | ENSAMEG00000007001/ENSAMEG00000013678/ENSAMEG00000016924/ENSAMEG00000007168/ENSAMEG00000008931/ENSAMEG00000000953/ENSAMEG00000001549/ENSAMEG00000013840/ENSAMEG00000008494/ENSAMEG00000010512/ENSAMEG00000001233/ENSAMEG00000012527/ENSAMEG00000001250/ENSAMEG00000017110/ENSAMEG00000017319/ENSAMEG00000002382/ENSAMEG00000001092/ENSAMEG00000001007/ENSAMEG00000002777/ENSAMEG00000000983/ENSAMEG00000013766/ENSAMEG00000003720/ENSAMEG00000011958 | 23 |
| GO:0000987 | proximal promoter sequence-specific DNA binding | 2.14E-05 | 6.27E-04 | 5.43E-04 | ENSAMEG00000012407/ENSAMEG00000017950/ENSAMEG00000009662/ENSAMEG00000007247/ENSAMEG00000008991/ENSAMEG00000000747/ENSAMEG00000003640/ENSAMEG00000001186/ENSAMEG00000010799/ENSAMEG00000017055 | 10 |
| GO:0005667 | transcription factor complex | 2.33E-05 | 6.72E-04 | 5.82E-04 | ENSAMEG00000012588/ENSAMEG00000017950/ENSAMEG00000000014/ENSAMEG00000009662/ENSAMEG00000007247/ENSAMEG00000008991/ENSAMEG00000004257/ENSAMEG00000012415/ENSAMEG00000012466/ENSAMEG00000013940/ENSAMEG00000017674/ENSAMEG00000017275/ENSAMEG00000003640/ENSAMEG00000007383/ENSAMEG00000015152/ENSAMEG00000003503/ENSAMEG00000015779/ENSAMEG00000009792/ENSAMEG00000008615/ENSAMEG00000007608/ENSAMEG00000004645/ENSAMEG00000008231/ENSAMEG00000006241/ENSAMEG00000003168/ENSAMEG00000011164 | 25 |
| GO:0004674 | protein serine/threonine kinase activity | 2.78E-05 | 7.89E-04 | 6.83E-04 | ENSAMEG00000015972/ENSAMEG00000003520/ENSAMEG00000007168/ENSAMEG00000002675/ENSAMEG00000002580/ENSAMEG00000013744/ENSAMEG00000014232/ENSAMEG00000010058/ENSAMEG00000000135/ENSAMEG00000015426/ENSAMEG00000004345/ENSAMEG00000013726/ENSAMEG00000014202/ENSAMEG00000017518/ENSAMEG00000003841/ENSAMEG00000012351/ENSAMEG00000008945/ENSAMEG00000017889/ENSAMEG00000001855/ENSAMEG00000001572/ENSAMEG00000008484/ENSAMEG00000018172/ENSAMEG00000000501/ENSAMEG00000004552/ENSAMEG00000016102/ENSAMEG00000003233/ENSAMEG00000014205/ENSAMEG00000018110/ENSAMEG00000017811/ENSAMEG00000008397/ENSAMEG00000003944/ENSAMEG00000003362/ENSAMEG00000013754/ENSAMEG00000016638/ENSAMEG00000015683/ENSAMEG00000003168/ENSAMEG00000000822/ENSAMEG00000009674/ENSAMEG00000018210/ENSAMEG00000018163/ENSAMEG00000017753/ENSAMEG00000015916/ENSAMEG00000005584/ENSAMEG00000001223/ENSAMEG00000009856 | 45 |
| GO:0017053 | transcriptional repressor complex | 2.84E-05 | 7.92E-04 | 6.86E-04 | ENSAMEG00000012588/ENSAMEG00000000402/ENSAMEG00000002934/ENSAMEG00000009592/ENSAMEG00000007608/ENSAMEG00000014535/ENSAMEG00000004496/ENSAMEG00000008231/ENSAMEG00000015417/ENSAMEG00000018211/ENSAMEG00000002532/ENSAMEG00000000707 | 12 |
| GO:0006913 | nucleocytoplasmic transport | 2.89E-05 | 7.95E-04 | 6.89E-04 | ENSAMEG00000012008/ENSAMEG00000019816/ENSAMEG00000000721/ENSAMEG00000015857/ENSAMEG00000010461/ENSAMEG00000016715/ENSAMEG00000009783/ENSAMEG00000009192/ENSAMEG00000014220 | 9 |
| GO:0007094 | mitotic spindle assembly checkpoint | 3.75E-05 | 1.02E-03 | 8.81E-04 | ENSAMEG00000009532/ENSAMEG00000012771/ENSAMEG00000002580/ENSAMEG00000008267/ENSAMEG00000014232/ENSAMEG00000013777/ENSAMEG00000017203 | 7 |
| GO:0016579 | protein deubiquitination | 3.82E-05 | 1.02E-03 | 8.84E-04 | ENSAMEG00000002210/ENSAMEG00000003616/ENSAMEG00000014832/ENSAMEG00000008169/ENSAMEG00000002927/ENSAMEG00000009899/ENSAMEG00000010726/ENSAMEG00000012591/ENSAMEG00000006687/ENSAMEG00000012420/ENSAMEG00000012970/ENSAMEG00000013061/ENSAMEG00000013920/ENSAMEG00000011298/ENSAMEG00000006853/ENSAMEG00000008960/ENSAMEG00000012785 | 17 |
| GO:0007080 | mitotic metaphase plate congression | 4.72E-05 | 1.24E-03 | 1.07E-03 | ENSAMEG00000014095/ENSAMEG00000001458/ENSAMEG00000012611/ENSAMEG00000014825/ENSAMEG00000001082/ENSAMEG00000014549/ENSAMEG00000013777/ENSAMEG00000004686/ENSAMEG00000019816/ENSAMEG00000016483 | 10 |
| GO:0005657 | replication fork | 5.95E-05 | 1.52E-03 | 1.32E-03 | ENSAMEG00000003466/ENSAMEG00000011823/ENSAMEG00000001064/ENSAMEG00000009089/ENSAMEG00000012375/ENSAMEG00000013304/ENSAMEG00000007626/ENSAMEG00000014866 | 8 |
| GO:0006275 | regulation of DNA replication | 5.95E-05 | 1.52E-03 | 1.32E-03 | ENSAMEG00000006109/ENSAMEG00000000137/ENSAMEG00000001260/ENSAMEG00000009089/ENSAMEG00000002148/ENSAMEG00000002927/ENSAMEG00000015675/ENSAMEG00000010211 | 8 |
| GO:0003774 | motor activity | 6.49E-05 | 1.64E-03 | 1.42E-03 | ENSAMEG00000014095/ENSAMEG00000014099/ENSAMEG00000009205/ENSAMEG00000001458/ENSAMEG00000012038/ENSAMEG00000016438/ENSAMEG00000012611/ENSAMEG00000014201/ENSAMEG00000014825/ENSAMEG00000018397/ENSAMEG00000014549/ENSAMEG00000004303/ENSAMEG00000002139/ENSAMEG00000004598/ENSAMEG00000000293/ENSAMEG00000016790/ENSAMEG00000004966/ENSAMEG00000017728/ENSAMEG00000016558/ENSAMEG00000017987 | 20 |
| GO:0032467 | positive regulation of cytokinesis | 6.86E-05 | 1.67E-03 | 1.44E-03 | ENSAMEG00000015972/ENSAMEG00000001093/ENSAMEG00000012611/ENSAMEG00000010721/ENSAMEG00000001035/ENSAMEG00000014201/ENSAMEG00000002580/ENSAMEG00000007868/ENSAMEG00000016790 | 9 |
| GO:0071897 | DNA biosynthetic process | 6.86E-05 | 1.67E-03 | 1.44E-03 | ENSAMEG00000014870/ENSAMEG00000015091/ENSAMEG00000014758/ENSAMEG00000009547/ENSAMEG00000017155/ENSAMEG00000013111/ENSAMEG00000010292/ENSAMEG00000013839/ENSAMEG00000017565 | 9 |
| GO:0051568 | histone H3-K4 methylation | 6.95E-05 | 1.67E-03 | 1.44E-03 | ENSAMEG00000013308/ENSAMEG00000001238/ENSAMEG00000011076/ENSAMEG00000007540/ENSAMEG00000000319/ENSAMEG00000000259/ENSAMEG00000006918 | 7 |
| GO:0007018 | microtubule-based movement | 6.97E-05 | 1.67E-03 | 1.44E-03 | ENSAMEG00000014095/ENSAMEG00000014099/ENSAMEG00000009205/ENSAMEG00000001458/ENSAMEG00000012038/ENSAMEG00000016438/ENSAMEG00000012611/ENSAMEG00000014201/ENSAMEG00000014825/ENSAMEG00000018397/ENSAMEG00000014549/ENSAMEG00000004598/ENSAMEG00000012392/ENSAMEG00000000293/ENSAMEG00000016790/ENSAMEG00000015805/ENSAMEG00000017728 | 17 |
| GO:0005694 | chromosome | 7.16E-05 | 1.67E-03 | 1.45E-03 | ENSAMEG00000002112/ENSAMEG00000017537/ENSAMEG00000005936/ENSAMEG00000000137/ENSAMEG00000000346/ENSAMEG00000000171/ENSAMEG00000002220/ENSAMEG00000004884/ENSAMEG00000012595/ENSAMEG00000019961/ENSAMEG00000018491/ENSAMEG00000000352/ENSAMEG00000012914/ENSAMEG00000005053/ENSAMEG00000018575/ENSAMEG00000018498/ENSAMEG00000010022/ENSAMEG00000001226/ENSAMEG00000018579/ENSAMEG00000018517/ENSAMEG00000002912/ENSAMEG00000001160/ENSAMEG00000017905/ENSAMEG00000015675/ENSAMEG00000012781/ENSAMEG00000009674/ENSAMEG00000000959 | 27 |
| GO:0005200 | structural constituent of cytoskeleton | 7.27E-05 | 1.67E-03 | 1.45E-03 | ENSAMEG00000003546/ENSAMEG00000012145/ENSAMEG00000012472/ENSAMEG00000013184/ENSAMEG00000006872/ENSAMEG00000016734/ENSAMEG00000016380/ENSAMEG00000015932/ENSAMEG00000014449/ENSAMEG00000011431/ENSAMEG00000016735/ENSAMEG00000009574/ENSAMEG00000007112 | 13 |
| GO:0035064 | methylated histone binding | 7.27E-05 | 1.67E-03 | 1.45E-03 | ENSAMEG00000003466/ENSAMEG00000007193/ENSAMEG00000008321/ENSAMEG00000013286/ENSAMEG00000012914/ENSAMEG00000001685/ENSAMEG00000014644/ENSAMEG00000006307/ENSAMEG00000005490/ENSAMEG00000008054/ENSAMEG00000006185/ENSAMEG00000000707/ENSAMEG00000004446 | 13 |
| GO:0003777 | microtubule motor activity | 8.44E-05 | 1.92E-03 | 1.66E-03 | ENSAMEG00000014095/ENSAMEG00000014099/ENSAMEG00000009205/ENSAMEG00000001458/ENSAMEG00000012038/ENSAMEG00000016438/ENSAMEG00000012611/ENSAMEG00000014201/ENSAMEG00000014825/ENSAMEG00000018397/ENSAMEG00000014549/ENSAMEG00000004598/ENSAMEG00000012392/ENSAMEG00000000293/ENSAMEG00000016790/ENSAMEG00000015805/ENSAMEG00000017728 | 17 |
| GO:0000079 | regulation of cyclin-dependent protein serine/threonine kinase activity | 9.50E-05 | 2.11E-03 | 1.83E-03 | ENSAMEG00000009668/ENSAMEG00000012055/ENSAMEG00000013843/ENSAMEG00000003344/ENSAMEG00000011370/ENSAMEG00000017478/ENSAMEG00000005590/ENSAMEG00000002515 | 8 |
| GO:0035097 | histone methyltransferase complex | 9.50E-05 | 2.11E-03 | 1.83E-03 | ENSAMEG00000013308/ENSAMEG00000001238/ENSAMEG00000011076/ENSAMEG00000000319/ENSAMEG00000000259/ENSAMEG00000007355/ENSAMEG00000000707/ENSAMEG00000006918 | 8 |
| GO:0004842 | ubiquitin-protein transferase activity | 1.09E-04 | 2.40E-03 | 2.07E-03 | ENSAMEG00000012547/ENSAMEG00000001259/ENSAMEG00000000171/ENSAMEG00000010307/ENSAMEG00000008442/ENSAMEG00000012159/ENSAMEG00000011892/ENSAMEG00000002389/ENSAMEG00000007673/ENSAMEG00000003804/ENSAMEG00000008730/ENSAMEG00000017610/ENSAMEG00000004969/ENSAMEG00000001323/ENSAMEG00000004224/ENSAMEG00000017832/ENSAMEG00000015109/ENSAMEG00000003779/ENSAMEG00000011651/ENSAMEG00000005054/ENSAMEG00000013426/ENSAMEG00000013719/ENSAMEG00000015826/ENSAMEG00000001353/ENSAMEG00000017319/ENSAMEG00000018293/ENSAMEG00000015281/ENSAMEG00000015087/ENSAMEG00000016602/ENSAMEG00000011549/ENSAMEG00000010249/ENSAMEG00000008807 | 32 |
| GO:0000122 | negative regulation of transcription by RNA polymerase II | 1.23E-04 | 2.67E-03 | 2.32E-03 | ENSAMEG00000012588/ENSAMEG00000017950/ENSAMEG00000003466/ENSAMEG00000015813/ENSAMEG00000000014/ENSAMEG00000007247/ENSAMEG00000004869/ENSAMEG00000018175/ENSAMEG00000002580/ENSAMEG00000008991/ENSAMEG00000002791/ENSAMEG00000009668/ENSAMEG00000004257/ENSAMEG00000014232/ENSAMEG00000014221/ENSAMEG00000012415/ENSAMEG00000011823/ENSAMEG00000003946/ENSAMEG00000002934/ENSAMEG00000013940/ENSAMEG00000012595/ENSAMEG00000009089/ENSAMEG00000003640/ENSAMEG00000018491/ENSAMEG00000009813/ENSAMEG00000017889/ENSAMEG00000002670/ENSAMEG00000017609/ENSAMEG00000014308/ENSAMEG00000018404/ENSAMEG00000009592/ENSAMEG00000011355/ENSAMEG00000011651/ENSAMEG00000008481/ENSAMEG00000017412/ENSAMEG00000003503/ENSAMEG00000004772/ENSAMEG00000017438/ENSAMEG00000013018/ENSAMEG00000007608/ENSAMEG00000014816/ENSAMEG00000004496/ENSAMEG00000010127/ENSAMEG00000002882/ENSAMEG00000008231/ENSAMEG00000013257/ENSAMEG00000008438/ENSAMEG00000008158/ENSAMEG00000015417/ENSAMEG00000018268/ENSAMEG00000006307/ENSAMEG00000017390/ENSAMEG00000004665/ENSAMEG00000015293/ENSAMEG00000018211/ENSAMEG00000003168/ENSAMEG00000010750/ENSAMEG00000005490/ENSAMEG00000010113/ENSAMEG00000016108/ENSAMEG00000003974/ENSAMEG00000017424/ENSAMEG00000018326/ENSAMEG00000011668/ENSAMEG00000010799/ENSAMEG00000000707/ENSAMEG00000002167 | 67 |
| GO:0005721 | pericentric heterochromatin | 1.28E-04 | 2.75E-03 | 2.38E-03 | ENSAMEG00000016838/ENSAMEG00000011823/ENSAMEG00000003458/ENSAMEG00000001188/ENSAMEG00000011177/ENSAMEG00000000707 | 6 |
| GO:0000723 | telomere maintenance | 1.46E-04 | 3.06E-03 | 2.65E-03 | ENSAMEG00000018028/ENSAMEG00000005460/ENSAMEG00000009668/ENSAMEG00000003270/ENSAMEG00000011132/ENSAMEG00000005590/ENSAMEG00000017712/ENSAMEG00000007706/ENSAMEG00000017492 | 9 |
| GO:0003887 | DNA-directed DNA polymerase activity | 1.46E-04 | 3.06E-03 | 2.65E-03 | ENSAMEG00000012174/ENSAMEG00000014870/ENSAMEG00000013454/ENSAMEG00000014758/ENSAMEG00000009547/ENSAMEG00000017155/ENSAMEG00000013111/ENSAMEG00000017565 | 8 |
| GO:0015630 | microtubule cytoskeleton | 1.84E-04 | 3.82E-03 | 3.31E-03 | ENSAMEG00000014095/ENSAMEG00000014998/ENSAMEG00000013678/ENSAMEG00000007168/ENSAMEG00000005225/ENSAMEG00000017084/ENSAMEG00000004603/ENSAMEG00000014549/ENSAMEG00000014232/ENSAMEG00000001746/ENSAMEG00000004686/ENSAMEG00000011827/ENSAMEG00000006872/ENSAMEG00000011264/ENSAMEG00000000280/ENSAMEG00000016790/ENSAMEG00000007205/ENSAMEG00000012697/ENSAMEG00000015215/ENSAMEG00000017110/ENSAMEG00000003100 | 21 |
| GO:0036459 | thiol-dependent ubiquitinyl hydrolase activity | 1.92E-04 | 3.93E-03 | 3.40E-03 | ENSAMEG00000002210/ENSAMEG00000014832/ENSAMEG00000008169/ENSAMEG00000002927/ENSAMEG00000009899/ENSAMEG00000010726/ENSAMEG00000012591/ENSAMEG00000006687/ENSAMEG00000013061/ENSAMEG00000013920/ENSAMEG00000011298/ENSAMEG00000006853/ENSAMEG00000008960/ENSAMEG00000012785/ENSAMEG00000004155 | 15 |
| GO:2000042 | negative regulation of double-strand break repair via homologous recombination | 1.98E-04 | 4.02E-03 | 3.48E-03 | ENSAMEG00000014870/ENSAMEG00000006369/ENSAMEG00000000109/ENSAMEG00000008461/ENSAMEG00000010022/ENSAMEG00000009566/ENSAMEG00000002043 | 7 |
| GO:0097431 | mitotic spindle pole | 2.19E-04 | 4.38E-03 | 3.79E-03 | ENSAMEG00000017540/ENSAMEG00000011957/ENSAMEG00000007168/ENSAMEG00000004449/ENSAMEG00000016790/ENSAMEG00000005053/ENSAMEG00000015675/ENSAMEG00000010861 | 8 |
| GO:0000228 | nuclear chromosome | 2.38E-04 | 4.66E-03 | 4.03E-03 | ENSAMEG00000007989/ENSAMEG00000005936/ENSAMEG00000014358/ENSAMEG00000019297/ENSAMEG00000013843/ENSAMEG00000009674 | 6 |
| GO:0030131 | clathrin adaptor complex | 2.38E-04 | 4.66E-03 | 4.03E-03 | ENSAMEG00000006083/ENSAMEG00000003279/ENSAMEG00000011318/ENSAMEG00000009898/ENSAMEG00000008223/ENSAMEG00000017248 | 6 |
| GO:0008094 | DNA-dependent ATPase activity | 2.87E-04 | 5.56E-03 | 4.82E-03 | ENSAMEG00000007989/ENSAMEG00000016965/ENSAMEG00000004713/ENSAMEG00000013843/ENSAMEG00000016796/ENSAMEG00000010406/ENSAMEG00000013304/ENSAMEG00000010868/ENSAMEG00000006307 | 9 |
| GO:0046982 | protein heterodimerization activity | 3.75E-04 | 7.19E-03 | 6.23E-03 | ENSAMEG00000003988/ENSAMEG00000002112/ENSAMEG00000007989/ENSAMEG00000017537/ENSAMEG00000000014/ENSAMEG00000009491/ENSAMEG00000007168/ENSAMEG00000005936/ENSAMEG00000014758/ENSAMEG00000002220/ENSAMEG00000006809/ENSAMEG00000013246/ENSAMEG00000003946/ENSAMEG00000005766/ENSAMEG00000003804/ENSAMEG00000011337/ENSAMEG00000019961/ENSAMEG00000005053/ENSAMEG00000005246/ENSAMEG00000015932/ENSAMEG00000017565/ENSAMEG00000018575/ENSAMEG00000003503/ENSAMEG00000000961/ENSAMEG00000018498/ENSAMEG00000016226/ENSAMEG00000015779/ENSAMEG00000008073/ENSAMEG00000018579/ENSAMEG00000014449/ENSAMEG00000017110/ENSAMEG00000018517/ENSAMEG00000001277/ENSAMEG00000015431/ENSAMEG00000006241/ENSAMEG00000002199/ENSAMEG00000017687/ENSAMEG00000015675/ENSAMEG00000009664/ENSAMEG00000000410/ENSAMEG00000006364/ENSAMEG00000012998/ENSAMEG00000007355/ENSAMEG00000000959/ENSAMEG00000013710/ENSAMEG00000016139/ENSAMEG00000017781 | 47 |
| GO:0042800 | histone methyltransferase activity (H3-K4 specific) | 4.09E-04 | 7.61E-03 | 6.60E-03 | ENSAMEG00000013308/ENSAMEG00000011076/ENSAMEG00000007540/ENSAMEG00000000319/ENSAMEG00000000259/ENSAMEG00000006918 | 6 |
| GO:0070182 | DNA polymerase binding | 4.09E-04 | 7.61E-03 | 6.60E-03 | ENSAMEG00000016051/ENSAMEG00000012957/ENSAMEG00000009089/ENSAMEG00000010868/ENSAMEG00000017492/ENSAMEG00000008124 | 6 |
| GO:0070577 | lysine-acetylated histone binding | 4.09E-04 | 7.61E-03 | 6.60E-03 | ENSAMEG00000013286/ENSAMEG00000002954/ENSAMEG00000011015/ENSAMEG00000002853/ENSAMEG00000004483/ENSAMEG00000010868 | 6 |
| GO:0004518 | nuclease activity | 4.16E-04 | 7.66E-03 | 6.64E-03 | ENSAMEG00000013934/ENSAMEG00000011867/ENSAMEG00000018028/ENSAMEG00000002666/ENSAMEG00000002114/ENSAMEG00000003608/ENSAMEG00000019971/ENSAMEG00000005019/ENSAMEG00000005278/ENSAMEG00000016594/ENSAMEG00000007626 | 11 |
| GO:0007093 | mitotic cell cycle checkpoint | 4.51E-04 | 8.22E-03 | 7.12E-03 | ENSAMEG00000014771/ENSAMEG00000005342/ENSAMEG00000012771/ENSAMEG00000003270/ENSAMEG00000001064/ENSAMEG00000003251/ENSAMEG00000012140/ENSAMEG00000013710 | 8 |
| GO:0034968 | histone lysine methylation | 4.73E-04 | 8.46E-03 | 7.33E-03 | ENSAMEG00000013308/ENSAMEG00000012595/ENSAMEG00000007540/ENSAMEG00000001160/ENSAMEG00000012781/ENSAMEG00000011260/ENSAMEG00000006918 | 7 |
| GO:0051225 | spindle assembly | 4.73E-04 | 8.46E-03 | 7.33E-03 | ENSAMEG00000014099/ENSAMEG00000012472/ENSAMEG00000011337/ENSAMEG00000000503/ENSAMEG00000016796/ENSAMEG00000012644/ENSAMEG00000002777 | 7 |
| GO:0001102 | RNA polymerase II activating transcription factor binding | 5.23E-04 | 9.27E-03 | 8.03E-03 | ENSAMEG00000013940/ENSAMEG00000017674/ENSAMEG00000003640/ENSAMEG00000017889/ENSAMEG00000007383/ENSAMEG00000009592/ENSAMEG00000008481/ENSAMEG00000015152/ENSAMEG00000008231 | 9 |
| GO:0035861 | site of double-strand break | 5.42E-04 | 9.51E-03 | 8.24E-03 | ENSAMEG00000000137/ENSAMEG00000003946/ENSAMEG00000012375/ENSAMEG00000011132/ENSAMEG00000001323/ENSAMEG00000000109/ENSAMEG00000008461/ENSAMEG00000010022/ENSAMEG00000007872/ENSAMEG00000007626 | 10 |
| GO:0047485 | protein N-terminus binding | 5.60E-04 | 9.73E-03 | 8.43E-03 | ENSAMEG00000005342/ENSAMEG00000000747/ENSAMEG00000011446/ENSAMEG00000012595/ENSAMEG00000010406/ENSAMEG00000011272/ENSAMEG00000004214/ENSAMEG00000018018/ENSAMEG00000011868/ENSAMEG00000007608/ENSAMEG00000010868/ENSAMEG00000016764/ENSAMEG00000015417/ENSAMEG00000010113/ENSAMEG00000010690 | 15 |
| GO:0090307 | mitotic spindle assembly | 6.25E-04 | 1.08E-02 | 9.33E-03 | ENSAMEG00000014099/ENSAMEG00000013678/ENSAMEG00000001458/ENSAMEG00000001187/ENSAMEG00000001637/ENSAMEG00000003831/ENSAMEG00000008484/ENSAMEG00000017740 | 8 |
| GO:0045893 | positive regulation of transcription, DNA-templated | 6.49E-04 | 1.11E-02 | 9.59E-03 | ENSAMEG00000012588/ENSAMEG00000012407/ENSAMEG00000006109/ENSAMEG00000000014/ENSAMEG00000004869/ENSAMEG00000001061/ENSAMEG00000000171/ENSAMEG00000008991/ENSAMEG00000011109/ENSAMEG00000009390/ENSAMEG00000012594/ENSAMEG00000005514/ENSAMEG00000014957/ENSAMEG00000004686/ENSAMEG00000013843/ENSAMEG00000013940/ENSAMEG00000017610/ENSAMEG00000019816/ENSAMEG00000002954/ENSAMEG00000001233/ENSAMEG00000009813/ENSAMEG00000017889/ENSAMEG00000008484/ENSAMEG00000015152/ENSAMEG00000018450/ENSAMEG00000003503/ENSAMEG00000017438/ENSAMEG00000014864/ENSAMEG00000014262/ENSAMEG00000009792/ENSAMEG00000007608/ENSAMEG00000004645/ENSAMEG00000010868/ENSAMEG00000001186/ENSAMEG00000001085/ENSAMEG00000007492/ENSAMEG00000016764/ENSAMEG00000000379/ENSAMEG00000008158/ENSAMEG00000018268/ENSAMEG00000006241/ENSAMEG00000006307/ENSAMEG00000003168/ENSAMEG00000010750/ENSAMEG00000000259/ENSAMEG00000020087/ENSAMEG00000003974/ENSAMEG00000012998/ENSAMEG00000009012/ENSAMEG00000008636/ENSAMEG00000006185/ENSAMEG00000010249/ENSAMEG00000010799/ENSAMEG00000002111/ENSAMEG00000004446/ENSAMEG00000008706 | 56 |
| GO:0007062 | sister chromatid cohesion | 6.64E-04 | 1.11E-02 | 9.63E-03 | ENSAMEG00000014825/ENSAMEG00000004713/ENSAMEG00000005053/ENSAMEG00000001648/ENSAMEG00000015675/ENSAMEG00000007626 | 6 |
| GO:0060236 | regulation of mitotic spindle organization | 6.64E-04 | 1.11E-02 | 9.63E-03 | ENSAMEG00000013678/ENSAMEG00000016924/ENSAMEG00000004686/ENSAMEG00000004449/ENSAMEG00000019816/ENSAMEG00000010861 | 6 |
| GO:0016363 | nuclear matrix | 7.31E-04 | 1.21E-02 | 1.05E-02 | ENSAMEG00000015869/ENSAMEG00000003466/ENSAMEG00000004257/ENSAMEG00000010051/ENSAMEG00000013843/ENSAMEG00000001664/ENSAMEG00000005053/ENSAMEG00000015417/ENSAMEG00000002281/ENSAMEG00000001648/ENSAMEG00000015675/ENSAMEG00000010861 | 12 |
| GO:0007010 | cytoskeleton organization | 7.68E-04 | 1.26E-02 | 1.09E-02 | ENSAMEG00000003546/ENSAMEG00000012145/ENSAMEG00000005465/ENSAMEG00000002203/ENSAMEG00000003459/ENSAMEG00000005915/ENSAMEG00000006872/ENSAMEG00000012351/ENSAMEG00000016734/ENSAMEG00000016380/ENSAMEG00000014080/ENSAMEG00000003949/ENSAMEG00000011431/ENSAMEG00000016735/ENSAMEG00000000987/ENSAMEG00000007112/ENSAMEG00000004155/ENSAMEG00000016021 | 18 |
| GO:0001833 | inner cell mass cell proliferation | 8.23E-04 | 1.26E-02 | 1.09E-02 | ENSAMEG00000009390/ENSAMEG00000001064/ENSAMEG00000002731/ENSAMEG00000014287/ENSAMEG00000012998 | 5 |
| GO:0006336 | DNA replication-independent nucleosome assembly | 8.23E-04 | 1.26E-02 | 1.09E-02 | ENSAMEG00000013206/ENSAMEG00000001673/ENSAMEG00000013676/ENSAMEG00000011366/ENSAMEG00000013815 | 5 |
| GO:0006783 | heme biosynthetic process | 8.23E-04 | 1.26E-02 | 1.09E-02 | ENSAMEG00000015511/ENSAMEG00000014813/ENSAMEG00000015509/ENSAMEG00000013467/ENSAMEG00000002188 | 5 |
| GO:0008409 | 5'-3' exonuclease activity | 8.23E-04 | 1.26E-02 | 1.09E-02 | ENSAMEG00000003270/ENSAMEG00000019971/ENSAMEG00000012538/ENSAMEG00000017693/ENSAMEG00000007626 | 5 |
| GO:0017116 | single-stranded DNA helicase activity | 8.23E-04 | 1.26E-02 | 1.09E-02 | ENSAMEG00000018028/ENSAMEG00000014870/ENSAMEG00000002148/ENSAMEG00000007469/ENSAMEG00000000109 | 5 |
| GO:0042054 | histone methyltransferase activity | 8.23E-04 | 1.26E-02 | 1.09E-02 | ENSAMEG00000002791/ENSAMEG00000005436/ENSAMEG00000012595/ENSAMEG00000011015/ENSAMEG00000005490 | 5 |
| GO:0043596 | nuclear replication fork | 8.23E-04 | 1.26E-02 | 1.09E-02 | ENSAMEG00000005645/ENSAMEG00000002533/ENSAMEG00000009089/ENSAMEG00000011177/ENSAMEG00000016419 | 5 |
| GO:1990023 | mitotic spindle midzone | 8.23E-04 | 1.26E-02 | 1.09E-02 | ENSAMEG00000002580/ENSAMEG00000014549/ENSAMEG00000016790/ENSAMEG00000010861/ENSAMEG00000000769 | 5 |
| GO:0007049 | cell cycle | 9.00E-04 | 1.37E-02 | 1.19E-02 | ENSAMEG00000014358/ENSAMEG00000007193/ENSAMEG00000008950/ENSAMEG00000000171/ENSAMEG00000015323/ENSAMEG00000011150/ENSAMEG00000003344/ENSAMEG00000017110/ENSAMEG00000016594 | 9 |
| GO:0000123 | histone acetyltransferase complex | 9.88E-04 | 1.49E-02 | 1.29E-02 | ENSAMEG00000013940/ENSAMEG00000018450/ENSAMEG00000016129/ENSAMEG00000006794/ENSAMEG00000010542/ENSAMEG00000012243/ENSAMEG00000010799 | 7 |
| GO:0007095 | mitotic G2 DNA damage checkpoint | 1.03E-03 | 1.50E-02 | 1.30E-02 | ENSAMEG00000000679/ENSAMEG00000003520/ENSAMEG00000013843/ENSAMEG00000007626/ENSAMEG00000001627/ENSAMEG00000014866 | 6 |
| GO:0048821 | erythrocyte development | 1.03E-03 | 1.50E-02 | 1.30E-02 | ENSAMEG00000012588/ENSAMEG00000018346/ENSAMEG00000003459/ENSAMEG00000002188/ENSAMEG00000003974/ENSAMEG00000002532 | 6 |
| GO:0051233 | spindle midzone | 1.03E-03 | 1.50E-02 | 1.30E-02 | ENSAMEG00000001093/ENSAMEG00000012611/ENSAMEG00000014232/ENSAMEG00000007868/ENSAMEG00000016790/ENSAMEG00000008461 | 6 |
| GO:0070530 | K63-linked polyubiquitin modification-dependent protein binding | 1.03E-03 | 1.50E-02 | 1.30E-02 | ENSAMEG00000007365/ENSAMEG00000007888/ENSAMEG00000000410/ENSAMEG00000007940/ENSAMEG00000016419/ENSAMEG00000004155 | 6 |
| GO:0002039 | p53 binding | 1.07E-03 | 1.55E-02 | 1.35E-02 | ENSAMEG00000012588/ENSAMEG00000013843/ENSAMEG00000013940/ENSAMEG00000001323/ENSAMEG00000008481/ENSAMEG00000003233/ENSAMEG00000010726/ENSAMEG00000007608/ENSAMEG00000010868/ENSAMEG00000006307/ENSAMEG00000007355/ENSAMEG00000010799 | 12 |
| GO:0032436 | positive regulation of proteasomal ubiquitin-dependent protein catabolic process | 1.10E-03 | 1.58E-02 | 1.37E-02 | ENSAMEG00000007168/ENSAMEG00000014232/ENSAMEG00000005766/ENSAMEG00000017567/ENSAMEG00000012527/ENSAMEG00000000272/ENSAMEG00000002882/ENSAMEG00000015759/ENSAMEG00000018812/ENSAMEG00000013920 | 10 |
| GO:0043966 | histone H3 acetylation | 1.16E-03 | 1.65E-02 | 1.43E-02 | ENSAMEG00000009390/ENSAMEG00000000336/ENSAMEG00000001319/ENSAMEG00000002161/ENSAMEG00000017390/ENSAMEG00000009201/ENSAMEG00000010542/ENSAMEG00000006364/ENSAMEG00000012243 | 9 |
| GO:0003690 | double-stranded DNA binding | 1.22E-03 | 1.73E-02 | 1.50E-02 | ENSAMEG00000016965/ENSAMEG00000013505/ENSAMEG00000010051/ENSAMEG00000015678/ENSAMEG00000004713/ENSAMEG00000018446/ENSAMEG00000014262/ENSAMEG00000001905/ENSAMEG00000002043/ENSAMEG00000009674/ENSAMEG00000013999 | 11 |
| GO:0045739 | positive regulation of DNA repair | 1.37E-03 | 1.91E-02 | 1.66E-02 | ENSAMEG00000000171/ENSAMEG00000009089/ENSAMEG00000010406/ENSAMEG00000010022/ENSAMEG00000007365/ENSAMEG00000004887/ENSAMEG00000011164 | 7 |
| GO:0000400 | four-way junction DNA binding | 1.40E-03 | 1.91E-02 | 1.66E-02 | ENSAMEG00000002666/ENSAMEG00000013843/ENSAMEG00000013304/ENSAMEG00000016250/ENSAMEG00000014262 | 5 |
| GO:0001556 | oocyte maturation | 1.40E-03 | 1.91E-02 | 1.66E-02 | ENSAMEG00000010721/ENSAMEG00000009390/ENSAMEG00000008267/ENSAMEG00000008158/ENSAMEG00000009340 | 5 |
| GO:0051571 | positive regulation of histone H3-K4 methylation | 1.40E-03 | 1.91E-02 | 1.66E-02 | ENSAMEG00000000171/ENSAMEG00000011823/ENSAMEG00000009813/ENSAMEG00000001238/ENSAMEG00000013257 | 5 |
| GO:0071168 | protein localization to chromatin | 1.40E-03 | 1.91E-02 | 1.66E-02 | ENSAMEG00000000137/ENSAMEG00000002791/ENSAMEG00000014232/ENSAMEG00000011015/ENSAMEG00000001648 | 5 |
| GO:0010971 | positive regulation of G2/M transition of mitotic cell cycle | 1.52E-03 | 2.03E-02 | 1.76E-02 | ENSAMEG00000003520/ENSAMEG00000001259/ENSAMEG00000000492/ENSAMEG00000001260/ENSAMEG00000008231/ENSAMEG00000000769 | 6 |
| GO:0030863 | cortical cytoskeleton | 1.52E-03 | 2.03E-02 | 1.76E-02 | ENSAMEG00000003988/ENSAMEG00000018346/ENSAMEG00000015596/ENSAMEG00000003459/ENSAMEG00000011446/ENSAMEG00000004831 | 6 |
| GO:0070979 | protein K11-linked ubiquitination | 1.52E-03 | 2.03E-02 | 1.76E-02 | ENSAMEG00000012547/ENSAMEG00000011892/ENSAMEG00000016275/ENSAMEG00000016483/ENSAMEG00000003779/ENSAMEG00000019700 | 6 |
| GO:0006325 | chromatin organization | 1.79E-03 | 2.37E-02 | 2.06E-02 | ENSAMEG00000011109/ENSAMEG00000001673/ENSAMEG00000005436/ENSAMEG00000012595/ENSAMEG00000018491/ENSAMEG00000007608/ENSAMEG00000006307/ENSAMEG00000010750/ENSAMEG00000018326/ENSAMEG00000011366/ENSAMEG00000015722 | 11 |
| GO:0007265 | Ras protein signal transduction | 1.85E-03 | 2.42E-02 | 2.09E-02 | ENSAMEG00000006109/ENSAMEG00000014672/ENSAMEG00000011823/ENSAMEG00000003640/ENSAMEG00000005246/ENSAMEG00000003671/ENSAMEG00000013719/ENSAMEG00000001160/ENSAMEG00000003168 | 9 |
| GO:0034644 | cellular response to UV | 1.85E-03 | 2.42E-02 | 2.09E-02 | ENSAMEG00000002580/ENSAMEG00000001398/ENSAMEG00000000135/ENSAMEG00000004884/ENSAMEG00000013940/ENSAMEG00000009089/ENSAMEG00000016796/ENSAMEG00000010288/ENSAMEG00000010799 | 9 |
| GO:0008234 | cysteine-type peptidase activity | 1.97E-03 | 2.56E-02 | 2.22E-02 | ENSAMEG00000002210/ENSAMEG00000012346/ENSAMEG00000011409/ENSAMEG00000012294/ENSAMEG00000014832/ENSAMEG00000002927/ENSAMEG00000009899/ENSAMEG00000009736/ENSAMEG00000009127/ENSAMEG00000012591/ENSAMEG00000013767/ENSAMEG00000006687/ENSAMEG00000013061/ENSAMEG00000013920/ENSAMEG00000008960/ENSAMEG00000006047 | 16 |
| GO:0001650 | fibrillar center | 2.08E-03 | 2.67E-02 | 2.32E-02 | ENSAMEG00000018347/ENSAMEG00000005284/ENSAMEG00000000792/ENSAMEG00000004713/ENSAMEG00000003608/ENSAMEG00000012524/ENSAMEG00000013333/ENSAMEG00000007104/ENSAMEG00000004021/ENSAMEG00000012301/ENSAMEG00000014644/ENSAMEG00000017412/ENSAMEG00000003944/ENSAMEG00000010127/ENSAMEG00000005029/ENSAMEG00000002562/ENSAMEG00000017687/ENSAMEG00000018342/ENSAMEG00000009674/ENSAMEG00000006277 | 20 |
| GO:0000132 | establishment of mitotic spindle orientation | 2.18E-03 | 2.77E-02 | 2.40E-02 | ENSAMEG00000002112/ENSAMEG00000010069/ENSAMEG00000014232/ENSAMEG00000013777/ENSAMEG00000004449/ENSAMEG00000010861 | 6 |
| GO:0035855 | megakaryocyte development | 2.18E-03 | 2.77E-02 | 2.40E-02 | ENSAMEG00000000014/ENSAMEG00000009186/ENSAMEG00000013940/ENSAMEG00000015779/ENSAMEG00000003974/ENSAMEG00000006281 | 6 |
| GO:0019825 | oxygen binding | 2.23E-03 | 2.80E-02 | 2.42E-02 | ENSAMEG00000006868/ENSAMEG00000013439/ENSAMEG00000006871/ENSAMEG00000013463/ENSAMEG00000006866 | 5 |
| GO:0045740 | positive regulation of DNA replication | 2.23E-03 | 2.80E-02 | 2.42E-02 | ENSAMEG00000018028/ENSAMEG00000005534/ENSAMEG00000000371/ENSAMEG00000009089/ENSAMEG00000002560 | 5 |
| GO:0008022 | protein C-terminus binding | 2.33E-03 | 2.90E-02 | 2.51E-02 | ENSAMEG00000015869/ENSAMEG00000007989/ENSAMEG00000001637/ENSAMEG00000009390/ENSAMEG00000011446/ENSAMEG00000013940/ENSAMEG00000004449/ENSAMEG00000005019/ENSAMEG00000013188/ENSAMEG00000005246/ENSAMEG00000010406/ENSAMEG00000016250/ENSAMEG00000010726/ENSAMEG00000016764/ENSAMEG00000016972/ENSAMEG00000016043/ENSAMEG00000007626/ENSAMEG00000010861/ENSAMEG00000001627 | 19 |
| GO:0001701 | in utero embryonic development | 2.83E-03 | 3.49E-02 | 3.02E-02 | ENSAMEG00000012588/ENSAMEG00000004603/ENSAMEG00000002139/ENSAMEG00000018105/ENSAMEG00000019061/ENSAMEG00000009575/ENSAMEG00000017216/ENSAMEG00000013304/ENSAMEG00000014287/ENSAMEG00000010877/ENSAMEG00000014449/ENSAMEG00000008231/ENSAMEG00000016764/ENSAMEG00000006241/ENSAMEG00000017728/ENSAMEG00000006307/ENSAMEG00000001394/ENSAMEG00000003974/ENSAMEG00000009769/ENSAMEG00000017987/ENSAMEG00000013710/ENSAMEG00000002167/ENSAMEG00000006989 | 23 |
| GO:0007017 | microtubule-based process | 2.84E-03 | 3.49E-02 | 3.02E-02 | ENSAMEG00000012145/ENSAMEG00000012472/ENSAMEG00000013184/ENSAMEG00000006872/ENSAMEG00000016734/ENSAMEG00000016380/ENSAMEG00000011431/ENSAMEG00000016735/ENSAMEG00000007112 | 9 |
| GO:0006298 | mismatch repair | 3.04E-03 | 3.71E-02 | 3.21E-02 | ENSAMEG00000013934/ENSAMEG00000009089/ENSAMEG00000011132/ENSAMEG00000007706/ENSAMEG00000017905/ENSAMEG00000012781 | 6 |
| GO:0000086 | G2/M transition of mitotic cell cycle | 3.25E-03 | 3.84E-02 | 3.32E-02 | ENSAMEG00000006109/ENSAMEG00000004869/ENSAMEG00000016520/ENSAMEG00000014232/ENSAMEG00000001064/ENSAMEG00000001855/ENSAMEG00000008484 | 7 |
| GO:0001578 | microtubule bundle formation | 3.25E-03 | 3.84E-02 | 3.32E-02 | ENSAMEG00000013667/ENSAMEG00000016438/ENSAMEG00000014232/ENSAMEG00000004686/ENSAMEG00000001233/ENSAMEG00000005084/ENSAMEG00000010861 | 7 |
| GO:0006284 | base-excision repair | 3.25E-03 | 3.84E-02 | 3.32E-02 | ENSAMEG00000018028/ENSAMEG00000003715/ENSAMEG00000014870/ENSAMEG00000015091/ENSAMEG00000011132/ENSAMEG00000010406/ENSAMEG00000007706 | 7 |
| GO:0070491 | repressing transcription factor binding | 3.25E-03 | 3.84E-02 | 3.32E-02 | ENSAMEG00000003503/ENSAMEG00000007608/ENSAMEG00000001277/ENSAMEG00000010211/ENSAMEG00000018326/ENSAMEG00000000707/ENSAMEG00000017055 | 7 |
| GO:0071479 | cellular response to ionizing radiation | 3.25E-03 | 3.84E-02 | 3.32E-02 | ENSAMEG00000001035/ENSAMEG00000015678/ENSAMEG00000019297/ENSAMEG00000013843/ENSAMEG00000004884/ENSAMEG00000016796/ENSAMEG00000001226 | 7 |
| GO:0000784 | nuclear chromosome, telomeric region | 3.43E-03 | 4.00E-02 | 3.47E-02 | ENSAMEG00000018028/ENSAMEG00000009254/ENSAMEG00000009390/ENSAMEG00000003270/ENSAMEG00000019971/ENSAMEG00000010022/ENSAMEG00000002560/ENSAMEG00000014841/ENSAMEG00000007626/ENSAMEG00000011474/ENSAMEG00000000707/ENSAMEG00000017492 | 12 |
| GO:0006338 | chromatin remodeling | 3.43E-03 | 4.00E-02 | 3.47E-02 | ENSAMEG00000016796/ENSAMEG00000009813/ENSAMEG00000008496/ENSAMEG00000017063/ENSAMEG00000010868/ENSAMEG00000010750/ENSAMEG00000011177/ENSAMEG00000009674/ENSAMEG00000015512/ENSAMEG00000015218/ENSAMEG00000005584/ENSAMEG00000002111 | 12 |
| GO:0016301 | kinase activity | 3.46E-03 | 4.01E-02 | 3.48E-02 | ENSAMEG00000015972/ENSAMEG00000003520/ENSAMEG00000009668/ENSAMEG00000013744/ENSAMEG00000014232/ENSAMEG00000017924/ENSAMEG00000010058/ENSAMEG00000008089/ENSAMEG00000000492/ENSAMEG00000015426/ENSAMEG00000009289/ENSAMEG00000010292/ENSAMEG00000013432/ENSAMEG00000013839/ENSAMEG00000012351/ENSAMEG00000009661/ENSAMEG00000018159/ENSAMEG00000005226/ENSAMEG00000001855/ENSAMEG00000012301/ENSAMEG00000013791/ENSAMEG00000004552/ENSAMEG00000016250/ENSAMEG00000016226/ENSAMEG00000018110/ENSAMEG00000015971/ENSAMEG00000008397/ENSAMEG00000003944/ENSAMEG00000007260/ENSAMEG00000006794/ENSAMEG00000003362/ENSAMEG00000013754/ENSAMEG00000017949/ENSAMEG00000007077/ENSAMEG00000003168/ENSAMEG00000000822/ENSAMEG00000018163/ENSAMEG00000006281/ENSAMEG00000015916/ENSAMEG00000005584/ENSAMEG00000009856 | 41 |
| GO:0015293 | symporter activity | 3.90E-03 | 4.46E-02 | 3.86E-02 | ENSAMEG00000002807/ENSAMEG00000001028/ENSAMEG00000010403/ENSAMEG00000008407/ENSAMEG00000003959/ENSAMEG00000001092/ENSAMEG00000005668/ENSAMEG00000010690 | 8 |
| GO:0032465 | regulation of cytokinesis | 3.90E-03 | 4.46E-02 | 3.86E-02 | ENSAMEG00000013667/ENSAMEG00000016438/ENSAMEG00000009390/ENSAMEG00000014232/ENSAMEG00000014294/ENSAMEG00000012781/ENSAMEG00000011958/ENSAMEG00000013710 | 8 |
| GO:0000226 | microtubule cytoskeleton organization | 3.98E-03 | 4.52E-02 | 3.92E-02 | ENSAMEG00000013667/ENSAMEG00000016762/ENSAMEG00000007168/ENSAMEG00000013864/ENSAMEG00000003841/ENSAMEG00000001233/ENSAMEG00000001250/ENSAMEG00000005084/ENSAMEG00000015215/ENSAMEG00000002382/ENSAMEG00000001007/ENSAMEG00000011574 | 12 |
| GO:0005680 | anaphase-promoting complex | 4.12E-03 | 4.66E-02 | 4.04E-02 | ENSAMEG00000012547/ENSAMEG00000001637/ENSAMEG00000016275/ENSAMEG00000016483/ENSAMEG00000003779/ENSAMEG00000005634 | 6 |
