# Supplementary material for: Gene expression profiles during postnatal development of the liver and pancreas in giant pandas
Source: Aging (Albany NY). 2020 Aug 15;12(15):15705–29. doi: 10.18632/aging.103783 (PMC7467380; doi:10.18632/aging.103783)
Supplement: Supplementary Table 5 [file aging-12-103783-s009..docx]

**Supplementary Table 5. Significantly enriched KEGG pathways for down-regulated DEGs in liver adult group compared with liver suckling group.**

| **ID** | **Description** | **pvalue** | **p.adjust** | **qvalue** | **geneID** | **Count** |
| --- | --- | --- | --- | --- | --- | --- |
| aml04110 | Cell cycle [PATH:aml04110] | 1.68E-30 | 4.67E-28 | 4.38E-28 | ENSAMEG00000014998/ENSAMEG00000003117/ENSAMEG00000009532/ENSAMEG00000001634/ENSAMEG00000006109/ENSAMEG00000005841/ENSAMEG00000009662/ENSAMEG00000010721/ENSAMEG00000006653/ENSAMEG00000012771/ENSAMEG00000009254/ENSAMEG00000011443/ENSAMEG00000018347/ENSAMEG00000001637/ENSAMEG00000008540/ENSAMEG00000016520/ENSAMEG00000004603/ENSAMEG00000009668/ENSAMEG00000001398/ENSAMEG00000014232/ENSAMEG00000012415/ENSAMEG00000000492/ENSAMEG00000016275/ENSAMEG00000011150/ENSAMEG00000012466/ENSAMEG00000017203/ENSAMEG00000007868/ENSAMEG00000001421/ENSAMEG00000013940/ENSAMEG00000012346/ENSAMEG00000011370/ENSAMEG00000017478/ENSAMEG00000009089/ENSAMEG00000003841/ENSAMEG00000017275/ENSAMEG00000005590/ENSAMEG00000003640/ENSAMEG00000001199/ENSAMEG00000005053/ENSAMEG00000008484/ENSAMEG00000016483/ENSAMEG00000005634/ENSAMEG00000016250/ENSAMEG00000013018/ENSAMEG00000007608/ENSAMEG00000004645/ENSAMEG00000017811/ENSAMEG00000008397/ENSAMEG00000001648/ENSAMEG00000003168/ENSAMEG00000009521/ENSAMEG00000015675/ENSAMEG00000008828/ENSAMEG00000010799/ENSAMEG00000010769 | 55 |
| aml03030 | DNA replication [PATH:aml03030] | 2.65E-14 | 3.68E-12 | 3.46E-12 | ENSAMEG00000014998/ENSAMEG00000001634/ENSAMEG00000005841/ENSAMEG00000018028/ENSAMEG00000008540/ENSAMEG00000015091/ENSAMEG00000013454/ENSAMEG00000014758/ENSAMEG00000009547/ENSAMEG00000011150/ENSAMEG00000001421/ENSAMEG00000017155/ENSAMEG00000013111/ENSAMEG00000019971/ENSAMEG00000009089/ENSAMEG00000011132/ENSAMEG00000017565/ENSAMEG00000004198/ENSAMEG00000017348/ENSAMEG00000007706 | 20 |
| aml03440 | Homologous recombination [PATH:aml03440] | 1.74E-10 | 1.61E-08 | 1.52E-08 | ENSAMEG00000011867/ENSAMEG00000009390/ENSAMEG00000013902/ENSAMEG00000005834/ENSAMEG00000003804/ENSAMEG00000017155/ENSAMEG00000013111/ENSAMEG00000008785/ENSAMEG00000011132/ENSAMEG00000000352/ENSAMEG00000014287/ENSAMEG00000004324/ENSAMEG00000017712/ENSAMEG00000007365/ENSAMEG00000007706/ENSAMEG00000004887/ENSAMEG00000007626 | 17 |
| aml03460 | Fanconi anemia pathway [PATH:aml03460] | 7.49E-09 | 5.21E-07 | 4.89E-07 | ENSAMEG00000011867/ENSAMEG00000012174/ENSAMEG00000009390/ENSAMEG00000011892/ENSAMEG00000016051/ENSAMEG00000002114/ENSAMEG00000012957/ENSAMEG00000008785/ENSAMEG00000015491/ENSAMEG00000011132/ENSAMEG00000015524/ENSAMEG00000014832/ENSAMEG00000014287/ENSAMEG00000015826/ENSAMEG00000011534/ENSAMEG00000007706/ENSAMEG00000017905 | 17 |
| aml04114 | Oocyte meiosis [PATH:aml04114] | 3.31E-06 | 1.84E-04 | 1.73E-04 | ENSAMEG00000003117/ENSAMEG00000009532/ENSAMEG00000007168/ENSAMEG00000006653/ENSAMEG00000001637/ENSAMEG00000000913/ENSAMEG00000016520/ENSAMEG00000004603/ENSAMEG00000009668/ENSAMEG00000014232/ENSAMEG00000000454/ENSAMEG00000016275/ENSAMEG00000011342/ENSAMEG00000001260/ENSAMEG00000012346/ENSAMEG00000017610/ENSAMEG00000005590/ENSAMEG00000005053/ENSAMEG00000016483/ENSAMEG00000007210/ENSAMEG00000004233/ENSAMEG00000008397/ENSAMEG00000003168/ENSAMEG00000015675 | 24 |
| aml05166 | Human T-cell leukemia virus 1 infection [PATH:aml05166] | 2.87E-05 | 1.27E-03 | 1.19E-03 | ENSAMEG00000006109/ENSAMEG00000004472/ENSAMEG00000009662/ENSAMEG00000006653/ENSAMEG00000001637/ENSAMEG00000004603/ENSAMEG00000009668/ENSAMEG00000016275/ENSAMEG00000017203/ENSAMEG00000013940/ENSAMEG00000012346/ENSAMEG00000017478/ENSAMEG00000005590/ENSAMEG00000003640/ENSAMEG00000005226/ENSAMEG00000002058/ENSAMEG00000008484/ENSAMEG00000016483/ENSAMEG00000015152/ENSAMEG00000007210/ENSAMEG00000013018/ENSAMEG00000013883/ENSAMEG00000006794/ENSAMEG00000005095/ENSAMEG00000002199/ENSAMEG00000003168/ENSAMEG00000009574/ENSAMEG00000009521/ENSAMEG00000000410/ENSAMEG00000010799/ENSAMEG00000013710/ENSAMEG00000006086/ENSAMEG00000017055 | 33 |
| aml04218 | Cellular senescence [PATH:aml04218] | 3.20E-05 | 1.27E-03 | 1.19E-03 | ENSAMEG00000003117/ENSAMEG00000006109/ENSAMEG00000009662/ENSAMEG00000004869/ENSAMEG00000003831/ENSAMEG00000004603/ENSAMEG00000009668/ENSAMEG00000001398/ENSAMEG00000012415/ENSAMEG00000003215/ENSAMEG00000017610/ENSAMEG00000017275/ENSAMEG00000005590/ENSAMEG00000003640/ENSAMEG00000005226/ENSAMEG00000005042/ENSAMEG00000008484/ENSAMEG00000004214/ENSAMEG00000017712/ENSAMEG00000013018/ENSAMEG00000017811/ENSAMEG00000014535/ENSAMEG00000005095/ENSAMEG00000003168/ENSAMEG00000007626 | 25 |
| aml04914 | Progesterone-mediated oocyte maturation [PATH:aml04914] | 5.08E-05 | 1.77E-03 | 1.66E-03 | ENSAMEG00000003117/ENSAMEG00000009532/ENSAMEG00000006109/ENSAMEG00000007168/ENSAMEG00000010721/ENSAMEG00000014825/ENSAMEG00000016520/ENSAMEG00000004603/ENSAMEG00000001398/ENSAMEG00000014232/ENSAMEG00000016275/ENSAMEG00000011342/ENSAMEG00000005226/ENSAMEG00000016483/ENSAMEG00000005634/ENSAMEG00000007210/ENSAMEG00000008397/ENSAMEG00000003168 | 18 |
| aml03430 | Mismatch repair [PATH:aml03430] | 2.02E-04 | 6.24E-03 | 5.86E-03 | ENSAMEG00000013934/ENSAMEG00000015091/ENSAMEG00000017155/ENSAMEG00000013111/ENSAMEG00000009089/ENSAMEG00000011132/ENSAMEG00000007706/ENSAMEG00000017905 | 8 |
| aml03420 | Nucleotide excision repair [PATH:aml03420] | 3.74E-04 | 9.28E-03 | 8.72E-03 | ENSAMEG00000015091/ENSAMEG00000013454/ENSAMEG00000009547/ENSAMEG00000017155/ENSAMEG00000013111/ENSAMEG00000009089/ENSAMEG00000011132/ENSAMEG00000010406/ENSAMEG00000016518/ENSAMEG00000007706/ENSAMEG00000010104 | 11 |
| aml05203 | Viral carcinogenesis [PATH:aml05203] | 3.84E-04 | 9.28E-03 | 8.72E-03 | ENSAMEG00000006109/ENSAMEG00000001637/ENSAMEG00000009668/ENSAMEG00000012415/ENSAMEG00000013940/ENSAMEG00000005590/ENSAMEG00000003640/ENSAMEG00000005226/ENSAMEG00000000895/ENSAMEG00000015152/ENSAMEG00000018498/ENSAMEG00000010726/ENSAMEG00000020264/ENSAMEG00000013018/ENSAMEG00000007608/ENSAMEG00000017811/ENSAMEG00000016518/ENSAMEG00000018517/ENSAMEG00000005095/ENSAMEG00000002199/ENSAMEG00000003168/ENSAMEG00000000410/ENSAMEG00000018326/ENSAMEG00000010799/ENSAMEG00000010769/ENSAMEG00000006086/ENSAMEG00000017055 | 27 |
| aml05206 | MicroRNAs in cancer [PATH:aml05206] | 4.01E-04 | 9.28E-03 | 8.72E-03 | ENSAMEG00000009662/ENSAMEG00000010721/ENSAMEG00000014201/ENSAMEG00000002791/ENSAMEG00000016520/ENSAMEG00000009668/ENSAMEG00000008899/ENSAMEG00000001398/ENSAMEG00000011823/ENSAMEG00000013940/ENSAMEG00000010298/ENSAMEG00000005590/ENSAMEG00000005226/ENSAMEG00000009575/ENSAMEG00000014704/ENSAMEG00000016250/ENSAMEG00000007608/ENSAMEG00000017811/ENSAMEG00000013257/ENSAMEG00000018326/ENSAMEG00000010799/ENSAMEG00000010769/ENSAMEG00000008705 | 23 |
| aml04115 | p53 signaling pathway [PATH:aml04115] | 5.69E-04 | 1.22E-02 | 1.14E-02 | ENSAMEG00000003117/ENSAMEG00000011372/ENSAMEG00000004603/ENSAMEG00000009668/ENSAMEG00000003329/ENSAMEG00000005590/ENSAMEG00000008484/ENSAMEG00000013018/ENSAMEG00000017811/ENSAMEG00000003168/ENSAMEG00000009274/ENSAMEG00000011413/ENSAMEG00000013710 | 13 |
| aml04120 | Ubiquitin mediated proteolysis [PATH:aml04120] | 7.12E-04 | 1.41E-02 | 1.33E-02 | ENSAMEG00000012547/ENSAMEG00000010307/ENSAMEG00000001637/ENSAMEG00000016275/ENSAMEG00000002389/ENSAMEG00000017610/ENSAMEG00000004224/ENSAMEG00000016483/ENSAMEG00000003779/ENSAMEG00000011651/ENSAMEG00000005634/ENSAMEG00000012193/ENSAMEG00000015826/ENSAMEG00000001353/ENSAMEG00000017319/ENSAMEG00000016518/ENSAMEG00000014025/ENSAMEG00000008551/ENSAMEG00000016602/ENSAMEG00000008807/ENSAMEG00000010104 | 21 |
| aml03410 | Base excision repair [PATH:aml03410] | 8.28E-04 | 1.53E-02 | 1.44E-02 | ENSAMEG00000003715/ENSAMEG00000015091/ENSAMEG00000013454/ENSAMEG00000009547/ENSAMEG00000003608/ENSAMEG00000017155/ENSAMEG00000013111/ENSAMEG00000019971/ENSAMEG00000009089 | 9 |
| aml05202 | Transcriptional misregulation in cancer [PATH:aml05202] | 1.96E-03 | 3.40E-02 | 3.20E-02 | ENSAMEG00000006109/ENSAMEG00000003337/ENSAMEG00000012594/ENSAMEG00000014221/ENSAMEG00000002934/ENSAMEG00000005436/ENSAMEG00000005341/ENSAMEG00000007748/ENSAMEG00000017674/ENSAMEG00000017478/ENSAMEG00000010056/ENSAMEG00000002954/ENSAMEG00000007383/ENSAMEG00000018575/ENSAMEG00000007625/ENSAMEG00000015779/ENSAMEG00000007608/ENSAMEG00000010127/ENSAMEG00000008231/ENSAMEG00000006241/ENSAMEG00000007181/ENSAMEG00000010769/ENSAMEG00000013710 | 23 |
