# Supplementary material for: Gene expression profiles during postnatal development of the liver and pancreas in giant pandas
Source: Aging (Albany NY). 2020 Aug 15;12(15):15705–29. doi: 10.18632/aging.103783 (PMC7467380; doi:10.18632/aging.103783)
Supplement: Supplementary Table 6 [file aging-12-103783-s004..docx]

**Supplementary Table 6. Significantly enriched GO categories for up-regulated DEGs in liver adult group compared with liver no feeding group.**

| **ID** | **Description** | **pvalue** | **p.adjust** | **qvalue** | **geneID** | **Count** |
| --- | --- | --- | --- | --- | --- | --- |
| GO:0005506 | iron ion binding | 1.06E-12 | 1.43E-09 | 1.35E-09 | ENSAMEG00000001708/ENSAMEG00000011960/ENSAMEG00000011824/ENSAMEG00000006243/ENSAMEG00000008842/ENSAMEG00000002179/ENSAMEG00000005138/ENSAMEG00000017058/ENSAMEG00000004376/ENSAMEG00000013900/ENSAMEG00000016104/ENSAMEG00000002316/ENSAMEG00000002714/ENSAMEG00000003107/ENSAMEG00000000342/ENSAMEG00000020100/ENSAMEG00000011816/ENSAMEG00000005596/ENSAMEG00000008222/ENSAMEG00000018432/ENSAMEG00000000726/ENSAMEG00000005633/ENSAMEG00000005288/ENSAMEG00000013650/ENSAMEG00000006439/ENSAMEG00000013578 | 26 |
| GO:0016705 | oxidoreductase activity, acting on paired donors, with incorporation or reduction of molecular oxygen | 7.68E-11 | 4.10E-08 | 3.87E-08 | ENSAMEG00000001708/ENSAMEG00000011960/ENSAMEG00000011824/ENSAMEG00000006243/ENSAMEG00000008842/ENSAMEG00000005138/ENSAMEG00000004376/ENSAMEG00000016104/ENSAMEG00000002316/ENSAMEG00000002714/ENSAMEG00000003107/ENSAMEG00000000342/ENSAMEG00000011816/ENSAMEG00000000726/ENSAMEG00000005288/ENSAMEG00000013650/ENSAMEG00000006439/ENSAMEG00000013578 | 18 |
| GO:0032981 | mitochondrial respiratory chain complex I assembly | 9.09E-11 | 4.10E-08 | 3.87E-08 | ENSAMEG00000000169/ENSAMEG00000008700/ENSAMEG00000004761/ENSAMEG00000010344/ENSAMEG00000015328/ENSAMEG00000009377/ENSAMEG00000011040/ENSAMEG00000000613/ENSAMEG00000014837/ENSAMEG00000005574/ENSAMEG00000006027/ENSAMEG00000010954/ENSAMEG00000003836/ENSAMEG00000004512 | 14 |
| GO:0006955 | immune response | 1.44E-10 | 4.88E-08 | 4.60E-08 | ENSAMEG00000010324/ENSAMEG00000004654/ENSAMEG00000002390/ENSAMEG00000002352/ENSAMEG00000002099/ENSAMEG00000002342/ENSAMEG00000007881/ENSAMEG00000002361/ENSAMEG00000014902/ENSAMEG00000015230/ENSAMEG00000004830/ENSAMEG00000001952/ENSAMEG00000001714/ENSAMEG00000015352/ENSAMEG00000016736/ENSAMEG00000005181/ENSAMEG00000019126/ENSAMEG00000001944/ENSAMEG00000005780/ENSAMEG00000010733/ENSAMEG00000017094/ENSAMEG00000016729/ENSAMEG00000016224/ENSAMEG00000008654/ENSAMEG00000007963/ENSAMEG00000012447/ENSAMEG00000015402/ENSAMEG00000017754 | 28 |
| GO:0042613 | MHC class II protein complex | 8.57E-09 | 2.32E-06 | 2.19E-06 | ENSAMEG00000004654/ENSAMEG00000002390/ENSAMEG00000002352/ENSAMEG00000002099/ENSAMEG00000002342/ENSAMEG00000002361/ENSAMEG00000001952/ENSAMEG00000001944 | 8 |
| GO:0016712 | oxidoreductase activity, acting on paired donors, with incorporation or reduction of molecular oxygen, reduced flavin or flavoprotein as one donor, and incorporation of one atom of oxygen | 2.08E-08 | 4.08E-06 | 3.85E-06 | ENSAMEG00000011960/ENSAMEG00000011824/ENSAMEG00000008842/ENSAMEG00000004376/ENSAMEG00000002316/ENSAMEG00000003107/ENSAMEG00000000342/ENSAMEG00000011816/ENSAMEG00000005288 | 9 |
| GO:0042101 | T cell receptor complex | 2.11E-08 | 4.08E-06 | 3.85E-06 | ENSAMEG00000013166/ENSAMEG00000013155/ENSAMEG00000004919/ENSAMEG00000007493/ENSAMEG00000000022/ENSAMEG00000005181/ENSAMEG00000010191 | 7 |
| GO:0002376 | immune system process | 3.15E-08 | 5.33E-06 | 5.03E-06 | ENSAMEG00000002390/ENSAMEG00000002352/ENSAMEG00000002099/ENSAMEG00000002342/ENSAMEG00000002361/ENSAMEG00000001952/ENSAMEG00000001714/ENSAMEG00000018451/ENSAMEG00000001961/ENSAMEG00000019126/ENSAMEG00000001944 | 11 |
| GO:0020037 | heme binding | 3.62E-08 | 5.44E-06 | 5.14E-06 | ENSAMEG00000001708/ENSAMEG00000011960/ENSAMEG00000015532/ENSAMEG00000011824/ENSAMEG00000006243/ENSAMEG00000008842/ENSAMEG00000013614/ENSAMEG00000005138/ENSAMEG00000004376/ENSAMEG00000001013/ENSAMEG00000016104/ENSAMEG00000002316/ENSAMEG00000002714/ENSAMEG00000003107/ENSAMEG00000000342/ENSAMEG00000011816/ENSAMEG00000005288/ENSAMEG00000005154/ENSAMEG00000013578 | 19 |
| GO:0005747 | mitochondrial respiratory chain complex I | 4.22E-08 | 5.72E-06 | 5.39E-06 | ENSAMEG00000000169/ENSAMEG00000004761/ENSAMEG00000010344/ENSAMEG00000015328/ENSAMEG00000002294/ENSAMEG00000011040/ENSAMEG00000000613/ENSAMEG00000014837/ENSAMEG00000006027/ENSAMEG00000010954/ENSAMEG00000003836/ENSAMEG00000004512 | 12 |
| GO:0002250 | adaptive immune response | 5.92E-08 | 7.28E-06 | 6.87E-06 | ENSAMEG00000002390/ENSAMEG00000005863/ENSAMEG00000002352/ENSAMEG00000002099/ENSAMEG00000002342/ENSAMEG00000002361/ENSAMEG00000001952/ENSAMEG00000004883/ENSAMEG00000005998/ENSAMEG00000001944 | 10 |
| GO:0002504 | antigen processing and presentation of peptide or polysaccharide antigen via MHC class II | 1.30E-07 | 1.36E-05 | 1.28E-05 | ENSAMEG00000002390/ENSAMEG00000002352/ENSAMEG00000002099/ENSAMEG00000002342/ENSAMEG00000002361/ENSAMEG00000001952/ENSAMEG00000001944 | 7 |
| GO:0019882 | antigen processing and presentation | 1.30E-07 | 1.36E-05 | 1.28E-05 | ENSAMEG00000004654/ENSAMEG00000002390/ENSAMEG00000002352/ENSAMEG00000002099/ENSAMEG00000002342/ENSAMEG00000002361/ENSAMEG00000001952/ENSAMEG00000001714/ENSAMEG00000002026/ENSAMEG00000001944 | 10 |
| GO:0004497 | monooxygenase activity | 2.53E-07 | 2.45E-05 | 2.31E-05 | ENSAMEG00000001708/ENSAMEG00000011960/ENSAMEG00000011824/ENSAMEG00000006243/ENSAMEG00000008842/ENSAMEG00000002179/ENSAMEG00000005138/ENSAMEG00000004376/ENSAMEG00000016104/ENSAMEG00000002316/ENSAMEG00000002714/ENSAMEG00000000342/ENSAMEG00000011816/ENSAMEG00000013578 | 14 |
| GO:0016491 | oxidoreductase activity | 3.12E-06 | 2.81E-04 | 2.66E-04 | ENSAMEG00000009706/ENSAMEG00000009705/ENSAMEG00000011960/ENSAMEG00000014033/ENSAMEG00000011824/ENSAMEG00000006243/ENSAMEG00000008842/ENSAMEG00000017964/ENSAMEG00000014144/ENSAMEG00000018686/ENSAMEG00000005138/ENSAMEG00000017058/ENSAMEG00000003452/ENSAMEG00000004376/ENSAMEG00000013900/ENSAMEG00000016104/ENSAMEG00000017356/ENSAMEG00000005398/ENSAMEG00000002714/ENSAMEG00000000342/ENSAMEG00000020100/ENSAMEG00000011816/ENSAMEG00000005596/ENSAMEG00000008222/ENSAMEG00000004853/ENSAMEG00000018432/ENSAMEG00000000726/ENSAMEG00000017675/ENSAMEG00000005633/ENSAMEG00000005535/ENSAMEG00000003442/ENSAMEG00000004236/ENSAMEG00000018748/ENSAMEG00000005154/ENSAMEG00000013650/ENSAMEG00000006439 | 36 |
| GO:0004364 | glutathione transferase activity | 2.69E-05 | 2.14E-03 | 2.02E-03 | ENSAMEG00000005027/ENSAMEG00000005016/ENSAMEG00000004970/ENSAMEG00000004985/ENSAMEG00000010971/ENSAMEG00000003550 | 6 |
| GO:0010596 | negative regulation of endothelial cell migration | 2.69E-05 | 2.14E-03 | 2.02E-03 | ENSAMEG00000017643/ENSAMEG00000016821/ENSAMEG00000012740/ENSAMEG00000004377/ENSAMEG00000006196/ENSAMEG00000002851 | 6 |
| GO:0015020 | glucuronosyltransferase activity | 1.14E-04 | 8.60E-03 | 8.12E-03 | ENSAMEG00000002540/ENSAMEG00000011718/ENSAMEG00000011749/ENSAMEG00000005730/ENSAMEG00000012397 | 5 |
| GO:0030247 | polysaccharide binding | 1.72E-04 | 1.22E-02 | 1.16E-02 | ENSAMEG00000010324/ENSAMEG00000002390/ENSAMEG00000008566/ENSAMEG00000015402/ENSAMEG00000017754 | 5 |
| GO:0008610 | lipid biosynthetic process | 2.49E-04 | 1.69E-02 | 1.59E-02 | ENSAMEG00000013900/ENSAMEG00000020100/ENSAMEG00000005596/ENSAMEG00000008222/ENSAMEG00000008506 | 5 |
| GO:0001666 | response to hypoxia | 3.05E-04 | 1.97E-02 | 1.85E-02 | ENSAMEG00000002179/ENSAMEG00000019096/ENSAMEG00000007445/ENSAMEG00000005307/ENSAMEG00000010258/ENSAMEG00000017263/ENSAMEG00000004179/ENSAMEG00000005154/ENSAMEG00000013650/ENSAMEG00000006439 | 10 |
| GO:0001540 | amyloid-beta binding | 3.28E-04 | 2.02E-02 | 1.90E-02 | ENSAMEG00000004654/ENSAMEG00000018451/ENSAMEG00000000141/ENSAMEG00000012740/ENSAMEG00000009675/ENSAMEG00000016224/ENSAMEG00000004179 | 7 |
| GO:0044344 | cellular response to fibroblast growth factor stimulus | 3.50E-04 | 2.06E-02 | 1.94E-02 | ENSAMEG00000014902/ENSAMEG00000004830/ENSAMEG00000016821/ENSAMEG00000008499/ENSAMEG00000005780 | 5 |
| GO:0045333 | cellular respiration | 4.92E-04 | 2.77E-02 | 2.62E-02 | ENSAMEG00000008842/ENSAMEG00000017776/ENSAMEG00000004358/ENSAMEG00000000613 | 4 |
| GO:0005044 | scavenger receptor activity | 5.20E-04 | 2.82E-02 | 2.66E-02 | ENSAMEG00000010324/ENSAMEG00000016126/ENSAMEG00000007493/ENSAMEG00000000074/ENSAMEG00000004457/ENSAMEG00000016224/ENSAMEG00000015402/ENSAMEG00000017754 | 8 |
| GO:0009986 | cell surface | 6.03E-04 | 3.12E-02 | 2.94E-02 | ENSAMEG00000004654/ENSAMEG00000002390/ENSAMEG00000015921/ENSAMEG00000017869/ENSAMEG00000018451/ENSAMEG00000005502/ENSAMEG00000000141/ENSAMEG00000004244/ENSAMEG00000001013/ENSAMEG00000004883/ENSAMEG00000009222/ENSAMEG00000017910/ENSAMEG00000011169/ENSAMEG00000016910/ENSAMEG00000004457/ENSAMEG00000011988/ENSAMEG00000005307/ENSAMEG00000001944/ENSAMEG00000008355/ENSAMEG00000005401/ENSAMEG00000001184/ENSAMEG00000016144/ENSAMEG00000016224/ENSAMEG00000005612/ENSAMEG00000019346/ENSAMEG00000016143 | 26 |
| GO:0030246 | carbohydrate binding | 6.30E-04 | 3.12E-02 | 2.94E-02 | ENSAMEG00000010014/ENSAMEG00000015088/ENSAMEG00000008566/ENSAMEG00000004634/ENSAMEG00000011948/ENSAMEG00000008580/ENSAMEG00000011988/ENSAMEG00000009716/ENSAMEG00000012137/ENSAMEG00000014065/ENSAMEG00000009675/ENSAMEG00000015775 | 12 |
| GO:0022900 | electron transport chain | 6.46E-04 | 3.12E-02 | 2.94E-02 | ENSAMEG00000009142/ENSAMEG00000013441/ENSAMEG00000017762/ENSAMEG00000015878/ENSAMEG00000000613/ENSAMEG00000006027/ENSAMEG00000014360/ENSAMEG00000005633/ENSAMEG00000006153 | 9 |
| GO:0005762 | mitochondrial large ribosomal subunit | 7.00E-04 | 3.23E-02 | 3.04E-02 | ENSAMEG00000010483/ENSAMEG00000009832/ENSAMEG00000001164/ENSAMEG00000003483/ENSAMEG00000000650/ENSAMEG00000011735/ENSAMEG00000011876/ENSAMEG00000014954 | 8 |
| GO:0010951 | negative regulation of endopeptidase activity | 7.15E-04 | 3.23E-02 | 3.04E-02 | ENSAMEG00000016366/ENSAMEG00000017742/ENSAMEG00000004046/ENSAMEG00000001013/ENSAMEG00000000456/ENSAMEG00000010076/ENSAMEG00000002621/ENSAMEG00000014526/ENSAMEG00000004316/ENSAMEG00000011447/ENSAMEG00000001045 | 11 |
| GO:0034364 | high-density lipoprotein particle | 7.48E-04 | 3.27E-02 | 3.08E-02 | ENSAMEG00000010218/ENSAMEG00000008681/ENSAMEG00000012740/ENSAMEG00000012737 | 4 |
| GO:0051537 | 2 iron, 2 sulfur cluster binding | 8.41E-04 | 3.56E-02 | 3.36E-02 | ENSAMEG00000009142/ENSAMEG00000010276/ENSAMEG00000006915/ENSAMEG00000004397/ENSAMEG00000005633 | 5 |
| GO:0031668 | cellular response to extracellular stimulus | 1.09E-03 | 4.31E-02 | 4.06E-02 | ENSAMEG00000000266/ENSAMEG00000009809/ENSAMEG00000018335/ENSAMEG00000007545 | 4 |
| GO:0046940 | nucleoside monophosphate phosphorylation | 1.09E-03 | 4.31E-02 | 4.06E-02 | ENSAMEG00000014118/ENSAMEG00000015149/ENSAMEG00000000012/ENSAMEG00000003223 | 4 |
| GO:0071346 | cellular response to interferon-gamma | 1.11E-03 | 4.31E-02 | 4.06E-02 | ENSAMEG00000014902/ENSAMEG00000018451/ENSAMEG00000017030/ENSAMEG00000011008/ENSAMEG00000005780/ENSAMEG00000005154/ENSAMEG00000007452 | 7 |
