# Supplementary material for: Gene expression profiles during postnatal development of the liver and pancreas in giant pandas
Source: Aging (Albany NY). 2020 Aug 15;12(15):15705–29. doi: 10.18632/aging.103783 (PMC7467380; doi:10.18632/aging.103783)
Supplement: Supplementary Table 7 [file aging-12-103783-s006..docx]

**Supplementary Table 7. Significantly enriched KEGG pathways for up-regulated DEGs in liver adult group compared with liver no feeding group.**

| **ID** | **Description** | **pvalue** | **p.adjust** | **qvalue** | **geneID** | **Count** |
| --- | --- | --- | --- | --- | --- | --- |
| aml05204 | Chemical carcinogenesis [PATH:aml05204] | 4.26E-14 | 1.16E-11 | 9.50E-12 | ENSAMEG00000005027/ENSAMEG00000011960/ENSAMEG00000015532/ENSAMEG00000011824/ENSAMEG00000006243/ENSAMEG00000008842/ENSAMEG00000005016/ENSAMEG00000011718/ENSAMEG00000004376/ENSAMEG00000004970/ENSAMEG00000016104/ENSAMEG00000016142/ENSAMEG00000005398/ENSAMEG00000015358/ENSAMEG00000003107/ENSAMEG00000011749/ENSAMEG00000004985/ENSAMEG00000005730/ENSAMEG00000010971/ENSAMEG00000003550 | 20 |
| aml05332 | Graft-versus-host disease [PATH:aml05332] | 1.83E-09 | 2.49E-07 | 2.05E-07 | ENSAMEG00000002390/ENSAMEG00000002352/ENSAMEG00000002099/ENSAMEG00000002342/ENSAMEG00000001952/ENSAMEG00000002302/ENSAMEG00000004634/ENSAMEG00000001714/ENSAMEG00000007201/ENSAMEG00000001944/ENSAMEG00000010733 | 11 |
| aml00830 | Retinol metabolism [PATH:aml00830] | 2.49E-08 | 2.25E-06 | 1.85E-06 | ENSAMEG00000011824/ENSAMEG00000006243/ENSAMEG00000008842/ENSAMEG00000011718/ENSAMEG00000004376/ENSAMEG00000016104/ENSAMEG00000003107/ENSAMEG00000011749/ENSAMEG00000005730/ENSAMEG00000011816/ENSAMEG00000012515/ENSAMEG00000005633/ENSAMEG00000004236 | 13 |
| aml04940 | Type I diabetes mellitus [PATH:aml04940] | 6.55E-08 | 4.23E-06 | 3.47E-06 | ENSAMEG00000002390/ENSAMEG00000002352/ENSAMEG00000002099/ENSAMEG00000002342/ENSAMEG00000001952/ENSAMEG00000002302/ENSAMEG00000001714/ENSAMEG00000005123/ENSAMEG00000007201/ENSAMEG00000001944/ENSAMEG00000010733 | 11 |
| aml00190 | Oxidative phosphorylation [PATH:aml00190] | 8.46E-08 | 4.23E-06 | 3.47E-06 | ENSAMEG00000000169/ENSAMEG00000004785/ENSAMEG00000004761/ENSAMEG00000003731/ENSAMEG00000013441/ENSAMEG00000017762/ENSAMEG00000011283/ENSAMEG00000010344/ENSAMEG00000007142/ENSAMEG00000015328/ENSAMEG00000002294/ENSAMEG00000002182/ENSAMEG00000011040/ENSAMEG00000014690/ENSAMEG00000007287/ENSAMEG00000000613/ENSAMEG00000006027/ENSAMEG00000010954/ENSAMEG00000003836/ENSAMEG00000006153/ENSAMEG00000004512 | 21 |
| aml00980 | Metabolism of xenobiotics by cytochrome P450 [PATH:aml00980] | 9.33E-08 | 4.23E-06 | 3.47E-06 | ENSAMEG00000005027/ENSAMEG00000011960/ENSAMEG00000008842/ENSAMEG00000005016/ENSAMEG00000011718/ENSAMEG00000004970/ENSAMEG00000005398/ENSAMEG00000011749/ENSAMEG00000004985/ENSAMEG00000005730/ENSAMEG00000011816/ENSAMEG00000010971/ENSAMEG00000003550 | 13 |
| aml04932 | Non-alcoholic fatty liver disease (NAFLD) [PATH:aml04932] | 2.96E-07 | 1.15E-05 | 9.44E-06 | ENSAMEG00000019175/ENSAMEG00000004830/ENSAMEG00000000169/ENSAMEG00000004785/ENSAMEG00000004761/ENSAMEG00000003731/ENSAMEG00000013441/ENSAMEG00000017762/ENSAMEG00000010344/ENSAMEG00000015328/ENSAMEG00000010733/ENSAMEG00000002294/ENSAMEG00000002182/ENSAMEG00000011040/ENSAMEG00000007287/ENSAMEG00000000613/ENSAMEG00000006027/ENSAMEG00000010954/ENSAMEG00000003836/ENSAMEG00000006153/ENSAMEG00000004512 | 21 |
| aml05330 | Allograft rejection [PATH:aml05330] | 7.87E-07 | 2.68E-05 | 2.20E-05 | ENSAMEG00000002390/ENSAMEG00000002352/ENSAMEG00000002099/ENSAMEG00000002342/ENSAMEG00000001952/ENSAMEG00000002302/ENSAMEG00000001714/ENSAMEG00000007201/ENSAMEG00000001944 | 9 |
| aml04610 | Complement and coagulation cascades [PATH:aml04610] | 9.08E-07 | 2.75E-05 | 2.25E-05 | ENSAMEG00000015230/ENSAMEG00000017524/ENSAMEG00000003480/ENSAMEG00000000141/ENSAMEG00000015352/ENSAMEG00000005307/ENSAMEG00000009692/ENSAMEG00000002621/ENSAMEG00000017094/ENSAMEG00000005097/ENSAMEG00000014526/ENSAMEG00000013668/ENSAMEG00000003516/ENSAMEG00000013566/ENSAMEG00000001045 | 15 |
| aml05322 | Systemic lupus erythematosus [PATH:aml05322] | 1.25E-06 | 3.40E-05 | 2.79E-05 | ENSAMEG00000002390/ENSAMEG00000002352/ENSAMEG00000002099/ENSAMEG00000002342/ENSAMEG00000015230/ENSAMEG00000001952/ENSAMEG00000015352/ENSAMEG00000019926/ENSAMEG00000019745/ENSAMEG00000007201/ENSAMEG00000019689/ENSAMEG00000001944/ENSAMEG00000017094/ENSAMEG00000013668/ENSAMEG00000019305/ENSAMEG00000013566/ENSAMEG00000001045 | 17 |
| aml04658 | Th1 and Th2 cell differentiation [PATH:aml04658] | 1.63E-06 | 4.04E-05 | 3.31E-05 | ENSAMEG00000002390/ENSAMEG00000002352/ENSAMEG00000002099/ENSAMEG00000002342/ENSAMEG00000007672/ENSAMEG00000013155/ENSAMEG00000013141/ENSAMEG00000001952/ENSAMEG00000011008/ENSAMEG00000016821/ENSAMEG00000005181/ENSAMEG00000018335/ENSAMEG00000001944/ENSAMEG00000003134/ENSAMEG00000004069 | 15 |
| aml00140 | Steroid hormone biosynthesis [PATH:aml00140] | 2.02E-06 | 4.57E-05 | 3.75E-05 | ENSAMEG00000011824/ENSAMEG00000006243/ENSAMEG00000008842/ENSAMEG00000011718/ENSAMEG00000004376/ENSAMEG00000016104/ENSAMEG00000003107/ENSAMEG00000011749/ENSAMEG00000005730/ENSAMEG00000004853/ENSAMEG00000004236 | 11 |
| aml04612 | Antigen processing and presentation [PATH:aml04612] | 2.21E-06 | 4.62E-05 | 3.79E-05 | ENSAMEG00000004654/ENSAMEG00000002390/ENSAMEG00000002352/ENSAMEG00000002099/ENSAMEG00000002342/ENSAMEG00000001952/ENSAMEG00000002302/ENSAMEG00000004634/ENSAMEG00000001714/ENSAMEG00000001976/ENSAMEG00000001944/ENSAMEG00000016394 | 12 |
| aml00982 | Drug metabolism - cytochrome P450 [PATH:aml00982] | 2.66E-06 | 5.17E-05 | 4.24E-05 | ENSAMEG00000005027/ENSAMEG00000008842/ENSAMEG00000005016/ENSAMEG00000011718/ENSAMEG00000004970/ENSAMEG00000011749/ENSAMEG00000004985/ENSAMEG00000005730/ENSAMEG00000010971/ENSAMEG00000005633/ENSAMEG00000003550 | 11 |
| aml04659 | Th17 cell differentiation [PATH:aml04659] | 5.07E-06 | 9.20E-05 | 7.55E-05 | ENSAMEG00000002390/ENSAMEG00000002352/ENSAMEG00000002099/ENSAMEG00000002342/ENSAMEG00000007672/ENSAMEG00000013155/ENSAMEG00000013141/ENSAMEG00000001952/ENSAMEG00000011008/ENSAMEG00000005181/ENSAMEG00000018335/ENSAMEG00000001944/ENSAMEG00000010733/ENSAMEG00000003134/ENSAMEG00000011163/ENSAMEG00000004069 | 16 |
| aml05320 | Autoimmune thyroid disease [PATH:aml05320] | 7.38E-06 | 1.25E-04 | 1.03E-04 | ENSAMEG00000002390/ENSAMEG00000002352/ENSAMEG00000002099/ENSAMEG00000002342/ENSAMEG00000001952/ENSAMEG00000002302/ENSAMEG00000001714/ENSAMEG00000007201/ENSAMEG00000001944 | 9 |
| aml00983 | Drug metabolism - other enzymes [PATH:aml00983] | 8.58E-06 | 1.37E-04 | 1.13E-04 | ENSAMEG00000005027/ENSAMEG00000005016/ENSAMEG00000004732/ENSAMEG00000011718/ENSAMEG00000004970/ENSAMEG00000011749/ENSAMEG00000004985/ENSAMEG00000005730/ENSAMEG00000000012/ENSAMEG00000010971/ENSAMEG00000003550/ENSAMEG00000008351 | 12 |
| aml00591 | Linoleic acid metabolism [PATH:aml00591] | 1.33E-05 | 2.01E-04 | 1.65E-04 | ENSAMEG00000006243/ENSAMEG00000008842/ENSAMEG00000004376/ENSAMEG00000016104/ENSAMEG00000003107/ENSAMEG00000000342/ENSAMEG00000018150/ENSAMEG00000003219 | 8 |
| aml00590 | Arachidonic acid metabolism [PATH:aml00590] | 2.23E-05 | 3.11E-04 | 2.55E-04 | ENSAMEG00000015532/ENSAMEG00000011824/ENSAMEG00000006243/ENSAMEG00000013340/ENSAMEG00000016104/ENSAMEG00000005398/ENSAMEG00000003107/ENSAMEG00000000342/ENSAMEG00000018150/ENSAMEG00000003219/ENSAMEG00000013578 | 11 |
| aml05150 | Staphylococcus aureus infection [PATH:aml05150] | 2.29E-05 | 3.11E-04 | 2.55E-04 | ENSAMEG00000002390/ENSAMEG00000002352/ENSAMEG00000002099/ENSAMEG00000002342/ENSAMEG00000001952/ENSAMEG00000001682/ENSAMEG00000005526/ENSAMEG00000001944/ENSAMEG00000008170/ENSAMEG00000013668/ENSAMEG00000013566/ENSAMEG00000001045 | 12 |
| aml05323 | Rheumatoid arthritis [PATH:aml05323] | 6.51E-05 | 8.43E-04 | 6.92E-04 | ENSAMEG00000002390/ENSAMEG00000002352/ENSAMEG00000002099/ENSAMEG00000002342/ENSAMEG00000014902/ENSAMEG00000004830/ENSAMEG00000001952/ENSAMEG00000018335/ENSAMEG00000007201/ENSAMEG00000001944/ENSAMEG00000011283/ENSAMEG00000005780/ENSAMEG00000010733 | 13 |
| aml05321 | Inflammatory bowel disease (IBD) [PATH:aml05321] | 1.78E-04 | 2.20E-03 | 1.80E-03 | ENSAMEG00000002390/ENSAMEG00000002352/ENSAMEG00000002099/ENSAMEG00000002342/ENSAMEG00000001952/ENSAMEG00000011008/ENSAMEG00000001944/ENSAMEG00000010733/ENSAMEG00000011163/ENSAMEG00000004069 | 10 |
| aml04714 | Thermogenesis [PATH:aml04714] | 2.21E-04 | 2.61E-03 | 2.14E-03 | ENSAMEG00000000169/ENSAMEG00000004785/ENSAMEG00000004761/ENSAMEG00000003731/ENSAMEG00000013441/ENSAMEG00000017762/ENSAMEG00000010344/ENSAMEG00000007142/ENSAMEG00000015328/ENSAMEG00000002294/ENSAMEG00000002182/ENSAMEG00000009377/ENSAMEG00000011040/ENSAMEG00000014690/ENSAMEG00000007287/ENSAMEG00000000613/ENSAMEG00000006027/ENSAMEG00000010954/ENSAMEG00000003836/ENSAMEG00000008506/ENSAMEG00000006153/ENSAMEG00000004512 | 22 |
| aml05133 | Pertussis [PATH:aml05133] | 2.51E-04 | 2.75E-03 | 2.26E-03 | ENSAMEG00000004830/ENSAMEG00000003480/ENSAMEG00000018335/ENSAMEG00000015508/ENSAMEG00000010733/ENSAMEG00000014526/ENSAMEG00000013668/ENSAMEG00000003516/ENSAMEG00000013566/ENSAMEG00000001045/ENSAMEG00000005154 | 11 |
| aml04514 | Cell adhesion molecules (CAMs) [PATH:aml04514] | 2.60E-04 | 2.75E-03 | 2.26E-03 | ENSAMEG00000002390/ENSAMEG00000002352/ENSAMEG00000002099/ENSAMEG00000002342/ENSAMEG00000007493/ENSAMEG00000001952/ENSAMEG00000002302/ENSAMEG00000001714/ENSAMEG00000019096/ENSAMEG00000007342/ENSAMEG00000007201/ENSAMEG00000008621/ENSAMEG00000001944/ENSAMEG00000018735/ENSAMEG00000019949 | 15 |
| aml05416 | Viral myocarditis [PATH:aml05416] | 2.68E-04 | 2.75E-03 | 2.26E-03 | ENSAMEG00000002390/ENSAMEG00000002352/ENSAMEG00000002099/ENSAMEG00000002342/ENSAMEG00000001952/ENSAMEG00000002302/ENSAMEG00000001714/ENSAMEG00000007201/ENSAMEG00000001944 | 9 |
| aml04640 | Hematopoietic cell lineage [PATH:aml04640] | 2.73E-04 | 2.75E-03 | 2.26E-03 | ENSAMEG00000002390/ENSAMEG00000002352/ENSAMEG00000002099/ENSAMEG00000002342/ENSAMEG00000013155/ENSAMEG00000013141/ENSAMEG00000001952/ENSAMEG00000001944/ENSAMEG00000010733/ENSAMEG00000014604/ENSAMEG00000016224/ENSAMEG00000004069 | 12 |
| aml04672 | Intestinal immune network for IgA production [PATH:aml04672] | 3.55E-04 | 3.45E-03 | 2.83E-03 | ENSAMEG00000002390/ENSAMEG00000002352/ENSAMEG00000002099/ENSAMEG00000002342/ENSAMEG00000001952/ENSAMEG00000007201/ENSAMEG00000001944 | 7 |
| aml05310 | Asthma [PATH:aml05310] | 4.34E-04 | 4.07E-03 | 3.34E-03 | ENSAMEG00000002390/ENSAMEG00000002352/ENSAMEG00000002099/ENSAMEG00000002342/ENSAMEG00000001952/ENSAMEG00000001944 | 6 |
| aml05140 | Leishmaniasis [PATH:aml05140] | 5.31E-04 | 4.82E-03 | 3.95E-03 | ENSAMEG00000002390/ENSAMEG00000002352/ENSAMEG00000002099/ENSAMEG00000015532/ENSAMEG00000002342/ENSAMEG00000001952/ENSAMEG00000018335/ENSAMEG00000001944/ENSAMEG00000010733/ENSAMEG00000005154 | 10 |
| aml04145 | Phagosome [PATH:aml04145] | 6.30E-04 | 5.53E-03 | 4.53E-03 | ENSAMEG00000002390/ENSAMEG00000002352/ENSAMEG00000002099/ENSAMEG00000002342/ENSAMEG00000001952/ENSAMEG00000002302/ENSAMEG00000001714/ENSAMEG00000001682/ENSAMEG00000013685/ENSAMEG00000001976/ENSAMEG00000008580/ENSAMEG00000001944/ENSAMEG00000011283/ENSAMEG00000016224/ENSAMEG00000013566 | 15 |
| aml05012 | Parkinson disease [PATH:aml05012] | 1.63E-03 | 1.38E-02 | 1.13E-02 | ENSAMEG00000002179/ENSAMEG00000014519/ENSAMEG00000000169/ENSAMEG00000004785/ENSAMEG00000004761/ENSAMEG00000003731/ENSAMEG00000013441/ENSAMEG00000003703/ENSAMEG00000017762/ENSAMEG00000010344/ENSAMEG00000015328/ENSAMEG00000002294/ENSAMEG00000002182/ENSAMEG00000011040/ENSAMEG00000007287/ENSAMEG00000000613/ENSAMEG00000006027/ENSAMEG00000010954/ENSAMEG00000003836/ENSAMEG00000006153/ENSAMEG00000004512 | 21 |
| aml04723 | Retrograde endocannabinoid signaling [PATH:aml04723] | 1.75E-03 | 1.44E-02 | 1.19E-02 | ENSAMEG00000015532/ENSAMEG00000004527/ENSAMEG00000000169/ENSAMEG00000004785/ENSAMEG00000004761/ENSAMEG00000010344/ENSAMEG00000015328/ENSAMEG00000002294/ENSAMEG00000002182/ENSAMEG00000011040/ENSAMEG00000000613/ENSAMEG00000006027/ENSAMEG00000010954/ENSAMEG00000003836/ENSAMEG00000004512 | 15 |
| aml05020 | Prion diseases [PATH:aml05020] | 2.71E-03 | 2.17E-02 | 1.78E-02 | ENSAMEG00000014902/ENSAMEG00000015230/ENSAMEG00000015352/ENSAMEG00000010733/ENSAMEG00000017094/ENSAMEG00000001045 | 6 |
| aml05168 | Herpes simplex virus 1 infection [PATH:aml05168] | 3.77E-03 | 2.93E-02 | 2.40E-02 | ENSAMEG00000004654/ENSAMEG00000002390/ENSAMEG00000002352/ENSAMEG00000002099/ENSAMEG00000002342/ENSAMEG00000014902/ENSAMEG00000019175/ENSAMEG00000001952/ENSAMEG00000002302/ENSAMEG00000001714/ENSAMEG00000010517/ENSAMEG00000001976/ENSAMEG00000010613/ENSAMEG00000001944/ENSAMEG00000005780/ENSAMEG00000010733/ENSAMEG00000014247/ENSAMEG00000008654/ENSAMEG00000001045 | 19 |
| aml05164 | Influenza A [PATH:aml05164] | 4.05E-03 | 3.06E-02 | 2.51E-02 | ENSAMEG00000002390/ENSAMEG00000002352/ENSAMEG00000002099/ENSAMEG00000002342/ENSAMEG00000014902/ENSAMEG00000019175/ENSAMEG00000004830/ENSAMEG00000001952/ENSAMEG00000010494/ENSAMEG00000010613/ENSAMEG00000001944/ENSAMEG00000005780/ENSAMEG00000010733/ENSAMEG00000008654 | 14 |
| aml05169 | Epstein-Barr virus infection [PATH:aml05169] | 4.30E-03 | 3.16E-02 | 2.59E-02 | ENSAMEG00000002390/ENSAMEG00000002352/ENSAMEG00000002099/ENSAMEG00000002342/ENSAMEG00000013155/ENSAMEG00000005608/ENSAMEG00000013141/ENSAMEG00000000266/ENSAMEG00000001952/ENSAMEG00000002302/ENSAMEG00000001714/ENSAMEG00000007357/ENSAMEG00000001976/ENSAMEG00000010613/ENSAMEG00000001944/ENSAMEG00000008654 | 16 |
| aml05166 | Human T-cell leukemia virus 1 infection [PATH:aml05166] | 4.82E-03 | 3.45E-02 | 2.83E-02 | ENSAMEG00000002390/ENSAMEG00000002352/ENSAMEG00000002099/ENSAMEG00000002342/ENSAMEG00000007672/ENSAMEG00000013155/ENSAMEG00000013141/ENSAMEG00000000266/ENSAMEG00000001952/ENSAMEG00000002302/ENSAMEG00000001714/ENSAMEG00000012308/ENSAMEG00000008499/ENSAMEG00000013093/ENSAMEG00000018335/ENSAMEG00000001944/ENSAMEG00000007545/ENSAMEG00000003134 | 18 |
| aml00053 | Ascorbate and aldarate metabolism [PATH:aml00053] | 5.46E-03 | 3.81E-02 | 3.12E-02 | ENSAMEG00000018686/ENSAMEG00000011718/ENSAMEG00000011749/ENSAMEG00000005730 | 4 |
| aml00480 | Glutathione metabolism [PATH:aml00480] | 5.77E-03 | 3.92E-02 | 3.22E-02 | ENSAMEG00000005027/ENSAMEG00000005016/ENSAMEG00000004970/ENSAMEG00000004985/ENSAMEG00000001748/ENSAMEG00000010971/ENSAMEG00000003550 | 7 |
| aml05010 | Alzheimer disease [PATH:aml05010] | 6.08E-03 | 4.04E-02 | 3.31E-02 | ENSAMEG00000015532/ENSAMEG00000000169/ENSAMEG00000004785/ENSAMEG00000004761/ENSAMEG00000003731/ENSAMEG00000013441/ENSAMEG00000012740/ENSAMEG00000000612/ENSAMEG00000017762/ENSAMEG00000010344/ENSAMEG00000015328/ENSAMEG00000010733/ENSAMEG00000002294/ENSAMEG00000001256/ENSAMEG00000002182/ENSAMEG00000011040/ENSAMEG00000003134/ENSAMEG00000007287/ENSAMEG00000000613/ENSAMEG00000006027/ENSAMEG00000010954/ENSAMEG00000003836/ENSAMEG00000005154/ENSAMEG00000006153/ENSAMEG00000004512 | 25 |
| aml05418 | Fluid shear stress and atherosclerosis [PATH:aml05418] | 7.32E-03 | 4.74E-02 | 3.89E-02 | ENSAMEG00000005027/ENSAMEG00000005016/ENSAMEG00000007357/ENSAMEG00000004970/ENSAMEG00000018335/ENSAMEG00000007342/ENSAMEG00000005307/ENSAMEG00000004985/ENSAMEG00000005780/ENSAMEG00000010733/ENSAMEG00000001347/ENSAMEG00000010971/ENSAMEG00000003550 | 13 |
