# Supplementary material for: Gene expression profiles during postnatal development of the liver and pancreas in giant pandas
Source: Aging (Albany NY). 2020 Aug 15;12(15):15705–29. doi: 10.18632/aging.103783 (PMC7467380; doi:10.18632/aging.103783)
Supplement: Supplementary Table 8 [file aging-12-103783-s005..docx]

**Supplementary Table 8. Significantly enriched GO categories for down-regulated DEGs in liver adult group compared with liver no feeding group.**

| **ID** | **Description** | **pvalue** | **p.adjust** | **qvalue** | **geneID** | **Count** |
| --- | --- | --- | --- | --- | --- | --- |
| GO:0006260 | DNA replication | 1.89E-17 | 3.36E-14 | 3.04E-14 | ENSAMEG00000014998/ENSAMEG00000001634/ENSAMEG00000005841/ENSAMEG00000018028/ENSAMEG00000005460/ENSAMEG00000009254/ENSAMEG00000014870/ENSAMEG00000013454/ENSAMEG00000013505/ENSAMEG00000018347/ENSAMEG00000018281/ENSAMEG00000015091/ENSAMEG00000009547/ENSAMEG00000008540/ENSAMEG00000013104/ENSAMEG00000000792/ENSAMEG00000014758/ENSAMEG00000005102/ENSAMEG00000011150/ENSAMEG00000013111/ENSAMEG00000013843/ENSAMEG00000017258/ENSAMEG00000002731/ENSAMEG00000001421/ENSAMEG00000009566/ENSAMEG00000010700/ENSAMEG00000017155/ENSAMEG00000000475/ENSAMEG00000000109/ENSAMEG00000017565/ENSAMEG00000011132/ENSAMEG00000008231 | 32 |
| GO:0000776 | kinetochore | 5.98E-15 | 5.31E-12 | 4.80E-12 | ENSAMEG00000015869/ENSAMEG00000017537/ENSAMEG00000014095/ENSAMEG00000009532/ENSAMEG00000006525/ENSAMEG00000010069/ENSAMEG00000012771/ENSAMEG00000009491/ENSAMEG00000002580/ENSAMEG00000005342/ENSAMEG00000000913/ENSAMEG00000001187/ENSAMEG00000017084/ENSAMEG00000014232/ENSAMEG00000017203/ENSAMEG00000014549/ENSAMEG00000016838/ENSAMEG00000009168/ENSAMEG00000006979/ENSAMEG00000014641/ENSAMEG00000000371/ENSAMEG00000005084/ENSAMEG00000005053/ENSAMEG00000003251/ENSAMEG00000005149/ENSAMEG00000012392/ENSAMEG00000008231/ENSAMEG00000014068/ENSAMEG00000001352 | 29 |
| GO:0007059 | chromosome segregation | 2.71E-13 | 1.26E-10 | 1.14E-10 | ENSAMEG00000015869/ENSAMEG00000017537/ENSAMEG00000007989/ENSAMEG00000016113/ENSAMEG00000010069/ENSAMEG00000009491/ENSAMEG00000000171/ENSAMEG00000000913/ENSAMEG00000001187/ENSAMEG00000006653/ENSAMEG00000017084/ENSAMEG00000013933/ENSAMEG00000000137/ENSAMEG00000010875/ENSAMEG00000016838/ENSAMEG00000012524/ENSAMEG00000014641/ENSAMEG00000000371/ENSAMEG00000012346/ENSAMEG00000001233/ENSAMEG00000011337/ENSAMEG00000000501 | 22 |
| GO:0000922 | spindle pole | 2.83E-13 | 1.26E-10 | 1.14E-10 | ENSAMEG00000015869/ENSAMEG00000014099/ENSAMEG00000017540/ENSAMEG00000013678/ENSAMEG00000014771/ENSAMEG00000010721/ENSAMEG00000000913/ENSAMEG00000001187/ENSAMEG00000013777/ENSAMEG00000014232/ENSAMEG00000000953/ENSAMEG00000004686/ENSAMEG00000010512/ENSAMEG00000004713/ENSAMEG00000010293/ENSAMEG00000006979/ENSAMEG00000007868/ENSAMEG00000002382/ENSAMEG00000000410/ENSAMEG00000001233/ENSAMEG00000009136/ENSAMEG00000019816/ENSAMEG00000001860/ENSAMEG00000010861/ENSAMEG00000012571/ENSAMEG00000012392 | 26 |
| GO:0005813 | centrosome | 2.16E-12 | 7.68E-10 | 6.94E-10 | ENSAMEG00000017945/ENSAMEG00000010767/ENSAMEG00000001259/ENSAMEG00000010721/ENSAMEG00000003520/ENSAMEG00000010069/ENSAMEG00000007168/ENSAMEG00000000013/ENSAMEG00000005225/ENSAMEG00000002675/ENSAMEG00000014201/ENSAMEG00000000913/ENSAMEG00000001187/ENSAMEG00000009390/ENSAMEG00000013840/ENSAMEG00000013455/ENSAMEG00000011443/ENSAMEG00000014232/ENSAMEG00000000953/ENSAMEG00000002666/ENSAMEG00000008991/ENSAMEG00000004603/ENSAMEG00000008931/ENSAMEG00000010512/ENSAMEG00000001549/ENSAMEG00000008540/ENSAMEG00000004713/ENSAMEG00000008648/ENSAMEG00000000503/ENSAMEG00000003270/ENSAMEG00000010293/ENSAMEG00000006979/ENSAMEG00000003215/ENSAMEG00000009369/ENSAMEG00000001250/ENSAMEG00000000983/ENSAMEG00000002382/ENSAMEG00000012346/ENSAMEG00000007412/ENSAMEG00000001121/ENSAMEG00000002590/ENSAMEG00000017740/ENSAMEG00000005084/ENSAMEG00000003586/ENSAMEG00000014294/ENSAMEG00000001233/ENSAMEG00000001855/ENSAMEG00000009136/ENSAMEG00000019816/ENSAMEG00000017370/ENSAMEG00000017365/ENSAMEG00000012848/ENSAMEG00000011164/ENSAMEG00000011827/ENSAMEG00000007026/ENSAMEG00000008333/ENSAMEG00000016790/ENSAMEG00000009815/ENSAMEG00000010861/ENSAMEG00000012571/ENSAMEG00000013304/ENSAMEG00000004426/ENSAMEG00000012392/ENSAMEG00000003208/ENSAMEG00000007023/ENSAMEG00000005486/ENSAMEG00000017216/ENSAMEG00000018345 | 68 |
| GO:0006270 | DNA replication initiation | 3.51E-11 | 1.04E-08 | 9.39E-09 | ENSAMEG00000014998/ENSAMEG00000001634/ENSAMEG00000005841/ENSAMEG00000011443/ENSAMEG00000013505/ENSAMEG00000009668/ENSAMEG00000008540/ENSAMEG00000010605/ENSAMEG00000007868/ENSAMEG00000011150/ENSAMEG00000001421/ENSAMEG00000005590 | 12 |
| GO:0000278 | mitotic cell cycle | 7.37E-11 | 1.87E-08 | 1.69E-08 | ENSAMEG00000015869/ENSAMEG00000017537/ENSAMEG00000014358/ENSAMEG00000007168/ENSAMEG00000009491/ENSAMEG00000014232/ENSAMEG00000000135/ENSAMEG00000007868/ENSAMEG00000000983/ENSAMEG00000014641/ENSAMEG00000000371/ENSAMEG00000012466/ENSAMEG00000012346/ENSAMEG00000003841/ENSAMEG00000001855/ENSAMEG00000009566/ENSAMEG00000017275/ENSAMEG00000009815/ENSAMEG00000013304/ENSAMEG00000016483/ENSAMEG00000013388 | 21 |
| GO:0008017 | microtubule binding | 1.66E-09 | 3.69E-07 | 3.33E-07 | ENSAMEG00000014095/ENSAMEG00000012038/ENSAMEG00000013667/ENSAMEG00000001458/ENSAMEG00000011957/ENSAMEG00000016762/ENSAMEG00000014099/ENSAMEG00000009205/ENSAMEG00000012611/ENSAMEG00000001093/ENSAMEG00000016438/ENSAMEG00000018397/ENSAMEG00000014201/ENSAMEG00000014825/ENSAMEG00000018442/ENSAMEG00000014232/ENSAMEG00000004686/ENSAMEG00000004598/ENSAMEG00000014549/ENSAMEG00000008648/ENSAMEG00000000293/ENSAMEG00000002382/ENSAMEG00000017728/ENSAMEG00000005084/ENSAMEG00000003586/ENSAMEG00000012351/ENSAMEG00000001233/ENSAMEG00000003475/ENSAMEG00000012232/ENSAMEG00000012848/ENSAMEG00000016790/ENSAMEG00000010861/ENSAMEG00000015215/ENSAMEG00000012239/ENSAMEG00000003927/ENSAMEG00000002951/ENSAMEG00000010788 | 37 |
| GO:0000724 | double-strand break repair via homologous recombination | 2.05E-09 | 4.05E-07 | 3.67E-07 | ENSAMEG00000005645/ENSAMEG00000000171/ENSAMEG00000009390/ENSAMEG00000013902/ENSAMEG00000016965/ENSAMEG00000002666/ENSAMEG00000015678/ENSAMEG00000002533/ENSAMEG00000010293/ENSAMEG00000010605/ENSAMEG00000014294/ENSAMEG00000001323/ENSAMEG00000010700/ENSAMEG00000013304/ENSAMEG00000016741/ENSAMEG00000016796/ENSAMEG00000004324/ENSAMEG00000011323/ENSAMEG00000011132 | 19 |
| GO:0032508 | DNA duplex unwinding | 5.37E-09 | 8.71E-07 | 7.87E-07 | ENSAMEG00000001634/ENSAMEG00000005841/ENSAMEG00000018028/ENSAMEG00000005460/ENSAMEG00000014870/ENSAMEG00000008540/ENSAMEG00000004713/ENSAMEG00000007469/ENSAMEG00000008785/ENSAMEG00000013843/ENSAMEG00000001421/ENSAMEG00000002148/ENSAMEG00000009566/ENSAMEG00000000475/ENSAMEG00000000109/ENSAMEG00000006307/ENSAMEG00000016233 | 17 |
| GO:0000281 | mitotic cytokinesis | 5.40E-09 | 8.71E-07 | 7.87E-07 | ENSAMEG00000015972/ENSAMEG00000016762/ENSAMEG00000009205/ENSAMEG00000002112/ENSAMEG00000001093/ENSAMEG00000016438/ENSAMEG00000014201/ENSAMEG00000009390/ENSAMEG00000001035/ENSAMEG00000013840/ENSAMEG00000014232/ENSAMEG00000016838/ENSAMEG00000010298/ENSAMEG00000014294/ENSAMEG00000002139/ENSAMEG00000011827 | 16 |
| GO:0005819 | spindle | 1.73E-08 | 2.57E-06 | 2.32E-06 | ENSAMEG00000015869/ENSAMEG00000013667/ENSAMEG00000016762/ENSAMEG00000014099/ENSAMEG00000013678/ENSAMEG00000016438/ENSAMEG00000014201/ENSAMEG00000014232/ENSAMEG00000004686/ENSAMEG00000001746/ENSAMEG00000016838/ENSAMEG00000009369/ENSAMEG00000001260/ENSAMEG00000002139/ENSAMEG00000003640/ENSAMEG00000012571/ENSAMEG00000016741 | 17 |
| GO:0006281 | DNA repair | 2.17E-08 | 2.97E-06 | 2.68E-06 | ENSAMEG00000013934/ENSAMEG00000003715/ENSAMEG00000011867/ENSAMEG00000014825/ENSAMEG00000000171/ENSAMEG00000009390/ENSAMEG00000012174/ENSAMEG00000013454/ENSAMEG00000016965/ENSAMEG00000002666/ENSAMEG00000011892/ENSAMEG00000015091/ENSAMEG00000015678/ENSAMEG00000003608/ENSAMEG00000016051/ENSAMEG00000001064/ENSAMEG00000013843/ENSAMEG00000012957/ENSAMEG00000005053/ENSAMEG00000009566/ENSAMEG00000010700/ENSAMEG00000019971/ENSAMEG00000000475/ENSAMEG00000003299/ENSAMEG00000012085/ENSAMEG00000013304/ENSAMEG00000016741/ENSAMEG00000016796/ENSAMEG00000004324/ENSAMEG00000011323/ENSAMEG00000011132 | 31 |
| GO:0000775 | chromosome, centromeric region | 2.98E-08 | 3.53E-06 | 3.19E-06 | ENSAMEG00000015869/ENSAMEG00000017537/ENSAMEG00000014095/ENSAMEG00000002112/ENSAMEG00000016113/ENSAMEG00000009491/ENSAMEG00000001082/ENSAMEG00000000913/ENSAMEG00000005435/ENSAMEG00000016838/ENSAMEG00000013510/ENSAMEG00000002148 | 12 |
| GO:0007052 | mitotic spindle organization | 2.98E-08 | 3.53E-06 | 3.19E-06 | ENSAMEG00000016924/ENSAMEG00000010069/ENSAMEG00000007168/ENSAMEG00000000013/ENSAMEG00000000953/ENSAMEG00000010875/ENSAMEG00000010298/ENSAMEG00000001250/ENSAMEG00000015472/ENSAMEG00000005084/ENSAMEG00000009136/ENSAMEG00000011337 | 12 |
| GO:0003678 | DNA helicase activity | 4.65E-08 | 5.16E-06 | 4.67E-06 | ENSAMEG00000014998/ENSAMEG00000001634/ENSAMEG00000005841/ENSAMEG00000018028/ENSAMEG00000008540/ENSAMEG00000004713/ENSAMEG00000008785/ENSAMEG00000011150/ENSAMEG00000013843/ENSAMEG00000001421/ENSAMEG00000009566/ENSAMEG00000000109/ENSAMEG00000006307/ENSAMEG00000016233 | 14 |
| GO:0000790 | nuclear chromatin | 5.61E-08 | 5.86E-06 | 5.30E-06 | ENSAMEG00000012407/ENSAMEG00000003466/ENSAMEG00000013206/ENSAMEG00000000014/ENSAMEG00000011867/ENSAMEG00000002791/ENSAMEG00000008991/ENSAMEG00000004713/ENSAMEG00000015678/ENSAMEG00000003946/ENSAMEG00000010750/ENSAMEG00000000622/ENSAMEG00000017674/ENSAMEG00000013676/ENSAMEG00000010700/ENSAMEG00000017275/ENSAMEG00000018491/ENSAMEG00000015417/ENSAMEG00000008529/ENSAMEG00000010868/ENSAMEG00000005560/ENSAMEG00000014262/ENSAMEG00000011337/ENSAMEG00000016271/ENSAMEG00000010799/ENSAMEG00000003503/ENSAMEG00000018402 | 27 |
| GO:0006974 | cellular response to DNA damage stimulus | 6.68E-08 | 6.59E-06 | 5.96E-06 | ENSAMEG00000007989/ENSAMEG00000001259/ENSAMEG00000002586/ENSAMEG00000005534/ENSAMEG00000000171/ENSAMEG00000009390/ENSAMEG00000014870/ENSAMEG00000013902/ENSAMEG00000013505/ENSAMEG00000016965/ENSAMEG00000011892/ENSAMEG00000015091/ENSAMEG00000004713/ENSAMEG00000003946/ENSAMEG00000003608/ENSAMEG00000016051/ENSAMEG00000001064/ENSAMEG00000010605/ENSAMEG00000011150/ENSAMEG00000013843/ENSAMEG00000002560/ENSAMEG00000000410/ENSAMEG00000015491/ENSAMEG00000012595/ENSAMEG00000005584/ENSAMEG00000009321/ENSAMEG00000001323/ENSAMEG00000008484/ENSAMEG00000006940/ENSAMEG00000014635/ENSAMEG00000000109/ENSAMEG00000012085/ENSAMEG00000013304/ENSAMEG00000003233/ENSAMEG00000016741/ENSAMEG00000001238/ENSAMEG00000004324/ENSAMEG00000009213/ENSAMEG00000015061 | 39 |
| GO:0030496 | midbody | 1.06E-07 | 9.88E-06 | 8.93E-06 | ENSAMEG00000015869/ENSAMEG00000013667/ENSAMEG00000017540/ENSAMEG00000009205/ENSAMEG00000012611/ENSAMEG00000001093/ENSAMEG00000016438/ENSAMEG00000003520/ENSAMEG00000002580/ENSAMEG00000014201/ENSAMEG00000001082/ENSAMEG00000001187/ENSAMEG00000001035/ENSAMEG00000013840/ENSAMEG00000014232/ENSAMEG00000004686/ENSAMEG00000001746/ENSAMEG00000004713/ENSAMEG00000016838/ENSAMEG00000016390/ENSAMEG00000014294/ENSAMEG00000002139/ENSAMEG00000008124/ENSAMEG00000016790/ENSAMEG00000002058 | 25 |
| GO:0018024 | histone-lysine N-methyltransferase activity | 1.56E-07 | 1.38E-05 | 1.25E-05 | ENSAMEG00000002791/ENSAMEG00000014221/ENSAMEG00000014455/ENSAMEG00000006918/ENSAMEG00000013308/ENSAMEG00000005436/ENSAMEG00000012595/ENSAMEG00000007540/ENSAMEG00000001160/ENSAMEG00000011260/ENSAMEG00000000319/ENSAMEG00000013007 | 12 |
| GO:0005814 | centriole | 1.76E-07 | 1.49E-05 | 1.35E-05 | ENSAMEG00000017945/ENSAMEG00000007989/ENSAMEG00000000013/ENSAMEG00000002675/ENSAMEG00000014232/ENSAMEG00000008931/ENSAMEG00000001549/ENSAMEG00000008648/ENSAMEG00000001250/ENSAMEG00000000293/ENSAMEG00000017728/ENSAMEG00000015698/ENSAMEG00000009136/ENSAMEG00000010387/ENSAMEG00000017365/ENSAMEG00000012848/ENSAMEG00000001860/ENSAMEG00000009815/ENSAMEG00000003208/ENSAMEG00000003927/ENSAMEG00000017216 | 21 |
| GO:0000070 | mitotic sister chromatid segregation | 3.31E-07 | 2.68E-05 | 2.42E-05 | ENSAMEG00000005342/ENSAMEG00000001187/ENSAMEG00000017084/ENSAMEG00000014232/ENSAMEG00000016838/ENSAMEG00000012346/ENSAMEG00000016796/ENSAMEG00000001352 | 8 |
| GO:0003774 | motor activity | 4.45E-07 | 3.44E-05 | 3.11E-05 | ENSAMEG00000014095/ENSAMEG00000012038/ENSAMEG00000001458/ENSAMEG00000014099/ENSAMEG00000009205/ENSAMEG00000012611/ENSAMEG00000016438/ENSAMEG00000018397/ENSAMEG00000014201/ENSAMEG00000014825/ENSAMEG00000004303/ENSAMEG00000004598/ENSAMEG00000014549/ENSAMEG00000000293/ENSAMEG00000017728/ENSAMEG00000005023/ENSAMEG00000017987/ENSAMEG00000016558/ENSAMEG00000002139/ENSAMEG00000016790/ENSAMEG00000002951/ENSAMEG00000004928 | 22 |
| GO:0007018 | microtubule-based movement | 4.73E-07 | 3.50E-05 | 3.17E-05 | ENSAMEG00000014095/ENSAMEG00000012038/ENSAMEG00000001458/ENSAMEG00000014099/ENSAMEG00000009205/ENSAMEG00000012611/ENSAMEG00000016438/ENSAMEG00000018397/ENSAMEG00000014201/ENSAMEG00000014825/ENSAMEG00000004598/ENSAMEG00000014549/ENSAMEG00000015805/ENSAMEG00000000293/ENSAMEG00000017728/ENSAMEG00000008937/ENSAMEG00000016790/ENSAMEG00000012392/ENSAMEG00000002951 | 19 |
| GO:0003777 | microtubule motor activity | 6.02E-07 | 4.28E-05 | 3.86E-05 | ENSAMEG00000014095/ENSAMEG00000012038/ENSAMEG00000001458/ENSAMEG00000014099/ENSAMEG00000009205/ENSAMEG00000012611/ENSAMEG00000016438/ENSAMEG00000018397/ENSAMEG00000014201/ENSAMEG00000014825/ENSAMEG00000004598/ENSAMEG00000014549/ENSAMEG00000015805/ENSAMEG00000000293/ENSAMEG00000017728/ENSAMEG00000008937/ENSAMEG00000016790/ENSAMEG00000012392/ENSAMEG00000002951 | 19 |
| GO:0007094 | mitotic spindle assembly checkpoint | 8.06E-07 | 5.50E-05 | 4.97E-05 | ENSAMEG00000009532/ENSAMEG00000012771/ENSAMEG00000002580/ENSAMEG00000013777/ENSAMEG00000014232/ENSAMEG00000008267/ENSAMEG00000017203/ENSAMEG00000016741 | 8 |
| GO:0051726 | regulation of cell cycle | 1.24E-06 | 8.12E-05 | 7.34E-05 | ENSAMEG00000017945/ENSAMEG00000006109/ENSAMEG00000001259/ENSAMEG00000009662/ENSAMEG00000011622/ENSAMEG00000002586/ENSAMEG00000019297/ENSAMEG00000008991/ENSAMEG00000009668/ENSAMEG00000012415/ENSAMEG00000017392/ENSAMEG00000012466/ENSAMEG00000005436/ENSAMEG00000017610/ENSAMEG00000001855/ENSAMEG00000004645/ENSAMEG00000005590/ENSAMEG00000003640/ENSAMEG00000003944/ENSAMEG00000007321 | 20 |
| GO:0003682 | chromatin binding | 1.28E-06 | 8.12E-05 | 7.34E-05 | ENSAMEG00000012588/ENSAMEG00000007989/ENSAMEG00000013934/ENSAMEG00000000014/ENSAMEG00000003520/ENSAMEG00000009254/ENSAMEG00000007193/ENSAMEG00000014870/ENSAMEG00000013454/ENSAMEG00000002114/ENSAMEG00000002791/ENSAMEG00000011892/ENSAMEG00000004713/ENSAMEG00000013104/ENSAMEG00000014221/ENSAMEG00000011823/ENSAMEG00000018450/ENSAMEG00000003344/ENSAMEG00000013940/ENSAMEG00000000371/ENSAMEG00000015779/ENSAMEG00000005029/ENSAMEG00000005777/ENSAMEG00000011651/ENSAMEG00000005053/ENSAMEG00000006185/ENSAMEG00000017674/ENSAMEG00000015499/ENSAMEG00000015524/ENSAMEG00000017275/ENSAMEG00000001685/ENSAMEG00000001160/ENSAMEG00000008529/ENSAMEG00000006307/ENSAMEG00000005807/ENSAMEG00000011337/ENSAMEG00000013360/ENSAMEG00000003040/ENSAMEG00000010799/ENSAMEG00000008231/ENSAMEG00000002954/ENSAMEG00000012914/ENSAMEG00000013007 | 43 |
| GO:0005874 | microtubule | 1.71E-06 | 9.75E-05 | 8.82E-05 | ENSAMEG00000014095/ENSAMEG00000001458/ENSAMEG00000016762/ENSAMEG00000014099/ENSAMEG00000017540/ENSAMEG00000013678/ENSAMEG00000012611/ENSAMEG00000016438/ENSAMEG00000018397/ENSAMEG00000014201/ENSAMEG00000014825/ENSAMEG00000018442/ENSAMEG00000012145/ENSAMEG00000012472/ENSAMEG00000004598/ENSAMEG00000014549/ENSAMEG00000013184/ENSAMEG00000017728/ENSAMEG00000016734/ENSAMEG00000001233/ENSAMEG00000001565/ENSAMEG00000007112/ENSAMEG00000006872/ENSAMEG00000003475/ENSAMEG00000012571/ENSAMEG00000016380/ENSAMEG00000016735 | 27 |
| GO:0007051 | spindle organization | 1.76E-06 | 9.75E-05 | 8.82E-05 | ENSAMEG00000014099/ENSAMEG00000017540/ENSAMEG00000007168/ENSAMEG00000002580/ENSAMEG00000017084/ENSAMEG00000010293/ENSAMEG00000006979/ENSAMEG00000008333 | 8 |
| GO:0007099 | centriole replication | 1.76E-06 | 9.75E-05 | 8.82E-05 | ENSAMEG00000002675/ENSAMEG00000000953/ENSAMEG00000008931/ENSAMEG00000001549/ENSAMEG00000001233/ENSAMEG00000017365/ENSAMEG00000007026/ENSAMEG00000008333 | 8 |
| GO:0051568 | histone H3-K4 methylation | 1.76E-06 | 9.75E-05 | 8.82E-05 | ENSAMEG00000014455/ENSAMEG00000006918/ENSAMEG00000013308/ENSAMEG00000007540/ENSAMEG00000000259/ENSAMEG00000001238/ENSAMEG00000000319/ENSAMEG00000013007 | 8 |
| GO:0051301 | cell division | 1.90E-06 | 1.02E-04 | 9.26E-05 | ENSAMEG00000004841/ENSAMEG00000012611/ENSAMEG00000014358/ENSAMEG00000000346/ENSAMEG00000008950/ENSAMEG00000007193/ENSAMEG00000012524/ENSAMEG00000003344/ENSAMEG00000007868/ENSAMEG00000000983/ENSAMEG00000000371/ENSAMEG00000005084/ENSAMEG00000017365/ENSAMEG00000003640 | 14 |
| GO:0032467 | positive regulation of cytokinesis | 2.22E-06 | 1.16E-04 | 1.05E-04 | ENSAMEG00000015972/ENSAMEG00000012611/ENSAMEG00000001093/ENSAMEG00000010721/ENSAMEG00000002580/ENSAMEG00000014201/ENSAMEG00000001035/ENSAMEG00000007868/ENSAMEG00000016790/ENSAMEG00000012571 | 10 |
| GO:0007098 | centrosome cycle | 2.30E-06 | 1.17E-04 | 1.05E-04 | ENSAMEG00000007168/ENSAMEG00000000171/ENSAMEG00000014232/ENSAMEG00000000503/ENSAMEG00000006979/ENSAMEG00000001121/ENSAMEG00000001233/ENSAMEG00000019816/ENSAMEG00000013304/ENSAMEG00000006467/ENSAMEG00000007432 | 11 |
| GO:0051571 | positive regulation of histone H3-K4 methylation | 3.02E-06 | 1.49E-04 | 1.34E-04 | ENSAMEG00000000171/ENSAMEG00000009813/ENSAMEG00000013257/ENSAMEG00000011823/ENSAMEG00000005777/ENSAMEG00000001238/ENSAMEG00000013007 | 7 |
| GO:0005667 | transcription factor complex | 5.46E-06 | 2.58E-04 | 2.33E-04 | ENSAMEG00000012588/ENSAMEG00000017950/ENSAMEG00000000014/ENSAMEG00000009662/ENSAMEG00000007247/ENSAMEG00000004257/ENSAMEG00000008991/ENSAMEG00000008615/ENSAMEG00000012415/ENSAMEG00000013940/ENSAMEG00000012466/ENSAMEG00000015779/ENSAMEG00000005885/ENSAMEG00000002197/ENSAMEG00000017674/ENSAMEG00000017275/ENSAMEG00000004645/ENSAMEG00000011164/ENSAMEG00000003640/ENSAMEG00000007383/ENSAMEG00000008231/ENSAMEG00000012525/ENSAMEG00000003503/ENSAMEG00000006241 | 24 |
| GO:0072686 | mitotic spindle | 5.52E-06 | 2.58E-04 | 2.33E-04 | ENSAMEG00000016924/ENSAMEG00000014099/ENSAMEG00000013678/ENSAMEG00000001093/ENSAMEG00000010767/ENSAMEG00000003520/ENSAMEG00000007168/ENSAMEG00000014201/ENSAMEG00000014825/ENSAMEG00000001035/ENSAMEG00000017084/ENSAMEG00000000492/ENSAMEG00000000410/ENSAMEG00000019816/ENSAMEG00000010861 | 15 |
| GO:0006310 | DNA recombination | 7.37E-06 | 3.36E-04 | 3.03E-04 | ENSAMEG00000013934/ENSAMEG00000005460/ENSAMEG00000000171/ENSAMEG00000016965/ENSAMEG00000015091/ENSAMEG00000013843/ENSAMEG00000009566/ENSAMEG00000000475/ENSAMEG00000016233/ENSAMEG00000004324/ENSAMEG00000011132 | 11 |
| GO:0005876 | spindle microtubule | 8.38E-06 | 3.63E-04 | 3.28E-04 | ENSAMEG00000004841/ENSAMEG00000016762/ENSAMEG00000014099/ENSAMEG00000003520/ENSAMEG00000007168/ENSAMEG00000014232/ENSAMEG00000004686/ENSAMEG00000014641/ENSAMEG00000005084/ENSAMEG00000010861 | 10 |
| GO:0051276 | chromosome organization | 8.38E-06 | 3.63E-04 | 3.28E-04 | ENSAMEG00000017537/ENSAMEG00000009491/ENSAMEG00000005936/ENSAMEG00000001082/ENSAMEG00000009390/ENSAMEG00000006653/ENSAMEG00000013902/ENSAMEG00000002220/ENSAMEG00000002560/ENSAMEG00000005053 | 10 |
| GO:0004386 | helicase activity | 9.61E-06 | 4.07E-04 | 3.67E-04 | ENSAMEG00000014998/ENSAMEG00000001634/ENSAMEG00000005841/ENSAMEG00000005460/ENSAMEG00000008540/ENSAMEG00000004713/ENSAMEG00000008785/ENSAMEG00000011150/ENSAMEG00000013843/ENSAMEG00000007401/ENSAMEG00000001421/ENSAMEG00000009566/ENSAMEG00000000475/ENSAMEG00000015543/ENSAMEG00000006307/ENSAMEG00000016233 | 16 |
| GO:0007080 | mitotic metaphase plate congression | 1.24E-05 | 5.13E-04 | 4.64E-04 | ENSAMEG00000014095/ENSAMEG00000001458/ENSAMEG00000012611/ENSAMEG00000014825/ENSAMEG00000001082/ENSAMEG00000013777/ENSAMEG00000004686/ENSAMEG00000014549/ENSAMEG00000019816/ENSAMEG00000016483 | 10 |
| GO:0000777 | condensed chromosome kinetochore | 1.37E-05 | 5.42E-04 | 4.90E-04 | ENSAMEG00000009532/ENSAMEG00000016113/ENSAMEG00000016983/ENSAMEG00000010069/ENSAMEG00000010875/ENSAMEG00000012524/ENSAMEG00000001352 | 7 |
| GO:0042800 | histone methyltransferase activity (H3-K4 specific) | 1.37E-05 | 5.42E-04 | 4.90E-04 | ENSAMEG00000014455/ENSAMEG00000006918/ENSAMEG00000013308/ENSAMEG00000007540/ENSAMEG00000000259/ENSAMEG00000000319/ENSAMEG00000013007 | 7 |
| GO:0005200 | structural constituent of cytoskeleton | 1.45E-05 | 5.61E-04 | 5.07E-04 | ENSAMEG00000003546/ENSAMEG00000012145/ENSAMEG00000012472/ENSAMEG00000013184/ENSAMEG00000014449/ENSAMEG00000016734/ENSAMEG00000001565/ENSAMEG00000007112/ENSAMEG00000006872/ENSAMEG00000015932/ENSAMEG00000009574/ENSAMEG00000016380/ENSAMEG00000016735 | 13 |
| GO:0032991 | protein-containing complex | 1.84E-05 | 6.96E-04 | 6.30E-04 | ENSAMEG00000003546/ENSAMEG00000007989/ENSAMEG00000014099/ENSAMEG00000013206/ENSAMEG00000000014/ENSAMEG00000000171/ENSAMEG00000001082/ENSAMEG00000001187/ENSAMEG00000009390/ENSAMEG00000000646/ENSAMEG00000013902/ENSAMEG00000008991/ENSAMEG00000005007/ENSAMEG00000008899/ENSAMEG00000016838/ENSAMEG00000015678/ENSAMEG00000012314/ENSAMEG00000011446/ENSAMEG00000018450/ENSAMEG00000006979/ENSAMEG00000015314/ENSAMEG00000000727/ENSAMEG00000008979/ENSAMEG00000013940/ENSAMEG00000000293/ENSAMEG00000002582/ENSAMEG00000011419/ENSAMEG00000005436/ENSAMEG00000016649/ENSAMEG00000010759/ENSAMEG00000005584/ENSAMEG00000016129/ENSAMEG00000015822/ENSAMEG00000008051/ENSAMEG00000014726/ENSAMEG00000017674/ENSAMEG00000013676/ENSAMEG00000004214/ENSAMEG00000019971/ENSAMEG00000010726/ENSAMEG00000008529/ENSAMEG00000014506/ENSAMEG00000006307/ENSAMEG00000009815/ENSAMEG00000010861/ENSAMEG00000005560/ENSAMEG00000013062/ENSAMEG00000006467/ENSAMEG00000014262/ENSAMEG00000011337/ENSAMEG00000016271/ENSAMEG00000003208/ENSAMEG00000003364/ENSAMEG00000004324/ENSAMEG00000011015 | 55 |
| GO:0019901 | protein kinase binding | 1.93E-05 | 7.10E-04 | 6.41E-04 | ENSAMEG00000013667/ENSAMEG00000004869/ENSAMEG00000011957/ENSAMEG00000014099/ENSAMEG00000013678/ENSAMEG00000006109/ENSAMEG00000012611/ENSAMEG00000001093/ENSAMEG00000016438/ENSAMEG00000010721/ENSAMEG00000007168/ENSAMEG00000013455/ENSAMEG00000000646/ENSAMEG00000016520/ENSAMEG00000014232/ENSAMEG00000009668/ENSAMEG00000005007/ENSAMEG00000012055/ENSAMEG00000001398/ENSAMEG00000001549/ENSAMEG00000016390/ENSAMEG00000001260/ENSAMEG00000004742/ENSAMEG00000001121/ENSAMEG00000011370/ENSAMEG00000008832/ENSAMEG00000017478/ENSAMEG00000001233/ENSAMEG00000018388/ENSAMEG00000009575/ENSAMEG00000008484/ENSAMEG00000015932/ENSAMEG00000019817/ENSAMEG00000010994/ENSAMEG00000009901/ENSAMEG00000017365/ENSAMEG00000005170/ENSAMEG00000009815/ENSAMEG00000005077/ENSAMEG00000003944/ENSAMEG00000002927/ENSAMEG00000005378 | 42 |
| GO:0031297 | replication fork processing | 1.96E-05 | 7.10E-04 | 6.41E-04 | ENSAMEG00000005645/ENSAMEG00000002114/ENSAMEG00000002666/ENSAMEG00000004713/ENSAMEG00000002533/ENSAMEG00000013843/ENSAMEG00000009321/ENSAMEG00000001323 | 8 |
| GO:0071897 | DNA biosynthetic process | 2.03E-05 | 7.22E-04 | 6.52E-04 | ENSAMEG00000014870/ENSAMEG00000015091/ENSAMEG00000009547/ENSAMEG00000014758/ENSAMEG00000013111/ENSAMEG00000010292/ENSAMEG00000017155/ENSAMEG00000013839/ENSAMEG00000017565 | 9 |
| GO:0007064 | mitotic sister chromatid cohesion | 2.62E-05 | 8.78E-04 | 7.94E-04 | ENSAMEG00000006653/ENSAMEG00000001637/ENSAMEG00000009369/ENSAMEG00000000622/ENSAMEG00000005053/ENSAMEG00000002148 | 6 |
| GO:0051298 | centrosome duplication | 2.62E-05 | 8.78E-04 | 7.94E-04 | ENSAMEG00000000013/ENSAMEG00000009390/ENSAMEG00000008931/ENSAMEG00000001549/ENSAMEG00000017740/ENSAMEG00000017365 | 6 |
| GO:0051382 | kinetochore assembly | 2.62E-05 | 8.78E-04 | 7.94E-04 | ENSAMEG00000017537/ENSAMEG00000002112/ENSAMEG00000009491/ENSAMEG00000013933/ENSAMEG00000011135/ENSAMEG00000000622 | 6 |
| GO:0035097 | histone methyltransferase complex | 3.16E-05 | 1.04E-03 | 9.40E-04 | ENSAMEG00000014455/ENSAMEG00000006918/ENSAMEG00000013308/ENSAMEG00000000259/ENSAMEG00000001238/ENSAMEG00000000319/ENSAMEG00000009213/ENSAMEG00000013007 | 8 |
| GO:0000082 | G1/S transition of mitotic cell cycle | 4.92E-05 | 1.56E-03 | 1.41E-03 | ENSAMEG00000014672/ENSAMEG00000013454/ENSAMEG00000009668/ENSAMEG00000005514/ENSAMEG00000011370/ENSAMEG00000017478/ENSAMEG00000006185/ENSAMEG00000005590/ENSAMEG00000003640/ENSAMEG00000011337/ENSAMEG00000002927 | 11 |
| GO:0003887 | DNA-directed DNA polymerase activity | 4.93E-05 | 1.56E-03 | 1.41E-03 | ENSAMEG00000012174/ENSAMEG00000014870/ENSAMEG00000013454/ENSAMEG00000009547/ENSAMEG00000014758/ENSAMEG00000013111/ENSAMEG00000017155/ENSAMEG00000017565 | 8 |
| GO:0007076 | mitotic chromosome condensation | 5.40E-05 | 1.68E-03 | 1.52E-03 | ENSAMEG00000000194/ENSAMEG00000014358/ENSAMEG00000005936/ENSAMEG00000008950/ENSAMEG00000007193/ENSAMEG00000002220 | 6 |
| GO:0030218 | erythrocyte differentiation | 6.75E-05 | 2.07E-03 | 1.87E-03 | ENSAMEG00000012588/ENSAMEG00000016195/ENSAMEG00000012407/ENSAMEG00000009186/ENSAMEG00000000014/ENSAMEG00000000508/ENSAMEG00000014449/ENSAMEG00000008051/ENSAMEG00000007576/ENSAMEG00000017518 | 10 |
| GO:0008094 | DNA-dependent ATPase activity | 8.89E-05 | 2.68E-03 | 2.42E-03 | ENSAMEG00000007989/ENSAMEG00000016965/ENSAMEG00000004713/ENSAMEG00000013843/ENSAMEG00000006307/ENSAMEG00000013304/ENSAMEG00000010868/ENSAMEG00000016796/ENSAMEG00000018402 | 9 |
| GO:0007010 | cytoskeleton organization | 1.19E-04 | 3.48E-03 | 3.15E-03 | ENSAMEG00000003546/ENSAMEG00000005465/ENSAMEG00000002203/ENSAMEG00000012145/ENSAMEG00000003459/ENSAMEG00000005915/ENSAMEG00000016021/ENSAMEG00000012351/ENSAMEG00000016734/ENSAMEG00000001565/ENSAMEG00000007112/ENSAMEG00000006872/ENSAMEG00000003475/ENSAMEG00000017022/ENSAMEG00000016380/ENSAMEG00000002998/ENSAMEG00000014080/ENSAMEG00000016735 | 18 |
| GO:0036297 | interstrand cross-link repair | 1.20E-04 | 3.48E-03 | 3.15E-03 | ENSAMEG00000015678/ENSAMEG00000000684/ENSAMEG00000003270/ENSAMEG00000015491/ENSAMEG00000006335/ENSAMEG00000001323/ENSAMEG00000015524 | 7 |
| GO:0010212 | response to ionizing radiation | 1.22E-04 | 3.51E-03 | 3.17E-03 | ENSAMEG00000000171/ENSAMEG00000013902/ENSAMEG00000013104/ENSAMEG00000001323/ENSAMEG00000011164/ENSAMEG00000012085/ENSAMEG00000003233/ENSAMEG00000016741/ENSAMEG00000001238 | 9 |
| GO:0016607 | nuclear speck | 1.27E-04 | 3.58E-03 | 3.24E-03 | ENSAMEG00000007247/ENSAMEG00000014825/ENSAMEG00000016777/ENSAMEG00000016520/ENSAMEG00000012055/ENSAMEG00000002220/ENSAMEG00000015996/ENSAMEG00000006296/ENSAMEG00000008321/ENSAMEG00000003215/ENSAMEG00000017412/ENSAMEG00000003804/ENSAMEG00000014934/ENSAMEG00000015431/ENSAMEG00000001807/ENSAMEG00000018018/ENSAMEG00000009372/ENSAMEG00000016270/ENSAMEG00000005341/ENSAMEG00000006089/ENSAMEG00000013117/ENSAMEG00000007576/ENSAMEG00000001685/ENSAMEG00000003904/ENSAMEG00000000259/ENSAMEG00000008529/ENSAMEG00000015502/ENSAMEG00000017518/ENSAMEG00000011668/ENSAMEG00000006373/ENSAMEG00000009101/ENSAMEG00000003944/ENSAMEG00000018299/ENSAMEG00000003200/ENSAMEG00000003503/ENSAMEG00000012914/ENSAMEG00000001352 | 37 |
| GO:0007093 | mitotic cell cycle checkpoint | 1.57E-04 | 4.36E-03 | 3.94E-03 | ENSAMEG00000014771/ENSAMEG00000012771/ENSAMEG00000005342/ENSAMEG00000003270/ENSAMEG00000001064/ENSAMEG00000003251/ENSAMEG00000012085/ENSAMEG00000013388 | 8 |
| GO:0003684 | damaged DNA binding | 1.75E-04 | 4.78E-03 | 4.32E-03 | ENSAMEG00000003715/ENSAMEG00000000171/ENSAMEG00000012174/ENSAMEG00000016965/ENSAMEG00000010293/ENSAMEG00000013940/ENSAMEG00000010937/ENSAMEG00000014262/ENSAMEG00000011323/ENSAMEG00000011132/ENSAMEG00000010799 | 11 |
| GO:0015630 | microtubule cytoskeleton | 2.01E-04 | 5.37E-03 | 4.86E-03 | ENSAMEG00000014095/ENSAMEG00000014998/ENSAMEG00000013678/ENSAMEG00000007168/ENSAMEG00000005225/ENSAMEG00000017084/ENSAMEG00000014232/ENSAMEG00000004686/ENSAMEG00000004603/ENSAMEG00000001746/ENSAMEG00000014549/ENSAMEG00000007205/ENSAMEG00000003100/ENSAMEG00000006872/ENSAMEG00000011827/ENSAMEG00000016790/ENSAMEG00000015215/ENSAMEG00000011264/ENSAMEG00000002951 | 19 |
| GO:0007017 | microtubule-based process | 2.03E-04 | 5.37E-03 | 4.86E-03 | ENSAMEG00000012145/ENSAMEG00000012472/ENSAMEG00000013184/ENSAMEG00000016734/ENSAMEG00000001565/ENSAMEG00000007112/ENSAMEG00000006872/ENSAMEG00000002951/ENSAMEG00000016380/ENSAMEG00000016735 | 10 |
| GO:0001578 | microtubule bundle formation | 2.20E-04 | 5.67E-03 | 5.13E-03 | ENSAMEG00000013667/ENSAMEG00000016438/ENSAMEG00000014232/ENSAMEG00000004686/ENSAMEG00000005084/ENSAMEG00000017378/ENSAMEG00000001233/ENSAMEG00000010861 | 8 |
| GO:0090307 | mitotic spindle assembly | 2.20E-04 | 5.67E-03 | 5.13E-03 | ENSAMEG00000001458/ENSAMEG00000014099/ENSAMEG00000013678/ENSAMEG00000001187/ENSAMEG00000003831/ENSAMEG00000001637/ENSAMEG00000017740/ENSAMEG00000008484 | 8 |
| GO:0000079 | regulation of cyclin-dependent protein serine/threonine kinase activity | 2.72E-04 | 6.89E-03 | 6.23E-03 | ENSAMEG00000009668/ENSAMEG00000012055/ENSAMEG00000003344/ENSAMEG00000013843/ENSAMEG00000011370/ENSAMEG00000017478/ENSAMEG00000005590 | 7 |
| GO:0000723 | telomere maintenance | 3.03E-04 | 7.48E-03 | 6.76E-03 | ENSAMEG00000018028/ENSAMEG00000005460/ENSAMEG00000009668/ENSAMEG00000003270/ENSAMEG00000005590/ENSAMEG00000016741/ENSAMEG00000011323/ENSAMEG00000011132 | 8 |
| GO:0000987 | proximal promoter sequence-specific DNA binding | 3.03E-04 | 7.48E-03 | 6.76E-03 | ENSAMEG00000012407/ENSAMEG00000017950/ENSAMEG00000009662/ENSAMEG00000007247/ENSAMEG00000000747/ENSAMEG00000008991/ENSAMEG00000003640/ENSAMEG00000010799 | 8 |
| GO:0004674 | protein serine/threonine kinase activity | 3.60E-04 | 8.77E-03 | 7.92E-03 | ENSAMEG00000015972/ENSAMEG00000003520/ENSAMEG00000007168/ENSAMEG00000002580/ENSAMEG00000002675/ENSAMEG00000014232/ENSAMEG00000010058/ENSAMEG00000000135/ENSAMEG00000013744/ENSAMEG00000013726/ENSAMEG00000014205/ENSAMEG00000004552/ENSAMEG00000015683/ENSAMEG00000016638/ENSAMEG00000011049/ENSAMEG00000004345/ENSAMEG00000012351/ENSAMEG00000015698/ENSAMEG00000005584/ENSAMEG00000003841/ENSAMEG00000001855/ENSAMEG00000008945/ENSAMEG00000008484/ENSAMEG00000015426/ENSAMEG00000004288/ENSAMEG00000005348/ENSAMEG00000016102/ENSAMEG00000005170/ENSAMEG00000017518/ENSAMEG00000018110/ENSAMEG00000003233/ENSAMEG00000016741/ENSAMEG00000003944/ENSAMEG00000017889/ENSAMEG00000009623/ENSAMEG00000000501/ENSAMEG00000015061 | 37 |
| GO:0006779 | porphyrin-containing compound biosynthetic process | 4.04E-04 | 9.56E-03 | 8.64E-03 | ENSAMEG00000003988/ENSAMEG00000014813/ENSAMEG00000015511/ENSAMEG00000002427/ENSAMEG00000015509 | 5 |
| GO:0017116 | single-stranded DNA helicase activity | 4.04E-04 | 9.56E-03 | 8.64E-03 | ENSAMEG00000018028/ENSAMEG00000014870/ENSAMEG00000007469/ENSAMEG00000002148/ENSAMEG00000000109 | 5 |
| GO:0003697 | single-stranded DNA binding | 4.31E-04 | 1.01E-02 | 9.10E-03 | ENSAMEG00000003715/ENSAMEG00000009390/ENSAMEG00000013505/ENSAMEG00000016965/ENSAMEG00000004713/ENSAMEG00000015678/ENSAMEG00000011150/ENSAMEG00000013843/ENSAMEG00000002560/ENSAMEG00000014262/ENSAMEG00000004324/ENSAMEG00000011323/ENSAMEG00000011132 | 13 |
| GO:0000781 | chromosome, telomeric region | 4.88E-04 | 1.13E-02 | 1.02E-02 | ENSAMEG00000005460/ENSAMEG00000002791/ENSAMEG00000003270/ENSAMEG00000013843/ENSAMEG00000008484/ENSAMEG00000010700/ENSAMEG00000016741/ENSAMEG00000011323/ENSAMEG00000011132 | 9 |
| GO:0006325 | chromatin organization | 5.06E-04 | 1.14E-02 | 1.03E-02 | ENSAMEG00000011109/ENSAMEG00000001673/ENSAMEG00000000727/ENSAMEG00000005436/ENSAMEG00000010750/ENSAMEG00000012595/ENSAMEG00000015722/ENSAMEG00000018491/ENSAMEG00000003299/ENSAMEG00000006307/ENSAMEG00000013007 | 11 |
| GO:0005815 | microtubule organizing center | 5.14E-04 | 1.14E-02 | 1.03E-02 | ENSAMEG00000007001/ENSAMEG00000016924/ENSAMEG00000013678/ENSAMEG00000007168/ENSAMEG00000013840/ENSAMEG00000000953/ENSAMEG00000008931/ENSAMEG00000010512/ENSAMEG00000001549/ENSAMEG00000001250/ENSAMEG00000008494/ENSAMEG00000000983/ENSAMEG00000002382/ENSAMEG00000001121/ENSAMEG00000001233/ENSAMEG00000017319/ENSAMEG00000014638/ENSAMEG00000000473 | 18 |
| GO:0004518 | nuclease activity | 5.15E-04 | 1.14E-02 | 1.03E-02 | ENSAMEG00000013934/ENSAMEG00000011867/ENSAMEG00000018028/ENSAMEG00000002114/ENSAMEG00000002666/ENSAMEG00000003608/ENSAMEG00000019835/ENSAMEG00000019971/ENSAMEG00000010715/ENSAMEG00000011323 | 10 |
| GO:0097431 | mitotic spindle pole | 5.50E-04 | 1.21E-02 | 1.09E-02 | ENSAMEG00000011957/ENSAMEG00000017540/ENSAMEG00000007168/ENSAMEG00000005053/ENSAMEG00000016790/ENSAMEG00000016572/ENSAMEG00000010861 | 7 |
| GO:0030863 | cortical cytoskeleton | 6.78E-04 | 1.46E-02 | 1.32E-02 | ENSAMEG00000003988/ENSAMEG00000018346/ENSAMEG00000015596/ENSAMEG00000003459/ENSAMEG00000011446/ENSAMEG00000004831 | 6 |
| GO:0003779 | actin binding | 6.81E-04 | 1.46E-02 | 1.32E-02 | ENSAMEG00000003546/ENSAMEG00000005465/ENSAMEG00000018442/ENSAMEG00000003459/ENSAMEG00000004303/ENSAMEG00000014549/ENSAMEG00000011446/ENSAMEG00000016390/ENSAMEG00000018357/ENSAMEG00000015314/ENSAMEG00000005915/ENSAMEG00000000086/ENSAMEG00000004831/ENSAMEG00000005023/ENSAMEG00000016649/ENSAMEG00000009181/ENSAMEG00000004952/ENSAMEG00000017987/ENSAMEG00000015932/ENSAMEG00000000895/ENSAMEG00000019817/ENSAMEG00000016558/ENSAMEG00000002139/ENSAMEG00000014877/ENSAMEG00000009574/ENSAMEG00000010357/ENSAMEG00000017022/ENSAMEG00000015215/ENSAMEG00000003927/ENSAMEG00000016450/ENSAMEG00000002998/ENSAMEG00000004928 | 32 |
| GO:0018105 | peptidyl-serine phosphorylation | 8.99E-04 | 1.90E-02 | 1.72E-02 | ENSAMEG00000000679/ENSAMEG00000003520/ENSAMEG00000012771/ENSAMEG00000014232/ENSAMEG00000013726/ENSAMEG00000011049/ENSAMEG00000004345/ENSAMEG00000015698/ENSAMEG00000008945/ENSAMEG00000019817/ENSAMEG00000005170/ENSAMEG00000016741/ENSAMEG00000017889/ENSAMEG00000009623/ENSAMEG00000017930 | 15 |
| GO:0001102 | RNA polymerase II activating transcription factor binding | 9.22E-04 | 1.93E-02 | 1.74E-02 | ENSAMEG00000013940/ENSAMEG00000017674/ENSAMEG00000003640/ENSAMEG00000007383/ENSAMEG00000009592/ENSAMEG00000008481/ENSAMEG00000017889/ENSAMEG00000008231 | 8 |
| GO:0090305 | nucleic acid phosphodiester bond hydrolysis | 9.51E-04 | 1.96E-02 | 1.77E-02 | ENSAMEG00000013934/ENSAMEG00000011867/ENSAMEG00000010455/ENSAMEG00000005534/ENSAMEG00000018028/ENSAMEG00000002114/ENSAMEG00000002666/ENSAMEG00000003608/ENSAMEG00000003270/ENSAMEG00000014205/ENSAMEG00000013111/ENSAMEG00000019835/ENSAMEG00000019971 | 13 |
| GO:0000226 | microtubule cytoskeleton organization | 1.10E-03 | 2.18E-02 | 1.97E-02 | ENSAMEG00000013667/ENSAMEG00000016762/ENSAMEG00000007168/ENSAMEG00000013864/ENSAMEG00000001250/ENSAMEG00000002382/ENSAMEG00000005084/ENSAMEG00000015698/ENSAMEG00000003841/ENSAMEG00000001233/ENSAMEG00000003475/ENSAMEG00000015215 | 12 |
| GO:0000228 | nuclear chromosome | 1.12E-03 | 2.18E-02 | 1.97E-02 | ENSAMEG00000007989/ENSAMEG00000014358/ENSAMEG00000005936/ENSAMEG00000019297/ENSAMEG00000013843 | 5 |
| GO:0019825 | oxygen binding | 1.12E-03 | 2.18E-02 | 1.97E-02 | ENSAMEG00000006868/ENSAMEG00000013439/ENSAMEG00000006871/ENSAMEG00000013463/ENSAMEG00000006866 | 5 |
| GO:0048188 | Set1C/COMPASS complex | 1.12E-03 | 2.18E-02 | 1.97E-02 | ENSAMEG00000014455/ENSAMEG00000018450/ENSAMEG00000013056/ENSAMEG00000007540/ENSAMEG00000000259 | 5 |
| GO:0060216 | definitive hemopoiesis | 1.12E-03 | 2.18E-02 | 1.97E-02 | ENSAMEG00000000014/ENSAMEG00000012594/ENSAMEG00000015779/ENSAMEG00000005885/ENSAMEG00000013007 | 5 |
| GO:0006302 | double-strand break repair | 1.15E-03 | 2.21E-02 | 2.00E-02 | ENSAMEG00000000171/ENSAMEG00000009390/ENSAMEG00000014870/ENSAMEG00000013902/ENSAMEG00000008267/ENSAMEG00000000137/ENSAMEG00000009586/ENSAMEG00000008484/ENSAMEG00000011164/ENSAMEG00000016796 | 10 |
| GO:0004842 | ubiquitin-protein transferase activity | 1.16E-03 | 2.21E-02 | 2.00E-02 | ENSAMEG00000012547/ENSAMEG00000001259/ENSAMEG00000012159/ENSAMEG00000000171/ENSAMEG00000010307/ENSAMEG00000011892/ENSAMEG00000008442/ENSAMEG00000008730/ENSAMEG00000003804/ENSAMEG00000002389/ENSAMEG00000001782/ENSAMEG00000017610/ENSAMEG00000011651/ENSAMEG00000001323/ENSAMEG00000017319/ENSAMEG00000007673/ENSAMEG00000004224/ENSAMEG00000004896/ENSAMEG00000013426/ENSAMEG00000003779/ENSAMEG00000004969/ENSAMEG00000014312/ENSAMEG00000000938/ENSAMEG00000015516/ENSAMEG00000013388/ENSAMEG00000013719 | 26 |
| GO:0032956 | regulation of actin cytoskeleton organization | 1.20E-03 | 2.28E-02 | 2.06E-02 | ENSAMEG00000003459/ENSAMEG00000015314/ENSAMEG00000019817/ENSAMEG00000014877/ENSAMEG00000009101/ENSAMEG00000007172/ENSAMEG00000003927/ENSAMEG00000009623/ENSAMEG00000015061 | 9 |
| GO:0000086 | G2/M transition of mitotic cell cycle | 1.34E-03 | 2.48E-02 | 2.24E-02 | ENSAMEG00000004869/ENSAMEG00000006109/ENSAMEG00000016520/ENSAMEG00000014232/ENSAMEG00000001064/ENSAMEG00000001855/ENSAMEG00000008484 | 7 |
| GO:0006275 | regulation of DNA replication | 1.38E-03 | 2.48E-02 | 2.24E-02 | ENSAMEG00000006109/ENSAMEG00000000137/ENSAMEG00000001260/ENSAMEG00000002148/ENSAMEG00000003927/ENSAMEG00000002927 | 6 |
| GO:0034968 | histone lysine methylation | 1.38E-03 | 2.48E-02 | 2.24E-02 | ENSAMEG00000006918/ENSAMEG00000013308/ENSAMEG00000012595/ENSAMEG00000007540/ENSAMEG00000001160/ENSAMEG00000011260 | 6 |
| GO:0051225 | spindle assembly | 1.38E-03 | 2.48E-02 | 2.24E-02 | ENSAMEG00000014099/ENSAMEG00000012472/ENSAMEG00000000503/ENSAMEG00000011337/ENSAMEG00000007432/ENSAMEG00000016796 | 6 |
| GO:0035064 | methylated histone binding | 1.38E-03 | 2.48E-02 | 2.24E-02 | ENSAMEG00000003466/ENSAMEG00000007193/ENSAMEG00000013286/ENSAMEG00000008321/ENSAMEG00000006185/ENSAMEG00000008054/ENSAMEG00000001685/ENSAMEG00000006307/ENSAMEG00000018402/ENSAMEG00000012914 | 10 |
| GO:0007596 | blood coagulation | 1.48E-03 | 2.60E-02 | 2.35E-02 | ENSAMEG00000006443/ENSAMEG00000018346/ENSAMEG00000009186/ENSAMEG00000002203/ENSAMEG00000017422/ENSAMEG00000011272/ENSAMEG00000018058/ENSAMEG00000011419/ENSAMEG00000010196 | 9 |
| GO:0042752 | regulation of circadian rhythm | 1.48E-03 | 2.60E-02 | 2.35E-02 | ENSAMEG00000007989/ENSAMEG00000003520/ENSAMEG00000002791/ENSAMEG00000003946/ENSAMEG00000008730/ENSAMEG00000017610/ENSAMEG00000000531/ENSAMEG00000010726/ENSAMEG00000009623 | 9 |
| GO:0030099 | myeloid cell differentiation | 1.71E-03 | 2.95E-02 | 2.66E-02 | ENSAMEG00000012588/ENSAMEG00000000014/ENSAMEG00000015101/ENSAMEG00000006878/ENSAMEG00000009213 | 5 |
| GO:0071539 | protein localization to centrosome | 1.71E-03 | 2.95E-02 | 2.66E-02 | ENSAMEG00000007168/ENSAMEG00000000013/ENSAMEG00000017740/ENSAMEG00000007026/ENSAMEG00000017216 | 5 |
| GO:0005680 | anaphase-promoting complex | 1.90E-03 | 3.18E-02 | 2.87E-02 | ENSAMEG00000012547/ENSAMEG00000001637/ENSAMEG00000016275/ENSAMEG00000003779/ENSAMEG00000016483/ENSAMEG00000005634 | 6 |
| GO:0045859 | regulation of protein kinase activity | 1.90E-03 | 3.18E-02 | 2.87E-02 | ENSAMEG00000001035/ENSAMEG00000009889/ENSAMEG00000004214/ENSAMEG00000010994/ENSAMEG00000005077/ENSAMEG00000009623 | 6 |
| GO:0070527 | platelet aggregation | 1.90E-03 | 3.18E-02 | 2.87E-02 | ENSAMEG00000012588/ENSAMEG00000002203/ENSAMEG00000018044/ENSAMEG00000014704/ENSAMEG00000011419/ENSAMEG00000004920 | 6 |
| GO:0031965 | nuclear membrane | 1.92E-03 | 3.19E-02 | 2.88E-02 | ENSAMEG00000015778/ENSAMEG00000013800/ENSAMEG00000012331/ENSAMEG00000010051/ENSAMEG00000008785/ENSAMEG00000015537/ENSAMEG00000007412/ENSAMEG00000007692/ENSAMEG00000004354/ENSAMEG00000006185/ENSAMEG00000005548/ENSAMEG00000019816/ENSAMEG00000012008/ENSAMEG00000013202/ENSAMEG00000000721/ENSAMEG00000005891/ENSAMEG00000011337/ENSAMEG00000002806/ENSAMEG00000013719/ENSAMEG00000014068/ENSAMEG00000005634 | 21 |
| GO:0000785 | chromatin | 2.18E-03 | 3.59E-02 | 3.24E-02 | ENSAMEG00000014998/ENSAMEG00000013206/ENSAMEG00000001061/ENSAMEG00000016777/ENSAMEG00000014232/ENSAMEG00000000137/ENSAMEG00000000792/ENSAMEG00000001064/ENSAMEG00000004742/ENSAMEG00000002148/ENSAMEG00000011337/ENSAMEG00000011132/ENSAMEG00000010691 | 13 |
| GO:0031648 | protein destabilization | 2.29E-03 | 3.73E-02 | 3.37E-02 | ENSAMEG00000014232/ENSAMEG00000013940/ENSAMEG00000017610/ENSAMEG00000015822/ENSAMEG00000009242/ENSAMEG00000000895/ENSAMEG00000013388/ENSAMEG00000010799 | 8 |
| GO:0060236 | regulation of mitotic spindle organization | 2.50E-03 | 4.03E-02 | 3.64E-02 | ENSAMEG00000016924/ENSAMEG00000013678/ENSAMEG00000004686/ENSAMEG00000019816/ENSAMEG00000010861 | 5 |
| GO:0000123 | histone acetyltransferase complex | 2.54E-03 | 4.07E-02 | 3.67E-02 | ENSAMEG00000018450/ENSAMEG00000013940/ENSAMEG00000016129/ENSAMEG00000006794/ENSAMEG00000010799/ENSAMEG00000014621 | 6 |
| GO:0016363 | nuclear matrix | 2.72E-03 | 4.32E-02 | 3.90E-02 | ENSAMEG00000015869/ENSAMEG00000003466/ENSAMEG00000004257/ENSAMEG00000001664/ENSAMEG00000010051/ENSAMEG00000013843/ENSAMEG00000007692/ENSAMEG00000005053/ENSAMEG00000015417/ENSAMEG00000010861 | 10 |
| GO:0045893 | positive regulation of transcription, DNA-templated | 2.78E-03 | 4.37E-02 | 3.95E-02 | ENSAMEG00000012588/ENSAMEG00000012407/ENSAMEG00000004869/ENSAMEG00000006109/ENSAMEG00000000014/ENSAMEG00000001061/ENSAMEG00000000171/ENSAMEG00000009390/ENSAMEG00000014957/ENSAMEG00000009813/ENSAMEG00000004686/ENSAMEG00000008991/ENSAMEG00000011109/ENSAMEG00000012594/ENSAMEG00000018450/ENSAMEG00000013940/ENSAMEG00000015198/ENSAMEG00000013843/ENSAMEG00000011903/ENSAMEG00000005514/ENSAMEG00000010750/ENSAMEG00000017610/ENSAMEG00000015822/ENSAMEG00000002197/ENSAMEG00000001233/ENSAMEG00000006185/ENSAMEG00000005325/ENSAMEG00000008484/ENSAMEG00000019816/ENSAMEG00000004645/ENSAMEG00000000259/ENSAMEG00000008529/ENSAMEG00000011155/ENSAMEG00000006307/ENSAMEG00000010868/ENSAMEG00000014962/ENSAMEG00000006438/ENSAMEG00000014262/ENSAMEG00000001747/ENSAMEG00000009209/ENSAMEG00000017889/ENSAMEG00000010799/ENSAMEG00000012525/ENSAMEG00000003503/ENSAMEG00000002954/ENSAMEG00000006241/ENSAMEG00000013007 | 47 |
| GO:0034644 | cellular response to UV | 2.80E-03 | 4.37E-02 | 3.95E-02 | ENSAMEG00000002580/ENSAMEG00000001398/ENSAMEG00000000135/ENSAMEG00000013940/ENSAMEG00000004884/ENSAMEG00000016796/ENSAMEG00000011323/ENSAMEG00000010799 | 8 |
| GO:0007420 | brain development | 2.95E-03 | 4.56E-02 | 4.12E-02 | ENSAMEG00000017540/ENSAMEG00000010606/ENSAMEG00000009390/ENSAMEG00000002427/ENSAMEG00000001711/ENSAMEG00000010292/ENSAMEG00000010759/ENSAMEG00000012351/ENSAMEG00000019817/ENSAMEG00000002139/ENSAMEG00000006307/ENSAMEG00000016741/ENSAMEG00000017216/ENSAMEG00000006241/ENSAMEG00000018402 | 15 |
| GO:0046777 | protein autophosphorylation | 3.09E-03 | 4.74E-02 | 4.28E-02 | ENSAMEG00000012771/ENSAMEG00000001187/ENSAMEG00000010058/ENSAMEG00000013726/ENSAMEG00000004552/ENSAMEG00000016638/ENSAMEG00000011049/ENSAMEG00000005584/ENSAMEG00000008945/ENSAMEG00000008484/ENSAMEG00000015426/ENSAMEG00000004288/ENSAMEG00000010994/ENSAMEG00000003233/ENSAMEG00000016741/ENSAMEG00000003944/ENSAMEG00000009623/ENSAMEG00000004928 | 18 |
