# Supplementary material for: Gene expression profiles during postnatal development of the liver and pancreas in giant pandas
Source: Aging (Albany NY). 2020 Aug 15;12(15):15705–29. doi: 10.18632/aging.103783 (PMC7467380; doi:10.18632/aging.103783)
Supplement: Supplementary Table 9 [file aging-12-103783-s017..docx]

**Supplementary Table 9. Significantly enriched KEGG pathways for down-regulated DEGs in liver adult group compared with liver no feeding group.**

| **ID** | **Description** | **pvalue** | **p.adjust** | **qvalue** | **geneID** | **Count** |
| --- | --- | --- | --- | --- | --- | --- |
| aml04110 | Cell cycle [PATH:aml04110] | 6.49E-23 | 1.71E-20 | 1.62E-20 | ENSAMEG00000014998/ENSAMEG00000009532/ENSAMEG00000003117/ENSAMEG00000006109/ENSAMEG00000001634/ENSAMEG00000010721/ENSAMEG00000009662/ENSAMEG00000005841/ENSAMEG00000012771/ENSAMEG00000009254/ENSAMEG00000006653/ENSAMEG00000011443/ENSAMEG00000016520/ENSAMEG00000014232/ENSAMEG00000001637/ENSAMEG00000018347/ENSAMEG00000009668/ENSAMEG00000004603/ENSAMEG00000017203/ENSAMEG00000001398/ENSAMEG00000008540/ENSAMEG00000012415/ENSAMEG00000013940/ENSAMEG00000007868/ENSAMEG00000011150/ENSAMEG00000000492/ENSAMEG00000016275/ENSAMEG00000012466/ENSAMEG00000012346/ENSAMEG00000011370/ENSAMEG00000003841/ENSAMEG00000017478/ENSAMEG00000005053/ENSAMEG00000001421/ENSAMEG00000008484/ENSAMEG00000001199/ENSAMEG00000017275/ENSAMEG00000004645/ENSAMEG00000005590/ENSAMEG00000003640/ENSAMEG00000016483/ENSAMEG00000016741/ENSAMEG00000010799/ENSAMEG00000005634 | 44 |
| aml03030 | DNA replication [PATH:aml03030] | 7.09E-11 | 9.35E-09 | 8.84E-09 | ENSAMEG00000014998/ENSAMEG00000001634/ENSAMEG00000005841/ENSAMEG00000018028/ENSAMEG00000013454/ENSAMEG00000015091/ENSAMEG00000009547/ENSAMEG00000008540/ENSAMEG00000014758/ENSAMEG00000011150/ENSAMEG00000013111/ENSAMEG00000001421/ENSAMEG00000019971/ENSAMEG00000017155/ENSAMEG00000017565/ENSAMEG00000011132 | 16 |
| aml03460 | Fanconi anemia pathway [PATH:aml03460] | 2.08E-06 | 1.83E-04 | 1.73E-04 | ENSAMEG00000011867/ENSAMEG00000009390/ENSAMEG00000012174/ENSAMEG00000002114/ENSAMEG00000011892/ENSAMEG00000016051/ENSAMEG00000008785/ENSAMEG00000015491/ENSAMEG00000012957/ENSAMEG00000015524/ENSAMEG00000010700/ENSAMEG00000011323/ENSAMEG00000011132 | 13 |
| aml04114 | Oocyte meiosis [PATH:aml04114] | 7.34E-06 | 4.84E-04 | 4.58E-04 | ENSAMEG00000009532/ENSAMEG00000003117/ENSAMEG00000007168/ENSAMEG00000000454/ENSAMEG00000000913/ENSAMEG00000006653/ENSAMEG00000016520/ENSAMEG00000014232/ENSAMEG00000001637/ENSAMEG00000009668/ENSAMEG00000004603/ENSAMEG00000007210/ENSAMEG00000001260/ENSAMEG00000016275/ENSAMEG00000012346/ENSAMEG00000017610/ENSAMEG00000005053/ENSAMEG00000011342/ENSAMEG00000005590/ENSAMEG00000008529/ENSAMEG00000016483 | 21 |
| aml03440 | Homologous recombination [PATH:aml03440] | 9.78E-06 | 5.17E-04 | 4.88E-04 | ENSAMEG00000011867/ENSAMEG00000009390/ENSAMEG00000013902/ENSAMEG00000005834/ENSAMEG00000008785/ENSAMEG00000003804/ENSAMEG00000013111/ENSAMEG00000017155/ENSAMEG00000016741/ENSAMEG00000004324/ENSAMEG00000011132 | 11 |
| aml05202 | Transcriptional misregulation in cancer [PATH:aml05202] | 1.52E-04 | 6.69E-03 | 6.32E-03 | ENSAMEG00000002934/ENSAMEG00000007625/ENSAMEG00000006109/ENSAMEG00000014221/ENSAMEG00000012594/ENSAMEG00000003337/ENSAMEG00000012773/ENSAMEG00000011764/ENSAMEG00000005436/ENSAMEG00000015779/ENSAMEG00000005885/ENSAMEG00000017478/ENSAMEG00000017674/ENSAMEG00000005341/ENSAMEG00000010127/ENSAMEG00000007748/ENSAMEG00000007383/ENSAMEG00000018575/ENSAMEG00000016741/ENSAMEG00000008231/ENSAMEG00000002954/ENSAMEG00000006241/ENSAMEG00000013007 | 23 |
| aml05166 | Human T-cell leukemia virus 1 infection [PATH:aml05166] | 2.30E-04 | 8.05E-03 | 7.61E-03 | ENSAMEG00000006109/ENSAMEG00000004472/ENSAMEG00000009662/ENSAMEG00000006653/ENSAMEG00000001637/ENSAMEG00000009668/ENSAMEG00000004603/ENSAMEG00000017203/ENSAMEG00000007210/ENSAMEG00000013940/ENSAMEG00000016275/ENSAMEG00000012346/ENSAMEG00000000410/ENSAMEG00000017478/ENSAMEG00000008484/ENSAMEG00000006794/ENSAMEG00000005590/ENSAMEG00000009574/ENSAMEG00000003640/ENSAMEG00000005170/ENSAMEG00000016483/ENSAMEG00000016741/ENSAMEG00000013883/ENSAMEG00000010799/ENSAMEG00000010798/ENSAMEG00000006086/ENSAMEG00000002058 | 27 |
| aml04914 | Progesterone-mediated oocyte maturation [PATH:aml04914] | 2.44E-04 | 8.05E-03 | 7.61E-03 | ENSAMEG00000009532/ENSAMEG00000003117/ENSAMEG00000006109/ENSAMEG00000010721/ENSAMEG00000007168/ENSAMEG00000014825/ENSAMEG00000016520/ENSAMEG00000014232/ENSAMEG00000004603/ENSAMEG00000001398/ENSAMEG00000007210/ENSAMEG00000016275/ENSAMEG00000011342/ENSAMEG00000016483/ENSAMEG00000005634 | 15 |
| aml05206 | MicroRNAs in cancer [PATH:aml05206] | 5.90E-04 | 1.73E-02 | 1.64E-02 | ENSAMEG00000010721/ENSAMEG00000009662/ENSAMEG00000014201/ENSAMEG00000016520/ENSAMEG00000002791/ENSAMEG00000009668/ENSAMEG00000008899/ENSAMEG00000001398/ENSAMEG00000013257/ENSAMEG00000011823/ENSAMEG00000010298/ENSAMEG00000013940/ENSAMEG00000012773/ENSAMEG00000014704/ENSAMEG00000009575/ENSAMEG00000005590/ENSAMEG00000005170/ENSAMEG00000016741/ENSAMEG00000009623/ENSAMEG00000010799 | 20 |
| aml03410 | Base excision repair [PATH:aml03410] | 1.14E-03 | 3.01E-02 | 2.85E-02 | ENSAMEG00000003715/ENSAMEG00000013454/ENSAMEG00000015091/ENSAMEG00000009547/ENSAMEG00000003608/ENSAMEG00000013111/ENSAMEG00000019971/ENSAMEG00000017155 | 8 |
| aml04611 | Platelet activation [PATH:aml04611] | 1.54E-03 | 3.71E-02 | 3.50E-02 | ENSAMEG00000009399/ENSAMEG00000019810/ENSAMEG00000003297/ENSAMEG00000018363/ENSAMEG00000009289/ENSAMEG00000018044/ENSAMEG00000007210/ENSAMEG00000011272/ENSAMEG00000011903/ENSAMEG00000014704/ENSAMEG00000013432/ENSAMEG00000017841/ENSAMEG00000009574/ENSAMEG00000010196/ENSAMEG00000016313/ENSAMEG00000008026 | 16 |
