# Supplementary material for: Gene expression profiles during postnatal development of the liver and pancreas in giant pandas
Source: Aging (Albany NY). 2020 Aug 15;12(15):15705–29. doi: 10.18632/aging.103783 (PMC7467380; doi:10.18632/aging.103783)
Supplement: Supplementary Table 10 [file aging-12-103783-s001..docx]

**Supplementary Table 10. Gene expression values of DEGs involved in metabolism-related terms in the liver.**

| **Gene category** | **Ensemble ID** | **Symbol** | **No feeding group (Mean CPM)** | **Suckling group (Mean CPM)** | **Adult group (Mean CPM)** |
| --- | --- | --- | --- | --- | --- |
| Carbohydrate metabolism and energy production related genes | ENSAMEG00000000169 | NDUFA1 | 22.66 | 48.41 | 115.09 |
|  | ENSAMEG00000000613 | NDUFS4 | 22.79 | 38.15 | 76.24 |
|  | ENSAMEG00000001684 | ENSAMEG00000001684 | 4.99 | 2.21 | 74.21 |
|  | ENSAMEG00000002182 | ENSAMEG00000002182 | 27.95 | 64.56 | 98.95 |
|  | ENSAMEG00000002294 | NDUFA12 | 14.11 | 26.64 | 52.51 |
|  | ENSAMEG00000003731 | COX6B2 | 0.05 | 0.24 | 1.49 |
|  | ENSAMEG00000003836 | NDUFB6 | 26.84 | 43.44 | 89.87 |
|  | ENSAMEG00000004512 | NDUFA5 | 10.61 | 23.13 | 31.45 |
|  | ENSAMEG00000004761 | NDUFB4 | 16.50 | 30.48 | 62.65 |
|  | ENSAMEG00000004785 | NDUFA4L2 | 0.63 | 3.47 | 18.55 |
|  | ENSAMEG00000005633 | AOX2 | 0.62 | 1.34 | 4.29 |
|  | ENSAMEG00000006027 | NDUFB3 | 21.62 | 32.80 | 82.45 |
|  | ENSAMEG00000006153 | COX7B | 74.04 | 136.79 | 270.20 |
|  | ENSAMEG00000006494 | GBA3 | 0.66 | 0.03 | 22.84 |
|  | ENSAMEG00000007142 | COX17 | 24.41 | 41.30 | 90.50 |
|  | ENSAMEG00000007287 | UQCRB | 74.66 | 140.03 | 316.96 |
|  | ENSAMEG00000007629 | SI | 59.41 | 3.69 | 672.68 |
|  | ENSAMEG00000009142 | FDX1 | 6.84 | 10.29 | 37.55 |
|  | ENSAMEG00000010324 | ENPP3 | 0.50 | 1.37 | 154.64 |
|  | ENSAMEG00000010344 | NDUFS5 | 37.23 | 54.08 | 141.43 |
|  | ENSAMEG00000010954 | NDUFA2 | 37.05 | 76.79 | 141.77 |
|  | ENSAMEG00000011040 | NDUFC1 | 13.02 | 22.37 | 44.76 |
|  | ENSAMEG00000011122 | GBE1 | 7.44 | 8.00 | 42.11 |
|  | ENSAMEG00000011283 | ATP6V0E1 | 30.15 | 42.96 | 102.60 |
|  | ENSAMEG00000013441 | COX7C | 136.09 | 264.13 | 677.36 |
|  | ENSAMEG00000014360 | ETFB | 70.31 | 140.12 | 301.10 |
|  | ENSAMEG00000014690 | COX11 | 5.47 | 7.79 | 17.49 |
|  | ENSAMEG00000014965 | MGAM | 13.95 | 5.34 | 60.72 |
|  | ENSAMEG00000015328 | NDUFB2 | 22.35 | 47.42 | 101.62 |
|  | ENSAMEG00000015878 | GLRX2 | 9.22 | 17.89 | 32.55 |
|  | ENSAMEG00000017601 | GCK | 1.41 | 1.01 | 269.40 |
|  | ENSAMEG00000017762 | UQCRQ | 104.16 | 155.40 | 490.18 |
|  | ENSAMEG00000017831 | PYGM | 3.46 | 1.70 | 7.56 |
| **Gene category** | **Ensemble ID** | **Symbol** | **No feeding group (Mean CPM)** | **Suckling group (Mean CPM)** | **Adult group (Mean CPM)** |
| Lipid metabolism related genes | ENSAMEG00000000342 | CYP2J2 | 4.08 | 7.68 | 37.32 |
|  | ENSAMEG00000003107 | ENSAMEG00000003107 | 0.51 | 2.31 | 21.11 |
|  | ENSAMEG00000003132 | CYP2E1 | 213.43 | 29.88 | 578.53 |
|  | ENSAMEG00000003219 | PLA2G2F | 0.13 | 0.11 | 1.25 |
|  | ENSAMEG00000004236 | HSD17B6 | 30.33 | 36.60 | 165.14 |
|  | ENSAMEG00000004376 | ENSAMEG00000004376 | 19.95 | 6.02 | 437.39 |
|  | ENSAMEG00000004846 | SLC27A6 | 156.38 | 96.30 | 405.22 |
|  | ENSAMEG00000004853 | HSD17B12 | 12.59 | 24.63 | 42.08 |
|  | ENSAMEG00000005398 | ENSAMEG00000005398 | 38.51 | 47.11 | 305.90 |
|  | ENSAMEG00000005596 | MSMO1 | 46.47 | 77.25 | 348.50 |
|  | ENSAMEG00000005730 | ENSAMEG00000005730 | 67.48 | 130.28 | 916.66 |
|  | ENSAMEG00000006243 | ENSAMEG00000006243 | 3.96 | 6.16 | 445.10 |
|  | ENSAMEG00000008222 | SC5D | 27.13 | 34.09 | 136.80 |
|  | ENSAMEG00000008506 | ACSL1 | 36.99 | 47.38 | 139.07 |
|  | ENSAMEG00000008842 | CYP1A2 | 3.44 | 6.33 | 718.08 |
|  | ENSAMEG00000008843 | SLC27A2 | 9.60 | 9.72 | 231.01 |
|  | ENSAMEG00000009440 | ENSAMEG00000009440 | 138.82 | 55.68 | 512.23 |
|  | ENSAMEG00000011617 | COMT | 51.75 | 45.63 | 173.83 |
|  | ENSAMEG00000011718 | ENSAMEG00000011718 | 0.51 | 3.68 | 32.54 |
|  | ENSAMEG00000011749 | ENSAMEG00000011749 | 24.63 | 40.09 | 229.05 |
|  | ENSAMEG00000011824 | CYP2B6 | 0.10 | 5.09 | 346.07 |
|  | ENSAMEG00000012056 | SLC27A3 | 12.44 | 9.27 | 33.99 |
|  | ENSAMEG00000012518 | ACSBG1 | 0.38 | 0.25 | 5.60 |
|  | ENSAMEG00000012592 | INSIG2 | 3.00 | 2.39 | 10.27 |
|  | ENSAMEG00000013340 | ENSAMEG00000013340 | 0.19 | 0.32 | 8.08 |
|  | ENSAMEG00000013578 | PTGIS | 1.27 | 0.56 | 9.45 |
|  | ENSAMEG00000013878 | ENSAMEG00000013878 | 46.69 | 12.32 | 164.71 |
|  | ENSAMEG00000013900 | AGMO | 1.99 | 8.86 | 27.12 |
|  | ENSAMEG00000015532 | PTGS2 | 0.34 | 1.32 | 11.38 |
|  | ENSAMEG00000016104 | ENSAMEG00000016104 | 16.84 | 18.07 | 528.65 |
|  | ENSAMEG00000016630 | MVK | 12.54 | 15.27 | 85.38 |
|  | ENSAMEG00000017636 | CYP1B1 | 0.08 | 0.03 | 0.55 |
|  | ENSAMEG00000017684 | ENSAMEG00000017684 | 50.51 | 4.45 | 346.98 |
|  | ENSAMEG00000018008 | AKR1D1 | 47.05 | 27.05 | 154.31 |
|  | ENSAMEG00000018150 | PLAAT3 | 14.31 | 18.25 | 58.74 |
|  | ENSAMEG00000020100 | CH25H | 0.07 | 0.02 | 1.49 |
| **Gene category** | **Ensemble ID** | **Symbol** | **No feeding group (Mean CPM)** | **Suckling group (Mean CPM)** | **Adult group (Mean CPM)** |
| Amino acid and protein metabolism related genes | ENSAMEG00000000028 | CTH | 74.74 | 22.14 | 93.47 |
|  | ENSAMEG00000000467 | ENSAMEG00000000467 | 8.52 | 6.74 | 26.23 |
|  | ENSAMEG00000001013 | HRG | 164.01 | 133.34 | 1383.11 |
|  | ENSAMEG00000001045 | C5 | 175.63 | 159.85 | 891.95 |
|  | ENSAMEG00000001264 | CTSK | 14.88 | 6.64 | 26.52 |
|  | ENSAMEG00000002184 | COL14A1 | 12.32 | 4.67 | 54.60 |
|  | ENSAMEG00000002287 | ENSAMEG00000002287 | 5564.64 | 1229.72 | 95194.15 |
|  | ENSAMEG00000002621 | SERPINE1 | 8.92 | 13.86 | 230.71 |
|  | ENSAMEG00000002945 | CFD | 139.21 | 93.42 | 728.40 |
|  | ENSAMEG00000003004 | PCSK6 | 6.99 | 1.06 | 35.85 |
|  | ENSAMEG00000003447 | PCSK5 | 0.31 | 0.45 | 4.22 |
|  | ENSAMEG00000003817 | COL4A3 | 1.24 | 0.10 | 10.80 |
|  | ENSAMEG00000004015 | ENSAMEG00000004015 | 4.98 | 2.13 | 20.33 |
|  | ENSAMEG00000004046 | ENSAMEG00000004046 | 24.47 | 43.27 | 626.87 |
|  | ENSAMEG00000004084 | COL4A4 | 1.45 | 0.38 | 2.92 |
|  | ENSAMEG00000004524 | COL9A2 | 5.48 | 0.65 | 9.53 |
|  | ENSAMEG00000004675 | SHMT1 | 153.91 | 79.84 | 389.45 |
|  | ENSAMEG00000004751 | SCRN2 | 9.60 | 8.99 | 51.31 |
|  | ENSAMEG00000005123 | CPE | 1.00 | 0.64 | 8.91 |
|  | ENSAMEG00000005307 | PLAT | 0.96 | 0.82 | 5.98 |
|  | ENSAMEG00000005445 | AOX1 | 119.45 | 85.85 | 567.17 |
|  | ENSAMEG00000005491 | GPT | 21.61 | 14.63 | 101.71 |
|  | ENSAMEG00000005545 | MMP25 | 6.20 | 1.04 | 5.51 |
|  | ENSAMEG00000005696 | BHMT | 1398.08 | 917.05 | 6455.10 |
|  | ENSAMEG00000005739 | DMGDH | 245.59 | 133.03 | 775.84 |
|  | ENSAMEG00000006033 | CFLAR | 16.52 | 14.31 | 87.84 |
|  | ENSAMEG00000006251 | CTSB | 220.33 | 191.43 | 673.39 |
|  | ENSAMEG00000006287 | ENSAMEG00000006287 | 0.12 | 0.03 | 9.82 |
|  | ENSAMEG00000006522 | CTSV | 171.89 | 92.90 | 512.69 |
|  | ENSAMEG00000007262 | LACTB | 32.98 | 25.55 | 80.27 |
|  | ENSAMEG00000007801 | PAMR1 | 2.62 | 0.57 | 6.26 |
|  | ENSAMEG00000008108 | ITIH4 | 2969.02 | 362.61 | 9324.75 |
|  | ENSAMEG00000008612 | SDSL | 2.22 | 0.55 | 9.37 |
|  | ENSAMEG00000008632 | SDS | 23.45 | 1.37 | 159.16 |
|  | ENSAMEG00000008842 | CYP1A2 | 3.44 | 6.33 | 718.08 |
|  | ENSAMEG00000008883 | HDC | 0.11 | 0.38 | 3.79 |
|  | ENSAMEG00000009070 | PSEN2 | 5.82 | 3.12 | 16.90 |
|  | ENSAMEG00000009102 | ALAS1 | 25.64 | 16.83 | 405.80 |
|  | ENSAMEG00000009461 | GZMA | 0.13 | 0.75 | 5.31 |
|  | ENSAMEG00000009730 | ALDH8A1 | 86.10 | 42.91 | 200.35 |
|  | ENSAMEG00000010076 | ENSAMEG00000010076 | 229.08 | 298.90 | 1388.63 |
|  | ENSAMEG00000010195 | GCAT | 3.38 | 1.27 | 5.17 |
|  | ENSAMEG00000010760 | ENSAMEG00000010760 | 24.34 | 19.33 | 67.84 |
|  | ENSAMEG00000010813 | HTRA1 | 4.35 | 4.94 | 49.78 |
|  | ENSAMEG00000010855 | ADAM33 | 0.54 | 0.13 | 1.38 |
|  | ENSAMEG00000011357 | ADAM15 | 9.22 | 2.84 | 11.20 |
|  | ENSAMEG00000011447 | CSTB | 44.29 | 38.68 | 153.73 |
|  | ENSAMEG00000011536 | ADAMTSL2 | 7.82 | 4.24 | 158.94 |
|  | ENSAMEG00000012033 | ENSAMEG00000012033 | 42.22 | 11.95 | 125.80 |
|  | ENSAMEG00000012158 | COL5A3 | 4.23 | 0.93 | 25.47 |
|  | ENSAMEG00000012647 | GATM | 5.77 | 36.69 | 411.91 |
|  | ENSAMEG00000012753 | GNMT | 587.50 | 304.90 | 3563.02 |
|  | ENSAMEG00000012868 | FGL2 | 9.15 | 4.77 | 32.72 |
|  | ENSAMEG00000013144 | CPB2 | 216.60 | 174.37 | 991.99 |
|  | ENSAMEG00000013559 | C1RL | 8.00 | 3.62 | 20.49 |
|  | ENSAMEG00000013566 | C1R | 474.77 | 342.89 | 2416.12 |
|  | ENSAMEG00000013869 | A2M | 1435.33 | 507.57 | 3752.85 |
|  | ENSAMEG00000013878 | ENSAMEG00000013878 | 46.69 | 12.32 | 164.71 |
|  | ENSAMEG00000014033 | AOC1 | 0.05 | 0.05 | 19.33 |
|  | ENSAMEG00000014114 | TIMP2 | 5.44 | 3.96 | 13.10 |
|  | ENSAMEG00000014526 | SERPING1 | 655.24 | 313.24 | 2927.60 |
|  | ENSAMEG00000014850 | ENPEP | 3.75 | 2.07 | 30.77 |
|  | ENSAMEG00000015089 | XPNPEP2 | 0.67 | 0.33 | 2.98 |
|  | ENSAMEG00000015468 | TDO2 | 111.09 | 4.93 | 707.85 |
|  | ENSAMEG00000015492 | ADAMTS4 | 0.78 | 1.41 | 13.47 |
|  | ENSAMEG00000015783 | SLC16A10 | 6.63 | 3.44 | 29.49 |
|  | ENSAMEG00000015921 | ACE2 | 0.81 | 0.45 | 56.98 |
|  | ENSAMEG00000016040 | SPOCK1 | 1.82 | 0.36 | 4.68 |
|  | ENSAMEG00000016329 | ACMSD | 14.30 | 11.61 | 250.23 |
|  | ENSAMEG00000016366 | COL28A1 | 0.14 | 0.03 | 3.72 |
|  | ENSAMEG00000016398 | PHEX | 0.34 | 0.03 | 0.90 |
|  | ENSAMEG00000016417 | MMP2 | 13.17 | 3.52 | 16.85 |
|  | ENSAMEG00000016581 | HGF | 10.17 | 2.10 | 20.41 |
|  | ENSAMEG00000016700 | AGXT | 735.03 | 324.00 | 1762.62 |
|  | ENSAMEG00000016828 | ATP1B2 | 0.66 | 0.08 | 0.75 |
|  | ENSAMEG00000017272 | ADAMTS1 | 15.05 | 8.58 | 52.24 |
|  | ENSAMEG00000017421 | CRIM1 | 16.14 | 4.77 | 46.63 |
|  | ENSAMEG00000017742 | TFPI2 | 4.03 | 1.99 | 34.41 |
|  | ENSAMEG00000017754 | TINAGL1 | 23.94 | 11.73 | 102.27 |
|  | ENSAMEG00000017831 | PYGM | 3.46 | 1.70 | 7.56 |
|  | ENSAMEG00000018429 | ENSAMEG00000018429 | 0.50 | 0.22 | 8.64 |
|  | ENSAMEG00000018686 | ALDH1B1 | 0.33 | 0.08 | 6.42 |
