# Supplementary material for: Gene expression profiles during postnatal development of the liver and pancreas in giant pandas
Source: Aging (Albany NY). 2020 Aug 15;12(15):15705–29. doi: 10.18632/aging.103783 (PMC7467380; doi:10.18632/aging.103783)
Supplement: Supplementary Tables 11, 12, 13 and 14 [file aging-12-103783-s018..pdf]

Supplementary Table 11. The top 10 of the most highly expressed genes in the three postnatal developmental stages of pancreas.

Group	Ensemble ID	Symbol	NH	YR
No feeding group	ENSAMEG00000005523	None	130105.54	66686.33
	ENSAMEG00000011940	LOC100480752	36461.33	13983.46
	ENSAMEG00000005552	CPA1	25373.42	15284.42
	ENSAMEG00000005195	PLA2G1B	26001.56	4386.25
	ENSAMEG00000013972	PNLIPRP1	11517.23	9267.59
	ENSAMEG00000023436	COX1	3886.31	11561.75
	ENSAMEG00000014546	LOC100464577	7614.33	2409.72
	ENSAMEG00000023448	ND4	2707.84	7291.34
	ENSAMEG00000023443	COX3	2261.81	6825.33
	ENSAMEG00000007786	EEF1A1	3658.02	5323.67

Group	Ensemble ID	Symbol	AB	LT
Suckling group	ENSAMEG00000005523	None	165814.10	175625.06
	ENSAMEG00000011940	LOC100480752	74841.27	85696.64
	ENSAMEG00000005552	CPA1	65402.91	49497.90
	ENSAMEG00000006262	LOC100471781	61142.86	16339.12
	ENSAMEG00000013972	PNLIPRP1	27734.17	46184.75
	ENSAMEG00000005195	PLA2G1B	31370.83	35330.32
	ENSAMEG00000002015	CELA1	43621.94	14477.40
	ENSAMEG00000014546	LOC100464577	20506.53	15850.52
	ENSAMEG00000000558	LOC100481063	14162.42	19156.90
	ENSAMEG00000010850	CPB1	8341.06	11919.32

Group	Ensemble ID	Symbol	DN	HT	YS	CC
Adult group	ENSAMEG00000002015	CELA1	116146.81	342947.14	150208.33	100347.51
	ENSAMEG00000001684	LOC100465041	63840.22	274839.39	178346.96	81741.61
	ENSAMEG00000006262	LOC100471781	120808.36	236774.31	104467.29	102473.71
	ENSAMEG00000011940	LOC100480752	103607.92	116245.93	83479.85	93495.03
	ENSAMEG00000005195	PLA2G1B	47788.19	201243.52	94425.42	44284.06
	ENSAMEG00000010850	CPB1	52942.14	91296.84	51531.31	38843.35
	ENSAMEG00000005552	CPA1	73809.22	35844.80	44489.24	45070.24
	ENSAMEG00000006287	None	48021.50	67543.05	33059.30	31087.28
	ENSAMEG00000014004	PNLIPRP2	17542.38	61695.19	39899.30	19433.31
	ENSAMEG00000006334	LOC100472031	50719.93	41412.54	11794.87	30339.94

Supplementary Table 12. Significantly enriched GO categories for up-regulated DEGs in pancreas suckling group compared with pancreas no feeding group.

ID	Description	pvalue	p.adjust	qvalue	geneID	Count
GO:0008236	serine-type peptidase activity	5.01E-04	1.37E-02	1.49E-03	ENSAMEG00000006334/ENSAMEG00000002015	2
GO:0004252	serine-type endopeptidase activity	9.46E-04	1.37E-02	1.49E-03	ENSAMEG00000006334/ENSAMEG00000002015	2
GO:0008233	peptidase activity	3.85E-03	2.01E-02	2.19E-03	ENSAMEG00000006334/ENSAMEG00000002015	2
GO:0006508	proteolysis	1.12E-02	2.95E-02	3.21E-03	ENSAMEG00000006334/ENSAMEG00000002015	2

Supplementary Table 13. Significantly enriched GO categories for down-regulated DEGs in pancreas suckling group compared with pancreas no feeding group.

ID	Description	pvalue	p.adjust	qvalue	geneID	Count
GO:0010951	negative regulation of endopeptidase activity	2.10E-08	2.16E-06	8.62E-07	ENSAMEG00000007161/ENSAMEG00000008201/ENSAMEG00000015644/ENSAMEG00000000994/ENSAMEG00000001036	5
GO:0004869	cysteine-type endopeptidase inhibitor activity	1.74E-04	8.05E-03	3.21E-03	ENSAMEG00000000994/ENSAMEG00000001036	2
GO:0035987	endodermal cell differentiation	2.35E-04	8.05E-03	3.21E-03	ENSAMEG00000001303/ENSAMEG00000003517	2
GO:0060395	SMAD protein signal transduction	4.40E-04	1.13E-02	4.51E-03	ENSAMEG00000018335/ENSAMEG00000004911	2
GO:0004867	serine-type endopeptidase inhibitor activity	1.04E-03	2.13E-02	8.50E-03	ENSAMEG00000008201/ENSAMEG00000015644	2
GO:0002020	protease binding	1.76E-03	3.01E-02	1.20E-02	ENSAMEG00000003517/ENSAMEG00000015644	2
GO:0062023	collagen-containing extracellular matrix	2.25E-03	3.31E-02	1.32E-02	ENSAMEG00000003517/ENSAMEG00000007837	2

Supplementary Table 14. Significantly enriched KEGG pathways for down-regulated DEGs in pancreas suckling group compared with pancreas no feeding group.

ID	Description	pvalue	p.adjust	qvalue	geneID	Count
aml04610	Complement and coagulation cascades [PATH:aml04610]	3.64E-06	2.55E-04	2.14E-04	ENSAMEG00000007161/ENSAMEG00000015960/ENSAMEG00000015644/ENSAMEG00000000994	4
aml05142	Chagas disease (American trypanosomiasis) [PATH:aml05142]	3.58E-04	1.25E-02	1.05E-02	ENSAMEG00000007161/ENSAMEG00000018335/ENSAMEG00000000994	3
