# Supplementary material for: Gene expression profiles during postnatal development of the liver and pancreas in giant pandas
Source: Aging (Albany NY). 2020 Aug 15;12(15):15705–29. doi: 10.18632/aging.103783 (PMC7467380; doi:10.18632/aging.103783)
Supplement: Supplementary Table 15 [file aging-12-103783-s011..docx]

**Supplementary Table 15. Significantly enriched GO categories for up-regulated DEGs in pancreas adult group compared with pancreas suckling group.**

| **ID** | **Description** | **pvalue** | **p.adjust** | **qvalue** | **geneID** | **Count** |
| --- | --- | --- | --- | --- | --- | --- |
| GO:0019882 | antigen processing and presentation | 2.86E-08 | 4.29E-05 | 3.88E-05 | ENSAMEG00000002361/ENSAMEG00000002390/ENSAMEG00000002352/ENSAMEG00000002342/ENSAMEG00000004654/ENSAMEG00000002099/ENSAMEG00000001714/ENSAMEG00000002026/ENSAMEG00000001901/ENSAMEG00000012673 | 10 |
| GO:0002376 | immune system process | 8.38E-08 | 4.49E-05 | 4.06E-05 | ENSAMEG00000002361/ENSAMEG00000002390/ENSAMEG00000002352/ENSAMEG00000002342/ENSAMEG00000002099/ENSAMEG00000018483/ENSAMEG00000001714/ENSAMEG00000005086/ENSAMEG00000001961/ENSAMEG00000001901 | 10 |
| GO:0042613 | MHC class II protein complex | 8.99E-08 | 4.49E-05 | 4.06E-05 | ENSAMEG00000002361/ENSAMEG00000002390/ENSAMEG00000002352/ENSAMEG00000002342/ENSAMEG00000004654/ENSAMEG00000002099/ENSAMEG00000001901 | 7 |
| GO:0006955 | immune response | 1.26E-06 | 4.19E-04 | 3.79E-04 | ENSAMEG00000002361/ENSAMEG00000002390/ENSAMEG00000002352/ENSAMEG00000002342/ENSAMEG00000004654/ENSAMEG00000002099/ENSAMEG00000010477/ENSAMEG00000005780/ENSAMEG00000001714/ENSAMEG00000015352/ENSAMEG00000012941/ENSAMEG00000017287/ENSAMEG00000001901/ENSAMEG00000015230/ENSAMEG00000005181/ENSAMEG00000017894/ENSAMEG00000010324/ENSAMEG00000000512/ENSAMEG00000008347/ENSAMEG00000009808 | 20 |
| GO:0002504 | antigen processing and presentation of peptide or polysaccharide antigen via MHC class II | 1.40E-06 | 4.19E-04 | 3.79E-04 | ENSAMEG00000002361/ENSAMEG00000002390/ENSAMEG00000002352/ENSAMEG00000002342/ENSAMEG00000002099/ENSAMEG00000001901 | 6 |
| GO:0006954 | inflammatory response | 1.80E-06 | 4.49E-04 | 4.06E-04 | ENSAMEG00000002782/ENSAMEG00000010292/ENSAMEG00000010616/ENSAMEG00000001230/ENSAMEG00000005780/ENSAMEG00000018483/ENSAMEG00000002178/ENSAMEG00000005086/ENSAMEG00000011331/ENSAMEG00000017287/ENSAMEG00000010153/ENSAMEG00000004128/ENSAMEG00000019911/ENSAMEG00000016020/ENSAMEG00000011987/ENSAMEG00000007766 | 16 |
| GO:0009986 | cell surface | 6.24E-06 | 1.34E-03 | 1.21E-03 | ENSAMEG00000006351/ENSAMEG00000002390/ENSAMEG00000000141/ENSAMEG00000015921/ENSAMEG00000003004/ENSAMEG00000004654/ENSAMEG00000010477/ENSAMEG00000001013/ENSAMEG00000019465/ENSAMEG00000017869/ENSAMEG00000013113/ENSAMEG00000019083/ENSAMEG00000007161/ENSAMEG00000017287/ENSAMEG00000000862/ENSAMEG00000006135/ENSAMEG00000004244/ENSAMEG00000001901/ENSAMEG00000016910/ENSAMEG00000011842/ENSAMEG00000007891/ENSAMEG00000009619/ENSAMEG00000017614/ENSAMEG00000014489/ENSAMEG00000000935/ENSAMEG00000010080/ENSAMEG00000010959/ENSAMEG00000005502 | 28 |
| GO:0010596 | negative regulation of endothelial cell migration | 1.08E-05 | 2.01E-03 | 1.82E-03 | ENSAMEG00000012740/ENSAMEG00000002851/ENSAMEG00000017287/ENSAMEG00000016821/ENSAMEG00000004377/ENSAMEG00000011842 | 6 |
| GO:2000379 | positive regulation of reactive oxygen species metabolic process | 1.61E-05 | 2.68E-03 | 2.43E-03 | ENSAMEG00000000266/ENSAMEG00000006847/ENSAMEG00000017287/ENSAMEG00000004529/ENSAMEG00000004295/ENSAMEG00000000991 | 6 |
| GO:0001965 | G-protein alpha-subunit binding | 2.02E-05 | 3.03E-03 | 2.74E-03 | ENSAMEG00000010141/ENSAMEG00000001809/ENSAMEG00000015947/ENSAMEG00000014830/ENSAMEG00000018157 | 5 |
| GO:0043235 | receptor complex | 4.14E-05 | 5.64E-03 | 5.10E-03 | ENSAMEG00000004223/ENSAMEG00000018483/ENSAMEG00000017869/ENSAMEG00000008175/ENSAMEG00000001124/ENSAMEG00000009619/ENSAMEG00000017614/ENSAMEG00000002209/ENSAMEG00000000512/ENSAMEG00000015621/ENSAMEG00000004069/ENSAMEG00000004634/ENSAMEG00000012532 | 13 |
| GO:0006915 | apoptotic process | 8.61E-05 | 1.08E-02 | 9.72E-03 | ENSAMEG00000001230/ENSAMEG00000012444/ENSAMEG00000016278/ENSAMEG00000017694/ENSAMEG00000016708/ENSAMEG00000002611/ENSAMEG00000018115/ENSAMEG00000006056/ENSAMEG00000019059/ENSAMEG00000006560/ENSAMEG00000018454/ENSAMEG00000014877/ENSAMEG00000006859/ENSAMEG00000006288/ENSAMEG00000008416 | 15 |
| GO:1901215 | negative regulation of neuron death | 1.10E-04 | 1.25E-02 | 1.13E-02 | ENSAMEG00000012740/ENSAMEG00000005086/ENSAMEG00000015799/ENSAMEG00000008234/ENSAMEG00000010421/ENSAMEG00000006711 | 6 |
| GO:0042326 | negative regulation of phosphorylation | 1.17E-04 | 1.25E-02 | 1.13E-02 | ENSAMEG00000000266/ENSAMEG00000017478/ENSAMEG00000003467/ENSAMEG00000015317/ENSAMEG00000010769 | 5 |
| GO:0008277 | regulation of G protein-coupled receptor signaling pathway | 2.27E-04 | 2.27E-02 | 2.05E-02 | ENSAMEG00000017869/ENSAMEG00000010141/ENSAMEG00000015947/ENSAMEG00000016800/ENSAMEG00000018157 | 5 |
| GO:0002250 | adaptive immune response | 2.86E-04 | 2.68E-02 | 2.42E-02 | ENSAMEG00000002361/ENSAMEG00000002390/ENSAMEG00000002352/ENSAMEG00000002342/ENSAMEG00000002099/ENSAMEG00000001901 | 6 |
| GO:0010942 | positive regulation of cell death | 4.03E-04 | 3.55E-02 | 3.21E-02 | ENSAMEG00000017869/ENSAMEG00000016708/ENSAMEG00000015317/ENSAMEG00000000991/ENSAMEG00000010769 | 5 |
| GO:0035924 | cellular response to vascular endothelial growth factor stimulus | 5.22E-04 | 4.33E-02 | 3.92E-02 | ENSAMEG00000010841/ENSAMEG00000010292/ENSAMEG00000016821/ENSAMEG00000002209/ENSAMEG00000007766 | 5 |
| GO:0030246 | carbohydrate binding | 5.83E-04 | 4.33E-02 | 3.92E-02 | ENSAMEG00000007036/ENSAMEG00000015761/ENSAMEG00000001960/ENSAMEG00000015777/ENSAMEG00000009716/ENSAMEG00000015088/ENSAMEG00000003928/ENSAMEG00000005796/ENSAMEG00000016701/ENSAMEG00000004634/ENSAMEG00000008566 | 11 |
| GO:0061028 | establishment of endothelial barrier | 5.92E-04 | 4.33E-02 | 3.92E-02 | ENSAMEG00000001809/ENSAMEG00000013593/ENSAMEG00000011968/ENSAMEG00000003264 | 4 |
| GO:0010951 | negative regulation of endopeptidase activity | 7.67E-04 | 4.33E-02 | 3.92E-02 | ENSAMEG00000014526/ENSAMEG00000001013/ENSAMEG00000000994/ENSAMEG00000002178/ENSAMEG00000007161/ENSAMEG00000006411/ENSAMEG00000012943/ENSAMEG00000007766/ENSAMEG00000004316/ENSAMEG00000016366 | 10 |
| GO:0032869 | cellular response to insulin stimulus | 7.80E-04 | 4.33E-02 | 3.92E-02 | ENSAMEG00000010477/ENSAMEG00000008695/ENSAMEG00000008175/ENSAMEG00000004255/ENSAMEG00000007766/ENSAMEG00000000935/ENSAMEG00000003623 | 7 |
| GO:0090051 | negative regulation of cell migration involved in sprouting angiogenesis | 8.31E-04 | 4.33E-02 | 3.92E-02 | ENSAMEG00000017287/ENSAMEG00000016821/ENSAMEG00000009976/ENSAMEG00000018326 | 4 |
| GO:1901214 | regulation of neuron death | 8.31E-04 | 4.33E-02 | 3.92E-02 | ENSAMEG00000000141/ENSAMEG00000015252/ENSAMEG00000012483/ENSAMEG00000015799 | 4 |
| GO:0001937 | negative regulation of endothelial cell proliferation | 8.36E-04 | 4.33E-02 | 3.92E-02 | ENSAMEG00000012740/ENSAMEG00000017287/ENSAMEG00000006135/ENSAMEG00000011842/ENSAMEG00000004295 | 5 |
| GO:0010628 | positive regulation of gene expression | 8.37E-04 | 4.33E-02 | 3.92E-02 | ENSAMEG00000010373/ENSAMEG00000010570/ENSAMEG00000004654/ENSAMEG00000014957/ENSAMEG00000017869/ENSAMEG00000005086/ENSAMEG00000009592/ENSAMEG00000016821/ENSAMEG00000016708/ENSAMEG00000016544/ENSAMEG00000015680/ENSAMEG00000016910/ENSAMEG00000016800/ENSAMEG00000010535/ENSAMEG00000003467/ENSAMEG00000015317/ENSAMEG00000008556/ENSAMEG00000012673/ENSAMEG00000010421/ENSAMEG00000007766/ENSAMEG00000000991 | 21 |
| GO:0050728 | negative regulation of inflammatory response | 8.91E-04 | 4.33E-02 | 3.92E-02 | ENSAMEG00000015830/ENSAMEG00000012740/ENSAMEG00000019175/ENSAMEG00000013578/ENSAMEG00000008499/ENSAMEG00000005003/ENSAMEG00000010324/ENSAMEG00000000935 | 8 |
| GO:0001540 | amyloid-beta binding | 8.95E-04 | 4.33E-02 | 3.92E-02 | ENSAMEG00000000141/ENSAMEG00000004179/ENSAMEG00000004654/ENSAMEG00000012740/ENSAMEG00000008175/ENSAMEG00000008234 | 6 |
| GO:0043407 | negative regulation of MAP kinase activity | 8.95E-04 | 4.33E-02 | 3.92E-02 | ENSAMEG00000011224/ENSAMEG00000012740/ENSAMEG00000015317/ENSAMEG00000008234/ENSAMEG00000018157/ENSAMEG00000000935 | 6 |
| GO:0001934 | positive regulation of protein phosphorylation | 8.96E-04 | 4.33E-02 | 3.92E-02 | ENSAMEG00000004654/ENSAMEG00000008175/ENSAMEG00000009057/ENSAMEG00000007161/ENSAMEG00000016708/ENSAMEG00000011340/ENSAMEG00000015799/ENSAMEG00000015786/ENSAMEG00000009141/ENSAMEG00000015317/ENSAMEG00000007766/ENSAMEG00000000935 | 12 |
| GO:0006869 | lipid transport | 8.97E-04 | 4.33E-02 | 3.92E-02 | ENSAMEG00000009417/ENSAMEG00000012740/ENSAMEG00000007296/ENSAMEG00000010449/ENSAMEG00000007369/ENSAMEG00000006849/ENSAMEG00000006380 | 7 |
