# Supplementary material for: Gene expression profiles during postnatal development of the liver and pancreas in giant pandas
Source: Aging (Albany NY). 2020 Aug 15;12(15):15705–29. doi: 10.18632/aging.103783 (PMC7467380; doi:10.18632/aging.103783)
Supplement: Supplementary Table 16 [file aging-12-103783-s007..docx]

**Supplementary Table 16. Significantly enriched KEGG pathways for up-regulated DEGs in pancreas adult group compared with pancreas suckling group.**

| **ID** | **Description** | **pvalue** | **p.adjust** | **qvalue** | **geneID** | **Count** |
| --- | --- | --- | --- | --- | --- | --- |
| aml04610 | Complement and coagulation cascades [PATH:aml04610] | 2.10E-08 | 5.80E-06 | 4.69E-06 | ENSAMEG00000014526/ENSAMEG00000000141/ENSAMEG00000000843/ENSAMEG00000000994/ENSAMEG00000002178/ENSAMEG00000015352/ENSAMEG00000019083/ENSAMEG00000011272/ENSAMEG00000007161/ENSAMEG00000006411/ENSAMEG00000003516/ENSAMEG00000015230/ENSAMEG00000011898/ENSAMEG00000002383/ENSAMEG00000019911/ENSAMEG00000003865 | 16 |
| aml04612 | Antigen processing and presentation [PATH:aml04612] | 4.95E-07 | 6.83E-05 | 5.52E-05 | ENSAMEG00000002390/ENSAMEG00000002352/ENSAMEG00000002342/ENSAMEG00000004654/ENSAMEG00000002099/ENSAMEG00000002302/ENSAMEG00000001976/ENSAMEG00000018827/ENSAMEG00000001714/ENSAMEG00000014772/ENSAMEG00000001901/ENSAMEG00000004634 | 12 |
| aml05332 | Graft-versus-host disease [PATH:aml05332] | 2.21E-06 | 2.04E-04 | 1.65E-04 | ENSAMEG00000002390/ENSAMEG00000002352/ENSAMEG00000002342/ENSAMEG00000002099/ENSAMEG00000002302/ENSAMEG00000001714/ENSAMEG00000001901/ENSAMEG00000004634 | 8 |
| aml04658 | Th1 and Th2 cell differentiation [PATH:aml04658] | 9.28E-06 | 6.41E-04 | 5.18E-04 | ENSAMEG00000002390/ENSAMEG00000002352/ENSAMEG00000002342/ENSAMEG00000002456/ENSAMEG00000002099/ENSAMEG00000018335/ENSAMEG00000016821/ENSAMEG00000001901/ENSAMEG00000010314/ENSAMEG00000005181/ENSAMEG00000017614/ENSAMEG00000004069/ENSAMEG00000013155 | 13 |
| aml04640 | Hematopoietic cell lineage [PATH:aml04640] | 1.49E-05 | 8.21E-04 | 6.63E-04 | ENSAMEG00000002390/ENSAMEG00000002352/ENSAMEG00000002342/ENSAMEG00000002354/ENSAMEG00000002099/ENSAMEG00000005086/ENSAMEG00000013113/ENSAMEG00000001901/ENSAMEG00000007891/ENSAMEG00000001740/ENSAMEG00000009808/ENSAMEG00000004069/ENSAMEG00000013155 | 13 |
| aml04940 | Type I diabetes mellitus [PATH:aml04940] | 2.27E-05 | 1.05E-03 | 8.46E-04 | ENSAMEG00000002390/ENSAMEG00000002352/ENSAMEG00000002342/ENSAMEG00000001325/ENSAMEG00000002099/ENSAMEG00000002302/ENSAMEG00000001714/ENSAMEG00000001901 | 8 |
| aml05330 | Allograft rejection [PATH:aml05330] | 3.73E-05 | 1.47E-03 | 1.19E-03 | ENSAMEG00000002390/ENSAMEG00000002352/ENSAMEG00000002342/ENSAMEG00000002099/ENSAMEG00000002302/ENSAMEG00000001714/ENSAMEG00000001901 | 7 |
| aml05166 | Human T-cell leukemia virus 1 infection [PATH:aml05166] | 1.34E-04 | 4.62E-03 | 3.74E-03 | ENSAMEG00000002390/ENSAMEG00000002352/ENSAMEG00000002342/ENSAMEG00000002099/ENSAMEG00000018335/ENSAMEG00000000266/ENSAMEG00000002302/ENSAMEG00000001714/ENSAMEG00000013113/ENSAMEG00000017478/ENSAMEG00000006847/ENSAMEG00000001901/ENSAMEG00000016544/ENSAMEG00000008499/ENSAMEG00000010314/ENSAMEG00000010068/ENSAMEG00000012673/ENSAMEG00000012452/ENSAMEG00000007766/ENSAMEG00000013155 | 20 |
| aml05150 | Staphylococcus aureus infection [PATH:aml05150] | 1.72E-04 | 5.23E-03 | 4.23E-03 | ENSAMEG00000002390/ENSAMEG00000002352/ENSAMEG00000002342/ENSAMEG00000002099/ENSAMEG00000000843/ENSAMEG00000002178/ENSAMEG00000011331/ENSAMEG00000007161/ENSAMEG00000001901/ENSAMEG00000011898 | 10 |
| aml05320 | Autoimmune thyroid disease [PATH:aml05320] | 1.89E-04 | 5.23E-03 | 4.23E-03 | ENSAMEG00000002390/ENSAMEG00000002352/ENSAMEG00000002342/ENSAMEG00000002099/ENSAMEG00000002302/ENSAMEG00000001714/ENSAMEG00000001901 | 7 |
| aml04974 | Protein digestion and absorption [PATH:aml04974] | 2.52E-04 | 6.33E-03 | 5.12E-03 | ENSAMEG00000015921/ENSAMEG00000002069/ENSAMEG00000020287/ENSAMEG00000003271/ENSAMEG00000005071/ENSAMEG00000007631/ENSAMEG00000017576/ENSAMEG00000010850/ENSAMEG00000010873/ENSAMEG00000001740/ENSAMEG00000003817/ENSAMEG00000016366 | 12 |
| aml04659 | Th17 cell differentiation [PATH:aml04659] | 3.52E-04 | 7.55E-03 | 6.10E-03 | ENSAMEG00000002390/ENSAMEG00000002352/ENSAMEG00000002342/ENSAMEG00000002456/ENSAMEG00000002099/ENSAMEG00000018335/ENSAMEG00000013113/ENSAMEG00000001901/ENSAMEG00000010314/ENSAMEG00000005181/ENSAMEG00000004069/ENSAMEG00000013155 | 12 |
| aml05169 | Epstein-Barr virus infection [PATH:aml05169] | 3.56E-04 | 7.55E-03 | 6.10E-03 | ENSAMEG00000002390/ENSAMEG00000002352/ENSAMEG00000002342/ENSAMEG00000016296/ENSAMEG00000002456/ENSAMEG00000002099/ENSAMEG00000000266/ENSAMEG00000002302/ENSAMEG00000001976/ENSAMEG00000001714/ENSAMEG00000001901/ENSAMEG00000007891/ENSAMEG00000012673/ENSAMEG00000004529/ENSAMEG00000007766/ENSAMEG00000013155/ENSAMEG00000010769 | 17 |
| aml00230 | Purine metabolism [PATH:aml00230] | 4.76E-04 | 9.39E-03 | 7.59E-03 | ENSAMEG00000011455/ENSAMEG00000016296/ENSAMEG00000010477/ENSAMEG00000007927/ENSAMEG00000015830/ENSAMEG00000006976/ENSAMEG00000007258/ENSAMEG00000003529/ENSAMEG00000016280/ENSAMEG00000006624/ENSAMEG00000010324/ENSAMEG00000013543/ENSAMEG00000004295/ENSAMEG00000005971 | 14 |
| aml05140 | Leishmaniasis [PATH:aml05140] | 8.11E-04 | 1.49E-02 | 1.21E-02 | ENSAMEG00000002390/ENSAMEG00000002352/ENSAMEG00000002342/ENSAMEG00000002456/ENSAMEG00000002099/ENSAMEG00000018335/ENSAMEG00000007161/ENSAMEG00000001901/ENSAMEG00000001552 | 9 |
| aml05152 | Tuberculosis [PATH:aml05152] | 9.24E-04 | 1.59E-02 | 1.29E-02 | ENSAMEG00000002390/ENSAMEG00000002352/ENSAMEG00000002342/ENSAMEG00000004654/ENSAMEG00000002456/ENSAMEG00000010292/ENSAMEG00000002099/ENSAMEG00000001230/ENSAMEG00000018483/ENSAMEG00000014772/ENSAMEG00000007161/ENSAMEG00000001901/ENSAMEG00000004770/ENSAMEG00000016020/ENSAMEG00000007766 | 15 |
| aml04972 | Pancreatic secretion [PATH:aml04972] | 1.07E-03 | 1.73E-02 | 1.40E-02 | ENSAMEG00000004305/ENSAMEG00000002069/ENSAMEG00000013928/ENSAMEG00000001684/ENSAMEG00000008518/ENSAMEG00000005071/ENSAMEG00000005972/ENSAMEG00000017576/ENSAMEG00000001124/ENSAMEG00000010850/ENSAMEG00000010873 | 11 |
| aml05323 | Rheumatoid arthritis [PATH:aml05323] | 1.28E-03 | 1.96E-02 | 1.58E-02 | ENSAMEG00000002390/ENSAMEG00000002352/ENSAMEG00000002342/ENSAMEG00000002099/ENSAMEG00000018335/ENSAMEG00000001230/ENSAMEG00000005780/ENSAMEG00000005086/ENSAMEG00000001901/ENSAMEG00000002209 | 10 |
| aml04145 | Phagosome [PATH:aml04145] | 1.52E-03 | 2.20E-02 | 1.78E-02 | ENSAMEG00000002390/ENSAMEG00000002352/ENSAMEG00000002342/ENSAMEG00000002099/ENSAMEG00000001230/ENSAMEG00000002302/ENSAMEG00000001976/ENSAMEG00000018483/ENSAMEG00000001714/ENSAMEG00000007161/ENSAMEG00000017287/ENSAMEG00000001901/ENSAMEG00000004770 | 13 |
| aml00760 | Nicotinate and nicotinamide metabolism [PATH:aml00760] | 1.59E-03 | 2.20E-02 | 1.78E-02 | ENSAMEG00000010477/ENSAMEG00000015830/ENSAMEG00000006624/ENSAMEG00000010324/ENSAMEG00000013351/ENSAMEG00000013281 | 6 |
| aml05310 | Asthma [PATH:aml05310] | 1.74E-03 | 2.28E-02 | 1.85E-02 | ENSAMEG00000002390/ENSAMEG00000002352/ENSAMEG00000002342/ENSAMEG00000002099/ENSAMEG00000001901 | 5 |
| aml04380 | Osteoclast differentiation [PATH:aml04380] | 2.05E-03 | 2.57E-02 | 2.08E-02 | ENSAMEG00000002456/ENSAMEG00000018335/ENSAMEG00000015695/ENSAMEG00000005086/ENSAMEG00000013113/ENSAMEG00000019175/ENSAMEG00000002611/ENSAMEG00000010314/ENSAMEG00000012673/ENSAMEG00000007766/ENSAMEG00000004097 | 11 |
| aml00250 | Alanine, aspartate and glutamate metabolism [PATH:aml00250] | 2.26E-03 | 2.71E-02 | 2.19E-02 | ENSAMEG00000004014/ENSAMEG00000007927/ENSAMEG00000002838/ENSAMEG00000010830/ENSAMEG00000007695/ENSAMEG00000003623 | 6 |
| aml05416 | Viral myocarditis [PATH:aml05416] | 2.66E-03 | 3.05E-02 | 2.47E-02 | ENSAMEG00000002390/ENSAMEG00000002352/ENSAMEG00000002342/ENSAMEG00000002099/ENSAMEG00000002302/ENSAMEG00000001714/ENSAMEG00000001901 | 7 |
