# Supplementary material for: Gene expression profiles during postnatal development of the liver and pancreas in giant pandas
Source: Aging (Albany NY). 2020 Aug 15;12(15):15705–29. doi: 10.18632/aging.103783 (PMC7467380; doi:10.18632/aging.103783)
Supplement: Supplementary Table 17 [file aging-12-103783-s016..docx]

**Supplementary Table 17. Significantly enriched GO categories for down-regulated DEGs in pancreas adult group compared with pancreas suckling group.**

| **ID** | **Description** | **pvalue** | **p.adjust** | **qvalue** | **geneID** | **Count** |
| --- | --- | --- | --- | --- | --- | --- |
| GO:0006260 | DNA replication | 1.31E-11 | 1.95E-08 | 1.81E-08 | ENSAMEG00000005841/ENSAMEG00000013104/ENSAMEG00000001634/ENSAMEG00000014998/ENSAMEG00000000475/ENSAMEG00000013454/ENSAMEG00000013505/ENSAMEG00000018281/ENSAMEG00000018347/ENSAMEG00000015145/ENSAMEG00000008804/ENSAMEG00000017258/ENSAMEG00000009254/ENSAMEG00000015091/ENSAMEG00000005102/ENSAMEG00000014758/ENSAMEG00000018028/ENSAMEG00000005460/ENSAMEG00000009236/ENSAMEG00000009089/ENSAMEG00000002731 | 21 |
| GO:0000776 | kinetochore | 4.92E-10 | 3.65E-07 | 3.39E-07 | ENSAMEG00000009491/ENSAMEG00000001187/ENSAMEG00000014095/ENSAMEG00000010069/ENSAMEG00000012771/ENSAMEG00000017203/ENSAMEG00000000371/ENSAMEG00000006525/ENSAMEG00000017537/ENSAMEG00000017084/ENSAMEG00000005342/ENSAMEG00000017542/ENSAMEG00000016838/ENSAMEG00000002580/ENSAMEG00000014549/ENSAMEG00000014232/ENSAMEG00000012392/ENSAMEG00000002642/ENSAMEG00000014641 | 19 |
| GO:0006270 | DNA replication initiation | 8.73E-09 | 3.62E-06 | 3.37E-06 | ENSAMEG00000005841/ENSAMEG00000001634/ENSAMEG00000014998/ENSAMEG00000011443/ENSAMEG00000007868/ENSAMEG00000013505/ENSAMEG00000010605/ENSAMEG00000009668/ENSAMEG00000005590 | 9 |
| GO:0030496 | midbody | 1.17E-08 | 3.62E-06 | 3.37E-06 | ENSAMEG00000016438/ENSAMEG00000017540/ENSAMEG00000001093/ENSAMEG00000001187/ENSAMEG00000009205/ENSAMEG00000003520/ENSAMEG00000011012/ENSAMEG00000002960/ENSAMEG00000017795/ENSAMEG00000001082/ENSAMEG00000016838/ENSAMEG00000013667/ENSAMEG00000002580/ENSAMEG00000004686/ENSAMEG00000002139/ENSAMEG00000014201/ENSAMEG00000014232/ENSAMEG00000006552/ENSAMEG00000004713/ENSAMEG00000013310/ENSAMEG00000016583 | 21 |
| GO:0000281 | mitotic cytokinesis | 1.22E-08 | 3.62E-06 | 3.37E-06 | ENSAMEG00000016438/ENSAMEG00000001093/ENSAMEG00000015972/ENSAMEG00000009390/ENSAMEG00000009205/ENSAMEG00000011827/ENSAMEG00000016762/ENSAMEG00000010298/ENSAMEG00000016838/ENSAMEG00000002139/ENSAMEG00000014201/ENSAMEG00000014232/ENSAMEG00000000690 | 13 |
| GO:0007059 | chromosome segregation | 1.74E-08 | 4.28E-06 | 3.99E-06 | ENSAMEG00000007989/ENSAMEG00000009491/ENSAMEG00000001187/ENSAMEG00000016113/ENSAMEG00000010069/ENSAMEG00000000371/ENSAMEG00000017537/ENSAMEG00000017084/ENSAMEG00000000171/ENSAMEG00000012346/ENSAMEG00000016838/ENSAMEG00000013933/ENSAMEG00000000137/ENSAMEG00000014641 | 14 |
| GO:0000922 | spindle pole | 4.31E-08 | 9.12E-06 | 8.48E-06 | ENSAMEG00000013678/ENSAMEG00000014099/ENSAMEG00000017540/ENSAMEG00000001187/ENSAMEG00000014771/ENSAMEG00000013777/ENSAMEG00000007868/ENSAMEG00000000953/ENSAMEG00000001860/ENSAMEG00000004686/ENSAMEG00000014232/ENSAMEG00000009136/ENSAMEG00000010721/ENSAMEG00000004713/ENSAMEG00000012392/ENSAMEG00000015514 | 16 |
| GO:0006281 | DNA repair | 1.20E-07 | 2.09E-05 | 1.95E-05 | ENSAMEG00000013934/ENSAMEG00000009390/ENSAMEG00000011892/ENSAMEG00000014825/ENSAMEG00000016965/ENSAMEG00000012174/ENSAMEG00000001064/ENSAMEG00000000475/ENSAMEG00000013454/ENSAMEG00000000171/ENSAMEG00000003370/ENSAMEG00000017063/ENSAMEG00000016051/ENSAMEG00000002666/ENSAMEG00000015091/ENSAMEG00000015678/ENSAMEG00000011341/ENSAMEG00000015826/ENSAMEG00000017712/ENSAMEG00000006324/ENSAMEG00000012375/ENSAMEG00000006254/ENSAMEG00000019971 | 23 |
| GO:0005876 | spindle microtubule | 1.27E-07 | 2.09E-05 | 1.95E-05 | ENSAMEG00000012031/ENSAMEG00000014099/ENSAMEG00000004841/ENSAMEG00000003520/ENSAMEG00000016762/ENSAMEG00000007168/ENSAMEG00000004686/ENSAMEG00000014631/ENSAMEG00000014232/ENSAMEG00000014641 | 10 |
| GO:0030199 | collagen fibril organization | 4.22E-07 | 6.25E-05 | 5.82E-05 | ENSAMEG00000007857/ENSAMEG00000011626/ENSAMEG00000011903/ENSAMEG00000017486/ENSAMEG00000017004/ENSAMEG00000002911/ENSAMEG00000018055/ENSAMEG00000014718/ENSAMEG00000008993/ENSAMEG00000011419 | 10 |
| GO:0000775 | chromosome, centromeric region | 7.11E-07 | 8.77E-05 | 8.16E-05 | ENSAMEG00000009491/ENSAMEG00000014095/ENSAMEG00000016113/ENSAMEG00000017537/ENSAMEG00000005435/ENSAMEG00000002960/ENSAMEG00000001082/ENSAMEG00000016838/ENSAMEG00000002443 | 9 |
| GO:0090307 | mitotic spindle assembly | 7.11E-07 | 8.77E-05 | 8.16E-05 | ENSAMEG00000013678/ENSAMEG00000014099/ENSAMEG00000001187/ENSAMEG00000001637/ENSAMEG00000003831/ENSAMEG00000002862/ENSAMEG00000001458/ENSAMEG00000017740/ENSAMEG00000008484 | 9 |
| GO:0005819 | spindle | 1.16E-06 | 1.30E-04 | 1.21E-04 | ENSAMEG00000013678/ENSAMEG00000014099/ENSAMEG00000016438/ENSAMEG00000016762/ENSAMEG00000017795/ENSAMEG00000016838/ENSAMEG00000013667/ENSAMEG00000004686/ENSAMEG00000002139/ENSAMEG00000014201/ENSAMEG00000014232/ENSAMEG00000001260 | 12 |
| GO:0007051 | spindle organization | 1.23E-06 | 1.30E-04 | 1.21E-04 | ENSAMEG00000014099/ENSAMEG00000017540/ENSAMEG00000017084/ENSAMEG00000007168/ENSAMEG00000002580/ENSAMEG00000000690/ENSAMEG00000008333 | 7 |
| GO:0005813 | centrosome | 2.03E-06 | 2.01E-04 | 1.87E-04 | ENSAMEG00000001187/ENSAMEG00000010069/ENSAMEG00000009390/ENSAMEG00000001259/ENSAMEG00000017945/ENSAMEG00000002675/ENSAMEG00000003520/ENSAMEG00000011012/ENSAMEG00000011443/ENSAMEG00000004603/ENSAMEG00000011827/ENSAMEG00000005225/ENSAMEG00000017795/ENSAMEG00000007168/ENSAMEG00000000953/ENSAMEG00000012346/ENSAMEG00000004449/ENSAMEG00000002666/ENSAMEG00000014631/ENSAMEG00000014201/ENSAMEG00000014232/ENSAMEG00000017740/ENSAMEG00000008931/ENSAMEG00000008991/ENSAMEG00000018002/ENSAMEG00000009136/ENSAMEG00000008333/ENSAMEG00000010721/ENSAMEG00000013264/ENSAMEG00000012375/ENSAMEG00000004713/ENSAMEG00000009089/ENSAMEG00000017786/ENSAMEG00000012392/ENSAMEG00000015514/ENSAMEG00000013310/ENSAMEG00000009312/ENSAMEG00000002590/ENSAMEG00000008413 | 39 |
| GO:0007076 | mitotic chromosome condensation | 3.88E-06 | 3.59E-04 | 3.34E-04 | ENSAMEG00000000194/ENSAMEG00000008950/ENSAMEG00000014358/ENSAMEG00000007193/ENSAMEG00000005936/ENSAMEG00000002220 | 6 |
| GO:0000070 | mitotic sister chromatid segregation | 7.45E-06 | 6.49E-04 | 6.04E-04 | ENSAMEG00000001187/ENSAMEG00000017084/ENSAMEG00000005342/ENSAMEG00000012346/ENSAMEG00000016838/ENSAMEG00000014232 | 6 |
| GO:0062023 | collagen-containing extracellular matrix | 1.34E-05 | 1.05E-03 | 9.76E-04 | ENSAMEG00000007909/ENSAMEG00000000126/ENSAMEG00000011626/ENSAMEG00000003517/ENSAMEG00000015429/ENSAMEG00000011687/ENSAMEG00000017465/ENSAMEG00000003815/ENSAMEG00000014718/ENSAMEG00000004454/ENSAMEG00000015203/ENSAMEG00000008993/ENSAMEG00000001432 | 13 |
| GO:0008017 | microtubule binding | 1.35E-05 | 1.05E-03 | 9.76E-04 | ENSAMEG00000012031/ENSAMEG00000014099/ENSAMEG00000016438/ENSAMEG00000001093/ENSAMEG00000014095/ENSAMEG00000018397/ENSAMEG00000011957/ENSAMEG00000007235/ENSAMEG00000009205/ENSAMEG00000012038/ENSAMEG00000014825/ENSAMEG00000001458/ENSAMEG00000011462/ENSAMEG00000016762/ENSAMEG00000013667/ENSAMEG00000014549/ENSAMEG00000004686/ENSAMEG00000015215/ENSAMEG00000014201/ENSAMEG00000014232/ENSAMEG00000016992/ENSAMEG00000000285 | 22 |
| GO:0006974 | cellular response to DNA damage stimulus | 1.59E-05 | 1.18E-03 | 1.10E-03 | ENSAMEG00000007989/ENSAMEG00000000409/ENSAMEG00000002586/ENSAMEG00000009390/ENSAMEG00000001259/ENSAMEG00000011892/ENSAMEG00000016965/ENSAMEG00000001064/ENSAMEG00000000171/ENSAMEG00000013505/ENSAMEG00000017063/ENSAMEG00000016051/ENSAMEG00000000352/ENSAMEG00000003572/ENSAMEG00000012463/ENSAMEG00000010605/ENSAMEG00000008484/ENSAMEG00000015091/ENSAMEG00000013902/ENSAMEG00000015826/ENSAMEG00000006324/ENSAMEG00000012375/ENSAMEG00000004713/ENSAMEG00000006254/ENSAMEG00000009312 | 25 |
| GO:0003697 | single-stranded DNA binding | 2.12E-05 | 1.50E-03 | 1.39E-03 | ENSAMEG00000009390/ENSAMEG00000001964/ENSAMEG00000016965/ENSAMEG00000013505/ENSAMEG00000015145/ENSAMEG00000012463/ENSAMEG00000015678/ENSAMEG00000012375/ENSAMEG00000004713/ENSAMEG00000006254/ENSAMEG00000008475/ENSAMEG00000009032 | 12 |
| GO:0000724 | double-strand break repair via homologous recombination | 2.28E-05 | 1.53E-03 | 1.43E-03 | ENSAMEG00000009390/ENSAMEG00000016965/ENSAMEG00000000171/ENSAMEG00000005645/ENSAMEG00000002666/ENSAMEG00000012463/ENSAMEG00000010605/ENSAMEG00000004921/ENSAMEG00000015678/ENSAMEG00000013902/ENSAMEG00000008475 | 11 |
| GO:0032508 | DNA duplex unwinding | 2.76E-05 | 1.78E-03 | 1.66E-03 | ENSAMEG00000005841/ENSAMEG00000001634/ENSAMEG00000000475/ENSAMEG00000008785/ENSAMEG00000017063/ENSAMEG00000017957/ENSAMEG00000018028/ENSAMEG00000005460/ENSAMEG00000009236/ENSAMEG00000004713 | 10 |
| GO:0005694 | chromosome | 3.49E-05 | 2.15E-03 | 2.00E-03 | ENSAMEG00000000346/ENSAMEG00000017537/ENSAMEG00000018491/ENSAMEG00000000171/ENSAMEG00000001226/ENSAMEG00000000352/ENSAMEG00000018517/ENSAMEG00000019576/ENSAMEG00000000137/ENSAMEG00000005936/ENSAMEG00000018538/ENSAMEG00000000959/ENSAMEG00000018498/ENSAMEG00000018579/ENSAMEG00000018575/ENSAMEG00000011527/ENSAMEG00000019742/ENSAMEG00000018489/ENSAMEG00000002220 | 19 |
| GO:0000132 | establishment of mitotic spindle orientation | 8.16E-05 | 4.67E-03 | 4.35E-03 | ENSAMEG00000006908/ENSAMEG00000010069/ENSAMEG00000013777/ENSAMEG00000002862/ENSAMEG00000004449/ENSAMEG00000014232 | 6 |
| GO:0007052 | mitotic spindle organization | 8.21E-05 | 4.67E-03 | 4.35E-03 | ENSAMEG00000010069/ENSAMEG00000016924/ENSAMEG00000010298/ENSAMEG00000007168/ENSAMEG00000000953/ENSAMEG00000004449/ENSAMEG00000009136 | 7 |
| GO:0007018 | microtubule-based movement | 8.81E-05 | 4.83E-03 | 4.50E-03 | ENSAMEG00000014099/ENSAMEG00000016438/ENSAMEG00000014095/ENSAMEG00000018397/ENSAMEG00000009205/ENSAMEG00000012038/ENSAMEG00000014825/ENSAMEG00000001458/ENSAMEG00000013139/ENSAMEG00000014549/ENSAMEG00000014201/ENSAMEG00000012392 | 12 |
| GO:0015630 | microtubule cytoskeleton | 9.64E-05 | 5.10E-03 | 4.75E-03 | ENSAMEG00000013678/ENSAMEG00000014095/ENSAMEG00000014998/ENSAMEG00000017084/ENSAMEG00000004603/ENSAMEG00000011827/ENSAMEG00000005225/ENSAMEG00000001384/ENSAMEG00000007168/ENSAMEG00000014549/ENSAMEG00000004686/ENSAMEG00000015215/ENSAMEG00000014232/ENSAMEG00000000285/ENSAMEG00000009032 | 15 |
| GO:0003777 | microtubule motor activity | 1.02E-04 | 5.19E-03 | 4.83E-03 | ENSAMEG00000014099/ENSAMEG00000016438/ENSAMEG00000014095/ENSAMEG00000018397/ENSAMEG00000009205/ENSAMEG00000012038/ENSAMEG00000014825/ENSAMEG00000001458/ENSAMEG00000013139/ENSAMEG00000014549/ENSAMEG00000014201/ENSAMEG00000012392 | 12 |
| GO:0019825 | oxygen binding | 1.33E-04 | 6.49E-03 | 6.04E-03 | ENSAMEG00000006871/ENSAMEG00000013463/ENSAMEG00000001404/ENSAMEG00000013439/ENSAMEG00000006868 | 5 |
| GO:0003678 | DNA helicase activity | 1.36E-04 | 6.49E-03 | 6.04E-03 | ENSAMEG00000005841/ENSAMEG00000001634/ENSAMEG00000014998/ENSAMEG00000008785/ENSAMEG00000017063/ENSAMEG00000017957/ENSAMEG00000018028/ENSAMEG00000004713 | 8 |
| GO:0051276 | chromosome organization | 1.44E-04 | 6.65E-03 | 6.19E-03 | ENSAMEG00000009491/ENSAMEG00000009390/ENSAMEG00000017537/ENSAMEG00000001082/ENSAMEG00000005936/ENSAMEG00000013902/ENSAMEG00000002220 | 7 |
| GO:0000278 | mitotic cell cycle | 1.75E-04 | 7.76E-03 | 7.22E-03 | ENSAMEG00000009491/ENSAMEG00000000135/ENSAMEG00000000371/ENSAMEG00000017537/ENSAMEG00000014358/ENSAMEG00000007868/ENSAMEG00000007168/ENSAMEG00000012346/ENSAMEG00000014232/ENSAMEG00000014641 | 10 |
| GO:0005201 | extracellular matrix structural constituent | 1.86E-04 | 7.76E-03 | 7.22E-03 | ENSAMEG00000011626/ENSAMEG00000011903/ENSAMEG00000017486/ENSAMEG00000015429/ENSAMEG00000016642/ENSAMEG00000016892/ENSAMEG00000014718 | 7 |
| GO:0007080 | mitotic metaphase plate congression | 1.86E-04 | 7.76E-03 | 7.22E-03 | ENSAMEG00000014095/ENSAMEG00000013777/ENSAMEG00000014825/ENSAMEG00000001458/ENSAMEG00000001082/ENSAMEG00000014549/ENSAMEG00000004686 | 7 |
| GO:0005874 | microtubule | 1.89E-04 | 7.76E-03 | 7.22E-03 | ENSAMEG00000012031/ENSAMEG00000013678/ENSAMEG00000014099/ENSAMEG00000016438/ENSAMEG00000017540/ENSAMEG00000014095/ENSAMEG00000018397/ENSAMEG00000007235/ENSAMEG00000012145/ENSAMEG00000014825/ENSAMEG00000001384/ENSAMEG00000001458/ENSAMEG00000016762/ENSAMEG00000014549/ENSAMEG00000014201/ENSAMEG00000016992/ENSAMEG00000007112 | 17 |
| GO:0007094 | mitotic spindle assembly checkpoint | 2.08E-04 | 8.32E-03 | 7.74E-03 | ENSAMEG00000012771/ENSAMEG00000017203/ENSAMEG00000013777/ENSAMEG00000002580/ENSAMEG00000014232 | 5 |
| GO:0003682 | chromatin binding | 3.83E-04 | 1.49E-02 | 1.39E-02 | ENSAMEG00000007989/ENSAMEG00000006908/ENSAMEG00000013104/ENSAMEG00000013934/ENSAMEG00000003344/ENSAMEG00000000371/ENSAMEG00000003520/ENSAMEG00000011892/ENSAMEG00000013454/ENSAMEG00000007193/ENSAMEG00000011651/ENSAMEG00000002791/ENSAMEG00000015145/ENSAMEG00000002443/ENSAMEG00000012637/ENSAMEG00000009254/ENSAMEG00000003040/ENSAMEG00000014221/ENSAMEG00000002114/ENSAMEG00000014100/ENSAMEG00000006324/ENSAMEG00000004713/ENSAMEG00000006254/ENSAMEG00000009733/ENSAMEG00000009089/ENSAMEG00000008475 | 26 |
| GO:0051965 | positive regulation of synapse assembly | 4.02E-04 | 1.53E-02 | 1.42E-02 | ENSAMEG00000016009/ENSAMEG00000014794/ENSAMEG00000000530/ENSAMEG00000003994/ENSAMEG00000017786/ENSAMEG00000013498 | 6 |
| GO:0006310 | DNA recombination | 4.68E-04 | 1.73E-02 | 1.61E-02 | ENSAMEG00000013934/ENSAMEG00000016965/ENSAMEG00000000475/ENSAMEG00000000171/ENSAMEG00000017957/ENSAMEG00000015091/ENSAMEG00000005460 | 7 |
| GO:0032467 | positive regulation of cytokinesis | 5.23E-04 | 1.89E-02 | 1.76E-02 | ENSAMEG00000001093/ENSAMEG00000015972/ENSAMEG00000007868/ENSAMEG00000002580/ENSAMEG00000014201/ENSAMEG00000010721 | 6 |
| GO:0031012 | extracellular matrix | 5.73E-04 | 2.02E-02 | 1.88E-02 | ENSAMEG00000007909/ENSAMEG00000011626/ENSAMEG00000011903/ENSAMEG00000014237/ENSAMEG00000001604/ENSAMEG00000013801/ENSAMEG00000003517/ENSAMEG00000018103/ENSAMEG00000008399/ENSAMEG00000007055/ENSAMEG00000017465/ENSAMEG00000018055/ENSAMEG00000008993 | 13 |
| GO:0001578 | microtubule bundle formation | 6.70E-04 | 2.31E-02 | 2.15E-02 | ENSAMEG00000012031/ENSAMEG00000016438/ENSAMEG00000013667/ENSAMEG00000004686/ENSAMEG00000014232/ENSAMEG00000005000 | 6 |
| GO:0016446 | somatic hypermutation of immunoglobulin genes | 7.96E-04 | 2.62E-02 | 2.44E-02 | ENSAMEG00000013934/ENSAMEG00000012463/ENSAMEG00000006324/ENSAMEG00000006254 | 4 |
| GO:0048407 | platelet-derived growth factor binding | 7.96E-04 | 2.62E-02 | 2.44E-02 | ENSAMEG00000011626/ENSAMEG00000011903/ENSAMEG00000017486/ENSAMEG00000016642 | 4 |
| GO:0072686 | mitotic spindle | 8.20E-04 | 2.64E-02 | 2.46E-02 | ENSAMEG00000013678/ENSAMEG00000014099/ENSAMEG00000001093/ENSAMEG00000016924/ENSAMEG00000003520/ENSAMEG00000017084/ENSAMEG00000014825/ENSAMEG00000007168/ENSAMEG00000014201 | 9 |
| GO:0005581 | collagen trimer | 8.48E-04 | 2.67E-02 | 2.49E-02 | ENSAMEG00000011626/ENSAMEG00000011903/ENSAMEG00000017486/ENSAMEG00000009885/ENSAMEG00000016642/ENSAMEG00000016892/ENSAMEG00000018055 | 7 |
| GO:0051301 | cell division | 8.77E-04 | 2.71E-02 | 2.52E-02 | ENSAMEG00000004841/ENSAMEG00000000346/ENSAMEG00000003344/ENSAMEG00000008950/ENSAMEG00000000371/ENSAMEG00000014358/ENSAMEG00000007193/ENSAMEG00000007868 | 8 |
| GO:0001662 | behavioral fear response | 1.14E-03 | 3.31E-02 | 3.08E-02 | ENSAMEG00000006752/ENSAMEG00000016852/ENSAMEG00000006117/ENSAMEG00000013264/ENSAMEG00000017091 | 5 |
| GO:0006298 | mismatch repair | 1.14E-03 | 3.31E-02 | 3.08E-02 | ENSAMEG00000013934/ENSAMEG00000006324/ENSAMEG00000006254/ENSAMEG00000009089/ENSAMEG00000009032 | 5 |
| GO:0031297 | replication fork processing | 1.14E-03 | 3.31E-02 | 3.08E-02 | ENSAMEG00000005645/ENSAMEG00000002666/ENSAMEG00000002114/ENSAMEG00000004713/ENSAMEG00000009089 | 5 |
| GO:0035249 | synaptic transmission, glutamatergic | 1.20E-03 | 3.36E-02 | 3.13E-02 | ENSAMEG00000004677/ENSAMEG00000013264/ENSAMEG00000010685/ENSAMEG00000013498 | 4 |
| GO:0071168 | protein localization to chromatin | 1.20E-03 | 3.36E-02 | 3.13E-02 | ENSAMEG00000002791/ENSAMEG00000014232/ENSAMEG00000000137/ENSAMEG00000006254 | 4 |
| GO:0051726 | regulation of cell cycle | 1.33E-03 | 3.66E-02 | 3.40E-02 | ENSAMEG00000002586/ENSAMEG00000001259/ENSAMEG00000017945/ENSAMEG00000019297/ENSAMEG00000004645/ENSAMEG00000019799/ENSAMEG00000009662/ENSAMEG00000008991/ENSAMEG00000009668/ENSAMEG00000000690/ENSAMEG00000005590 | 11 |
| GO:0000228 | nuclear chromosome | 1.74E-03 | 4.52E-02 | 4.21E-02 | ENSAMEG00000007989/ENSAMEG00000019297/ENSAMEG00000014358/ENSAMEG00000005936 | 4 |
| GO:0031018 | endocrine pancreas development | 1.74E-03 | 4.52E-02 | 4.21E-02 | ENSAMEG00000019033/ENSAMEG00000005843/ENSAMEG00000000819/ENSAMEG00000012637 | 4 |
| GO:0046940 | nucleoside monophosphate phosphorylation | 1.74E-03 | 4.52E-02 | 4.21E-02 | ENSAMEG00000007410/ENSAMEG00000000012/ENSAMEG00000016421/ENSAMEG00000002640 | 4 |
