# Supplementary material for: Gene expression profiles during postnatal development of the liver and pancreas in giant pandas
Source: Aging (Albany NY). 2020 Aug 15;12(15):15705–29. doi: 10.18632/aging.103783 (PMC7467380; doi:10.18632/aging.103783)
Supplement: Supplementary Table 18 [file aging-12-103783-s019..pdf]

Supplementary Table 18. Significantly enriched KEGG pathways for down-regulated DEGs in pancreas adult group compared with pancreas suckling group.

ID	Description	pvalue	p.adjust	qvalue	geneID	Count
aml04110	Cell cycle [PATH:aml04110]	1.97E-10	4.98E-08	4.64E-08	ENSAMEG00000003117/ENSAMEG00000005841/ENSAMEG00000001637/ENSAMEG00000012771/ENSAMEG00000001634/ENSAMEG00000017203/ENSAMEG00000014998/ENSAMEG00000011443/ENSAMEG00000004603/ENSAMEG00000004645/ENSAMEG00000007868/ENSAMEG00000018347/ENSAMEG00000012346/ENSAMEG00000014631/ENSAMEG00000001398/ENSAMEG00000009254/ENSAMEG00000009662/ENSAMEG00000014232/ENSAMEG00000008484/ENSAMEG00000009668/ENSAMEG00000010721/ENSAMEG00000000328/ENSAMEG00000009089/ENSAMEG00000005590	24
aml03030	DNA replication [PATH:aml03030]	1.27E-07	1.60E-05	1.49E-05	ENSAMEG00000005841/ENSAMEG00000001634/ENSAMEG00000014998/ENSAMEG00000013454/ENSAMEG00000015145/ENSAMEG00000015091/ENSAMEG00000014758/ENSAMEG00000018028/ENSAMEG00000009236/ENSAMEG00000019971/ENSAMEG00000009089	11
aml04512	ECM-receptor interaction [PATH:aml04512]	4.65E-07	3.92E-05	3.65E-05	ENSAMEG00000011903/ENSAMEG00000017486/ENSAMEG00000016281/ENSAMEG00000003517/ENSAMEG00000012170/ENSAMEG00000016642/ENSAMEG00000000087/ENSAMEG00000015074/ENSAMEG00000004524/ENSAMEG00000000779/ENSAMEG00000000836/ENSAMEG00000016892/ENSAMEG00000017465/ENSAMEG00000014404/ENSAMEG00000016273/ENSAMEG00000011419	16
aml03430	Mismatch repair [PATH:aml03430]	2.87E-06	1.81E-04	1.69E-04	ENSAMEG00000013934/ENSAMEG00000015145/ENSAMEG00000015091/ENSAMEG00000009236/ENSAMEG00000006324/ENSAMEG00000006254/ENSAMEG00000009089/ENSAMEG00000009032	8
aml03460	Fanconi anemia pathway [PATH:aml03460]	8.32E-05	4.21E-03	3.93E-03	ENSAMEG00000009390/ENSAMEG00000011892/ENSAMEG00000012174/ENSAMEG00000008785/ENSAMEG00000016051/ENSAMEG00000011341/ENSAMEG00000002114/ENSAMEG00000015826/ENSAMEG00000009032	9
aml04974	Protein digestion and absorption [PATH:aml04974]	5.69E-04	2.40E-02	2.24E-02	ENSAMEG00000011626/ENSAMEG00000011903/ENSAMEG00000005540/ENSAMEG00000017486/ENSAMEG00000012170/ENSAMEG00000000748/ENSAMEG00000008339/ENSAMEG00000016642/ENSAMEG00000013732/ENSAMEG00000004524/ENSAMEG00000016892/ENSAMEG00000006555	12
aml04950	Maturity onset diabetes of the young [PATH:aml04950]	6.73E-04	2.43E-02	2.27E-02	ENSAMEG00000019033/ENSAMEG00000006908/ENSAMEG00000005843/ENSAMEG00000017601/ENSAMEG00000000055	5
