# Supplementary material for: Gene expression profiles during postnatal development of the liver and pancreas in giant pandas
Source: Aging (Albany NY). 2020 Aug 15;12(15):15705–29. doi: 10.18632/aging.103783 (PMC7467380; doi:10.18632/aging.103783)
Supplement: Supplementary Table 19 [file aging-12-103783-s012..docx]

**Supplementary Table 19. Significantly enriched GO categories for up-regulated DEGs in pancreas adult group compared with pancreas no feeding group.**

| **ID** | **Description** | **pvalue** | **p.adjust** | **qvalue** | **geneID** | **Count** |
| --- | --- | --- | --- | --- | --- | --- |
| GO:0005840 | ribosome | 1.25E-06 | 1.17E-03 | 1.13E-03 | ENSAMEG00000015647/ENSAMEG00000019284/ENSAMEG00000008470/ENSAMEG00000011876/ENSAMEG00000011918/ENSAMEG00000004583/ENSAMEG00000016537/ENSAMEG00000002153/ENSAMEG00000011350/ENSAMEG00000014954/ENSAMEG00000004181/ENSAMEG00000014027/ENSAMEG00000004644/ENSAMEG00000011356/ENSAMEG00000016931/ENSAMEG00000017399/ENSAMEG00000008591/ENSAMEG00000017676/ENSAMEG00000001047/ENSAMEG00000012349/ENSAMEG00000004730/ENSAMEG00000007270/ENSAMEG00000014020/ENSAMEG00000015966/ENSAMEG00000013932/ENSAMEG00000019379/ENSAMEG00000017519/ENSAMEG00000001703/ENSAMEG00000019255/ENSAMEG00000015424/ENSAMEG00000004917/ENSAMEG00000001463/ENSAMEG00000012068/ENSAMEG00000010878/ENSAMEG00000000605/ENSAMEG00000003764/ENSAMEG00000014251/ENSAMEG00000004630/ENSAMEG00000001269 | 39 |
| GO:0003735 | structural constituent of ribosome | 1.45E-06 | 1.17E-03 | 1.13E-03 | ENSAMEG00000015647/ENSAMEG00000019284/ENSAMEG00000008470/ENSAMEG00000011876/ENSAMEG00000011918/ENSAMEG00000004583/ENSAMEG00000016292/ENSAMEG00000008391/ENSAMEG00000016537/ENSAMEG00000002153/ENSAMEG00000011350/ENSAMEG00000014954/ENSAMEG00000004181/ENSAMEG00000014027/ENSAMEG00000004644/ENSAMEG00000011356/ENSAMEG00000019969/ENSAMEG00000016931/ENSAMEG00000017399/ENSAMEG00000007264/ENSAMEG00000017676/ENSAMEG00000012349/ENSAMEG00000004730/ENSAMEG00000007270/ENSAMEG00000014020/ENSAMEG00000015966/ENSAMEG00000013932/ENSAMEG00000019379/ENSAMEG00000017519/ENSAMEG00000001703/ENSAMEG00000019255/ENSAMEG00000015424/ENSAMEG00000004917/ENSAMEG00000001463/ENSAMEG00000012068/ENSAMEG00000010878/ENSAMEG00000000605/ENSAMEG00000003764/ENSAMEG00000014251/ENSAMEG00000004630/ENSAMEG00000001269 | 41 |
| GO:0005747 | mitochondrial respiratory chain complex I | 2.00E-06 | 1.17E-03 | 1.13E-03 | ENSAMEG00000002294/ENSAMEG00000010954/ENSAMEG00000003326/ENSAMEG00000010344/ENSAMEG00000007918/ENSAMEG00000010930/ENSAMEG00000014837/ENSAMEG00000015852/ENSAMEG00000011040/ENSAMEG00000004489/ENSAMEG00000011470/ENSAMEG00000013027/ENSAMEG00000004761 | 13 |
| GO:0032981 | mitochondrial respiratory chain complex I assembly | 5.08E-06 | 2.24E-03 | 2.16E-03 | ENSAMEG00000010954/ENSAMEG00000003326/ENSAMEG00000010344/ENSAMEG00000007918/ENSAMEG00000014837/ENSAMEG00000015852/ENSAMEG00000011040/ENSAMEG00000011470/ENSAMEG00000013027/ENSAMEG00000004761/ENSAMEG00000002795/ENSAMEG00000012966 | 12 |
| GO:0002376 | immune system process | 6.66E-06 | 2.34E-03 | 2.26E-03 | ENSAMEG00000002361/ENSAMEG00000002390/ENSAMEG00000002352/ENSAMEG00000002342/ENSAMEG00000002099/ENSAMEG00000001714/ENSAMEG00000001961/ENSAMEG00000017583/ENSAMEG00000019126/ENSAMEG00000018483/ENSAMEG00000001952 | 11 |
| GO:0042613 | MHC class II protein complex | 1.00E-05 | 2.56E-03 | 2.47E-03 | ENSAMEG00000002361/ENSAMEG00000002390/ENSAMEG00000002352/ENSAMEG00000002342/ENSAMEG00000004654/ENSAMEG00000002099/ENSAMEG00000001952 | 7 |
| GO:0022900 | electron transport chain | 1.02E-05 | 2.56E-03 | 2.47E-03 | ENSAMEG00000001051/ENSAMEG00000017762/ENSAMEG00000003502/ENSAMEG00000015234/ENSAMEG00000011765/ENSAMEG00000014373/ENSAMEG00000013441/ENSAMEG00000006153/ENSAMEG00000018274/ENSAMEG00000006052/ENSAMEG00000005242/ENSAMEG00000006540/ENSAMEG00000011554/ENSAMEG00000009142/ENSAMEG00000018091 | 15 |
| GO:0019882 | antigen processing and presentation | 1.70E-05 | 3.75E-03 | 3.61E-03 | ENSAMEG00000002361/ENSAMEG00000002390/ENSAMEG00000002352/ENSAMEG00000002342/ENSAMEG00000004654/ENSAMEG00000002099/ENSAMEG00000002026/ENSAMEG00000001714/ENSAMEG00000012673/ENSAMEG00000001952 | 10 |
| GO:0002504 | antigen processing and presentation of peptide or polysaccharide antigen via MHC class II | 7.76E-05 | 1.50E-02 | 1.45E-02 | ENSAMEG00000002361/ENSAMEG00000002390/ENSAMEG00000002352/ENSAMEG00000002342/ENSAMEG00000002099/ENSAMEG00000001952 | 6 |
| GO:0006412 | translation | 8.51E-05 | 1.50E-02 | 1.45E-02 | ENSAMEG00000006602/ENSAMEG00000015647/ENSAMEG00000019284/ENSAMEG00000008470/ENSAMEG00000011876/ENSAMEG00000011918/ENSAMEG00000004583/ENSAMEG00000011963/ENSAMEG00000016537/ENSAMEG00000002153/ENSAMEG00000011350/ENSAMEG00000014954/ENSAMEG00000004181/ENSAMEG00000014027/ENSAMEG00000004644/ENSAMEG00000011356/ENSAMEG00000019969/ENSAMEG00000016931/ENSAMEG00000017399/ENSAMEG00000010168/ENSAMEG00000017676/ENSAMEG00000012349/ENSAMEG00000004730/ENSAMEG00000007270/ENSAMEG00000014020/ENSAMEG00000015966/ENSAMEG00000013932/ENSAMEG00000019379/ENSAMEG00000017519/ENSAMEG00000001703/ENSAMEG00000019255/ENSAMEG00000015424/ENSAMEG00000004917/ENSAMEG00000001463/ENSAMEG00000012068/ENSAMEG00000010878/ENSAMEG00000000605/ENSAMEG00000003764/ENSAMEG00000014251/ENSAMEG00000004630/ENSAMEG00000001269 | 41 |
