# Supplementary material for: Gene expression profiles during postnatal development of the liver and pancreas in giant pandas
Source: Aging (Albany NY). 2020 Aug 15;12(15):15705–29. doi: 10.18632/aging.103783 (PMC7467380; doi:10.18632/aging.103783)
Supplement: Supplementary Table 20 [file aging-12-103783-s002..docx]

**Supplementary Table 20. Significantly enriched KEGG pathways for up-regulated DEGs in pancreas adult group compared with pancreas no feeding group.**

| **ID** | **Description** | **pvalue** | **p.adjust** | **qvalue** | **geneID** | **Count** |
| --- | --- | --- | --- | --- | --- | --- |
| aml03010 | Ribosome [PATH:aml03010] | 2.10E-11 | 6.37E-09 | 5.76E-09 | ENSAMEG00000015647/ENSAMEG00000008470/ENSAMEG00000011876/ENSAMEG00000011918/ENSAMEG00000004583/ENSAMEG00000016537/ENSAMEG00000011350/ENSAMEG00000014954/ENSAMEG00000004181/ENSAMEG00000014027/ENSAMEG00000000051/ENSAMEG00000004644/ENSAMEG00000011356/ENSAMEG00000017399/ENSAMEG00000008591/ENSAMEG00000017676/ENSAMEG00000001047/ENSAMEG00000012349/ENSAMEG00000004730/ENSAMEG00000014020/ENSAMEG00000015966/ENSAMEG00000013932/ENSAMEG00000019379/ENSAMEG00000001703/ENSAMEG00000004917/ENSAMEG00000001463/ENSAMEG00000012068/ENSAMEG00000010878/ENSAMEG00000000605/ENSAMEG00000003764/ENSAMEG00000014251/ENSAMEG00000004630/ENSAMEG00000001269 | 33 |
| aml00190 | Oxidative phosphorylation [PATH:aml00190] | 4.39E-10 | 6.65E-08 | 6.00E-08 | ENSAMEG00000002294/ENSAMEG00000010954/ENSAMEG00000001230/ENSAMEG00000001051/ENSAMEG00000017762/ENSAMEG00000003502/ENSAMEG00000005090/ENSAMEG00000003326/ENSAMEG00000010344/ENSAMEG00000007918/ENSAMEG00000010930/ENSAMEG00000007287/ENSAMEG00000015852/ENSAMEG00000011040/ENSAMEG00000013441/ENSAMEG00000004489/ENSAMEG00000006153/ENSAMEG00000018274/ENSAMEG00000009115/ENSAMEG00000011283/ENSAMEG00000011470/ENSAMEG00000017980/ENSAMEG00000005242/ENSAMEG00000013945/ENSAMEG00000002182/ENSAMEG00000009428/ENSAMEG00000004761/ENSAMEG00000018091/ENSAMEG00000002493/ENSAMEG00000003731 | 30 |
| aml04932 | Non-alcoholic fatty liver disease (NAFLD) [PATH:aml04932] | 4.84E-08 | 4.89E-06 | 4.42E-06 | ENSAMEG00000002294/ENSAMEG00000010954/ENSAMEG00000019175/ENSAMEG00000001051/ENSAMEG00000017762/ENSAMEG00000003502/ENSAMEG00000003326/ENSAMEG00000010344/ENSAMEG00000007918/ENSAMEG00000010930/ENSAMEG00000007287/ENSAMEG00000015852/ENSAMEG00000013275/ENSAMEG00000011040/ENSAMEG00000007766/ENSAMEG00000013441/ENSAMEG00000004489/ENSAMEG00000006153/ENSAMEG00000018274/ENSAMEG00000011470/ENSAMEG00000005242/ENSAMEG00000013945/ENSAMEG00000002182/ENSAMEG00000009428/ENSAMEG00000004761/ENSAMEG00000018091/ENSAMEG00000002493/ENSAMEG00000003731 | 28 |
| aml04714 | Thermogenesis [PATH:aml04714] | 1.12E-06 | 7.50E-05 | 6.77E-05 | ENSAMEG00000002456/ENSAMEG00000000376/ENSAMEG00000002294/ENSAMEG00000010954/ENSAMEG00000001051/ENSAMEG00000017762/ENSAMEG00000003502/ENSAMEG00000006919/ENSAMEG00000003326/ENSAMEG00000010344/ENSAMEG00000007918/ENSAMEG00000010930/ENSAMEG00000007287/ENSAMEG00000018818/ENSAMEG00000015852/ENSAMEG00000011040/ENSAMEG00000006865/ENSAMEG00000013441/ENSAMEG00000004489/ENSAMEG00000006153/ENSAMEG00000018274/ENSAMEG00000009115/ENSAMEG00000011470/ENSAMEG00000003368/ENSAMEG00000005242/ENSAMEG00000013945/ENSAMEG00000002182/ENSAMEG00000017702/ENSAMEG00000009428/ENSAMEG00000013819/ENSAMEG00000004761/ENSAMEG00000018091/ENSAMEG00000002493/ENSAMEG00000012966/ENSAMEG00000003731 | 35 |
| aml04612 | Antigen processing and presentation [PATH:aml04612] | 1.24E-06 | 7.50E-05 | 6.77E-05 | ENSAMEG00000002390/ENSAMEG00000002352/ENSAMEG00000002342/ENSAMEG00000004654/ENSAMEG00000002099/ENSAMEG00000001714/ENSAMEG00000002302/ENSAMEG00000001976/ENSAMEG00000016394/ENSAMEG00000004634/ENSAMEG00000016433/ENSAMEG00000014772/ENSAMEG00000018827/ENSAMEG00000006251/ENSAMEG00000001952 | 15 |
| aml05169 | Epstein-Barr virus infection [PATH:aml05169] | 1.06E-04 | 4.86E-03 | 4.39E-03 | ENSAMEG00000002456/ENSAMEG00000002390/ENSAMEG00000002352/ENSAMEG00000002342/ENSAMEG00000002099/ENSAMEG00000016296/ENSAMEG00000000376/ENSAMEG00000001714/ENSAMEG00000002302/ENSAMEG00000015629/ENSAMEG00000001976/ENSAMEG00000000266/ENSAMEG00000012673/ENSAMEG00000013275/ENSAMEG00000017735/ENSAMEG00000007766/ENSAMEG00000016347/ENSAMEG00000018412/ENSAMEG00000015000/ENSAMEG00000004221/ENSAMEG00000010613/ENSAMEG00000008781/ENSAMEG00000004529/ENSAMEG00000017232/ENSAMEG00000007891/ENSAMEG00000001952 | 26 |
| aml05012 | Parkinson disease [PATH:aml05012] | 1.12E-04 | 4.86E-03 | 4.39E-03 | ENSAMEG00000002294/ENSAMEG00000010954/ENSAMEG00000001051/ENSAMEG00000012526/ENSAMEG00000017762/ENSAMEG00000003502/ENSAMEG00000003326/ENSAMEG00000010344/ENSAMEG00000007918/ENSAMEG00000010930/ENSAMEG00000007287/ENSAMEG00000015852/ENSAMEG00000011040/ENSAMEG00000011224/ENSAMEG00000013441/ENSAMEG00000004489/ENSAMEG00000011356/ENSAMEG00000006153/ENSAMEG00000018274/ENSAMEG00000009115/ENSAMEG00000011470/ENSAMEG00000014519/ENSAMEG00000005242/ENSAMEG00000013945/ENSAMEG00000002182/ENSAMEG00000016075/ENSAMEG00000003000/ENSAMEG00000009428/ENSAMEG00000004761/ENSAMEG00000018091/ENSAMEG00000002493/ENSAMEG00000003731 | 32 |
| aml05332 | Graft-versus-host disease [PATH:aml05332] | 1.44E-04 | 5.46E-03 | 4.94E-03 | ENSAMEG00000002390/ENSAMEG00000002352/ENSAMEG00000002342/ENSAMEG00000002099/ENSAMEG00000001714/ENSAMEG00000002302/ENSAMEG00000004634/ENSAMEG00000001952 | 8 |
| aml05164 | Influenza A [PATH:aml05164] | 2.17E-04 | 7.32E-03 | 6.61E-03 | ENSAMEG00000002390/ENSAMEG00000002352/ENSAMEG00000002342/ENSAMEG00000013261/ENSAMEG00000006334/ENSAMEG00000002099/ENSAMEG00000006262/ENSAMEG00000019175/ENSAMEG00000005780/ENSAMEG00000015629/ENSAMEG00000007766/ENSAMEG00000013494/ENSAMEG00000016347/ENSAMEG00000015000/ENSAMEG00000006252/ENSAMEG00000004043/ENSAMEG00000010613/ENSAMEG00000008781/ENSAMEG00000014772/ENSAMEG00000017232/ENSAMEG00000002212/ENSAMEG00000001952 | 22 |
| aml04974 | Protein digestion and absorption [PATH:aml04974] | 5.79E-04 | 1.65E-02 | 1.49E-02 | ENSAMEG00000010850/ENSAMEG00000006334/ENSAMEG00000003734/ENSAMEG00000017576/ENSAMEG00000006262/ENSAMEG00000002069/ENSAMEG00000015921/ENSAMEG00000005506/ENSAMEG00000010873/ENSAMEG00000003271/ENSAMEG00000020287/ENSAMEG00000005071/ENSAMEG00000014546/ENSAMEG00000007631/ENSAMEG00000003817/ENSAMEG00000001740 | 16 |
| aml05016 | Huntington disease [PATH:aml05016] | 6.00E-04 | 1.65E-02 | 1.49E-02 | ENSAMEG00000002294/ENSAMEG00000010954/ENSAMEG00000001051/ENSAMEG00000012526/ENSAMEG00000000993/ENSAMEG00000017762/ENSAMEG00000003502/ENSAMEG00000003326/ENSAMEG00000010344/ENSAMEG00000007918/ENSAMEG00000010930/ENSAMEG00000007287/ENSAMEG00000015852/ENSAMEG00000013275/ENSAMEG00000016461/ENSAMEG00000011040/ENSAMEG00000013441/ENSAMEG00000004489/ENSAMEG00000006153/ENSAMEG00000018274/ENSAMEG00000009115/ENSAMEG00000011470/ENSAMEG00000005242/ENSAMEG00000013945/ENSAMEG00000002182/ENSAMEG00000016075/ENSAMEG00000009428/ENSAMEG00000004761/ENSAMEG00000018091/ENSAMEG00000015480/ENSAMEG00000002493/ENSAMEG00000017758/ENSAMEG00000014592/ENSAMEG00000003731 | 34 |
| aml04972 | Pancreatic secretion [PATH:aml04972] | 6.63E-04 | 1.67E-02 | 1.51E-02 | ENSAMEG00000013928/ENSAMEG00000004305/ENSAMEG00000010850/ENSAMEG00000006334/ENSAMEG00000003734/ENSAMEG00000017576/ENSAMEG00000006262/ENSAMEG00000001684/ENSAMEG00000002069/ENSAMEG00000008518/ENSAMEG00000005506/ENSAMEG00000014004/ENSAMEG00000010873/ENSAMEG00000005071/ENSAMEG00000014546/ENSAMEG00000005195 | 16 |
| aml04940 | Type I diabetes mellitus [PATH:aml04940] | 1.20E-03 | 2.72E-02 | 2.46E-02 | ENSAMEG00000002390/ENSAMEG00000002352/ENSAMEG00000002342/ENSAMEG00000001325/ENSAMEG00000002099/ENSAMEG00000001714/ENSAMEG00000002302/ENSAMEG00000001952 | 8 |
| aml05330 | Allograft rejection [PATH:aml05330] | 1.29E-03 | 2.72E-02 | 2.46E-02 | ENSAMEG00000002390/ENSAMEG00000002352/ENSAMEG00000002342/ENSAMEG00000002099/ENSAMEG00000001714/ENSAMEG00000002302/ENSAMEG00000001952 | 7 |
| aml04260 | Cardiac muscle contraction [PATH:aml04260] | 1.35E-03 | 2.72E-02 | 2.46E-02 | ENSAMEG00000011561/ENSAMEG00000003734/ENSAMEG00000001051/ENSAMEG00000017762/ENSAMEG00000003502/ENSAMEG00000005071/ENSAMEG00000007287/ENSAMEG00000013441/ENSAMEG00000006153/ENSAMEG00000018274/ENSAMEG00000005242/ENSAMEG00000013945/ENSAMEG00000018091/ENSAMEG00000003731 | 14 |
| aml04723 | Retrograde endocannabinoid signaling [PATH:aml04723] | 1.76E-03 | 3.33E-02 | 3.01E-02 | ENSAMEG00000002456/ENSAMEG00000000376/ENSAMEG00000002294/ENSAMEG00000010954/ENSAMEG00000004527/ENSAMEG00000003326/ENSAMEG00000010344/ENSAMEG00000007918/ENSAMEG00000010930/ENSAMEG00000015852/ENSAMEG00000011040/ENSAMEG00000004489/ENSAMEG00000011470/ENSAMEG00000002182/ENSAMEG00000009428/ENSAMEG00000004761/ENSAMEG00000008528/ENSAMEG00000002493/ENSAMEG00000013963/ENSAMEG00000006749 | 20 |
| aml04142 | Lysosome [PATH:aml04142] | 2.36E-03 | 4.21E-02 | 3.80E-02 | ENSAMEG00000012483/ENSAMEG00000001230/ENSAMEG00000016812/ENSAMEG00000003106/ENSAMEG00000007876/ENSAMEG00000010968/ENSAMEG00000000312/ENSAMEG00000010020/ENSAMEG00000015418/ENSAMEG00000017980/ENSAMEG00000004770/ENSAMEG00000015113/ENSAMEG00000011117/ENSAMEG00000008287/ENSAMEG00000013409/ENSAMEG00000002787/ENSAMEG00000006251/ENSAMEG00000012013 | 18 |
