# Supplementary material for: Gene expression profiles during postnatal development of the liver and pancreas in giant pandas
Source: Aging (Albany NY). 2020 Aug 15;12(15):15705–29. doi: 10.18632/aging.103783 (PMC7467380; doi:10.18632/aging.103783)
Supplement: Supplementary Table 21 [file aging-12-103783-s015..docx]

**Supplementary Table 21. Significantly enriched GO categories for down-regulated DEGs in pancreas adult group compared with pancreas no feeding group.**

| **ID** | **Description** | **pvalue** | **p.adjust** | **qvalue** | **geneID** | **Count** |
| --- | --- | --- | --- | --- | --- | --- |
| GO:0005604 | basement membrane | 1.05E-10 | 2.02E-07 | 1.84E-07 | ENSAMEG00000003517/ENSAMEG00000012108/ENSAMEG00000001577/ENSAMEG00000003184/ENSAMEG00000000748/ENSAMEG00000018103/ENSAMEG00000016892/ENSAMEG00000016281/ENSAMEG00000017465/ENSAMEG00000001889/ENSAMEG00000002289/ENSAMEG00000015656/ENSAMEG00000002001/ENSAMEG00000010741/ENSAMEG00000016747/ENSAMEG00000004392/ENSAMEG00000007475/ENSAMEG00000012067/ENSAMEG00000000609/ENSAMEG00000005463/ENSAMEG00000016573 | 21 |
| GO:0005201 | extracellular matrix structural constituent | 1.28E-08 | 1.22E-05 | 1.12E-05 | ENSAMEG00000011626/ENSAMEG00000011903/ENSAMEG00000001303/ENSAMEG00000017486/ENSAMEG00000015429/ENSAMEG00000012108/ENSAMEG00000016642/ENSAMEG00000001577/ENSAMEG00000011644/ENSAMEG00000014718/ENSAMEG00000016892/ENSAMEG00000002289/ENSAMEG00000010741/ENSAMEG00000012067 | 14 |
| GO:0090307 | mitotic spindle assembly | 2.14E-08 | 1.36E-05 | 1.25E-05 | ENSAMEG00000013678/ENSAMEG00000014099/ENSAMEG00000001187/ENSAMEG00000001637/ENSAMEG00000003831/ENSAMEG00000001458/ENSAMEG00000002862/ENSAMEG00000017740/ENSAMEG00000008484/ENSAMEG00000005671/ENSAMEG00000015929/ENSAMEG00000015353/ENSAMEG00000004613 | 13 |
| GO:0030199 | collagen fibril organization | 4.16E-08 | 1.87E-05 | 1.71E-05 | ENSAMEG00000011626/ENSAMEG00000011903/ENSAMEG00000017486/ENSAMEG00000007857/ENSAMEG00000008993/ENSAMEG00000018055/ENSAMEG00000011644/ENSAMEG00000014718/ENSAMEG00000002911/ENSAMEG00000017004/ENSAMEG00000005313/ENSAMEG00000010741/ENSAMEG00000002331/ENSAMEG00000017807 | 14 |
| GO:0062023 | collagen-containing extracellular matrix | 5.13E-08 | 1.87E-05 | 1.71E-05 | ENSAMEG00000003517/ENSAMEG00000011626/ENSAMEG00000000126/ENSAMEG00000015429/ENSAMEG00000007909/ENSAMEG00000008993/ENSAMEG00000001577/ENSAMEG00000014718/ENSAMEG00000002750/ENSAMEG00000012439/ENSAMEG00000009506/ENSAMEG00000017465/ENSAMEG00000010136/ENSAMEG00000003815/ENSAMEG00000010741/ENSAMEG00000004454/ENSAMEG00000007837/ENSAMEG00000002331/ENSAMEG00000015203/ENSAMEG00000002854/ENSAMEG00000019198/ENSAMEG00000016573 | 22 |
| GO:0031012 | extracellular matrix | 5.86E-08 | 1.87E-05 | 1.71E-05 | ENSAMEG00000003517/ENSAMEG00000011626/ENSAMEG00000011903/ENSAMEG00000001604/ENSAMEG00000007909/ENSAMEG00000008993/ENSAMEG00000018055/ENSAMEG00000007055/ENSAMEG00000001577/ENSAMEG00000018103/ENSAMEG00000008399/ENSAMEG00000017465/ENSAMEG00000008251/ENSAMEG00000013522/ENSAMEG00000014237/ENSAMEG00000008850/ENSAMEG00000008895/ENSAMEG00000013801/ENSAMEG00000017907/ENSAMEG00000010741/ENSAMEG00000011584/ENSAMEG00000007837/ENSAMEG00000002331/ENSAMEG00000017143/ENSAMEG00000005051/ENSAMEG00000000609/ENSAMEG00000016417 | 27 |
| GO:0008017 | microtubule binding | 7.70E-08 | 1.88E-05 | 1.72E-05 | ENSAMEG00000012031/ENSAMEG00000016438/ENSAMEG00000014099/ENSAMEG00000001093/ENSAMEG00000018397/ENSAMEG00000014095/ENSAMEG00000011957/ENSAMEG00000007235/ENSAMEG00000009205/ENSAMEG00000012038/ENSAMEG00000016762/ENSAMEG00000001458/ENSAMEG00000015215/ENSAMEG00000004686/ENSAMEG00000014825/ENSAMEG00000013667/ENSAMEG00000014232/ENSAMEG00000014549/ENSAMEG00000011462/ENSAMEG00000003927/ENSAMEG00000006673/ENSAMEG00000005069/ENSAMEG00000000223/ENSAMEG00000016992/ENSAMEG00000000285/ENSAMEG00000018442/ENSAMEG00000009934/ENSAMEG00000005084/ENSAMEG00000003586/ENSAMEG00000005671/ENSAMEG00000012919/ENSAMEG00000002966/ENSAMEG00000014201/ENSAMEG00000000908/ENSAMEG00000010358/ENSAMEG00000015353/ENSAMEG00000008024/ENSAMEG00000012636 | 38 |
| GO:0000281 | mitotic cytokinesis | 7.85E-08 | 1.88E-05 | 1.72E-05 | ENSAMEG00000016438/ENSAMEG00000001093/ENSAMEG00000015972/ENSAMEG00000002139/ENSAMEG00000009390/ENSAMEG00000009205/ENSAMEG00000011827/ENSAMEG00000016762/ENSAMEG00000010298/ENSAMEG00000000690/ENSAMEG00000016838/ENSAMEG00000014232/ENSAMEG00000014201/ENSAMEG00000003412/ENSAMEG00000015353/ENSAMEG00000012636 | 16 |
| GO:0000776 | kinetochore | 1.62E-07 | 3.44E-05 | 3.14E-05 | ENSAMEG00000001187/ENSAMEG00000014095/ENSAMEG00000010069/ENSAMEG00000012771/ENSAMEG00000017203/ENSAMEG00000009491/ENSAMEG00000006525/ENSAMEG00000000371/ENSAMEG00000017537/ENSAMEG00000017084/ENSAMEG00000009168/ENSAMEG00000005342/ENSAMEG00000016838/ENSAMEG00000014232/ENSAMEG00000014549/ENSAMEG00000002580/ENSAMEG00000002642/ENSAMEG00000005084/ENSAMEG00000005671/ENSAMEG00000014641/ENSAMEG00000006979/ENSAMEG00000013280 | 22 |
| GO:0005813 | centrosome | 2.13E-07 | 4.08E-05 | 3.73E-05 | ENSAMEG00000001187/ENSAMEG00000010069/ENSAMEG00000009390/ENSAMEG00000001259/ENSAMEG00000002675/ENSAMEG00000003520/ENSAMEG00000011012/ENSAMEG00000017945/ENSAMEG00000005225/ENSAMEG00000011827/ENSAMEG00000018213/ENSAMEG00000004603/ENSAMEG00000011443/ENSAMEG00000017795/ENSAMEG00000013598/ENSAMEG00000017740/ENSAMEG00000002666/ENSAMEG00000007168/ENSAMEG00000012346/ENSAMEG00000008991/ENSAMEG00000000013/ENSAMEG00000010721/ENSAMEG00000004449/ENSAMEG00000000953/ENSAMEG00000014232/ENSAMEG00000014631/ENSAMEG00000013264/ENSAMEG00000002590/ENSAMEG00000004713/ENSAMEG00000008931/ENSAMEG00000017811/ENSAMEG00000004999/ENSAMEG00000012375/ENSAMEG00000000490/ENSAMEG00000015514/ENSAMEG00000005084/ENSAMEG00000002356/ENSAMEG00000018002/ENSAMEG00000015773/ENSAMEG00000003586/ENSAMEG00000003208/ENSAMEG00000017415/ENSAMEG00000009312/ENSAMEG00000002966/ENSAMEG00000014201/ENSAMEG00000011164/ENSAMEG00000005940/ENSAMEG00000010293/ENSAMEG00000001855/ENSAMEG00000015929/ENSAMEG00000002634/ENSAMEG00000009793/ENSAMEG00000017786/ENSAMEG00000003843/ENSAMEG00000008333/ENSAMEG00000008024/ENSAMEG00000013567/ENSAMEG00000003215/ENSAMEG00000000983/ENSAMEG00000003720/ENSAMEG00000006979/ENSAMEG00000013280/ENSAMEG00000003767/ENSAMEG00000010512 | 64 |
| GO:0007051 | spindle organization | 4.68E-07 | 8.14E-05 | 7.44E-05 | ENSAMEG00000014099/ENSAMEG00000017540/ENSAMEG00000017084/ENSAMEG00000007168/ENSAMEG00000000690/ENSAMEG00000002580/ENSAMEG00000010293/ENSAMEG00000008333/ENSAMEG00000006979 | 9 |
| GO:0006260 | DNA replication | 1.20E-06 | 1.92E-04 | 1.75E-04 | ENSAMEG00000013104/ENSAMEG00000005841/ENSAMEG00000001634/ENSAMEG00000013454/ENSAMEG00000014998/ENSAMEG00000000475/ENSAMEG00000018347/ENSAMEG00000013505/ENSAMEG00000009254/ENSAMEG00000014758/ENSAMEG00000018028/ENSAMEG00000018281/ENSAMEG00000017258/ENSAMEG00000001820/ENSAMEG00000005102/ENSAMEG00000009236/ENSAMEG00000015091/ENSAMEG00000013843/ENSAMEG00000011711/ENSAMEG00000005940/ENSAMEG00000002731 | 21 |
| GO:0001764 | neuron migration | 2.18E-06 | 3.22E-04 | 2.94E-04 | ENSAMEG00000017540/ENSAMEG00000006908/ENSAMEG00000002139/ENSAMEG00000015074/ENSAMEG00000016040/ENSAMEG00000003994/ENSAMEG00000004633/ENSAMEG00000016510/ENSAMEG00000012157/ENSAMEG00000000572/ENSAMEG00000003213/ENSAMEG00000012019/ENSAMEG00000006858/ENSAMEG00000005781/ENSAMEG00000000609/ENSAMEG00000015975/ENSAMEG00000016138/ENSAMEG00000005717 | 18 |
| GO:0000922 | spindle pole | 3.03E-06 | 4.15E-04 | 3.79E-04 | ENSAMEG00000013678/ENSAMEG00000014099/ENSAMEG00000017540/ENSAMEG00000014771/ENSAMEG00000001187/ENSAMEG00000013777/ENSAMEG00000007868/ENSAMEG00000004686/ENSAMEG00000010721/ENSAMEG00000000953/ENSAMEG00000014232/ENSAMEG00000008012/ENSAMEG00000004713/ENSAMEG00000001860/ENSAMEG00000015514/ENSAMEG00000010293/ENSAMEG00000006979/ENSAMEG00000003767/ENSAMEG00000010512 | 19 |
| GO:0005581 | collagen trimer | 3.39E-06 | 4.32E-04 | 3.95E-04 | ENSAMEG00000011626/ENSAMEG00000011903/ENSAMEG00000017486/ENSAMEG00000012108/ENSAMEG00000018055/ENSAMEG00000016642/ENSAMEG00000011644/ENSAMEG00000004114/ENSAMEG00000009885/ENSAMEG00000016892/ENSAMEG00000010741/ENSAMEG00000004392/ENSAMEG00000012067 | 13 |
| GO:0005876 | spindle microtubule | 6.08E-06 | 7.27E-04 | 6.65E-04 | ENSAMEG00000012031/ENSAMEG00000014099/ENSAMEG00000004841/ENSAMEG00000003520/ENSAMEG00000016762/ENSAMEG00000004686/ENSAMEG00000007168/ENSAMEG00000014232/ENSAMEG00000014631/ENSAMEG00000005084/ENSAMEG00000014641 | 11 |
| GO:0051965 | positive regulation of synapse assembly | 7.44E-06 | 7.74E-04 | 7.07E-04 | ENSAMEG00000016009/ENSAMEG00000003994/ENSAMEG00000018950/ENSAMEG00000013498/ENSAMEG00000019328/ENSAMEG00000016005/ENSAMEG00000012949/ENSAMEG00000013799/ENSAMEG00000017786/ENSAMEG00000014794 | 10 |
| GO:0006974 | cellular response to DNA damage stimulus | 7.50E-06 | 7.74E-04 | 7.07E-04 | ENSAMEG00000007989/ENSAMEG00000009390/ENSAMEG00000001259/ENSAMEG00000002586/ENSAMEG00000000409/ENSAMEG00000012463/ENSAMEG00000000171/ENSAMEG00000001064/ENSAMEG00000000352/ENSAMEG00000013505/ENSAMEG00000016965/ENSAMEG00000011892/ENSAMEG00000016051/ENSAMEG00000001323/ENSAMEG00000004713/ENSAMEG00000005534/ENSAMEG00000006254/ENSAMEG00000013902/ENSAMEG00000015091/ENSAMEG00000004483/ENSAMEG00000017539/ENSAMEG00000016741/ENSAMEG00000010605/ENSAMEG00000004283/ENSAMEG00000013843/ENSAMEG00000007872/ENSAMEG00000008484/ENSAMEG00000012375/ENSAMEG00000016250/ENSAMEG00000017063/ENSAMEG00000015826/ENSAMEG00000009312/ENSAMEG00000003946/ENSAMEG00000006324/ENSAMEG00000015491/ENSAMEG00000012636/ENSAMEG00000011912/ENSAMEG00000010974/ENSAMEG00000003572 | 39 |
| GO:0007155 | cell adhesion | 7.68E-06 | 7.74E-04 | 7.07E-04 | ENSAMEG00000003517/ENSAMEG00000017136/ENSAMEG00000000126/ENSAMEG00000007091/ENSAMEG00000006455/ENSAMEG00000016880/ENSAMEG00000009794/ENSAMEG00000012971/ENSAMEG00000004995/ENSAMEG00000010273/ENSAMEG00000016273/ENSAMEG00000018131/ENSAMEG00000011916/ENSAMEG00000000087/ENSAMEG00000018950/ENSAMEG00000002289/ENSAMEG00000016249/ENSAMEG00000012157/ENSAMEG00000001479/ENSAMEG00000012004/ENSAMEG00000000572/ENSAMEG00000017907/ENSAMEG00000010741/ENSAMEG00000016747/ENSAMEG00000003213/ENSAMEG00000007738/ENSAMEG00000004392/ENSAMEG00000008575/ENSAMEG00000012952/ENSAMEG00000016517/ENSAMEG00000005964/ENSAMEG00000010800/ENSAMEG00000010408/ENSAMEG00000007475/ENSAMEG00000008296/ENSAMEG00000003496/ENSAMEG00000007537/ENSAMEG00000016005/ENSAMEG00000013866/ENSAMEG00000004693/ENSAMEG00000013507/ENSAMEG00000000779/ENSAMEG00000004187 | 43 |
| GO:0050840 | extracellular matrix binding | 1.44E-05 | 1.38E-03 | 1.26E-03 | ENSAMEG00000001604/ENSAMEG00000007909/ENSAMEG00000009506/ENSAMEG00000008895/ENSAMEG00000003815/ENSAMEG00000017907/ENSAMEG00000014603/ENSAMEG00000005051 | 8 |
| GO:0000070 | mitotic sister chromatid segregation | 2.37E-05 | 2.16E-03 | 1.97E-03 | ENSAMEG00000001187/ENSAMEG00000017084/ENSAMEG00000012346/ENSAMEG00000005342/ENSAMEG00000016838/ENSAMEG00000014232/ENSAMEG00000005671 | 7 |
| GO:0006270 | DNA replication initiation | 2.66E-05 | 2.31E-03 | 2.12E-03 | ENSAMEG00000005841/ENSAMEG00000001634/ENSAMEG00000014998/ENSAMEG00000011443/ENSAMEG00000007868/ENSAMEG00000013505/ENSAMEG00000010605/ENSAMEG00000009668 | 8 |
| GO:0000724 | double-strand break repair via homologous recombination | 3.20E-05 | 2.66E-03 | 2.43E-03 | ENSAMEG00000009390/ENSAMEG00000012463/ENSAMEG00000000171/ENSAMEG00000016965/ENSAMEG00000002666/ENSAMEG00000005645/ENSAMEG00000008475/ENSAMEG00000001323/ENSAMEG00000015678/ENSAMEG00000002533/ENSAMEG00000013902/ENSAMEG00000016741/ENSAMEG00000010605/ENSAMEG00000010293/ENSAMEG00000004921 | 15 |
| GO:0007059 | chromosome segregation | 3.93E-05 | 3.10E-03 | 2.83E-03 | ENSAMEG00000007989/ENSAMEG00000001187/ENSAMEG00000010069/ENSAMEG00000016113/ENSAMEG00000009491/ENSAMEG00000000371/ENSAMEG00000017537/ENSAMEG00000017084/ENSAMEG00000000171/ENSAMEG00000000137/ENSAMEG00000012346/ENSAMEG00000016838/ENSAMEG00000013933/ENSAMEG00000014641 | 14 |
| GO:0042060 | wound healing | 4.05E-05 | 3.10E-03 | 2.83E-03 | ENSAMEG00000003517/ENSAMEG00000011626/ENSAMEG00000005313/ENSAMEG00000015456/ENSAMEG00000003213/ENSAMEG00000009982/ENSAMEG00000011584/ENSAMEG00000007537/ENSAMEG00000006743/ENSAMEG00000001993/ENSAMEG00000003383/ENSAMEG00000014162 | 12 |
| GO:0007094 | mitotic spindle assembly checkpoint | 4.73E-05 | 3.49E-03 | 3.19E-03 | ENSAMEG00000012771/ENSAMEG00000017203/ENSAMEG00000013777/ENSAMEG00000014232/ENSAMEG00000002580/ENSAMEG00000016741/ENSAMEG00000005671 | 7 |
| GO:0090179 | planar cell polarity pathway involved in neural tube closure | 7.70E-05 | 5.46E-03 | 4.99E-03 | ENSAMEG00000002750/ENSAMEG00000003213/ENSAMEG00000009982/ENSAMEG00000011584/ENSAMEG00000018377/ENSAMEG00000014162 | 6 |
| GO:0007052 | mitotic spindle organization | 1.34E-04 | 9.14E-03 | 8.36E-03 | ENSAMEG00000010069/ENSAMEG00000016924/ENSAMEG00000010298/ENSAMEG00000007168/ENSAMEG00000000013/ENSAMEG00000004449/ENSAMEG00000000953/ENSAMEG00000005084/ENSAMEG00000015472 | 9 |
| GO:0035904 | aorta development | 1.51E-04 | 9.67E-03 | 8.84E-03 | ENSAMEG00000002139/ENSAMEG00000018055/ENSAMEG00000011127/ENSAMEG00000004387/ENSAMEG00000007837/ENSAMEG00000005699/ENSAMEG00000014745 | 7 |
| GO:0000400 | four-way junction DNA binding | 1.57E-04 | 9.67E-03 | 8.84E-03 | ENSAMEG00000002666/ENSAMEG00000006254/ENSAMEG00000013843/ENSAMEG00000016250/ENSAMEG00000006324/ENSAMEG00000016990 | 6 |
| GO:0007076 | mitotic chromosome condensation | 1.57E-04 | 9.67E-03 | 8.84E-03 | ENSAMEG00000000194/ENSAMEG00000014358/ENSAMEG00000008950/ENSAMEG00000005936/ENSAMEG00000007193/ENSAMEG00000002220 | 6 |
| GO:0005819 | spindle | 1.71E-04 | 1.02E-02 | 9.33E-03 | ENSAMEG00000013678/ENSAMEG00000016438/ENSAMEG00000014099/ENSAMEG00000002139/ENSAMEG00000016762/ENSAMEG00000017795/ENSAMEG00000004686/ENSAMEG00000013667/ENSAMEG00000016838/ENSAMEG00000014232/ENSAMEG00000001260/ENSAMEG00000016741/ENSAMEG00000014201 | 13 |
| GO:0000278 | mitotic cell cycle | 2.06E-04 | 1.20E-02 | 1.09E-02 | ENSAMEG00000000135/ENSAMEG00000009491/ENSAMEG00000014358/ENSAMEG00000000371/ENSAMEG00000017537/ENSAMEG00000007868/ENSAMEG00000007168/ENSAMEG00000012346/ENSAMEG00000014232/ENSAMEG00000001855/ENSAMEG00000009793/ENSAMEG00000014641/ENSAMEG00000000983/ENSAMEG00000013280 | 14 |
| GO:0016324 | apical plasma membrane | 2.28E-04 | 1.28E-02 | 1.17E-02 | ENSAMEG00000003517/ENSAMEG00000000655/ENSAMEG00000007091/ENSAMEG00000017954/ENSAMEG00000018312/ENSAMEG00000000864/ENSAMEG00000008339/ENSAMEG00000017883/ENSAMEG00000018206/ENSAMEG00000013870/ENSAMEG00000005255/ENSAMEG00000006743/ENSAMEG00000014610/ENSAMEG00000015975/ENSAMEG00000001586/ENSAMEG00000007018/ENSAMEG00000004798/ENSAMEG00000014162/ENSAMEG00000002620/ENSAMEG00000005330/ENSAMEG00000002410/ENSAMEG00000015459/ENSAMEG00000005706/ENSAMEG00000010368 | 24 |
| GO:0030496 | midbody | 3.01E-04 | 1.65E-02 | 1.50E-02 | ENSAMEG00000016438/ENSAMEG00000017540/ENSAMEG00000001093/ENSAMEG00000001187/ENSAMEG00000002139/ENSAMEG00000003520/ENSAMEG00000011012/ENSAMEG00000009205/ENSAMEG00000017795/ENSAMEG00000004686/ENSAMEG00000001082/ENSAMEG00000013667/ENSAMEG00000016838/ENSAMEG00000014232/ENSAMEG00000008012/ENSAMEG00000002580/ENSAMEG00000004713/ENSAMEG00000006552/ENSAMEG00000010291/ENSAMEG00000014201/ENSAMEG00000004750 | 21 |
| GO:0071230 | cellular response to amino acid stimulus | 3.23E-04 | 1.72E-02 | 1.57E-02 | ENSAMEG00000011626/ENSAMEG00000011903/ENSAMEG00000017486/ENSAMEG00000012108/ENSAMEG00000011644/ENSAMEG00000002443/ENSAMEG00000003994/ENSAMEG00000011823/ENSAMEG00000009623/ENSAMEG00000016417 | 10 |
| GO:0005887 | integral component of plasma membrane | 3.43E-04 | 1.78E-02 | 1.62E-02 | ENSAMEG00000000655/ENSAMEG00000006455/ENSAMEG00000001063/ENSAMEG00000016880/ENSAMEG00000018346/ENSAMEG00000008476/ENSAMEG00000009123/ENSAMEG00000009794/ENSAMEG00000014828/ENSAMEG00000003994/ENSAMEG00000019226/ENSAMEG00000018950/ENSAMEG00000015456/ENSAMEG00000003959/ENSAMEG00000016499/ENSAMEG00000004387/ENSAMEG00000000836/ENSAMEG00000000485/ENSAMEG00000008462/ENSAMEG00000007486/ENSAMEG00000008575/ENSAMEG00000010947/ENSAMEG00000006143/ENSAMEG00000000130/ENSAMEG00000014734/ENSAMEG00000016164/ENSAMEG00000005206/ENSAMEG00000000742/ENSAMEG00000017143/ENSAMEG00000018407/ENSAMEG00000015361/ENSAMEG00000000245/ENSAMEG00000016543/ENSAMEG00000009829/ENSAMEG00000017994/ENSAMEG00000015804/ENSAMEG00000016005/ENSAMEG00000007018/ENSAMEG00000000527/ENSAMEG00000005792/ENSAMEG00000016829/ENSAMEG00000007213/ENSAMEG00000003727/ENSAMEG00000016101/ENSAMEG00000005817/ENSAMEG00000018771/ENSAMEG00000003711/ENSAMEG00000009890/ENSAMEG00000017496/ENSAMEG00000014794/ENSAMEG00000020060/ENSAMEG00000013970/ENSAMEG00000005717/ENSAMEG00000005131/ENSAMEG00000013387/ENSAMEG00000000703/ENSAMEG00000013239/ENSAMEG00000016574/ENSAMEG00000005229/ENSAMEG00000020104 | 60 |
| GO:0007420 | brain development | 4.25E-04 | 2.13E-02 | 1.95E-02 | ENSAMEG00000017540/ENSAMEG00000012305/ENSAMEG00000006908/ENSAMEG00000002139/ENSAMEG00000009390/ENSAMEG00000016642/ENSAMEG00000004633/ENSAMEG00000005313/ENSAMEG00000001820/ENSAMEG00000000223/ENSAMEG00000012019/ENSAMEG00000010606/ENSAMEG00000016741/ENSAMEG00000015776/ENSAMEG00000017881/ENSAMEG00000018402/ENSAMEG00000003974/ENSAMEG00000006241/ENSAMEG00000005229 | 19 |
| GO:0010951 | negative regulation of endopeptidase activity | 4.34E-04 | 2.13E-02 | 1.95E-02 | ENSAMEG00000007857/ENSAMEG00000000748/ENSAMEG00000016040/ENSAMEG00000012851/ENSAMEG00000002160/ENSAMEG00000017004/ENSAMEG00000013869/ENSAMEG00000008251/ENSAMEG00000001041/ENSAMEG00000008201/ENSAMEG00000008012/ENSAMEG00000001036/ENSAMEG00000015644/ENSAMEG00000018209/ENSAMEG00000008108/ENSAMEG00000007712/ENSAMEG00000001045/ENSAMEG00000010204 | 18 |
| GO:0007411 | axon guidance | 4.59E-04 | 2.20E-02 | 2.01E-02 | ENSAMEG00000006908/ENSAMEG00000002139/ENSAMEG00000001345/ENSAMEG00000011012/ENSAMEG00000001384/ENSAMEG00000016510/ENSAMEG00000011584/ENSAMEG00000000223/ENSAMEG00000013702/ENSAMEG00000006574/ENSAMEG00000000609/ENSAMEG00000005792/ENSAMEG00000001611/ENSAMEG00000014794 | 14 |
| GO:0001736 | establishment of planar polarity | 4.97E-04 | 2.30E-02 | 2.11E-02 | ENSAMEG00000003213/ENSAMEG00000009982/ENSAMEG00000011584/ENSAMEG00000015975/ENSAMEG00000001611/ENSAMEG00000014162 | 6 |
| GO:0030198 | extracellular matrix organization | 5.06E-04 | 2.30E-02 | 2.11E-02 | ENSAMEG00000001604/ENSAMEG00000012108/ENSAMEG00000014718/ENSAMEG00000018103/ENSAMEG00000009506/ENSAMEG00000017465/ENSAMEG00000005313/ENSAMEG00000009966/ENSAMEG00000017907/ENSAMEG00000002331/ENSAMEG00000007537/ENSAMEG00000005051/ENSAMEG00000015873 | 13 |
| GO:0006281 | DNA repair | 5.48E-04 | 2.44E-02 | 2.23E-02 | ENSAMEG00000009390/ENSAMEG00000013934/ENSAMEG00000013454/ENSAMEG00000000171/ENSAMEG00000001064/ENSAMEG00000012174/ENSAMEG00000011341/ENSAMEG00000000475/ENSAMEG00000016965/ENSAMEG00000002666/ENSAMEG00000011892/ENSAMEG00000016051/ENSAMEG00000014825/ENSAMEG00000008438/ENSAMEG00000015678/ENSAMEG00000006254/ENSAMEG00000015091/ENSAMEG00000004483/ENSAMEG00000016741/ENSAMEG00000013843/ENSAMEG00000012375/ENSAMEG00000017063/ENSAMEG00000015826/ENSAMEG00000019971/ENSAMEG00000006324 | 25 |
| GO:0030425 | dendrite | 5.64E-04 | 2.46E-02 | 2.24E-02 | ENSAMEG00000016531/ENSAMEG00000001384/ENSAMEG00000015003/ENSAMEG00000005313/ENSAMEG00000018950/ENSAMEG00000013264/ENSAMEG00000016591/ENSAMEG00000011572/ENSAMEG00000006143/ENSAMEG00000000223/ENSAMEG00000013993/ENSAMEG00000008944/ENSAMEG00000015975/ENSAMEG00000009623/ENSAMEG00000017131/ENSAMEG00000012523/ENSAMEG00000016396/ENSAMEG00000014794/ENSAMEG00000002450 | 19 |
| GO:0031297 | replication fork processing | 5.88E-04 | 2.50E-02 | 2.29E-02 | ENSAMEG00000002666/ENSAMEG00000005645/ENSAMEG00000001323/ENSAMEG00000002114/ENSAMEG00000004713/ENSAMEG00000002533/ENSAMEG00000013843 | 7 |
| GO:0072686 | mitotic spindle | 7.46E-04 | 3.10E-02 | 2.84E-02 | ENSAMEG00000013678/ENSAMEG00000014099/ENSAMEG00000001093/ENSAMEG00000016924/ENSAMEG00000003520/ENSAMEG00000017084/ENSAMEG00000014825/ENSAMEG00000007168/ENSAMEG00000017390/ENSAMEG00000011481/ENSAMEG00000014201/ENSAMEG00000000492/ENSAMEG00000015353 | 13 |
| GO:0000086 | G2/M transition of mitotic cell cycle | 7.94E-04 | 3.17E-02 | 2.89E-02 | ENSAMEG00000004869/ENSAMEG00000001064/ENSAMEG00000014232/ENSAMEG00000016520/ENSAMEG00000008484/ENSAMEG00000001855/ENSAMEG00000009793/ENSAMEG00000012330 | 8 |
| GO:0000775 | chromosome, centromeric region | 7.94E-04 | 3.17E-02 | 2.89E-02 | ENSAMEG00000014095/ENSAMEG00000016113/ENSAMEG00000009491/ENSAMEG00000005435/ENSAMEG00000017537/ENSAMEG00000002443/ENSAMEG00000001082/ENSAMEG00000016838 | 8 |
| GO:0021987 | cerebral cortex development | 8.95E-04 | 3.43E-02 | 3.13E-02 | ENSAMEG00000011626/ENSAMEG00000017540/ENSAMEG00000003994/ENSAMEG00000006752/ENSAMEG00000005313/ENSAMEG00000000953/ENSAMEG00000006179/ENSAMEG00000000130/ENSAMEG00000002526 | 9 |
| GO:0043588 | skin development | 8.95E-04 | 3.43E-02 | 3.13E-02 | ENSAMEG00000011626/ENSAMEG00000011903/ENSAMEG00000008993/ENSAMEG00000011644/ENSAMEG00000014209/ENSAMEG00000012157/ENSAMEG00000010741/ENSAMEG00000012952/ENSAMEG00000005463 | 9 |
| GO:0048407 | platelet-derived growth factor binding | 9.70E-04 | 3.57E-02 | 3.26E-02 | ENSAMEG00000011626/ENSAMEG00000011903/ENSAMEG00000017486/ENSAMEG00000016642/ENSAMEG00000010741 | 5 |
| GO:0051298 | centrosome duplication | 9.70E-04 | 3.57E-02 | 3.26E-02 | ENSAMEG00000009390/ENSAMEG00000017740/ENSAMEG00000000013/ENSAMEG00000008931/ENSAMEG00000015929 | 5 |
| GO:0007018 | microtubule-based movement | 1.12E-03 | 4.03E-02 | 3.68E-02 | ENSAMEG00000016438/ENSAMEG00000014099/ENSAMEG00000018397/ENSAMEG00000014095/ENSAMEG00000009205/ENSAMEG00000012038/ENSAMEG00000010320/ENSAMEG00000001458/ENSAMEG00000014825/ENSAMEG00000013139/ENSAMEG00000014549/ENSAMEG00000005069/ENSAMEG00000015348/ENSAMEG00000012919/ENSAMEG00000014201 | 15 |
| GO:0001568 | blood vessel development | 1.16E-03 | 4.10E-02 | 3.75E-02 | ENSAMEG00000011626/ENSAMEG00000011903/ENSAMEG00000017486/ENSAMEG00000006908/ENSAMEG00000018055/ENSAMEG00000009123/ENSAMEG00000010741/ENSAMEG00000016747/ENSAMEG00000005699/ENSAMEG00000001699 | 10 |
| GO:0003777 | microtubule motor activity | 1.30E-03 | 4.51E-02 | 4.12E-02 | ENSAMEG00000016438/ENSAMEG00000014099/ENSAMEG00000018397/ENSAMEG00000014095/ENSAMEG00000009205/ENSAMEG00000012038/ENSAMEG00000010320/ENSAMEG00000001458/ENSAMEG00000014825/ENSAMEG00000013139/ENSAMEG00000014549/ENSAMEG00000005069/ENSAMEG00000015348/ENSAMEG00000012919/ENSAMEG00000014201 | 15 |
| GO:0032508 | DNA duplex unwinding | 1.34E-03 | 4.51E-02 | 4.12E-02 | ENSAMEG00000005841/ENSAMEG00000001634/ENSAMEG00000008785/ENSAMEG00000000475/ENSAMEG00000018028/ENSAMEG00000004713/ENSAMEG00000009236/ENSAMEG00000013843/ENSAMEG00000017063/ENSAMEG00000014749/ENSAMEG00000017957 | 11 |
| GO:0051301 | cell division | 1.34E-03 | 4.51E-02 | 4.12E-02 | ENSAMEG00000004841/ENSAMEG00000000346/ENSAMEG00000014358/ENSAMEG00000008950/ENSAMEG00000000371/ENSAMEG00000003344/ENSAMEG00000007868/ENSAMEG00000007193/ENSAMEG00000005084/ENSAMEG00000010291/ENSAMEG00000000983 | 11 |
| GO:0007626 | locomotory behavior | 1.46E-03 | 4.74E-02 | 4.33E-02 | ENSAMEG00000006117/ENSAMEG00000011491/ENSAMEG00000013264/ENSAMEG00000016591/ENSAMEG00000003213/ENSAMEG00000006143/ENSAMEG00000006574/ENSAMEG00000017191/ENSAMEG00000005699/ENSAMEG00000008061/ENSAMEG00000013970/ENSAMEG00000002179 | 12 |
| GO:0005814 | centriole | 1.46E-03 | 4.74E-02 | 4.33E-02 | ENSAMEG00000007989/ENSAMEG00000002675/ENSAMEG00000017945/ENSAMEG00000018213/ENSAMEG00000000013/ENSAMEG00000014232/ENSAMEG00000003927/ENSAMEG00000008931/ENSAMEG00000001860/ENSAMEG00000015126/ENSAMEG00000015514/ENSAMEG00000002356/ENSAMEG00000003208/ENSAMEG00000017415/ENSAMEG00000002966/ENSAMEG00000003767 | 16 |
