# Supplementary material for: Gene expression profiles during postnatal development of the liver and pancreas in giant pandas
Source: Aging (Albany NY). 2020 Aug 15;12(15):15705–29. doi: 10.18632/aging.103783 (PMC7467380; doi:10.18632/aging.103783)
Supplement: Supplementary Table 22 [file aging-12-103783-s008..docx]

**Supplementary Table 22. Significantly enriched KEGG pathways for down-regulated DEGs in pancreas adult group compared with pancreas no feeding group.**

| **ID** | **Description** | **pvalue** | **p.adjust** | **qvalue** | **geneID** | **Count** |
| --- | --- | --- | --- | --- | --- | --- |
| aml04512 | ECM-receptor interaction [PATH:aml04512] | 9.94E-13 | 2.91E-10 | 2.61E-10 | ENSAMEG00000003517/ENSAMEG00000011903/ENSAMEG00000017486/ENSAMEG00000012108/ENSAMEG00000016642/ENSAMEG00000015074/ENSAMEG00000014404/ENSAMEG00000016892/ENSAMEG00000016281/ENSAMEG00000004524/ENSAMEG00000004995/ENSAMEG00000017465/ENSAMEG00000008251/ENSAMEG00000016273/ENSAMEG00000001889/ENSAMEG00000012170/ENSAMEG00000000087/ENSAMEG00000002289/ENSAMEG00000012157/ENSAMEG00000000836/ENSAMEG00000016747/ENSAMEG00000012952/ENSAMEG00000002331/ENSAMEG00000007475/ENSAMEG00000008296/ENSAMEG00000012067/ENSAMEG00000005051/ENSAMEG00000005463/ENSAMEG00000000779/ENSAMEG00000016573 | 30 |
| aml04110 | Cell cycle [PATH:aml04110] | 5.94E-11 | 7.72E-09 | 6.90E-09 | ENSAMEG00000003117/ENSAMEG00000005841/ENSAMEG00000012771/ENSAMEG00000001634/ENSAMEG00000001637/ENSAMEG00000017203/ENSAMEG00000014998/ENSAMEG00000004603/ENSAMEG00000011443/ENSAMEG00000018347/ENSAMEG00000007868/ENSAMEG00000004645/ENSAMEG00000009254/ENSAMEG00000015727/ENSAMEG00000012346/ENSAMEG00000010721/ENSAMEG00000014232/ENSAMEG00000014631/ENSAMEG00000010838/ENSAMEG00000009662/ENSAMEG00000017811/ENSAMEG00000001398/ENSAMEG00000016520/ENSAMEG00000016741/ENSAMEG00000008484/ENSAMEG00000012415/ENSAMEG00000016250/ENSAMEG00000016275/ENSAMEG00000000328/ENSAMEG00000017888/ENSAMEG00000005940/ENSAMEG00000009668/ENSAMEG00000000492/ENSAMEG00000012330 | 34 |
| aml04974 | Protein digestion and absorption [PATH:aml04974] | 7.90E-11 | 7.72E-09 | 6.90E-09 | ENSAMEG00000011626/ENSAMEG00000011903/ENSAMEG00000001303/ENSAMEG00000017486/ENSAMEG00000002184/ENSAMEG00000005540/ENSAMEG00000012108/ENSAMEG00000016642/ENSAMEG00000011644/ENSAMEG00000006555/ENSAMEG00000000748/ENSAMEG00000014368/ENSAMEG00000016892/ENSAMEG00000004524/ENSAMEG00000008251/ENSAMEG00000012170/ENSAMEG00000008339/ENSAMEG00000010741/ENSAMEG00000004392/ENSAMEG00000004573/ENSAMEG00000012067/ENSAMEG00000017991/ENSAMEG00000007610/ENSAMEG00000013732/ENSAMEG00000005330/ENSAMEG00000009890/ENSAMEG00000015861/ENSAMEG00000015459/ENSAMEG00000015089 | 29 |
| aml05206 | MicroRNAs in cancer [PATH:aml05206] | 6.86E-06 | 5.02E-04 | 4.49E-04 | ENSAMEG00000006620/ENSAMEG00000002443/ENSAMEG00000010298/ENSAMEG00000002791/ENSAMEG00000015727/ENSAMEG00000008899/ENSAMEG00000010721/ENSAMEG00000009966/ENSAMEG00000009662/ENSAMEG00000017811/ENSAMEG00000001398/ENSAMEG00000002331/ENSAMEG00000016520/ENSAMEG00000016741/ENSAMEG00000017143/ENSAMEG00000011823/ENSAMEG00000015975/ENSAMEG00000001535/ENSAMEG00000016250/ENSAMEG00000009623/ENSAMEG00000006447/ENSAMEG00000009337/ENSAMEG00000017227/ENSAMEG00000014201/ENSAMEG00000017888/ENSAMEG00000009668/ENSAMEG00000000779/ENSAMEG00000012636 | 28 |
| aml04510 | Focal adhesion [PATH:aml04510] | 1.22E-05 | 7.16E-04 | 6.40E-04 | ENSAMEG00000003517/ENSAMEG00000011903/ENSAMEG00000017486/ENSAMEG00000012108/ENSAMEG00000016642/ENSAMEG00000015074/ENSAMEG00000016892/ENSAMEG00000016281/ENSAMEG00000004524/ENSAMEG00000004995/ENSAMEG00000008251/ENSAMEG00000015727/ENSAMEG00000012170/ENSAMEG00000000087/ENSAMEG00000002289/ENSAMEG00000012157/ENSAMEG00000012004/ENSAMEG00000016747/ENSAMEG00000003514/ENSAMEG00000012952/ENSAMEG00000002331/ENSAMEG00000007475/ENSAMEG00000008296/ENSAMEG00000004952/ENSAMEG00000018407/ENSAMEG00000012067/ENSAMEG00000007297/ENSAMEG00000017191/ENSAMEG00000005051/ENSAMEG00000001535/ENSAMEG00000017017/ENSAMEG00000000779/ENSAMEG00000009273/ENSAMEG00000013280 | 34 |
| aml05222 | Small cell lung cancer [PATH:aml05222] | 4.45E-05 | 1.87E-03 | 1.67E-03 | ENSAMEG00000003517/ENSAMEG00000012108/ENSAMEG00000016642/ENSAMEG00000003344/ENSAMEG00000016892/ENSAMEG00000016281/ENSAMEG00000002289/ENSAMEG00000012157/ENSAMEG00000016747/ENSAMEG00000009662/ENSAMEG00000012952/ENSAMEG00000017811/ENSAMEG00000007475/ENSAMEG00000008296/ENSAMEG00000012067/ENSAMEG00000017888/ENSAMEG00000009668/ENSAMEG00000009273/ENSAMEG00000012330/ENSAMEG00000011912 | 20 |
| aml04024 | cAMP signaling pathway [PATH:aml04024] | 4.46E-05 | 1.87E-03 | 1.67E-03 | ENSAMEG00000016009/ENSAMEG00000019810/ENSAMEG00000007716/ENSAMEG00000010871/ENSAMEG00000015030/ENSAMEG00000002486/ENSAMEG00000000839/ENSAMEG00000002582/ENSAMEG00000012329/ENSAMEG00000008225/ENSAMEG00000000712/ENSAMEG00000000864/ENSAMEG00000017173/ENSAMEG00000006179/ENSAMEG00000002011/ENSAMEG00000003514/ENSAMEG00000006143/ENSAMEG00000000819/ENSAMEG00000014200/ENSAMEG00000004573/ENSAMEG00000014007/ENSAMEG00000002199/ENSAMEG00000002572/ENSAMEG00000003383/ENSAMEG00000016182/ENSAMEG00000018046/ENSAMEG00000013653/ENSAMEG00000017017/ENSAMEG00000016101/ENSAMEG00000013970/ENSAMEG00000009273/ENSAMEG00000013280/ENSAMEG00000016574 | 33 |
| aml05165 | Human papillomavirus infection [PATH:aml05165] | 1.79E-04 | 6.55E-03 | 5.86E-03 | ENSAMEG00000003517/ENSAMEG00000011903/ENSAMEG00000017486/ENSAMEG00000019810/ENSAMEG00000012108/ENSAMEG00000016642/ENSAMEG00000015074/ENSAMEG00000017954/ENSAMEG00000016892/ENSAMEG00000016281/ENSAMEG00000004524/ENSAMEG00000004995/ENSAMEG00000008251/ENSAMEG00000015727/ENSAMEG00000012170/ENSAMEG00000000087/ENSAMEG00000014179/ENSAMEG00000002289/ENSAMEG00000012157/ENSAMEG00000016747/ENSAMEG00000018377/ENSAMEG00000012952/ENSAMEG00000017811/ENSAMEG00000002331/ENSAMEG00000016741/ENSAMEG00000007475/ENSAMEG00000005255/ENSAMEG00000008296/ENSAMEG00000012067/ENSAMEG00000002199/ENSAMEG00000005051/ENSAMEG00000015975/ENSAMEG00000012415/ENSAMEG00000009623/ENSAMEG00000018046/ENSAMEG00000010578/ENSAMEG00000002620/ENSAMEG00000009668/ENSAMEG00000000779/ENSAMEG00000012636/ENSAMEG00000003598/ENSAMEG00000009273/ENSAMEG00000015340 | 43 |
| aml04926 | Relaxin signaling pathway [PATH:aml04926] | 4.06E-04 | 1.32E-02 | 1.18E-02 | ENSAMEG00000011626/ENSAMEG00000011903/ENSAMEG00000017486/ENSAMEG00000019810/ENSAMEG00000012108/ENSAMEG00000016642/ENSAMEG00000016892/ENSAMEG00000016591/ENSAMEG00000003514/ENSAMEG00000012067/ENSAMEG00000002199/ENSAMEG00000002572/ENSAMEG00000013993/ENSAMEG00000001535/ENSAMEG00000010842/ENSAMEG00000018046/ENSAMEG00000017017/ENSAMEG00000013165/ENSAMEG00000009273/ENSAMEG00000002450/ENSAMEG00000016417 | 21 |
| aml03460 | Fanconi anemia pathway [PATH:aml03460] | 6.38E-04 | 1.87E-02 | 1.67E-02 | ENSAMEG00000009390/ENSAMEG00000012174/ENSAMEG00000011341/ENSAMEG00000008785/ENSAMEG00000011892/ENSAMEG00000016051/ENSAMEG00000002114/ENSAMEG00000015826/ENSAMEG00000011534/ENSAMEG00000014832/ENSAMEG00000015491 | 11 |
| aml04914 | Progesterone-mediated oocyte maturation [PATH:aml04914] | 1.09E-03 | 2.91E-02 | 2.60E-02 | ENSAMEG00000003117/ENSAMEG00000002486/ENSAMEG00000004603/ENSAMEG00000014825/ENSAMEG00000007168/ENSAMEG00000010721/ENSAMEG00000014232/ENSAMEG00000014631/ENSAMEG00000003514/ENSAMEG00000001398/ENSAMEG00000016520/ENSAMEG00000002572/ENSAMEG00000009322/ENSAMEG00000016275/ENSAMEG00000017017/ENSAMEG00000009273 | 16 |
| aml04979 | Cholesterol metabolism [PATH:aml04979] | 1.23E-03 | 3.01E-02 | 2.70E-02 | ENSAMEG00000015443/ENSAMEG00000013086/ENSAMEG00000004937/ENSAMEG00000004472/ENSAMEG00000012141/ENSAMEG00000010784/ENSAMEG00000003097/ENSAMEG00000001014/ENSAMEG00000012737/ENSAMEG00000008662/ENSAMEG00000005131 | 11 |
| aml05200 | Pathways in cancer [PATH:aml05200] | 1.41E-03 | 3.17E-02 | 2.83E-02 | ENSAMEG00000003517/ENSAMEG00000019810/ENSAMEG00000009390/ENSAMEG00000012108/ENSAMEG00000016642/ENSAMEG00000016880/ENSAMEG00000006727/ENSAMEG00000014209/ENSAMEG00000003344/ENSAMEG00000013864/ENSAMEG00000010798/ENSAMEG00000016892/ENSAMEG00000016281/ENSAMEG00000005658/ENSAMEG00000015727/ENSAMEG00000006696/ENSAMEG00000000864/ENSAMEG00000002289/ENSAMEG00000012157/ENSAMEG00000002011/ENSAMEG00000003178/ENSAMEG00000016747/ENSAMEG00000009662/ENSAMEG00000018377/ENSAMEG00000003514/ENSAMEG00000012952/ENSAMEG00000017811/ENSAMEG00000006254/ENSAMEG00000006552/ENSAMEG00000001930/ENSAMEG00000010606/ENSAMEG00000007475/ENSAMEG00000008296/ENSAMEG00000012067/ENSAMEG00000002572/ENSAMEG00000013993/ENSAMEG00000003873/ENSAMEG00000015975/ENSAMEG00000016250/ENSAMEG00000009623/ENSAMEG00000010842/ENSAMEG00000017888/ENSAMEG00000010578/ENSAMEG00000017017/ENSAMEG00000009668/ENSAMEG00000005137/ENSAMEG00000006324/ENSAMEG00000003956/ENSAMEG00000006241/ENSAMEG00000012636/ENSAMEG00000003598/ENSAMEG00000009273/ENSAMEG00000009713/ENSAMEG00000002450/ENSAMEG00000012330/ENSAMEG00000011912/ENSAMEG00000016417/ENSAMEG00000016574 | 58 |
| aml03030 | DNA replication [PATH:aml03030] | 1.66E-03 | 3.38E-02 | 3.03E-02 | ENSAMEG00000005841/ENSAMEG00000001634/ENSAMEG00000013454/ENSAMEG00000014998/ENSAMEG00000014758/ENSAMEG00000018028/ENSAMEG00000009236/ENSAMEG00000015091/ENSAMEG00000019971 | 9 |
| aml04911 | Insulin secretion [PATH:aml04911] | 1.73E-03 | 3.38E-02 | 3.03E-02 | ENSAMEG00000019810/ENSAMEG00000002582/ENSAMEG00000017601/ENSAMEG00000019226/ENSAMEG00000003026/ENSAMEG00000014732/ENSAMEG00000004573/ENSAMEG00000002199/ENSAMEG00000002572/ENSAMEG00000005653/ENSAMEG00000011508/ENSAMEG00000018046/ENSAMEG00000016689/ENSAMEG00000002450/ENSAMEG00000020104 | 15 |
| aml04950 | Maturity onset diabetes of the young [PATH:aml04950] | 1.99E-03 | 3.65E-02 | 3.27E-02 | ENSAMEG00000006908/ENSAMEG00000019033/ENSAMEG00000005843/ENSAMEG00000017601/ENSAMEG00000000001/ENSAMEG00000000055 | 6 |
