# Supplementary material for: Gene expression profiles during postnatal development of the liver and pancreas in giant pandas
Source: Aging (Albany NY). 2020 Aug 15;12(15):15705–29. doi: 10.18632/aging.103783 (PMC7467380; doi:10.18632/aging.103783)
Supplement: Supplementary Table 23 [file aging-12-103783-s003..docx]

**Supplementary Table 23. Gene expression values of DEGs involved in metabolism-related terms in the pancreas.**

| **Gene category** | **Ensemble ID** | **Symbol** | **No feeding group (Mean CPM)** | **Suckling group (Mean CPM)** | **Adult group (Mean CPM)** |
| --- | --- | --- | --- | --- | --- |
| Carbohydrate metabolism and energy production related genes | ENSAMEG00000001051 | COX7A1 | 5.82 | 21.10 | 34.47 |
|  | ENSAMEG00000001230 | TCIRG1 | 4.10 | 3.39 | 23.49 |
|  | ENSAMEG00000002182 | ENSAMEG00000002182 | 20.97 | 33.26 | 47.81 |
|  | ENSAMEG00000002294 | NDUFA12 | 9.31 | 22.22 | 32.59 |
|  | ENSAMEG00000002493 | ENSAMEG00000002493 | 52.70 | 127.18 | 132.04 |
|  | ENSAMEG00000003326 | NDUFS7 | 16.14 | 27.73 | 49.86 |
|  | ENSAMEG00000003502 | ENSAMEG00000003502 | 23.16 | 43.04 | 74.00 |
|  | ENSAMEG00000003731 | COX6B2 | 0.28 | 0.63 | 1.15 |
|  | ENSAMEG00000004489 | ENSAMEG00000004489 | 14.64 | 29.93 | 39.08 |
|  | ENSAMEG00000004761 | NDUFB4 | 11.73 | 21.34 | 26.04 |
|  | ENSAMEG00000005090 | ATP6V1F | 14.34 | 40.63 | 40.28 |
|  | ENSAMEG00000005242 | ENSAMEG00000005242 | 46.19 | 85.32 | 114.47 |
|  | ENSAMEG00000006052 | CYC1 | 54.64 | 71.62 | 133.46 |
|  | ENSAMEG00000006153 | COX7B | 50.10 | 106.22 | 126.68 |
|  | ENSAMEG00000006540 | GLRX5 | 13.66 | 28.82 | 30.12 |
|  | ENSAMEG00000007287 | UQCRB | 25.32 | 63.62 | 70.26 |
|  | ENSAMEG00000007918 | ENSAMEG00000007918 | 16.17 | 32.08 | 45.46 |
|  | ENSAMEG00000009115 | ATP5PD | 35.94 | 67.58 | 90.90 |
|  | ENSAMEG00000009142 | FDX1 | 5.66 | 9.89 | 19.16 |
|  | ENSAMEG00000009428 | NDUFA6 | 20.50 | 38.13 | 53.55 |
|  | ENSAMEG00000010344 | NDUFS5 | 28.93 | 59.27 | 88.90 |
|  | ENSAMEG00000010930 | NDUFS6 | 16.73 | 34.29 | 54.53 |
|  | ENSAMEG00000010954 | NDUFA2 | 17.59 | 41.92 | 64.88 |
|  | ENSAMEG00000011040 | NDUFC1 | 6.65 | 15.96 | 19.04 |
|  | ENSAMEG00000011283 | ATP6V0E1 | 18.02 | 41.41 | 43.56 |
|  | ENSAMEG00000011470 | NDUFB7 | 21.08 | 42.79 | 52.04 |
|  | ENSAMEG00000011554 | TXNRD2 | 10.23 | 16.08 | 22.95 |
|  | ENSAMEG00000011765 | ENSAMEG00000011765 | 6.12 | 12.68 | 24.06 |
|  | ENSAMEG00000013441 | COX7C | 54.95 | 96.37 | 161.43 |
|  | ENSAMEG00000013945 | ENSAMEG00000013945 | 51.58 | 84.91 | 131.77 |
|  | ENSAMEG00000014373 | NQO2 | 7.90 | 9.24 | 23.02 |
|  | ENSAMEG00000015234 | PTGES2 | 13.15 | 24.16 | 52.97 |
|  | ENSAMEG00000015852 | NDUFB9 | 32.43 | 52.85 | 108.84 |
|  | ENSAMEG00000017762 | UQCRQ | 27.44 | 64.26 | 89.97 |
|  | ENSAMEG00000017980 | ATP6V0C | 33.91 | 62.32 | 80.18 |
|  | ENSAMEG00000018091 | COX8A | 31.74 | 60.30 | 67.79 |
|  | ENSAMEG00000018274 | ENSAMEG00000018274 | 98.92 | 162.87 | 275.30 |
| **Gene category** | **Ensemble ID** | **Symbol** | **No feeding group (Mean CPM)** | **Suckling group (Mean CPM)** | **Adult group (Mean CPM)** |
| Lipid metabolism related genes | ENSAMEG00000001014 | ENSAMEG00000001014 | 6.36 | 8.79 | 1.74 |
|  | ENSAMEG00000003097 | LIPC | 2.46 | 0.19 | 0.05 |
|  | ENSAMEG00000004472 | TSPO2 | 1.23 | 0.66 | 0.03 |
|  | ENSAMEG00000004937 | ANGPTL4 | 8.35 | 71.13 | 0.67 |
|  | ENSAMEG00000005131 | ABCA1 | 27.41 | 10.30 | 8.78 |
|  | ENSAMEG00000008662 | APOA1 | 267.61 | 178.67 | 77.73 |
|  | ENSAMEG00000010784 | ABCB11 | 1.26 | 0.08 | 0.05 |
|  | ENSAMEG00000012141 | SOAT2 | 2.47 | 0.30 | 0.08 |
|  | ENSAMEG00000012737 | APOC1 | 35.47 | 33.23 | 10.41 |
|  | ENSAMEG00000013086 | ENSAMEG00000013086 | 40.98 | 18.95 | 1.06 |
|  | ENSAMEG00000015443 | APOA2 | 22.96 | 31.61 | 0.05 |
| **Gene category** | **Ensemble ID** | **Symbol** | **No feeding group (Mean CPM)** | **Suckling group (Mean CPM)** | **Adult group (Mean CPM)** |
| Amino acid and protein metabolism related genes | ENSAMEG00000000748 | ENSAMEG00000000748 | 6.21 | 4.01 | 0.59 |
|  | ENSAMEG00000000994 | KNG1 | 11.81 | 0.30 | 11.03 |
|  | ENSAMEG00000001013 | HRG | 10.87 | 9.03 | 55.63 |
|  | ENSAMEG00000001036 | FETUB | 32.58 | 0.02 | 0.37 |
|  | ENSAMEG00000001303 | COL11A1 | 13.35 | 0.65 | 0.24 |
|  | ENSAMEG00000001740 | MME | 0.19 | 0.08 | 3.24 |
|  | ENSAMEG00000002015 | CELA1 | 1185.86 | 29049.67 | 177412.45 |
|  | ENSAMEG00000002069 | KCNQ1 | 20.52 | 20.11 | 128.32 |
|  | ENSAMEG00000002178 | ENSAMEG00000002178 | 26.53 | 5.50 | 47.14 |
|  | ENSAMEG00000002184 | COL14A1 | 56.60 | 16.18 | 7.89 |
|  | ENSAMEG00000002838 | ALDH4A1 | 26.37 | 17.26 | 69.77 |
|  | ENSAMEG00000003271 | ENSAMEG00000003271 | 1.51 | 1.25 | 7.12 |
|  | ENSAMEG00000003623 | GOT1 | 15.96 | 19.90 | 62.24 |
|  | ENSAMEG00000003734 | ENSAMEG00000003734 | 2.22 | 9.19 | 25.87 |
|  | ENSAMEG00000003817 | COL4A3 | 1.31 | 1.29 | 5.34 |
|  | ENSAMEG00000004014 | GLS2 | 2.15 | 2.23 | 326.01 |
|  | ENSAMEG00000004316 | WFDC2 | 25.39 | 33.02 | 86.59 |
|  | ENSAMEG00000004392 | COL15A1 | 23.58 | 11.98 | 5.18 |
|  | ENSAMEG00000004524 | COL9A2 | 3.11 | 1.91 | 0.16 |
|  | ENSAMEG00000004573 | ENSAMEG00000004573 | 1.12 | 0.42 | 0.16 |
|  | ENSAMEG00000005071 | ATP1A1 | 267.72 | 264.89 | 1097.35 |
|  | ENSAMEG00000005330 | DPP4 | 2.51 | 0.30 | 0.73 |
|  | ENSAMEG00000005506 | CPA2 | 561.81 | 3624.20 | 6168.95 |
|  | ENSAMEG00000005540 | ENSAMEG00000005540 | 4452.49 | 1750.85 | 71.45 |
|  | ENSAMEG00000006262 | ENSAMEG00000006262 | 3120.31 | 38740.99 | 141130.92 |
|  | ENSAMEG00000006334 | ENSAMEG00000006334 | 147.19 | 5065.09 | 33566.82 |
|  | ENSAMEG00000006411 | SERPINA5 | 5.50 | 0.65 | 9.86 |
|  | ENSAMEG00000006555 | COL21A1 | 7.59 | 2.69 | 0.73 |
|  | ENSAMEG00000007161 | C3 | 179.76 | 16.41 | 96.05 |
|  | ENSAMEG00000007610 | ENSAMEG00000007610 | 1.06 | 0.44 | 0.16 |
|  | ENSAMEG00000007631 | SLC7A8 | 4.70 | 3.15 | 26.95 |
|  | ENSAMEG00000007695 | GLUL | 49.59 | 33.33 | 107.42 |
|  | ENSAMEG00000007766 | AKT1 | 42.09 | 48.67 | 119.75 |
|  | ENSAMEG00000007927 | ADSS1 | 6.08 | 5.61 | 21.32 |
|  | ENSAMEG00000008201 | ITIH3 | 70.00 | 0.89 | 4.70 |
|  | ENSAMEG00000008251 | COL6A3 | 108.63 | 22.20 | 19.84 |
|  | ENSAMEG00000008339 | SLC9A3 | 7.22 | 8.89 | 0.81 |
|  | ENSAMEG00000009890 | MEP1B | 0.42 | 0.30 | 0.03 |
|  | ENSAMEG00000010741 | COL5A1 | 164.01 | 70.03 | 27.16 |
|  | ENSAMEG00000010830 | ABAT | 1.54 | 0.24 | 2.78 |
|  | ENSAMEG00000010850 | CPB1 | 1384.42 | 10130.19 | 58653.41 |
|  | ENSAMEG00000010873 | CPA3 | 3.36 | 6.98 | 85.61 |
|  | ENSAMEG00000011626 | COL3A1 | 1507.61 | 384.23 | 37.74 |
|  | ENSAMEG00000011644 | COL5A2 | 87.79 | 27.43 | 11.66 |
|  | ENSAMEG00000011903 | COL1A1 | 1635.38 | 366.93 | 24.81 |
|  | ENSAMEG00000012067 | COL4A5 | 31.53 | 17.96 | 13.05 |
|  | ENSAMEG00000012108 | COL4A6 | 12.05 | 3.60 | 1.39 |
|  | ENSAMEG00000012170 | COL9A1 | 1.09 | 1.49 | 0.03 |
|  | ENSAMEG00000012943 | TIMP1 | 11.06 | 15.43 | 39.73 |
|  | ENSAMEG00000013732 | KCNK5 | 2.57 | 3.91 | 0.62 |
|  | ENSAMEG00000014368 | COL12A1 | 64.34 | 17.86 | 6.52 |
|  | ENSAMEG00000014526 | SERPING1 | 29.36 | 13.92 | 127.89 |
|  | ENSAMEG00000014546 | ENSAMEG00000014546 | 5012.02 | 18178.53 | 22717.17 |
|  | ENSAMEG00000015089 | XPNPEP2 | 2.72 | 0.62 | 0.57 |
|  | ENSAMEG00000015459 | SLC7A9 | 1.19 | 0.41 | 0.22 |
|  | ENSAMEG00000015644 | SERPINC1 | 19.12 | 0.28 | 1.19 |
|  | ENSAMEG00000015861 | SLC36A4 | 4.80 | 1.84 | 1.50 |
|  | ENSAMEG00000015921 | ACE2 | 0.79 | 0.37 | 6.62 |
|  | ENSAMEG00000016366 | COL28A1 | 8.04 | 1.09 | 6.02 |
|  | ENSAMEG00000016642 | COL4A1 | 419.22 | 215.24 | 51.15 |
|  | ENSAMEG00000016892 | COL4A2 | 237.97 | 147.84 | 53.17 |
|  | ENSAMEG00000017486 | COL1A2 | 908.39 | 230.97 | 27.03 |
|  | ENSAMEG00000017576 | CTRL | 247.25 | 961.87 | 7332.21 |
|  | ENSAMEG00000017991 | COL17A1 | 1.04 | 0.44 | 0.10 |
|  | ENSAMEG00000020287 | KCNE3 | 8.15 | 6.43 | 54.49 |
